# Short Synthesis of Structurally Diverse *N*-Acylhomoserine Lactone Analogs and Discovery of Novel Quorum Quenchers Against Gram-Negative Pathogens

**DOI:** 10.3390/ijms26041775

**Published:** 2025-02-19

**Authors:** Marina Porras, Dácil Hernández, Alicia Boto

**Affiliations:** Instituto de Productos Naturales y Agrobiología del CSIC, Avda. Astrofísico Fco. Sánchez, 3, 38206 La Laguna, Tenerife, Spain; mporras@ipna.csic.es

**Keywords:** quorum quenchers, acyl homoserine analogs, drug synthesis, in silico ADME

## Abstract

Quorum quenchers are emerging as an alternative to conventional antimicrobials, since they hinder the development of virulence or resistance mechanisms but without killing the microorganisms, thus, reducing the risk of antimicrobial resistance. Many quorum quenchers are analogs of the natural quorum-sensing signaling molecules or autoinducers. Thus, different analogs of natural N-acylhomoserine lactones (AHLs) have been reported for controlling virulence or reducing the production of biofilms in Gram-negative pathogens. Herein we report the preparation of AHL analogs with a variety of N-substituents in just two steps from readily available *N*-substituted hydroxyproline esters. The substrates underwent an oxidative radical scission of the pyrrolidine ring. The resulting *N*-substituted β-aminoaldehyde underwent reduction and in situ cyclization to give a variety of homoserine lactones, with *N*- and *N*,*N*-substituted amino derivatives and with high optical purity. The libraries were screened for the inhibition of violacein production in *Chromobacterium violaceum*, a Gram-negative pathogen. For the first time, *N*,*N-*disubstituted AHL analogs were studied. Several *N*-sulfonyl derivatives, one carbamoyl, and one *N-*alkyl-*N-*sulfonyl homoserine lactone displayed a promising inhibitory activity. Moreover, they did not display microbicide action against *S. aureus*, *C. jejuni*, *S. enterica*, *P. aeruginosa*, and *C. albicans*, confirming a pure QQ activity. The determination of structure–activity relationships and in silico ADME studies are also reported, which are valuable for the design of next generations QQ agents.

## 1. Introduction

Antimicrobial resistance is a major threat to health and food security, according to the WHO and FAO, and it is urgent to develop new treatments [1,2]. Among the alternatives to current antimicrobials, one of the most promising is the discovery of quorum quenchers (QQs), compounds that disrupt the microbial communication systems known as quorum sensing (QS) [3,4,5]. The latter mediates coordinated actions, such as the activation of virulence and coordinated attacks on the host, production of toxins and proteolytic enzymes, swarming, generation of defensive biofilms, and activation of other resistance mechanisms [3,4,5,6,7,8,9,10]. Quorum sensing has been discovered both in bacteria [4,6,7,8] and fungi [9,10,11,12], and there is also an interkingdom communication that modulates the host defensive responses [13,14,15,16,17,18]. Therefore, the molecules that are able to regulate QS could be critical to prevent or reduce pathogenicity [3,4,5,19,20,21,22,23,24,25]. In addition, since pure QQs do not display a microbicidal action, the pressure towards the emergence of resistance is greatly reduced [3,4,5,26].

**Figure 1:**
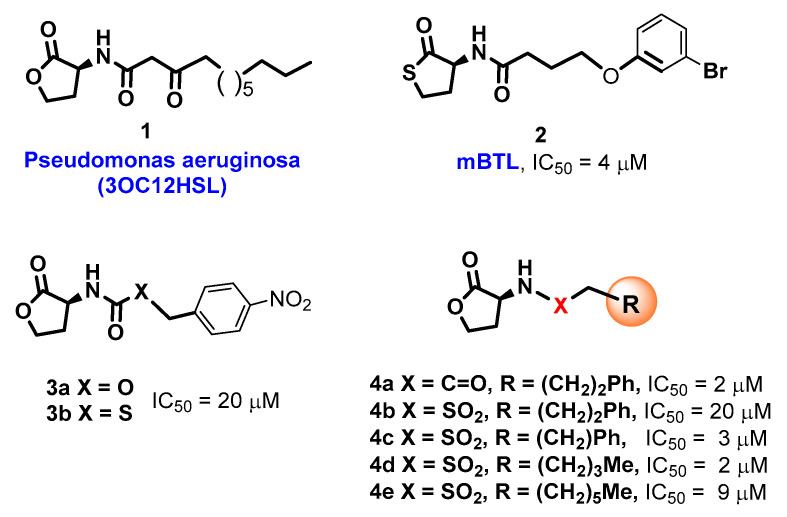
Examples of natural quorum-sensing modulators: the natural agonist **1** and the antagonists **2**–**4**, showing that structural fine-tuning can greatly influence the activity on quorum sensing.

There are many QS systems, but all are mediated by signaling molecules known as autoinducers [3,4,5,6,7,8,9,10,27]. Among the most important QS signals are *N*-acylhomoserine lactones (AHLs), which are produced and/or recognized by Gram-negative bacteria [17,18], including pathogens such as *Escherichia coli*., *Salmonella* sp., or *Pseudomonas aeruginosa* [6,27,28], and they are also involved in interkingdom communication [17]. For instance, homoserine lactone (**1**, 3OC8CHSL, Figure 1) is a quorum-sensing signal in the biofilm-producing pathogen *Pseudomonas aeruginosa* [6], which causes severe complications in cystic fibrosis and contaminates burns, wounds, and even medical devices.

Due to their importance, different AHL analogs have been developed as potential QQs, either by replacing the lactone ring by other hetero- or carbocycles and even a few acyclic chains [29,30,31,32,33,34,35], or by changing the *N*-substituents [36,37,38,39]. In other cases, the natural acyl chains have been replaced by synthetic acyl and 3-oxoacyl chains with unnatural carbocyclic, aromatic, or heteroaromatic groups and also with heteroatoms (e.g., N, S, O) inserted in the alkyl chains [6,36,37,38,39,40,41,42,43,44,45,46]. For instance, Bassler et al. reported that synthetic compound (**2**), mBTL, an analog of compound (**1**) containing a thiolactone ring and a modified acyl chain, inhibited the generation of biofilms and of the virulence factor pyocianin (IC_50_ = 4 µM). Moreover, mBTL protected human epithelial cells and even pluricelullar organisms such as the nematode *Caenorhabditis elegans* from death caused by *P. aeruginosa* [6].

The *N*-acyl group has also been replaced by thiocarbamoyl and carbamoyl groups [47,48], sulfonamides [49,50,51], and sulfonylureas [49,52]. For instance, Queneau and Soulére reported that p-nitrobenzylcarbamate **3a** and its thiocarbamate analog **3b** (Figure 1) had a promising quorum-quenching activity in *Vibrio fischeri*. Both compounds were similarly active with an IC_50_ value of about 20 µM [47]. In another example, different compounds were tested as competitive inhibitors of 3-oxohexanoyl-L-homoserine lactone, the ligand of transcriptional regulator LuxR in *Vibrio fischeri*. The most active compounds inhibited bioluminescence in the pathogen. Amide inhibitor **4a** was taken as reference (IC_50_ = 2 µM). When the amide function was replaced by a sulfonamide (compound **4b**), the activity was considerably reduced. However, when the chain length was slightly decreased (compound **4c**), the activity was similar to the reference compound. Moreover, replacement of the previous chain by a pentenyl group (compound **4d**) also gave good results. Increasing the acyclic chain length (as in compound **4e**) again decreased the inhibitory activity [50]. This example shows how structural fine-tuning can have an important impact in bioactivity.

**Figure 2:**
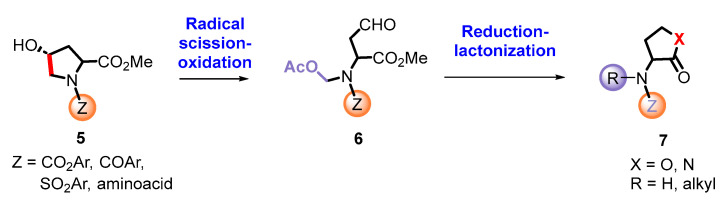
Proposed synthetic route for the conversion of low-cost hydroxyproline derivatives **5** into aldehydes **6** and AHL analogs **7**, as potential quorum quenchers.

Blackwell [30,34,39,40,46], Nagarajan [34], Subba Reddy and Padmajan [49], and other groups have reported important collections of potential quorum-sensing inhibitors. Some of these replacements have yielded potent quorum quenchers that are being evaluated for medical use. These encouraging results have fueled more work in the area. We noticed, for instance, that most sulfonamides are alkylsulfonyl derivatives [49,50], while there are few examples of *N*-arylsulfonyl AHL, mostly *p*-aminophenylsulfonyl derivatives [49,51]. To our surprise, the substituted benzamido groups are also scarcely studied [36,37,38,39,45,46], the same as carbamoyl substituents [47]. Moreover, there were no reports on the activity of *N*,*N*-disubstituted AHLs. In order to study these new derivatives, a versatile synthetic methodology was optimized, starting from low-cost substrates derived from natural 4-hydroxy-L-proline (Hyp, Figure 2). This methodology avoids commercial amino-γ-lactone as a substrate, which is prone to epimerization during the *N*-acylation/functionalization step. Instead, the hydroxyproline derivatives **5** would undergo an oxidative radical fragmentation of the pyrrolidine ring through the C_4_-C_5_ bond, to give aldehydes **6**. This scission was previously reported for *N*-carbamoyl and *N*-acyl derivatives but not for sulfonamides [53,54,55,56,57]. Then, the aldehydes **6** would be transformed into the lactones **7** using a reduction–lactonization reaction. It must be noted that the method reported herein affords both *N*-substituted (X = O, R = H) and *N*,*N*-disubstituted (R, Z ≠ H) amino lactones **7** in a simple way. Moreover, small variations in the proposed synthetic route could afford different heterocycles (e.g., reductive aminations would yield X = *N*-alkyl), although the present work is devoted to the lactones.

## 2. Results and Discussion

The subheadings of this section describe experimental results and their interpretation.

### 2.1. Preparation of Libraries

The libraries were prepared from a variety of *N*-substituted hydroxyproline substrates **5a**–**p** (Table 1). Their conversion into the aldehydes **6a**–**p** has been developed by our group [53,54,55,56,57]. Under treatment with (diacetoxyiodo)benzene (DIB) and iodine, a 4-hypoiodite is formed. Then, irradiation with visible light provides energy for the homolytic cleavage of the O-I bond. The resultant *O*-radical undergoes a regioselective β-fragmentation, and, thus, the C_4_-C_5_ bond is cleaved and a *N*-methyl radical is formed. This radical species is stabilized by the nitrogen function, which accounts for the regioselectivity observed. However, it quickly reacts with iodine generating an unstable N-CH_2_-I moiety. Extrusion of iodide gives intermediate iminium ions **8a**–**p**, which are trapped by acetate ions from the DIB reagent, yielding the aldehydes **6a**–**p** [53,54,55,56,57]. The process took place in good yields, and the resulting products presented an α-chain and an *N*,*O*-acetal, which could be manipulated independently, as shown below.

Interestingly, this is the first comparison of an oxidative radical scission generating an *N*-sulfonyliminium ion and related scissions affording an *N*-acyliminium ion intermediate. The sulfonamides **5a**–**e** gave fragmentation yields similar to the benzamides **5f**–**3j**. The acyl groups with alkyl chains and the carbamoyl groups also gave good, although slightly lower, yields. It should be noted that the scission of substrate **5m** (entry 13) gave only one enantiomer, showing that the scission proceeded without epimerization.

The reduction of the aldehydes and in situ lactonization was carried out under different conditions. Using the standard conditions of sodium borohydride in methanol, the reduction of the aldehyde was accompanied by cleavage of the acetoxymethyl group, to give lactones **7a**–**p** (Table 2). In effect, traces of sodium methoxide formed in situ caused the saponification of the acetate, and the lactonization released more methoxide. Under these conditions, a small epimerization was detected, as evidenced by changes in the optical rotation of the product in processes carried out at different reaction times. In order to avoid it, an optimized process was developed. Thus, after the reduction a quick work-up was performed, the solvent was removed, and the residue was dissolved in dichloromethane, treated with triethylamine and refluxed for 1 h. Under these mild conditions, lactones **7a**–**p** were obtained as shown in Table 2, with reproducible optical rotations, and moreover, the lactonization of substrate **6m** provided a single isomer. The cyclization of the phenyl carbamate **6p** deserves comment, as the cyclization proceeded to the six-membered carbamate **7p** and not to the desired five-membered lactone, thus, demonstrating that the phenoxy function is an excellent leaving group.

**Figure 3:**
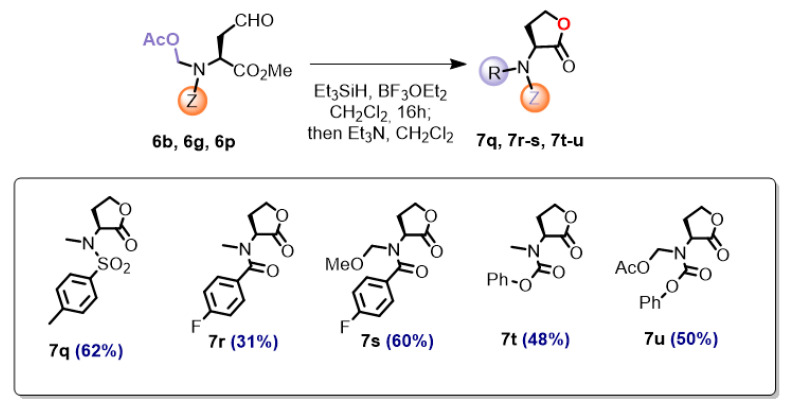
Preparation of *N*,*N*-disubstituted AHL analogs from aldehydes **6b**, **6g**, and **6p** using triethylsilane as a reducing reagent.

The preparation of *N*,*N*-disubstituted homoserine lactones required a different procedure, using our reported conditions for the reduction of *N*-acetoxymethyl groups [56]. The selected aldehyde substrates were the sulfonamide **6b**, the benzamide **6g**, and the carbamate **6p** (Figure 3), as representative examples of the most frequent protecting groups, Z. Therefore, these substrates were treated with triethylsilane in the presence of boron trifluoride etherate. In the non-polar solvent, the *N*,*O*-acetal would generate an iminium ion, which would be reduced by the silane to a *N*-methyl group.

The reduction of sulfone **6b** afforded the *N*-methyl lactone **7q** in 62% yield. To our surprise, in the case of benzamide substrate **6g** the *N*-methyl ester was obtained as the minor product (31%), the major being the *N*,*O*-acetal **7s** (60%). The carbamate substrate **6p** yielded a mixture of the *N*-methyl **7t** and *N*-acetoxymethyl **7u** products in a 1:1 ratio (98% yield).

These results point to a probable mechanism of the process. As shown for compounds **7t/1u**, the lactone is formed first to give compound **7u**, and then the acetoxymethyl group is transformed into an imine, which is reduced to an *N*-methyl group, affording compound **7t**. Since the conversion of **7u** into **7t** was incomplete, a mixture of the two compounds was isolated. In the case of the sulfonamide substrate **6b**, the reaction was completed to give only compound **7q**. The sulfonyl protecting group makes the imine intermediate more electrophilic than the carbamate-protected imine and, therefore, more reactive with the silane. In the case of benzamides **7r**/**7s**, an intermediate *N*-acetoxymethyl lactone similar to **7u** is likely formed. Then, the *N*-acetoxymethyl group evolves to an imine, which is either reduced to an *N*-methyl group (compound **7r**) or trapped by methoxy ions to give the methoxy derivative **7s**. The methoxy ions are formed from the methyl ester during the intramolecular lactonization reaction.

The introduction of R = alkyl likely alters interactions with the biological target with respect to *N*-monosubstituted AHLs (R = H), allowing interesting structure–activity relationships to be determined.

In summary, a library of AHLs with a variety of *N*-substituents and a library of AHL aldehyde precursors were prepared in good yields and from readily available, low-cost hydroxyproline substrates. The evaluation of their quorum-quenching and antimicrobial activities is detailed below.

### 2.2. Evaluation of Quorum Quencher and Antimicrobial Activities

The purpose of the libraries is to identify a compound with quorum-quenching activity but not bactericidal action, so that it can prevent bacterial infections but avoid damage to these microorganisms. In this way, the risk of eliciting antibiotic resistance is greatly reduced and the beneficial microbiota is spared. Therefore, the best quorum quenchers should display negligible antibiotic action.

**Figure 4:**
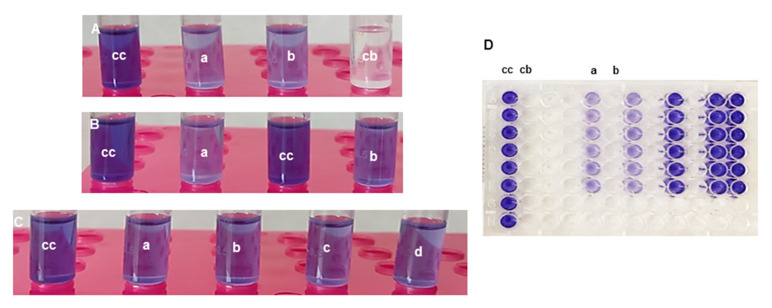
Images after pigment extraction. (**A**) Treatment at 200 μM: (cc) control without treatment, (a) treatment with product **1a** and (b) treatment with **1o**, (cb) blank. (**B**) Treatment with product **1a**: (cc) control without treatment, (a) treatment at 200 μM, and (b) treatment at 100 μM. (**C**) Treatment at 50 μM: (cc) control, (a) treatment with **1a**, (b) treatment with **1b**, (c) treatment with **1c**, and (d) treatment with **1o**. (**D**) Treatment at 200 μM: (cc) control, (cb) blank, (a) treatment with **1a** and (b) treatment with **1o**.

To determine the quorum-quenching activity, the reporter strain *Chromobacterium violaceum* CECT 494 (also called ATCC 12472) was used [18,51,58,59]. This Gram-negative pathogen has a LuxIR-type circuit, called CviIR, which regulates the production of the autoinducer *N*-decanoyl homoserine lactone (C10-HSL) [58,59]. When the autoinducer released into the extracellular space reaches a certain threshold, it re-enters the cytoplasm and binds to the transcriptional activator CviR, activating the expression of genes necessary for the production of the pigment violacein [13,58,59,60]. Therefore, treatment of the CECT 494 strain with quorum quenchers will decrease the generation of the violet pigment, which could be measured by a colorimetric assay (Figure 4), according to the procedure reported by Choo et al. [60].

The results are shown in Table 3, with respect to an untreated control. In addition, since the benzylcarbamate **7o** is a known quorum quencher, it was used as a positive control [47] while the known inactive *N*-acetyl derivative **7l** was used as a negative control [61]. It should be said that although **7l** possesses the *N*-acyl homoserine lactone moiety, the size of the *N*-acyl chain is small, causing the loss of activity. In contrast, many homoserine lactones with bulkier *N*-substituents (such as **1o)** are usually active for a variety of Gram-negative bacteria, such as *Chromobacterium violaceum*, *Vibrio fischeri*, *Escherichia coli*, etc. 

The most active compounds were the sulfonamides **7a**–**c** and **7e**, the reference compound benzyl carbamate **7o**, and the *N*-methyl toluenesulfonamide **7q**. It was ruled out that the reduction in pigment production was due to growth inhibition, since the count of viable colony-forming units gave similar values for the treated biosensor and the untreated control.

The most potent sulfonamides were studied at three doses of 200, 100, and 50 µM. Compound **7a** achieved about 70% inhibition of violacein production at 200 µM, but even when the dose was successively halved, the inhibition did not decrease proportionally but was maintained at satisfactory levels (57% and 42% for 100 and 50 µM). Compound **7b**, which displayed 62% inhibition at 200 µM, also maintained a good activity when the dose was reduced (50% and 32% for 100 and 50 µM). Interestingly, the halo derivatives did not increase inhibition, although the *p*-chlorophenylsulfonamide **7c** also displayed a satisfactory activity at 200 µM (51% inhibition), which was only slightly reduced at 100 µM (44%). The *p*-iodophenylsulfonamide **7d** had a much lower activity, perhaps because the steric hindrance of the iodo group complicated the interaction with the Lux receptor. In fact, comparing **7a** and **7b**, it is clear that the *p*-alkyl substituent led to lower activity. When the *p*-substituent was the nitro group (compound **7e**), the activity was somewhat recovered (49% at 200 µM but 37% at 100 µM).

In contrast to the sulfonamides, the benzamides **7f**–**m** displayed little quorum-quenching activity. However, the reference benzyl carbamate **7o** displayed the second best inhibition (67%, 52%, and 44% at 200, 100, and 50 µM, respectively). The activity dropped when the bulky *t-*butyl carbamate **7n** was used (30% at 200 µM).

With respect to the role of *N*-substitution, when toluenesulfonamide **7b** was compared with its *N*-methyl analog **7q**, it was observed that higher substitution decreased activity (62% for 1b and 44% for 1s at 200 µM). The same happened when the benzamide **7g** was compared with the *N*-methyl analog **7r** (26% vs. 12% inhibition at 200 µM). When the *N*-methyl group was replaced by a bulkier *N*-methoxymethyl moiety, the activity was further reduced (about 8% inhibition at 200 µM). The substituted phenyl carbamoyl derivatives **7t** and **7u** displayed little quorum-quenching activity, supporting that *N*-substitution is deleterious for quorum quenching.

**Figure 5:**
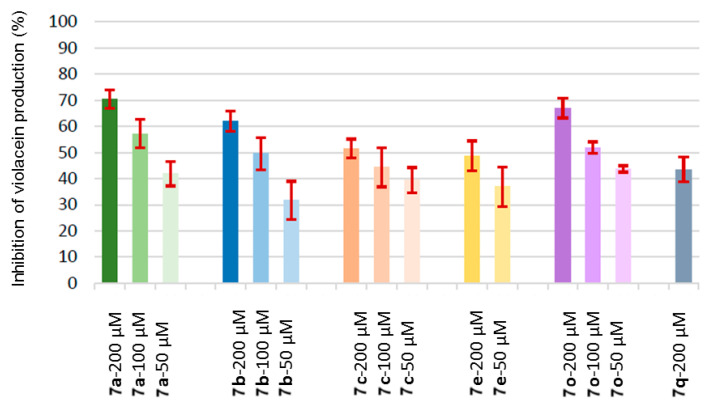
Representation of the percentage of inhibition in violacein production, *C. violaceum* CECT 494 strain. All values show significant differences with the untreated control according to the one-way ANOVA statistical procedure.

A summary of the dose–effect relationship for the most promising compounds (sulfonamides **7a**–**c** and **7e**, benzyl carbamate **7o**, and the *N*-methyl toluenesulfonamide **7q**) is shown in Figure 5. As commented before, the inhibitory effect slowly decreases upon lowering the dose, but a satisfactory activity is, nevertheless, maintained.

As commented on in the introduction, different groups have worked on the development of quorum-sensing modulators, and many inhibitors have been discovered. However, active work takes place in the area. Most of the work on sulfonamides has been carried out with alkylsulfonyl derivatives [49,50], as shown by compounds **4b**–**4e** in Figure 1 [50], where small changes in the chain length and substituents can notably alter the quorum-quenching activity. In contrast, there are few examples of the *N*-arylsulfonyl AHL [49,51], mostly *p*-amidophenylsulfonyl derivatives with bulky *N*-acyl substituents [51]. However, Reddy and Padmaja report the *p*-toluene sulfonamide **7b** and the *p*-nitrophenyl derivative **7e**, as well as the *p*-amino analog of **7e** [49]. The halogenated derivatives **7c**–**d** and the unsubstituted compound **7a** were not tested. The only compound with significative QSI activity was **7b**, which matches our results where **7b** had a considerable quorum-quenching activity, quite superior to **7e**. However, our results show that a simpler sulfonamide **7a** is the most potent derivative, and more importantly, that a considerable activity is retained when the dose is reduced. As commented on later, there are other advantages with respect to **7b**: the lack of antimicrobial activity for the tested Gram-negative and Gram-positive pathogens and a low risk of eliciting antimicrobial resistance.

For the first time, very related sulfonamides and benzamides are compared, with the first displaying a promising activity, in contrast with the second, whose inhibitory activity was quite low. It is interesting that while the literature reports many examples of *N*-acyl homoserine lactones, including examples where alkyl chains are attached to aromatic groups [36,37,38], the *N*-benzoyl derivatives are scarcely reported [39,45,46]. Blackwell et al. carried out the most complete study for benzamides [39,46], with a p-bromo benzamide being a potent QscR antagonists in *P. aeruginosa* [46]. However, when a set of benzamides was studied as potential quorum-sensing modulators in *E. coli*, the benzamides were among the few compounds that did not activate the promiscuous SdiA receptor [39]. In our case, the benzamides had little activity on the CviR receptor in *C. violaceum*, even the p-chloro and p-iodo derivatives **7h** and **7i**. Therefore, the sulfonamide derivatives are preferred to the benzamides.

The carbamoyl and thiocarbamoyl substituents have received some attention, as commented on in the Introduction for *Vibrio fischeri* [47]. Interestingly, both types of carbamate displayed similar activity in the reported examples by Queneau et al. The benzyl carbamates (and particularly the p-nitrobenzyl derivatives) were the most potent [47], as in our case, while the t-butyl carbamate showed a relatively small activity.

Finally, for the first time, this article compares the activity of *N*-methyl derivatives with the demethylated products (7q vs. 7b), showing that the second gave superior inhibition. These results support that the binding of the compounds to the quorum-sensing receptor CviR is enhanced by a hydrogen bond between the N-H group and the receptor. *N*,*N*-disubstituted AHL analogs would lack the ability to form this bond, and the interaction would decrease.

The antibiotic activity was then checked. As commented before, it was observed that the lactones did not affect bacterial growth and the number of colony-forming units of *C. violaceum*. However, they could have antimicrobial activity against other microorganisms, in particular the sulfonamide derivatives. Therefore, the broth microdilution method [62,63] was used to identify the compounds that at 200 μM presented activity against the Gram-positive pathogen *Staphylococcus aureus* CECT 794 and the Gram-negative bacteria *Campylobacter jejuni* CECT 9112, *Salmonella enterica* CECT 456, and *Pseudomonas aeruginosa* CECT108 (Table 4). None of the compounds displayed a minimum bactericidal concentration (MBC) or minimum inhibitory concentration (MIC) below 200 μM against *S. enterica* and *P. aeruginosa*. The *p*-nitrophenylsulfonamide lactone **7e** presented an MIC 101–150 μM against *S. aureus*, and the dinitrobenzamide compound **7k** presented an MIC in the range 155–199 μM against *S. aureus* and *C. jejuni*. This low antimicrobial activity is a requisite for pure QQ agents. To our satisfaction, the most active QQs **7a**–**c**, **7e**, **7o**, and **7q** had negligible direct antimicrobial action.

The aldehyde precursors **6b**–**p** were also tested using EUCAST strains [64,65] as shown in Table 4. All the sulfonamides **6b**–**e** displayed a promising activity against *S. aureus* and *C. jejuni*, although still inferior to the antibiotic standard (tetracycline). The dinitrobenzamide **6k** was also active against *S. aureus* and *C. jejuni.* None of the aldehydes displayed activity against *S. enterica* and *P. aeruginosa*.

The contrast between the antimicrobial activity of the lactones **7b**–**e** and the aldehydes **6b**–**e** suggest that the 4-carbonyl group in the latter interacts with nucleophilic moieties in the receptors. The study of the antimicrobial activity of these aldehydes is in course and will be published in due time.

### 2.3. In Silico ADME Study of the AHL Analogs

In order to determine whether the lactones and the most active aldehydes had appropriate ADME properties, an in silico study was carried out using the SwissADME tool (www.swissadme.ch), accessed on 8 February 2025 [66,67]. The results are shown in Table 5 and Table 6. Table 5 is devoted to the molecular and physicochemical descriptors, such as the MW, number of rotable bonds, H-acceptors, and H-donors, as well as the Topological Polar Surface (TPSA) [68], a useful descriptor to estimate properties such as absorption, brain access, etc., as commented on later. 

Another important parameter is log P_o/w_, the partition coefficient of the compound in its neutral form between water and n-octanol, which is critical for barrier crossing and biodistribution. Since SwissADME provides different values obtained from different calculation methods (iLOGP, XLOGP3, WLOGP, MLOGP, and SILICOS-IT), an average “consensus” value is shown in the table [69,70,71,72]. Most of the compounds have a positive Log P and are, therefore, lipophilic. Only the dinitrobenzamide **7l** and the acetamide **6k** have negative LogP and, thus, have more hydrophilic characteristics.

The solubility can also be calculated using different methods, but the table shows LogS obtained with SILICOS-IT, a fragmental method, which has a high linear correlation between theoretical and experimental values (R^2^ = 0.75) [73]. Most compounds have calculated logS values between 0 and −4 and, therefore, should be soluble or very soluble, and only the dipeptide **7m** presents a value between −4 and −6 (moderately soluble).

Table 6 displays the estimated pharmacokinetic parameters/properties and the druglikeness. The gastrointestinal absorption for most compounds was estimated to be high (white in the BOILED-Egg method) [67], except for the *p*-nitrosulfonamide aldehyde **6e** and the dinitroderivatives **7k** and **6k**. As for the ability to cross the Blood–Brain Barrier (BBB), most compounds could not cross it, with the exception (yolk of the BOILED-Egg method) of unsubstituted or halo-substituted benzamides **7f**–**i**, the benzylcarbamate derivative **7o**, and the *N*-alkyl derivatives **7q**–**7t**, which could be very interesting to fight bacterial infections causing meningitis.

SwissADME also predicts whether a compound can be a substrate for the permeability glycoprotein (P-gp, an ABC transporter) and, therefore, undergo active efflux through membranes, such as the BBB or the GI wall to the lumen [74]. Most of the compounds are not expected to be substrates, with the exception of the acetamide **6l**, the dipeptide **6m**, and the aldehydes **6e**–**2k**. However, lipophilic compounds may cross barriers in a passive way, as commented before for BBB-permeable compounds.

The interaction of the compounds with cytochromes (CYP) is key for their metabolic transformation and subsequent elimination [75]. There are different CYP isoforms such as CYP1A2, CYP2C19, CYP2C9, CYP2D6, and CYP3A4. Their inhibition promotes unwanted drug–drug interactions, accumulation of the drug or its metabolites, and related side-effects. Fortunately, most AHLs were predicted not to inhibit any of the major isoforms, with the exception of halo derivatives **7d**, **7h**, and **7i**, the dipeptide **7m**, and the *N*-methyl derivative **7q**, but in those cases, only one isoform was expected to be affected.

Skin permeation (in cm/s, topical use) was also calculated. When the log Kp exceeds −2.5 cm/s, the molecule presents low skin permeation, which is the case for the studied AHLs and aldehydes. This could be positive for fighting topical infections without causing systemic/intradermal effects [76].

Druglikeness is a qualitative assessment of the oral bioavailability of a drug candidate. Five filters were applied: the Lipinski (Pfizer) [72], Ghose (Amgen) [77], Veber (Glaxo-Smith-Kline) [78], Egan (Pharmacia) [79], and Muegge (Bayer) [80]. The Lipinski and Veber filters are the best known, and the first was implemented as follows: MW < 500, MLOGP < 415, N or O < 10, NH or OH < 5. In addition, the Veber filter requires that n° rotational bonds < 10 and TPSA < 140. In the Ghose filter, 160 < MW < 480 and −0.4 < WLOGP < 5.6, and the number of atoms should be in the range 20–70. The Muegge filter requires that 200 < MW < 600, the number of H-bond acceptors < 10, the number of rotable bonds < 15, TPSA < 150, and −2 < XLOGP < 5. Finally, the Egan filter determines that WLOGP < 5.88 and TPSA < 131.6.

All the compounds met Lipinski’s rules, and only the dinitrobenzamide compound **7k** did not meet the Veber and Egan rules due to the high TPSA value. Finally, some compounds (**7l**, **7p**) had a low MW for the Muegge and Ghose rules, and aldehydes **6e** and **6k** did not meet several criteria, such as suitable TPSA range, number of heteroatoms and rotors, etc. In general, most compounds showed good druglikeness.

The Abbot Bioavailability Score is used to predict the probability of presenting at least 10% oral bioavailability in rat or Caco-2 permeability [81]. Since all the compounds had a 0.55 or higher score, this oral bioavailability criteria was met.

The last column is devoted to PAINS [82] and Brenk [83] alerts. PAINS (pan assay interference compounds) refer to promiscuous compounds that give strong responses in assays for a variety of protein targets (and, therefore, false positives). To our satisfaction, no PAINS alerts were obtained.

The Brenk alarm points out chemical moieties that are known to cause toxicity or instability or those that are dyes. The alarm was obtained for nitrobenzene derivatives (as potential carcinogens), aldehydes (reactive electrophilic moiety), or iodo aromatic groups (depending on the dose may interfere with thyroid function). Since the ester groups may be hydrolyzed by proteases, reducing in vivo stability, some compounds with more than 2 ester groups elicited alarms. It must be said, however, that the Brenk alerts are orientative and do not exclude these compounds from pharmaceutical development; they simply recommend further toxicology or stability assays at early stages. Fortunately, many of our active compounds showed no alerts at all.

**Figure 6:**
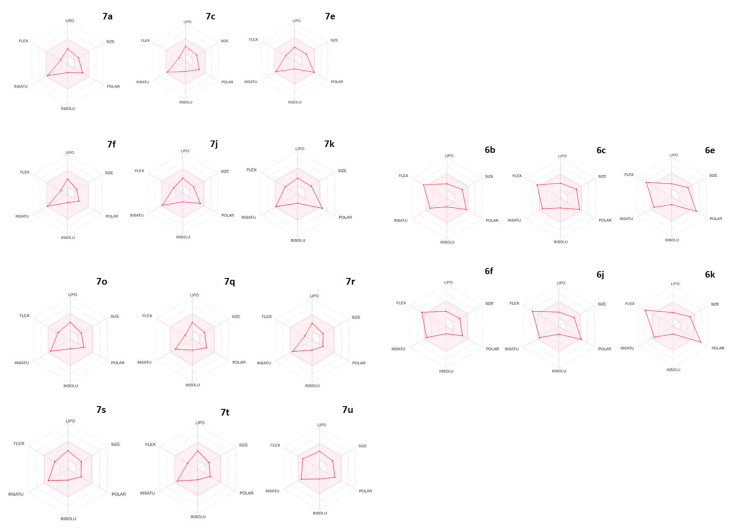
Representations of oral bioavailability of selected compounds; a good one corresponds to products in the pink area. From the top and clockwise, the web points read: LIPO (liposolubility), SIZE, POLAR, INSOLU (insolubility), INSATU (insaturation degree), and FLEX (flexibility).

Finally, Figure 6 shows the Bioavailability Radar of selected compounds, namely a representation of the oral bioavailability based on their molecular and physicochemical properties. The compounds with predicted good oral availability should fall in the pink area and, therefore, would have MW between 150 and 500 g/mol, TPSA in the 20–130 Å^2^ range, logS < 6, a maximum of 9(10) rotatable bonds for optimum flexibility, and suitable lipophilicity (XLOGP3 between −0.7 and + 5.0). The sulfonamide and benzamide AHL derivatives met this requirement, except for dinitro compound **7k**, which was slightly more polar than recommended. The benzyl carbamate **7o** and the *N*-alkyl derivatives **7q**–**u** also met the criteria. However, aldehydes were predicted to not be orally available, due to their high flexibility, and in some cases (**6e**, **6k**) also because they were too polar.

## 3. Materials and Methods

### 3.1. Synthetic Procedures and Characterization Data

**General Methods**. Commercially available reagents and solvents were analytical grade or were purified by standard procedures prior to use. All reactions involving air- or moisture-sensitive materials were carried out under a nitrogen atmosphere. Melting points were determined with a hot-stage apparatus and are uncorrected. Optical rotations were measured at the sodium line at ambient temperature (26 °C) in CHCl_3_ solutions. NMR spectra were determined at 500 or 400 MHz for ^1^H and 125.7 or 100.6 MHz for ^13^C, at 25 °C or 70 °C, as stated for each case. Sometimes, due to slower rotamer interconversion at 26 °C, two (or more) sets of signals are visible at room temperature, while only one set of signals (rotamer average) is seen at 70 °C, due to faster rotamer interconversion. For some compounds, the ^1^H NMR spectra shows some signals as **broad bands** (br b) due to equilibria between rotamers.

^1^H NMR spectra are reported as follows: s = singlet, d = doublet, t = triplet, dd = doublet of doublets, ddd = doublet of doublet of doublets, q = quartet, m = multiplet, br = broad, br b = broad band, and br s = broad singlet; coupling constant(s) were in Hz. Mass spectra were carried out using electrospray ionization techniques (ESI). Merck silica gel 60 PF_254_ and 60 (0.063–0.2 mm) were used for preparative thin-layer chromatography and column chromatography, respectively. The reagent for TLC analysis was KMnO_4_ in NaOH/K_2_CO_3_ aqueous solution, and the TLC was heated until the development of color.

The preparation of substrates **5a**–**5p** is commented on in the Supplementary Materials. Compounds **5f** [84], **5l** [57], and **5m** [57] have been previously reported. Compounds **5n** and **5o** are commercial products. The synthesis of the aldehydes **6a**–**p** and the lactones **7a**–**u** is described below.

**General Procedure for the synthesis of aldehydes by oxidative radical scission of *N*-substituted hydroxypyrrolidines.** To a solution of the *N*-substituted hydroxypyrrolidine (1.0 mmol) in dry dichloromethane (20 mL), iodine (127.0 mg, 0.50 mmol) and PhI(OAc)_2_ (644.0 mg, 2.0 mmol) were added. The resulting mixture was stirred for 30–90 min at 26 °C under irradiation with visible light (cool white LED lamp). Then, the reaction mixture was poured into 10% aqueous Na_2_S_2_O_3_ (10 mL) and extracted with CH_2_Cl_2_. The organic layer was dried over sodium sulfate, filtered, and concentrated under vacuum. The residue was purified by chromatography on silica gel (hexanes/ethyl acetate) to give the scission products **2a**−**2p**.

**Methyl (2*S*)-*N*-(acetoxymethyl)-*N*-(phenylsulfonyl)-4*-*oxo-L-homoalanine (6a).** Obtained from *N*-phenylsulfonyl-L-hydroxyproline **5a** (228.0 mg, 0.80 mmol) according to the general pyrrolidine scission procedure. After work-up and solvent evaporation, the residue was purified by radial chromatography (hexanes/EtOAc, 60:40), yielding aldehyde **6a** (218.6 mg, 0.64 mmol, 80%) as a colorless viscous oil. [α]_D_: −29 (*c* 0.45, CHCl_3_). IR (CHCl_3_) ν_max_ 3023, 1745, 1448, 1437 cm^−1^. ^1^H NMR (500 MHz, CDCl_3_, 26 °C) δ_H_ 9.63 (s, 1H), 7.88 (br d, *J* = 9.0 Hz, 2H), 7.62 (t, *J* = 7.8 Hz, 1H), 7.53 (t, *J* = 7.4 Hz, 2H), 5.60 (d, *J* = 12.4 Hz, 1H), 5.39 (d, *J* = 12.4 Hz, 1H), 5.08 (t, *J* = 6.9 Hz, 1H), 3.58 (s, 3H), 3.21 (ddd, *J* = 18.3, 7.4, 0.8 Hz, 1H), 2.88 (ddd, *J* = 18.1, 6.5, 1.0 Hz, 1H), 1.94 (s, 3H). ^13^C RMN (125.7 MHz, CDCl_3_, 26 °C) δ_C_ 197.4 (CH), 170.1 (C), 169.7 (C), 139.7 (C), 133.5 (CH), 129.1 (2 × CH), 127.8 (2 × CH), 71.1 (CH_2_), 54.4 (CH), 53.0 (CH_3_), 44.6 (CH_2_), 20.8 (CH_3_). HRMS (ESI) calculated for C_15_H_21_NO_8_SNa [M + MeOH + Na]^+^ 398.0886, found 398.0878. Anal. Calcd for C_14_H_17_NO_7_S: C, 48.97; H, 4.99; N, 4.08; S, 9.34. Found: C, 49.09; H, 4.98; N, 4.04; S, 9.04.

**Methyl (2*S*)-*N*-(acetoxymethyl)-*N*-(toluenesulfonyl)-4*-*oxo-L-homoalanine (6b).** Obtained from *N*-toluensulfonyl-L-hydroxyproline **5b** (228.0 mg, 0.80 mmol) according to the general pyrrolidine scission procedure. After work-up and solvent evaporation, the residue was purified by radial chromatography (hexanes/EtOAc, 60:40), yielding aldehyde **6b** (156.5 mg, 0.44 mmol, 73%) as a yellow oil. [α]_D_: −39 (*c* 0.86, CHCl_3_). IR (CHCl_3_) ν_max_ 3029, 1744, 1365, 1352 cm^−1^. ^1^H NMR (500 MHz, CDCl_3_, 26 °C) δ_H_ 9.62 (s, 1H), 7.75 (d, *J* = 8.4 Hz, 2H), 7.31 (d, *J* = 7.9 Hz, 2H), 5.60 (d, *J* = 12.4 Hz, 1H), 5.37 (d, *J* = 12.2 Hz, 1H), 5.05 (t, *J* = 6.9 Hz, 1H), 3.60 (s, 3H), 3.19 (dd, *J* = 18.1, 7.2 Hz, 1H), 2.86 (dd, *J* = 18.1, 6.4 Hz, 1H), 2.43 (s, 3H), 1.95 (s, 3H). ^13^C RMN (125.7 MHz, CDCl_3_, 26 °C) δ_C_ 197.5 (CH), 170.2 (C), 169.8 (C), 144.5 (C), 136.8 (C), 129.7 (2 × CH), 127.9 (2 × CH), 71.2 (CH_2_), 54.4 (CH), 53.0 (CH_3_), 44.7 (CH_2_), 21.7 (CH_3_), 20.9 (CH_3_). HRMS (ESI) calculated for C_16_H_23_NO_8_SNa [M + MeOH + Na]^+^ 412.1042, found 412.1050. Anal. Calcd for C_15_H_19_NO_7_S: C, 50.41; H, 5.36; N, 3.92; S, 8.97. Found: C, 50.28; H, 5.25; N, 4.24; S, 8.92.

**Methyl (2*S*)-*N*-(acetoxymethyl)-*N*-(*p*-chlorophenylsulfonyl)-4*-*oxo-L-homoalanine (6c).** Obtained from *N*-(*p*-chlorophenylsulfonyl)-L-hydroxyproline **5c** (255.2 mg, 0.80 mmol) according to the general pyrrolidine scission procedure. After work-up and solvent evaporation, the residue was purified by radial chromatography (hexanes/EtOAc, 70:30), yielding aldehyde **6c** (220.4 mg, 0.58 mmol, 73%) as a colorless oil. [α]_D_: −24 (*c* 0.34, CHCl_3_). IR (CHCl_3_) ν_max_ 3022, 1746, 1397, 1356, 1167 cm^−1^. ^1^H NMR (500 MHz, CDCl_3_, 26 °C) δ_H_ 9.65 (br s, 1H), 7.83 (br d, *J* = 8.3 Hz, 2H), 7.50 (br d, *J* = 8.9 Hz, 2H), 5.58 (d, *J* = 11.8 Hz, 1H), 5.36 (d, *J* = 12.5 Hz, 1H), 5.07 (t, *J* = 6.9 Hz, 1H), 3.60 (s, 3H), 3.23 (ddd, *J* = 18.2, 7.1, 0.8 Hz, 1H), 2.91 (ddd, *J* = 18.2, 6.5, 1.0 Hz, 1H), 1.94 (s, 3H). ^13^C RMN (125.7 MHz, CDCl_3_, 26 °C) δ_C_ 197.2 (CH), 170.1 (C), 169.7 (C), 140.1 (C), 138.2 (C), 129.38 (2 × CH), 129.36 (2 × CH), 70.9 (CH_2_), 54.5 (CH), 53.1 (CH_3_), 44.6 (CH_2_), 20.8 (CH_3_). HRMS (ESI) calculated for C_15_H_20_ClNO_8_SNa [M + MeOH + Na]^+^ 432.0496, found 432.0508. Anal. Calcd for C_14_H_16_ClNO_7_S: C, 44.51; H, 4.27; N, 3.71; S, 8.49. Found: C, 44.31; H, 3.88; N, 3.60; S, 8.30.

**Methyl (2*S*)-*N*-(acetoxymethyl)-*N*-(*p*-iodophenylsulfonyl)-4*-*oxo-L-homoalanine (6d).** Obtained from *N*-(*p*-iodophenylsulfonyl)-L-hydroxyproline **5d** (328.8 mg, 0.80 mmol) according to the general pyrrolidine scission procedure. After work-up and solvent evaporation, the residue was purified by radial chromatography (hexanes/EtOAc, 60:40), yielding aldehyde **6d** (320.6 mg, 0.68 mmol, 86%) as a yellow oil. [α]_D_: −39 (*c* 0.34, CHCl_3_). IR (CHCl_3_) ν_max_ 3027, 2955, 2847, 1747, 1570 cm^−1^. ^1^H NMR (500 MHz, CDCl_3_, 26 °C) δ_H_ 9.66 (s, 1H), 7.89 (br b, *J* = 8.7 Hz, 2H), 7.60 (br b, *J* = 8.7 Hz, 2H), 5.58 (d, *J* = 12.4 Hz, 1H), 5.37 (d, *J* = 12.4 Hz, 1H), 5.06 (t, *J* = 6.9 Hz, 1H), 3.61 (s, 3H), 3.23 (dd, *J* = 17.9, 7.2 Hz, 1H), 2.92 (dd, *J* = 18.2, 6.5 Hz, 1H), 1.95 (s, 3H). ^13^C RMN (125.7 MHz, CDCl_3_, 26 °C) δ_C_ 197.2 (CH), 170.1 (C), 169.6 (C), 139.4 (C), 138.4 (2 × CH), 129.2 (2 × CH), 101.1 (C), 70.9 (CH_2_), 54.5 (CH), 53.1 (CH_3_), 44.6 (CH_2_), 20.8 (CH_3_). HRMS (ESI) calculated for C_15_H_20_INO_8_SNa [M + MeOH + Na]^+^ 523.9852, found 523.9852. Anal. Calcd for C_14_H_16_INO_7_S: C, 35.83; H, 3.44; N, 2.98; S, 6.83. Found: C, 35.57; H, 3.47; N, 2.93; S, 6.78.

**Methyl (2*S*)-*N*-(acetoxymethyl)-*N*-(*p*-nitrophenylsulfonyl)-4*-*oxo-L-homoalanine (6e).** Obtained from *N*-(*p*-nitrophenylsulfonyl)-L-hydroxyproline **5e** (204.6 mg, 0.60 mmol) according to the general pyrrolidine scission procedure. After work-up and solvent evaporation, the residue was purified by radial chromatography (hexanes/EtOAc, 60:40), yielding aldehyde **6e** (171.4 mg, 0.44 mmol, 71%) as a yellow oil. [α]_D_: −17 (*c* 0.23, CHCl_3_). IR (CHCl_3_) ν_max_ 3019, 1749, 1535, 1350 cm^−1^. ^1^H NMR (500 MHz, CDCl_3_, 26 °C) δ_H_ 9.60 (s, 1H), 8.30 (br b, *J* = 8.5 Hz, 2H), 8.04 (br b, *J* = 8.5 Hz, 2H), 5.57 (d, *J* = 12.4 Hz, 1H), 5.33 (d, *J* = 12.4 Hz, 1H), 5.06 (t, *J* = 6.9 Hz, 1H), 3.56 (s, 3H), 3.20 (dd, *J* = 18.4, 6.6 Hz, 1H), 2.94 (dd, *J* = 18.5, 7.1 Hz, 1H), 1.87 (s, 3H). ^13^C RMN (125.7 MHz, CDCl_3_, 26 °C) δ_C_ 196.9 (CH), 170.0 (C), 169.5 (C), 150.5 (C), 145.4 (C), 129.4 (2 × CH), 124.2 (2 × CH), 70.5 (CH_2_), 54.6 (CH), 53.2 (CH_3_), 44.5 (CH_2_), 20.7 (CH_3_). HRMS (ESI) calculated for C_15_H_20_N_2_O_10_SNa [M + MeOH + Na]^+^ 443.0736, found 443.0730. Anal. Calcd for C_14_H_16_N_2_O_9_S: C, 43.30; H, 4.15; N, 7.21; S, 8.26. Found: C, 43.26; H, 4.22; N, 7.31; S, 8.47.

**Methyl (2*S*)-*N*-(acetoxymethyl)-*N*-benzoyl-4-oxo-L-homoalanine (6f).** Obtained from *N*-benzoyl-L-hydroxyproline **5f** (249.1 mg, 1.0 mmol) according to the general pyrrolidine scission procedure. After work-up and solvent evaporation, the residue was purified by radial chromatography (hexanes/EtOAc, 60:40), yielding aldehyde **6f** (270.8 mg, 0.88 mmol, 88%) as a yellow oil, whose characterization data were already reported [56]. [α]_D_: −76 (c 0.42, CHCl_3_); ^1^H NMR (500 MHz, CDCl_3_, 26 °C): δ_H_ 2.12 (3H, s), 3.25 (1H, dd, *J* = 7.6, 18.8 Hz), 3.51 (1H, br d, *J* = 16.7 Hz), 3.78 (3H, s), 4.95 (1H, dd, *J* = 5.3, 7.7 Hz), 5.43 (2H, br s), 7.35–7.53 (5H, m), 9.80 (1H, s); HRMS (ESI-TOF): calcd for C_15_H_17_NO_6_Na (M^+^ + Na), 330.0954; found, 330.0952.

**Methyl (2*S*)-*N*-(acetoxymethyl)-*N*-(*p*-fluorobenzoyl)-4-oxo-L-homoalanine (6g).** Obtained from *N*-(*p*-fluorobenzoyl)-L-hydroxyproline **5g** (267.1 mg, 1.0 mmol) according to the general pyrrolidine scission procedure. After work-up and solvent evaporation, the residue was purified by radial chromatography (hexanes/EtOAc, 60:40), yielding aldehyde **6g** (277.7 mg, 0.85 mmol, 85%) as a yellow oil. [α]_D_: −56 (*c* 0.39, CHCl_3_). IR (CHCl_3_) ν_max_ 3021, 1745, 1658, 1604 cm^−1^. ^1^H NMR (500 MHz, CDCl_3_, 55 °C) δ_H_ 9.81 (s, 1H), 7.55–7.50 (m, 2H), 7.10 (t, *J_H_*_,*H*_ = 8.6, *J_H_*_,*F*_ = 8.6, Hz, 2H), 5.43 (d, *J* = 11.6 Hz, 1H), 5.38 (d, *J* = 11.6 Hz, 1H), 4.95 (dd, *J* = 7.9, 5.2 Hz, 1H), 3.77 (s, 3H), 3.47 (dd, *J* = 18.6, 5.2 Hz, 1H), 3.20 (dd, *J* = 18.6, 8.0 Hz, 1H), 2.10 (s, 3H). ^13^C RMN (125.7 MHz, CDCl_3_, 55 °C) δ_C_ 198.4 (CH), 171.7 (C), 170.4 (C), 170.0 (C), 164.4 (C, d, *J_CF_* = 252.7 Hz), 130.6 (C, d, *J_CF_* = 3.68 Hz), 130.1 (2 × CH, d, *J_CF_* = 8.72 Hz), 115.8 (2 × CH, d, *J_CF_* = 22.1 Hz), 74.1 (CH_2_), 55.2 (CH), 52.9 (CH_3_), 44.2 (CH_2_), 20.9 (CH_3_). HRMS (ESI) calculated for C_16_H_20_FNO_7_Na [M + MeOH + Na]^+^ 380.1121, found 380.1126. Anal. Calcd for C_15_H_16_FNO_6_: C, 55.39; H, 4.96; N, 4.31. Found: C, 55.44; H, 5.31; N, 4.24.

**Methyl (2*S*)-*N*-(acetoxymethyl)-*N*-(*p*-chlorobenzoyl)-4-oxo-L-homoalanine (6h).** Obtained from *N*-(*p*-chlorobenzoyl)-L-hydroxyproline **5h** (226.5 mg, 0.80 mmol) according to the general pyrrolidine scission procedure. After work-up and solvent evaporation, the residue was purified by radial chromatography (hexanes/EtOAc, 60:40), yielding aldehyde **6h** (222.7 mg, 0.65 mmol, 82%) as a yellow oil. [α]_D_: −63 (*c* 0.36, CHCl_3_). IR (CHCl_3_) ν_max_ 3021, 1746, 1656, 1598 cm^−1^. ^1^H NMR (500 MHz, CDCl_3_, 26 °C) δ_H_ 9.80 (s, 1H), 7.45 (br d, *J* = 8.6, 2H), 7.40 (br d, *J* = 8.6 Hz, 2H), 5.44–5.35 (br b, 2H), 4.91 (dd, *J* = 8.2, 5.0 Hz, 1H), 3.76 (s, 3H), 3.54–3.45 (m, 1H), 3.24 (dd, *J* = 19.1, 8.1 Hz, 1H), 2.11 (s, 3H). ^13^C RMN (125.7 MHz, CDCl_3_, 26 °C) δ_C_ 198.8 (CH), 171.7 (C), 170.6 (C), 170.0 (C), 137.3 (C), 132.7 (C), 129.1 (2 × CH), 128.9 (2 × CH), 74.2 (CH_2_), 55.0 (CH), 53.0 (CH_3_), 44.1 (CH_2_), 20.9 (CH_3_). HRMS (ESI) calculated for C_16_H_20_NO_7_ClNa [M + MeOH + Na]^+^ 396.0826, found 396.0821. Anal. Calcd for C_15_H_16_NO_6_Cl: C, 52.72; H, 4.72; N, 4.10. Found: C, 52.15; H, 4.51; N, 4.00.

**Methyl (2*S*)-*N*-(acetoxymethyl)-*N*-(*p*-iodobenzoyl)-4-oxo-L-homoalanine (6i).** Obtained from *N*-(*p*-iodobenzoyl)-L-hydroxyproline **5i** (300.0 mg, 0.80 mmol) according to the general pyrrolidine scission procedure. After work-up and solvent evaporation, the residue was purified by radial chromatography (hexanes/EtOAc, 70:30), yielding aldehyde **6i** (251.0 mg, 0.58 mmol, 73%) as a colorless oil. [α]_D_: −66 (*c* 0.36, CHCl_3_). IR (CHCl_3_) ν_max_ 3021, 1745, 1658, 1587 cm^−1^. ^1^H NMR (500 MHz, CDCl_3_, 26 °C) δ_H_ 9.81 (s, 1H), 7.78 (br b, *J* = 8.4 Hz, 2H), 7.23 (d, *J* = 8.4 Hz, 2H), 5.38 (br b, 2H), 4.91 (dd, *J* = 8.1, 4.9 Hz, 1H), 3.76 (s, 3H), 3.54–3.46 (m, 1H), 3.25 (dd, *J* = 18.9, 8.1 Hz, 1H), 2.11 (s, 3H). ^13^C RMN (125.7 MHz, CDCl_3_, 26 °C) δ_C_ 198.8 (CH), 171.9 (C), 170.6 (C), 169.9 (C), 137.8 (2 × CH), 133.7 (C), 129.2 (2 × CH), 97.7 (C), 74.2 (CH_2_), 55.0 (CH), 53.0 (CH_3_), 44.1 (CH_2_), 20.9 (CH_3_). HRMS (ESI) calculated for C_16_H_20_INO_7_Na [M + MeOH + Na]^+^ 488.0182, found 488.0181. Anal. Calcd for C_15_H_16_INO_6_: C, 41.59; H, 3.72; N, 3.23. Found: C, 41.77; H, 3.86; N, 3.53.

**Methyl (2*S*)-*N*-(acetoxymethyl)-*N*-(*p*-nitrobenzoyl)-4-oxo-L-homoalanine (6j).** Obtained from *N*-(*p*-nitrobenzoyl)-L-hydroxyproline **5j** (235.3 mg, 0.80 mmol) according to the general pyrrolidine scission procedure. After work-up and solvent evaporation, the residue was purified by radial chromatography (hexanes/EtOAc, 60:40), yielding aldehyde **6j** (245.4 mg, 0.70 mmol, 87%) as a colorless oil. [α]_D_: −63 (*c* 0.33, CHCl_3_). IR (CHCl_3_) ν_max_ 3022, 1748, 1662, 1528, 1347 cm^−1^. ^1^H NMR (500 MHz, CDCl_3_, 26 °C) δ_H_ 9.83 (s, 1H), 8.29 (d, *J* = 8.6 Hz, 2H), 7.66 (d, *J* = 8.7 Hz, 2H), 5.39 (d, *J* = 11.8 Hz, 1H), 5.32 (d, *J* = 11.8 Hz, 1H), 4.94 (dd, *J* = 8.5, 4.6 Hz, 1H), 3.78 (s, 3H), 3.53 (dd, *J* = 19.1, 4.6 Hz, 1H), 3.33 (dd, *J* = 19.2, 8.5 Hz, 1H), 2.11 (s, 3H). ^13^C RMN (125.7 MHz, CDCl_3_, 26 °C) δ_C_ 198.8 (CH), 170.7 (C), 170.6 (C), 169.6 (C), 149.1 (C), 140.4 (C), 128.7 (2 × CH), 123.9 (2 × CH), 73.7 (CH_2_), 55.0 (CH), 53.2 (CH_3_), 44.0 (CH_2_), 20.9 (CH_3_). HRMS (ESI) calculated for C_16_H_20_N_2_O_9_Na [M + MeOH + Na]^+^ 407.1066, found 407.1066. Anal. Calcd for C_15_H_16_N_2_O_8_: C, 51.14; H, 4.58; N, 7.95. Found: C, 51.44; H, 4.73; N, 7.60.

**Methyl (2*S*)-*N*-(acetoxymethyl)-*N*-(3,5-dinitrobenzoyl)-4-oxo-L-homoalanine (6k).** Obtained from *N*-(3,5-dinitrobenzoyl)-L-hydroxyproline **5k** (339.1 mg, 1.00 mmol) according to the general pyrrolidine scission procedure. After work-up and solvent evaporation, the residue was purified by radial chromatography (hexanes/EtOAc, 60:40), yielding aldehyde **6k** (309.4 mg, 0.78 mmol, 78%) as a yellow oil. [α]_D_: −54 (*c* 0.35, CHCl_3_). IR (CHCl_3_) ν_max_ 1749, 1670, 1548, 1344 cm^−1^. ^1^H NMR (500 MHz, CDCl_3_, 26 °C) δ_H_ 9.84 (s, 1H), 9.15 (t, *J* = 2.1 Hz, 1H), 8.72 (br b, 2H), 5.42 (d, *J* = 11.9 Hz, 1H), 5.29 (d, *J* = 11.9 Hz, 1H), 5.04–4.96 (m, 1H), 3.81 (s, 3H), 3.59–3.49 (m, 1H), 3.38 (dd, *J* = 19.2, 9.0 Hz, 1H), 2.16 (s, 3H). ^13^C RMN (125.7 MHz, CDCl_3_, 26 °C) δ_C_ 198.4 (CH), 170.5 (C), 169.3 (C), 168.2 (C), 148.5 (2 × C), 137.8 (C), 128.1 (2 × CH), 120.7 (CH), 73.5 (CH_2_), 55.4 (CH), 53.4 (CH_3_), 43.9 (CH_2_), 20.7 (CH_3_). HRMS (ESI) calculated for C_16_H_19_N_3_O_11_Na [M + MeOH + Na]^+^ 452.0917, found 452.0922. Anal. Calcd for C_15_H_15_N_3_O_10_: C, 45.35; H, 3.81; N, 10.58. Found: C, 45.59; H, 3.56; N, 10.86.

**Methyl (2*S*)-*N*-(acetyl)-*N*-(acetoxymethyl)-4-oxo-L-homoalanine (6l).** Obtained from *N*-(acetyl)-L-hydroxyproline **5l** (112.1 mg, 0.60 mmol) according to the general pyrrolidine scission procedure. After work-up and solvent evaporation, the residue was purified by radial chromatography (hexanes/EtOAc, 50:50), yielding aldehyde **6l** (105.7 mg, 0.43 mmol, 72%) as a yellow oil. [α]_D_: −80 (*c* 0.33, CHCl_3_). IR (CHCl_3_) ν_max_ 3023, 3012, 1744, 1673 cm^−1^. ^1^H NMR (500 MHz, CDCl_3_, 26 °C) δ_H_ 9.74 (s, 1H), 5.55 (d, *J* = 12.0 Hz, 1H), 5.38 (d, *J* = 12.0 Hz, 1H), 4.79 (dd, *J* = 7.9, 4.9 Hz, 1H), 3.69 (s, 3H), 3.41 (dd, *J* = 18.9, 4.9 Hz, 1H), 3.12 (dd, *J* = 18.9, 8.2 Hz, 1H), 2.22 (s, 3H), 2.09 (s, 3H). ^13^C RMN (125.7 MHz, CDCl_3_, 26 °C) δ_C_ 199.2 (CH), 172.0 (C), 170.7 (C), 170.2 (C), 73.5 (CH_2_), 55.2 (CH_2_), 52.9 (CH_3_), 44.5 (CH_2_), 21.6 (CH_3_), 20.9 (CH_3_). HRMS (ESI) calculated for C_11_H_19_NO_7_Na [M + MeOH + Na]^+^ 300.1059, found 300.1054. Anal. Calcd for C_10_H_15_NO_6_: C, 48.98; H, 6.17; N, 5.71. Found: C, 48.71; H, 6.18; N, 5.62.

**Methyl (2*S*)-*N*-(acetoxymethyl)-*N*-[*N*-(benzyloxycarbonyl)phenylalanyl]-4-oxo -L-homoalanine (6m).** Obtained from *N*-[*N*-(benzyloxycarbonyl)phenylalanyl]- L-hydroxyproline **5m** (298.3 mg, 0.70 mmol) according to the general pyrrolidine scission procedure. After work-up and solvent evaporation, the residue was purified by radial chromatography (hexanes/EtOAc, 50:50), yielding aldehyde **6m** (238.6 mg, 0.49 mmol, 70%) as a yellow oil. [α]_D_: −26 (*c* 0.36, CHCl_3_). IR (CHCl_3_) ν_max_ 3301, 1716, 1639, 1524, 1435 cm^−1^. ^1^H NMR (500 MHz, CDCl_3_, 26 °C) δ_H_ 9.64 (br b, 1H), 7.38–7.04 (m, 10H), 5.47 (d, *J* = 8.4 Hz, 1H), 5.37 (d, *J* = 12.2 Hz, 1H), 5.19 (d, *J* = 12.2 Hz, 1H), 5.12 (d, *J* = 12.3 Hz, 1H), 5.08 (d, *J* = 12.4 Hz, 1H), 5.08–5.03 (m, 1H), 4.70 (dd, *J* = 7.6, 5.1 Hz, 1H), 3.63 (s, 3H), 3.34 (dd, *J* = 18.9, 5.2 Hz, 1H), 3.04–2.96 (m, 2H), 2.81 (dd, *J* = 18.8, 7.6 Hz, 1H), 2.01 (s, 3H). ^13^C RMN (125.7 MHz, CDCl_3_, 26 °C) δ_C_ 198.5 (CH), 173.2 (C), 170.6 (C), 169.7 (C), 155.5 (C), 136.4 (C), 135.7 (C), 129.6 (2 × CH), 128.7 (2 × CH), 128.7 (2 × CH), 128.3 (CH), 128.2 (2 × CH), 127.4 (CH), 72.1 (CH_2_), 67.1 (CH_2_), 55.7 (CH), 52.8 (CH_3_), 52.4 (CH), 44.0 (CH_2_), 40.0 (CH_2_), 20.7 (CH_3_). HRMS (ESI) calculated for C_26_H_32_N_2_O_9_Na [M + MeOH + Na]^+^ 507.1743, found 507.1740. Anal. Calcd for C_25_H_28_N_2_O_8_: C, 61.98; H, 5.83; N, 5.78. Found: C, 61.81; H, 6.02; N, 5.41.

**Methyl (2*S*)-*N*-(acetoxymethyl)-*N*-(*terc*-butoxycarbonyl)-4-oxo-L-homoalanine (6n).** Obtained from *N*-(*tert*-butoxycarbonyl)-L-hydroxyproline **5n** (147.2 mg, 0.60 mmol) according to the general pyrrolidine scission procedure. After work-up and solvent evaporation, the residue was purified by radial chromatography (hexanes/EtOAc, 80:20), yielding aldehyde **6n** (126.0 mg, 0.42 mmol, 70%) as a yellow oil whose characterization data were already reported [56]. [α]_D_: −80 (c 0.33, CHCl_3_); ^1^H NMR (500 MHz, CDCl_3_, 70 °C) rotamer mixture at 26 °C, one visible rotamer at 70 °C: δ_H_ 1.47 (9H, s), 2.05 (3H, s/s), 2.95 (1H, m), 3.31 (1H, dd, *J* = 6.1, 18 Hz), 3.73 (3H, s), 4.83 (1H, m), 5.41 (2H, br s), 9.76 (1H, s); HRMS (ESI-TOF): calcd for C_14_H_25_NO_8_Na (M^+^ + Na + MeOH), 358.1478; found, 358.1467.

**Methyl (2*S*)-*N*-(acetoxymethyl)-*N*-(benzyloxycarbonyl)-4-oxo-L-homoalanine (6o).** Obtained from *N*-(benzyloxycarbonyl)-L-hydroxyproline **5o** (223.3 mg, 0.80 mmol) according to the general pyrrolidine scission procedure. After work-up and solvent evaporation, the residue was purified by radial chromatography (hexanes/EtOAc, 70:30), yielding aldehyde **6o** (152.2 mg, 0.45 mmol, 56%) as a colorless oil. [α]_D_: −70 (*c* 0.36, CHCl_3_). IR (CHCl_3_) ν_max_ 1726, 1437, 1421, 1367 cm^−1^. ^1^H NMR (500 MHz, CD_3_CN, 70 °C) δ_H_ 9.68 (s, 1H), 7.42–7.32 (m, 5H), 5.43 (d, *J* = 11.3 Hz, 1H), 5.41 (d, *J* = 11.2 Hz, 1H), 5.16 (br b, 2H), 4.92 (t, *J* = 6.7 Hz, 1H), 3.61 (s, 3H), 3.24 (dd, *J* = 18.1, 6.4 Hz, 1H), 2.95 (dd, *J* = 18.2, 7.0 Hz, 1H), 1.99 (s, 3H). ^13^C RMN (125.7 MHz, CD_3_CN, 70 °C) δ_C_ 200.4 (CH), 171.8 (C), 171.7 (C), 156.4 (C), 137.6 (C), 129.8 (2 × CH), 129.5 (CH), 129.1 (2 × CH), 73.4 (CH_2_), 69.1 (CH_2_), 56.6 (CH), 53.4 (CH_3_), 45.5 (CH_2_), 21.2 (CH_3_). HRMS (ESI) calculated for C_17_H_23_NO_8_Na [M + MeOH + Na]^+^ 392.1321, found 392.1319. Anal. Calcd for C_16_H_19_NO_7_: C, 56.97; H, 5.68; N, 4.15. Found: C, 56.87; H, 5.87; N, 4.08.

**Methyl (2*S*)-*N*-(acetoxymethyl)-*N*-(phenyloxycarbonyl)-4-oxo-L-homoalanine (6p).** Obtained from *N*-(phenyloxycarbonyl)-L-hydroxyproline **5p** (265.1 mg, 1.00 mmol) according to the general pyrrolidine scission procedure. After work-up and solvent evaporation, the residue was purified by radial chromatography (hexanes/EtOAc, 70:30), yielding aldehyde **6p** (256.4 mg, 0.79 mmol, 79%) as a yellow oil. [α]_D_: −89 (*c* 0.36, CHCl_3_). IR (CHCl_3_) ν_max_ 1731, 1599, 1417, 1288, 1198 cm^−1^. ^1^H NMR (500 MHz, CD_3_CN, 70 °C) δ_H_ 9.76 (s, 1H), 7.42 (br t, *J* = 7.8 Hz, 2H), 7.28 (t, *J* = 7.7 Hz, 1H), 7.14 (d, *J* = 7.9 Hz, 2H), 5.64–5.47 (m, 2H), 5.09–4.96 (m, 1H), 3.74 (s, 3H), 3.34 (dd, *J* = 18.2, 5.7 Hz, 1H), 3.09 (dd, *J* = 17.9, 5.6 Hz, 1H), 2.02 (s, 3H). ^13^C RMN (125.7 MHz, CD_3_CN, 70 °C) δ_C_ 200.3 (CH), 171.8 (C), 171.6 (C), 155.1 (C), 152.4 (C), 130.7 (2 × CH), 127.1 (CH), 122.7 (2 × CH), 73.5 (CH_2_), 56.9 (CH), 53.6 (CH_3_), 45.4 (CH_2_), 21.2 (CH_3_). HRMS (ESI) calculated for C_16_H_21_NO_8_Na [M + MeOH + Na]^+^ 378.1165, found 378.1168. Anal. Calcd for C_15_H_17_NO_7_: C, 55.73; H, 5.30; N, 4.33. Found: C, 55.61; H, 5.43; N, 4.43.

**General procedure for the preparation of homoserine lactones.**

**Method A**: A solution of the 4-oxo-L-homoalanine derivative (0.20 mmol) in dry methanol (3.0 mL), at room temperature, was treated with NaBH_4_ (9.8 mg, 0.26 mmol, 1.3 equiv.). The reaction mixture was stirred at 45 °C for 4 h. Then, the solvent was removed under vacuum, and the residue was poured into water and extracted with EtOAc. The organic phase was dried over anhydrous sodium sulfate, filtered, and concentrated under vacuum. The crude oil obtained was dissolved in dichloromethane (3.0 mL), and Et_3_N (100 μL) was added. The mixture was stirred at 40 °C for 1 h, and then the solvent was removed under vacuum. The residue was purified by radial chromatography on silica gel (n-hexane/EtOAc) to obtain the corresponding α-amino lactones.

**Method B**: To a solution of the 4-oxo-L-homoalanine derivative (0.20 mmol) in dry dichloromethane (4.0 mL), boron trifluoride diethyleterate (50 μL, 57.5 mg, 0.40 mmol, 2.0 equiv.) and triethylsilane (80 μL, 58.0 mg, 0.57 mmol, 2.5 equiv.) were added. The reaction mixture was stirred at 26 °C for 16 h under a nitrogen atmosphere. Then, Et_3_N (100 μL) was added, and stirring was continued for 2 h. The mixture was concentrated under vacuum and the residue was purified by radial chromatography on silica gel (n-hexane/EtOAc mixtures), yielding the corresponding *N*-alkyl-α-amino lactones.

**(2*S*)-*N*-(Benzenesulfonyl)homoserine lactone (7a). **Obtained from aldehyde **6a** (68.6 mg, 0.20 mmol) according to method A of the general procedure for the preparation of lactones. After work-up and solvent evaporation, the residue was purified by radial chromatography (hexanes/EtOAc, 70:30), yielding lactone **7a** (30.4 mg, 0.13 mmol, 63%) as a colorless oil, which was known [85] but not completely characterized: [α]_D_: −2 (*c* 0.25, CHCl_3_). IR (CHCl_3_) ν_max_ 3330, 1785, 1602, 1349, 1169 cm^−1^. ^1^H NMR (500 MHz, CDCl_3_, 26 °C) δ_H_ 7.91 (d, *J* = 7.8 Hz, 2H), 7.62 (t, *J* = 7.5 Hz, 1H), 7.54 (t, *J* = 7.8 Hz, 2H), 4.41 (t, *J* = 9.1 Hz, 1H), 4.19 (ddd, *J* = 11.7, 9.4, 5.7 Hz, 1H), 3.97 (dd, *J* = 11.6, 8.4 Hz, 1H), 2.69 (ddd, *J* = 12.7, 8.5, 5.6 Hz, 1H), 2.31–2.21 (m, 1H). ^13^C RMN (125.7 MHz, CDCl_3_, 26 °C) δ_C_ 174.2 (C), 139.1 (C), 133.4 (CH), 129.5 (2 × CH), 127.4 (2 × CH), 66.2 (CH_2_), 51.9 (CH), 31.3 (CH_2_). HRMS (ESI) calculated for C_10_H_11_NO_4_SNa [M + Na]^+^ 264.0306, found 264.0304. Anal. Calcd for C_10_H_11_NO_4_S: C, 49.78; H, 4.60; N, 5.81; S, 13.29. Found: C, 49.62; H, 4.70; N, 5.80; S, 13.54.

**(2*S*)-*N*-(*p*-Toluenesulfonyl)homoserine lactone (7b). **Obtained from aldehyde **6b** (38.2 mg, 0.11 mmol) according to method A for the preparation of homoserine lactones. After work-up and solvent evaporation, the residue was purified by radial chromatography (hexanes/EtOAc, 70:30), yielding lactone **7b** (17.7 mg, 0.07 mmol, 65%) as a crystalline solid whose characterization data were already reported [49], but since the deuterated solvent is different (CDCl3/d-DMSO) our data are given herein. [α]_D_: −2 (*c* 0.48, CHCl_3_). ^1^H NMR (500 MHz, CDCl_3_, 26 °C) δ_H_ 7.81–7.77 (m, 2H), 7.33 (d, *J* = 7.8 Hz, 2H), 5.30 (d, *J* = 2.1 Hz, 1H), 4.41 (t, *J* = 9.2 Hz, 1H), 4.18 (ddd, *J* = 11.7, 9.4, 5.6 Hz, 1H), 3.92 (ddd, *J* = 11.9, 8.3, 3.8 Hz, 1H), 2.69 (m, 1H), 2.43 (s, 3H), 2.27 (m, 1H). HRMS (ESI) calculated for C_11_H_13_NO_4_SNa [M + Na]^+^ 278.0463, found 278.0461.

**(2*S*)-*N*-(*p*-Chlorophenylsulfonyl)homoserine lactone (7c).** Obtained from aldehyde **6c** (75.4 mg, 0.20 mmol) according to method A for the preparation of homoserine lactones. After work-up and solvent evaporation, the residue was purified by radial chromatography (hexanes/EtOAc, 60:40), yielding lactone **7c** (16.0 mg, 0.06 mmol, 29%) as a colorless oil. [α]_D_: 3 (*c* 0.33, (CH_3_)_2_CO). IR (ATR) ν_max_ 3022, 1746, 1356, 1232, 1167 cm^−1^. ^1^H NMR (500 MHz, CDCl_3_, 26 °C) δ_H_ 7.85 (br d, *J* = 8.5 Hz, 2H), 7.51 (br d, *J* = 8.6 Hz, 2H), 5.60–5.40 (br b, 1H), 4.43 (t, *J* = 9.1 Hz, 1H), 4.20 (ddd, *J* = 11.6, 9.5, 5.6 Hz, 1H), 4.00 (dd, *J* = 11.7, 8.4 Hz, 1H), 2.70 (dddd, *J* = 12.8, 8.3, 5.6, 1.2 Hz, 1H), 2.31–2.21 (m, 1H). ^13^C RMN (125.7 MHz, CDCl_3_, 26 °C) δ_C_ 174.1 (C), 140.0 (C), 137.8 (C), 129.8 (2 × CH), 128.9 (2 × CH), 66.2 (CH_2_), 52.0 (CH), 31.3 (CH_2_, 4-C). HRMS (ESI) calculated for C_10_H_10_ClNO_4_SNa [M + Na]^+^ 297.9917, found 297.9919.

**(2*S*)-*N*-(*p*-Iodophenylsulfonyl)homoserine lactone (7d).** Obtained from aldehyde **6d** (93.8 mg, 0.20 mmol) according to method A for the preparation of homoserine lactones. After work-up and solvent evaporation, the residue was purified by radial chromatography (hexanes/EtOAc, 70:30), yielding lactone **7d** (38.2 mg, 0.10 mmol, 52%) as a crystalline solid: mp 141–143 °C (from n-hexane/EtOAc); [α]_D_: +4 (*c* 0.40, (CH_3_)_2_CO). IR (ATR) ν_max_ 3238, 2921, 1780, 1337, 1162 cm^−1^. ^1^H NMR (500 MHz, (CD_3_)_2_CO, 26 °C) δ_H_ 8.00 (br d, *J* = 8.6 Hz), 7.71 (d, *J* = 8.7 Hz, 2H), 4.41 (dd, *J* = 11.6, 8.6 Hz, 1H), 4.34 (td, *J* = 8.9, 1.4 Hz, 1H), 4.24 (ddd, *J* = 11.1, 9.1, 5.9 Hz, 1H), 2.53 (dddd, *J* = 12.5, 8.6, 5.9, 1.4 Hz, 1H), 2.20–2.10 (m, 1H). ^13^C RMN (125.7 MHz, (CD_3_)_2_CO, 26 °C) δ_C_ 174.6 (C), 142.3 (C), 139.2 (2 × CH), 129.5 (2 × CH), 100.1 (C), 66.1 (CH_2_), 52.7 (CH), 31.2 (CH_2_). HRMS (ESI) calculated for C_10_H_10_INO_4_SNa [M + Na]^+^ 389.9273, found 389.9269.

**(2*S*)-*N*-(*p*-Nitrophenylsulfonyl)homoserine lactone (7e).**Obtained from aldehyde **6e** (77.6 mg, 0.20 mmol) according to method A for the preparation of homoserine lactones. After work-up and solvent evaporation, the residue was purified by radial chromatography (hexanes/EtOAc, 70:30), yielding lactone **7e** (32.0 mg, 0.11 mmol, 56%) as a crystalline solid: mp 162–164 °C (from n-hexane/EtOAc); [α]_D_: +1 (*c* 0.33, (CH_3_)_2_CO). IR (ATR) ν_max_ 3297, 1781, 1534, 1347, 1169 cm^−1^. ^1^H NMR (500 MHz, (CD_3_)_2_CO, 26 °C) δ_H_ 8.43 (br d, *J* = 9.0 Hz, 2H), 8.20 (br d, *J* = 9.1 Hz, 2H), 4.54 (dd, *J* = 11.6, 8.5 Hz, 1H), 4.36 (ddd, *J* = 8.9, 8.9, 1.4 Hz, 1H), 4.26 (ddd, *J* = 11.1, 9.1, 5.9 Hz, 1H), 2.59 (dddd, *J* = 12.5, 8.6, 6.0, 1.4 Hz, 1H), 2.25–2.17 (m, 1H). ^13^C RMN (125.7 MHz, (CD_3_)_2_CO, 26 °C) δ_C_ 174.6 (C), 151.1 (C), 148.2 (C), 129.4 (2 × CH), 125.2 (2 × CH), 66.1 (CH_2_), 52.8 (CH), 31.1 (CH_2_). HRMS (ESI) calculated for C_10_H_10_N_2_O_6_SNa [M + Na]^+^ 309.0157, found 309.0158. Anal. Calcd for C_10_H_10_N_2_O_6_S: C, 41.96; H, 3.52; N, 9.79; S, 11.20. Found: C, 41.69; H, 3.53; N, 9.51; S, 10.94.

**(2*S*)-*N*-(Benzoyl)homoserine lactone (7f). **Obtained from aldehyde **6f** (30.7 mg, 0.10 mmol) according to method A for the preparation of homoserine lactones. After work-up and solvent evaporation, the residue was purified by radial chromatography (hexanes/EtOAc, 50:50), yielding lactone **7f** (13.7 mg, 0.01 mmol, 67%) as a crystalline solid: mp 126–128 °C (from n-hexane/EtOAc); [α]_D_: +15 (*c* 0.34, CHCl_3_). IR (CHCl_3_) ν_max_ 3430, 3017, 1779, 1667, 1514, 1486 cm^−1^. ^1^H NMR (500 MHz, CD_3_CN, 70 °C) δ_H_ 7.83–7.79 (m, 2H), 7.56 (br t, *J* = 7.5 Hz, 1H), 7.48 (br t, *J* = 7.5 Hz, 2H), 4.71 (ddd, *J* = 11.0, 9.2, 7.9 Hz, 1H), 4.45 (ddd, *J* = 9.0, 9.0, 2.0 Hz, 1H), 4.28 (ddd, *J* = 10.4, 9.0, 6.6 Hz, 1H), 2.60–2.52 (m, 1H), 2.46–2.36 (m, 1H). ^13^C RMN (125.7 MHz, CD_3_CN, 70 °C) δ_C_ 176.3 (C), 168.3 (C), 135.3 (C), 133.0 (CH), 129.9 (2 × CH), 128.4 (2 × CH), 67.0 (CH_2_), 50.3 (CH), 29.7 (CH_2_). HRMS (ESI) calculated for C_11_H_11_NO_3_Na [M + Na]^+^ 228.0637, found 228.0635. Anal. Calcd for C_11_H_11_NO_3_: C, 64.38; H, 5.40; N, 6.83. Found: C, 64.36; H, 5.78; N, 6.58.

**(2*S*)-*N*-(*p*-Fluorobenzoyl)homoserine lactone (7g). **Obtained from aldehyde **6g** (65.0 mg, 0.20 mmol) according to method A for the preparation of homoserine lactones. After work-up and solvent evaporation, the residue was purified by radial chromatography (hexanes/EtOAc, 50:50), yielding lactone **7g** (30.0 mg, 0.13 mmol, 67%) as a crystalline solid: mp 152–154 °C (from n-hexane/EtOAc); [α]_D_: +1 (*c* 0.81, (CH_3_)_2_CO). IR (CHCl_3_) ν_max_ 3426, 1780, 1668, 1604, 1493 cm^−1^. ^1^H NMR (500 MHz, CDCl_3_, 26 °C) δ_H_ 7.85–7.77 (m, 2H), 7.08 (br t, *J_H_*_,*H*_ = 8.5, *J_H_*_,*F*_ = 8.5 Hz, 2H), 7.01–6.92 (m, 1H), 4.81–4.72 (m, 1H), 4.52 (t, *J* = 9.1 Hz, 1H), 4.35 (ddd, *J* = 11.1, 9.2, 6.0 Hz, 1H), 2.94–2.85 (m, 1H), 2.35–2.24 (m, 1H). ^13^C RMN (125.7 MHz, CDCl_3_, 26 °C) δ_C_ 176.1 (C), 166.8 (C), 165.5 (C, d, *J*_CF_ = 252.7 Hz), 129.6 (2 × CH, d, *J*_CF_ = 9.2 Hz), 129.2 (C, br s), 116.0 (2 × CH, d, *J*_CF_ = 22.5 Hz), 66.5 (CH_2_), 49.8 (CH), 30.4 (CH_2_). HRMS (ESI) calculated for C_11_H_10_FNO_3_Na [M + Na]^+^ 246.0542, found 246.0541. Anal. Calcd for C_11_H_10_FNO_3_: C, 59.19; H, 4.52; N, 6.28. Found: C, 59.23; H, 4.87; N, 6.00.

**(2*S*)-*N*-(*p*-Chlorobenzoyl)homoserine lactone (7h). **Obtained from aldehyde **6h** (68.2 mg, 0.20 mmol) according to method A for the preparation of homoserine lactones. After work-up and solvent evaporation, the residue was purified by radial chromatography (CH_2_Cl_2_/MeOH, 98:2), yielding lactone **7h** (18.8 mg; 0.08 mmol; 40%) as an amorphous solid. [α]_D_: −1 (*c* 0.30, (CH_3_)_2_CO). IR (ATR) ν_max_ 3409, 1764, 1662, 1528, 1485 cm^−1^. ^1^H NMR (500 MHz, (CD_3_)_2_CO, 26 °C) δ_H_ 8.28 (br d, *J* = 4.6 Hz, 1H), 7.93 (br d, *J* = 8.9 Hz, 2H), 7.52 (br d, *J* = 8.9 Hz, 2H), 4.88 (ddd, *J* = 11.0, 9.1, 8.0 Hz, 1H), 4.47 (ddd, *J* = 9.0, 9.0, 1.9 Hz, 1H), 4.36 (ddd, *J* = 10.4, 8.8, 6.4 Hz, 1H), 2.70–2.61 (m, 1H), 2.50–2.41 (m, 1H). ^13^C RMN (125.7 MHz, (CD_3_)_2_CO, 26 °C) δ_C_ 175.4 (C), 166.3 (C), 138.0 (C), 133.7 (C), 129.9 (2 × CH), 129.5 (2 × CH), 66.2 (CH_2_), 49.7 (CH), 29.5 (CH_2_). HRMS (ESI) calculated for C_11_H_10_ClNO_3_Na [M + Na]^+^ 262.0247, found 262.0239. Anal. Calcd for C_11_H_10_ClNO_3_: C, 55.13; H, 4.21; N, 5.84. Found: C, 54.97; H, 4.22; N, 5.67.

**(2*S*)-*N*-(*p*-Iodobenzoyl)homoserine lactone (7i).** Obtained from aldehyde **6i** (86.6 mg, 0.20 mmol) according to method A for the preparation of homoserine lactones. After work-up and solvent evaporation, the residue was purified by radial chromatography (CH_2_Cl_2_/MeOH, 99:1), yielding lactone **7i** (38.7 mg, 0.12 mmol, 59%) as a crystalline solid: mp 206–208 °C (from CH_2_Cl_2_/MeOH); [α]_D_: −1 (*c* 0.33, (CH_3_)_2_CO). IR (CHCl_3_) ν_max_ 3258, 1773, 1644, 15,485, 1540 cm^−1^. ^1^H NMR (500 MHz, (CD_3_)_2_CO, 26 °C) δ_H_ 8.27 (br d, *J* = 6.7 Hz, 1H), 7.89 (br d, *J* = 9.1 Hz, 2H), 7.70 (br d, *J* = 9.1 Hz, 2H), 4.88 (ddd, *J* = 11.0, 9.1, 8.0 Hz, 1H), 4.46 (ddd, *J* = 8.9, 8.9, 1.8 Hz, 1H), 4.36 (ddd, *J* = 10.5, 8.9, 6.5 Hz, 1H), 2.69–2.61 (m, 1H), 2.45 (m, 1H). ^13^C RMN (125.7 MHz, (CD_3_)_2_CO, 26 °C) δ_C_ 175.4 (C), 166.7 (C), 138.6 (2 × CH), 134.5 (C), 130.0 (2 × CH), 98.9 (C), 66.2 (CH_2_), 49.7 (CH), 29.5 (CH_2_). HRMS (ESI) calculated for C_11_H_10_INO_3_Na [M + Na]^+^ 353.9603, found 353.9605.

**(2*S*)-*N*-(*p*-Nitrobenzoyl)homoserine lactone (7j). **Obtained from aldehyde **6j** (70.4 mg, 0.20 mmol) according to method A for the preparation of homoserine lactones. After work-up and solvent evaporation, the residue was purified by radial chromatography (n-hexane/EtOAc, 40:60), yielding lactone **7j** (26.5 mg, 0.11 mmol, 53%) as a crystalline solid: mp 200–202 °C (from n-hexane/EtOAc); [α]_D_: −1 (*c* 0.34, (CH_3_)_2_CO). IR (ATR) ν_max_ 3309, 1742, 1650, 1598, 1519 cm^−1^. ^1^H NMR (500 MHz, (CD_3_)_2_CO, 26 °C) δ_H_ 8.53 (br b, *J* = 6.6 Hz, 1H), 8.35 (br d, *J* = 8.8 Hz, 2H), 8.16 (br d, *J* = 8.9 Hz, 2H), 4.93 (ddd, *J* = 11.1, 9.2, 7.9 Hz, 1H), 4.49 (ddd, *J* = 9.0, 8.9, 1.9 Hz, 1H), 4.38 (ddd, *J* = 10.6, 9.0, 6.5 Hz, 1H), 2.73–2.65 (m, 1H), 2.54–2.44 (m, 1H). ^13^C RMN (125.7 MHz, (CD_3_)_2_CO, 26 °C) δ_C_ 175.2 (C), 165.7 (C), 150.7 (C), 140.4 (C), 129.6 (2 × CH), 124.5 (2 × CH), 66.2 (CH_2_), 49.9 (CH), 29.4 (CH_2_). HRMS (ESI) calculated for C_11_H_10_N_2_O_5_Na [M + Na]^+^ 273.0487, found 273.0487. Anal. Calcd for C_11_H_10_N_2_O_5_: C, 52.80; H, 4.03; N, 11.20. Found: C, 53.07; H, 4.07; N, 10.83.

**(2*S*)-*N*-(3,5-Dinitrobenzoyl)homoserine lactone (7k).** Obtained from aldehyde **6k** (79.4 mg, 0.20 mmol) according to method A for the preparation of homoserine lactones. After work-up and solvent evaporation, the residue was purified by radial chromatography (n-hexane/EtOAc, 50:50), yielding lactone **7k** (20.2 mg, 0.07 mmol, 34%) as a crystalline solid: mp 216–218 °C (from n-hexane/EtOAc); [α]_D_: −16 (*c* 0.23, (CH_3_)_2_CO). IR (ATR) ν_max_ 3335, 1763, 1666, 1541, 1344 cm^−1^. ^1^H NMR (500 MHz, (CD_3_)_2_CO, 26 °C) δ_H_ 9.11 (s, 3H), 8.93 (br b, 1H), 5.05–4.97 (m, 1H), 4.51 (ddd, *J* = 9.1, 9.0, 1.9 Hz, 1H), 4.41 (ddd, *J* = 10.6, 9.0, 6.4 Hz, 1H), 2.77–2.70 (m, 1H), 2.57–2.47 (m, 1H). ^13^C RMN (125.7 MHz, (CD_3_)_2_CO, 26 °C) δ_C_ 175.0 (C), 163.5 (C), 149.7 (2 × C), 137.8 (C), 128.3 (2 × CH), 122.0 (CH), 66.3 (CH_2_), 50.2 (CH), 29.4 (CH_2_). HRMS (ESI) calculated for C_11_H_9_N_3_O_7_Na [M + Na]^+^ 318.0338, found 318.0339.

**(2*S*)-*N*-(Acetyl)homoserine lactone (7l). **Obtained from aldehyde **6l** (24.5 mg, 0.10 mmol) according to method A for the preparation of homoserine lactones. After work-up and solvent evaporation, the residue was purified by radial chromatography (n-hexane/EtOAc, 10:90), yielding lactone **7l** (6.2 mg, 0.04 mmol, 43%) as a crystalline solid whose characterization data have been reported but using different conditions/d-solvent [86]. [α]_D_: −12 (c 0.23, CH_3_COCH_3_). ^1^H NMR (500 MHz, CDCl_3_, 26 °C) δ_H_ 6.45–6.30 (s.a, 1H), 4.58 (ddd, *J* = 11.7, 8.6, 6.1 Hz, 1H), 4.46 (ddd, *J* = 9.1, 9.1, 1.0 Hz, 1H), 4.27 (ddd, *J* = 11.3, 9.3, 6.0 Hz, 1H), 2.76–2.84 (m, 1H), 2.15 (m, 1H), 2.05 (s, 3H). HRMS (ESI) calculated for C_6_H_9_NO_3_Na [M + Na]^+^ 166.0480, found 166.0476.

**(2*S*)-*N*-[*N*-(Benzyloxycarbonyl)phenylalanyl]homoserine lactone (7m).** Obtained from aldehyde **6m** (96.8 mg, 0.20 mmol) according to method A for the preparation of homoserine lactones. After work-up and solvent evaporation, the residue was purified by radial chromatography (n-hexane/EtOAc, 40:60), yielding lactone **7m** (43.7 mg, 0.12 mmol, 57%) as a crystalline solid: mp 124–126 °C (from n-hexane/EtOAc); [α]_D_: −3 (*c* 0.88, CHCl_3_). IR (CHCl_3_) ν_max_ 3447, 3011, 1785, 1672, 1509 cm^−1^. ^1^H NMR (500 MHz, CD_3_CN, 70 °C) δ_H_ 7.38–7.22 (m, 10H), 7.04–6.95 (m, 1H), 5.85–5.60 (br b, 1H), 5.06 (d, *J* = 12.7 Hz, 1H), 5.01 (d, *J* = 12.7 Hz, 1H), 4.54–4.45 (m, 1H), 4.43–4.33 (m, 2H), 4.27–4.19 (m, 1H), 3.21–3.12 (m, 1H), 2.92 (dd, *J* = 14.1, 8.6 Hz, 1H), 2.54–2.43 (m, 1H), 2.25–2.14 (m, 1H). ^13^C RMN (125.7 MHz, CD_3_CN, 70 °C) δ_C_ 176.0 (C), 172.7 (C), 157.2 (C), 138.7 (C), 138.5 (C), 130.7 (2 × CH), 129.74 (2 × CH), 129.68 (2 × CH), 129.2 (CH), 128.9 (2 × CH), 128.0 (CH), 67.6 (CH_2_), 66.9 (CH_2_), 57.6 (CH), 49.9 (CH), 39.2 (CH_2_), 29.7 (CH_2_). HRMS (ESI) calculated for C_21_H_22_N_2_O_5_Na [M + Na]^+^ 405.1426, found 405.1426. Anal. Calcd for C_21_H_22_N_2_O_5_: C, 65.96; H, 5.80; N, 7.33. Found: C, 66.22; H, 6.05; N, 7.45.

**(2*S*)-*N*-[*tert*-(Butoxycarbonyl)homoserine lactone (7n). **Obtained from aldehyde **6n** (53.0 mg, 0.17 mmol) according to method A for the preparation of homoserine lactones. After work-up and solvent evaporation, the residue was purified by radial chromatography (n-hexane/EtOAc, 70:30), yielding lactone **7n** (24.0 mg, 0.12 mmol, 70%) as a crystalline solid: mp 102–104 °C (from n-hexane/EtOAc); [α]_D_: −1 (*c* 0.30, CHCl_3_). IR (CHCl_3_) ν_max_ 3432, 1783, 1713, 1503, 1161 cm^−1^. ^1^H NMR (500 MHz, CDCl_3_, 26 °C) δ_H_ 5.09 (s, 1H), 4.44 (t, *J* = 9.7 Hz, 1H), 4.39–4.30 (m, 1H), 4.24 (ddd, *J* = 11.4, 9.3, 5.9 Hz, 1H), 2.80–2.70 (m, 1H), 2.25–2.13 (m, 1H), 1.45 (s, 9H). ^13^C RMN (125.7 MHz, CDCl_3_, 26 °C) δ_C_ 175.5 (C), 155.6 (C), 80.7 (C), 65.9 (CH_2_), 50.3 (CH), 30.8 (CH_2_), 28.4 (3 × CH_3_). HRMS (ESI) calculated for C_9_H_15_NO_4_Na [M + Na]^+^ 224.0899, found 224.0897. Anal. Calcd for C_9_H_15_NO_4_: C, 53.72; H, 7.51; N, 6.96. Found: C, 53.97; H, 7.45; N, 6.71.

**(2*S*)-*N*-[Benzyloxycarbonyl)homoserine lactone (7o).** Obtained from aldehyde **6o** (67.4 mg, 0.20 mmol) according to method A for the preparation of homoserine lactones. After work-up and solvent evaporation, the residue was purified by radial chromatography (n-hexane/EtOAc, 60:40), yielding lactone **7o** (14.3 mg, 0.06 mmol, 30%) as a crystalline solid whose characterization data have been reported (commercial compound) [86]. ^1^H NMR (500 MHz, CDCl_3_, 26 °C) δ_H_ 7.39–7.30 (m, 5H), 5.47–5.34 (s.a, 1H), 5.16–5.10 (s.a, 2H), 4.48–4.37 (m, 2H), 4.29–4.20 (m, 1H), 2.82–2.72 (m, 1H), 2.27–2.15 (m, 1H). HRMS (ESI) calculated for C_12_H_13_NO_4_Na [M + Na]^+^ 258.0742, found 258.0743.

**Methyl 2-oxo-1,3-oxazinane-4-carboxylate (7p)**. Obtained from aldehyde **6p** (64.6 mg, 0.20 mmol) according to method A for the preparation of lactones. After work-up and solvent evaporation, the residue was purified by radial chromatography (n-hexane/EtOAc, 60:40), yielding lactone **7p** (7.3 mg, 0.05 mmol, 23%) as an amorphous solid. ^1^H NMR (500 MHz, CDCl_3_, 26 °C) δ_H_ 5.34–5.21 (s, 1H), 4.45 (t, *J* = 8.4 Hz, 1H), 4.43–4.34 (m, 1H), 4.26 (ddd, *J* = 11.3, 9.3, 5.8 Hz, 1H), 3.71 (s, 3H), 2.82 – 2.74 (m, 1H), 2.27–2.16 (m, 1H). ^13^C RMN (125.7 MHz, CDCl_3_, 26 °C) δ_C_ 175.1 (C), 156.9 (C), 65.9 (CH_2_), 52.8 (CH), 50.6 (CH_3_), 30.6 (CH_2_). HRMS (ESI) calculated for C_6_H_9_NO_4_Na [M + Na]^+^ 182.0429, found 182.0430.

**(2*S*)-*N*-Methyl-*N*-(*p*-toluenesulfonyl)homoserine lactone (7q).** Obtained from aldehyde **6q** (36.6 mg, 0.10 mmol) according to method B for the preparation of homoserine lactones. After work-up and solvent evaporation, the residue was purified by radial chromatography (n-hexane/EtOAc, 70:30), yielding lactone **7q** (17.0 mg, 0.06 mmol, 62%) as a crystalline solid: mp 131–133 °C (from n-hexane/EtOAc); [α]_D_: −33 (*c* 0.80, CHCl_3_). IR (ATR) ν_max_ 1785, 1363, 1340, 1193, 1164 cm^−1^. ^1^H NMR (500 MHz, CDCl_3_, 26 °C) δ_H_ 7.82–7.68 (m, 2H), 7.35–7.27 (m, 2H), 5.04–4.96 (m, 1H), 4.44–4.36 (m, 1H), 4.29–4.20 (m, 1H), 2.77–2.74 (m, 3H), 2.46–2.40 (m, 4H), 2.37–2.26 (m, 1H). ^13^C RMN (125.7 MHz, CDCl_3_, 26 °C) δ_C_ 172.5 (C), 144.0 (C), 135.7 (CH), 129.8 (2 × CH), 127.7 (2 × CH), 65.3 (CH_2_), 56.6 (CH), 30.3 (CH_3_), 25.8 (CH_2_), 21.7 (CH_3_). HRMS (ESI) calculated for C_12_H_15_NO_4_SNa [M + Na]^+^ 292.0619, found 292.0618.

***N*-Methyl-*N*-(*p*-fluorobenzoyl)homoserine lactone (7r) and (2*S*)-*N*-methoxymethyl-*N*-(*p*-fluorobenzoyl)homoserine lactone (7s).** Obtained from aldehyde **6g** (65.0 mg, 0.20 mmol) according to method B for the preparation of homoserine lactones. After work-up and solvent evaporation, the residue was purified by radial chromatography (n-hexane/EtOAc, 80:20), yielding the *N*-methylaminolactone **7r** (14.7 mg, 0.06 mmol, 31%) as an amorphous solid and the lactone **7s** (32.0 mg, 0.12 mmol, 60%) as a colorless oil.

**Product 7r.** ^1^H NMR (500 MHz, CDCl_3_, 26 °C) δ_H_ 7.55–7.48 (m, 2H), 7.11 (t, *J_H_*_,*H*_ = 8.6, *J_H_*_,*F*_ = 8.6 Hz, 2H), 5.27–5.03 (m, 1H), 4.61–4.49 (m, 1H), 4.40–4.28 (m, 1H), 3.02 (s, 3H), 2.65–2.40 (m, 2H). ^13^C RMN (125.7 MHz, CDCl_3_, 26 °C) δ_C_ 173.6 (C), 171.5 (C), 163.7 (C, d, *J*_CF_ = 250.8 Hz), 130.9 (CH), 129.8 (2 × CH), 115.7 (2 × CH, d, *J*_CF_ = 19.3 Hz), 65.8 (CH_2_), 55.4 (CH), 35.9 (CH_3_), 25.6 (CH_2_). HRMS (ESI) calculated for C_12_H_12_FNO_3_Na [M + Na]^+^ 260.0693, found 260.0702.

**Product 7s**. [α]_D_: −79 (*c* 0.80, CHCl_3_). IR (CHCl_3_) ν_max_ 1740, 1646, 604, 1420, 1055 cm^−1^. ^1^H NMR (500 MHz, CDCl_3_, 26 °C) δ_H_ 7.51 (t, *J_H_*_,*H*_ = 6.9, *J_H_*_,*F*_ = 6.9 Hz, 2H), 7.11 (br t, *J_H_*_,*H*_ = 8.6, *J_H_*_,*F*_ = 8.6 Hz, 2H), 5.55–5.43 (m, 1H), 5.15–5.02 (m, 1H), 4.80–4.70 (m, 1H), 4.01 (dd, *J* = 12.3, 4.2 Hz, 1H), 3.82 (s, 3H), 3.64 (td, *J* = 12.2, 3.0 Hz, 1H), 2.33–2.10 (m, 2H). ^13^C RMN (125.7 MHz, CDCl_3_, 26 °C) δ_C_ 171.1 (C), 169.8 (C), 164.0 (C, d, *J*_CF_ = 250.8 Hz), 130.1 (CH), 129.9 (2 × CH, d, *J*_CF_ = 4.6 Hz), 115.8 (2 × CH, d, *J*_CF_ = 22.0 Hz), 77.6 (CH_2_), 65.4 (CH_2_), 52.8 (CH_3_), 51.0 (CH), 27.0 (CH_2_). HRMS (ESI) calculated for C_13_H_14_FNO_4_Na [M + Na]^+^ 290.0805, found 290.0805. Anal. Calcd for C_13_H_14_FNO_4_: C, 58.42; H, 5.28; N, 5.24. Found: C, 58.19; H, 5.27; N, 5.11.

***N*-(Phenyloxycarbonyl)-*N*-(methyl)homoserine lactone (7t) and (2*S*)-*N*-(phenyloxycarbonyl)-*N*-(methoxymethyl)homoserine lactone (7u).** Obtained from aldehyde **6p** (64.6 mg; 0.20 mmol) according to method B for the preparation of homoserine lactones. After work-up and solvent evaporation, the residue was purified by radial chromatography (n-hexane/EtOAc, 70:30), yielding the *N*-methylaminolactone **7t** (22.6 mg, 0.10 mmol, 48%) as a crystalline solid, and the lactone **7u** (26.7 mg, 0.10 mmol, 50%) as a colorless oil.

**Product 7t.** Mp 72–74 °C (from n-hexane/EtOAc); [α]_D_: −16 (*c* 0.61, CHCl_3_). IR (CHCl_3_) ν_max_ 3019, 1784, 1713, 1372, 1163 cm^−1^. ^1^H NMR (500 MHz, CD_3_CN, 70 °C) δ_H_ 7.40 (br t, *J* = 8.2 Hz, 2H), 7.25 (br t, *J* = 7.9 Hz, 1H), 7.14 (d, *J* = 8.2 Hz, 2H), 4.85–4.71 (m, 1H), 4.43 (ddd, *J* = 9.0, 8.9, 3.1 Hz, 1H), 4.27 (dt, *J* = 7.1, 9.1 Hz, 1H), 3.06 (s, 3H), 2.62–2.45 (m, 2H). ^13^C RMN (125.7 MHz, CD_3_CN, 70 °C) δ_C_ 175.2 (C), 152.9 (2 x C), 130.5 (2 × CH), 126.7 (CH), 122.9 (2 × CH), 66.8 (CH_2_), 58.4 (CH), 34.3 (CH_3_), 26.6 (CH_2_). HRMS (ESI) calculated for C_12_H_13_NO_4_Na [M + Na]^+^ 258.0742, found 258.0744. Anal. Calcd for C_12_H_13_NO_4_: C, 61.27; H, 5.57; N, 5.95. Found: C, 60.92; H, 5.96; N, 6.05.

**Product 7u.** [α]_D_: −96 (*c* 0.71, CHCl_3_). IR (CHCl_3_) ν_max_ 3019, 1724, 1438, 1415, 1065 cm^−1^. ^1^H NMR (500 MHz, CD_3_CN, 70 °C) δ_H_ 7.45–7.35 (m, 2H), 7.29–7.21 (m, 1H), 7.17–7.09 (m, 2H), 5.62–5.38 (m, 1H), 5.10–4.95 (m, 1H), 4.75–4.60 (m, 1H), 4.01–3.92 (m, 1H), 3.83–3.77 (m, 3H), 3.66–3.56 (m, 1H), 2.30–2.17 (m, 1H), 2.15–2.06 (m, 1H). ^13^C RMN (125.7 MHz, CD_3_CN, 70 °C) δ_C_ 172.3 (C), 154.4 (C), 152.7 (C), 130.6 (2 × CH), 126.8 (CH), 122.9 (2 × CH), 76.0 (CH_2_), 65.5 (CH_2_), 54.5 (CH), 53.3 (CH_3_), 27.5 (CH_2_). HRMS (ESI) calculated for C_13_H_15_NO_5_Na [M + Na]^+^ 288.0848, found 288.0844. Anal. Calcd for C_13_H_15_NO_5_: C, 58.86; H, 5.70; N, 5.28. Found: C, 58.72; H, 5.89; N, 5.16.

### 3.2. Biological Screenings

#### 3.2.1. Quorum-Quenching Activity: Quantification of Violacein Production

The determination of the influence of the compounds on the production of violacein was carried out with the indicator strain C. violaceum CECT 494, according to the procedure reported by Choo et al. [60].

A standard 40 mM solution of the compounds (in DMSO) was diluted in LB medium so that after mixing 1 mL of the diluted solution and 1 mL of the inoculum, a final concentration of 200 μM was obtained (for the most active compounds, concentrations of 100 and 50 μM were also tested). In a similar way, the inoculum was prepared by diluting a preinoculum in LB media, so that after mixing with the product solution, the final inoculum density was 0.7–1 × 10^8^ CFU/mL. A control without treatment (vehicle only) was also prepared to compare the treated and untreated cultures.

The cultures were incubated at 30 °C, with constant shaking for 24 h. For the extraction of the violacein pigment, 1 mL of each culture was subjected to two cycles of centrifugation (Spectrafuge 24D Labnet centrifuge) at 14,000× *g* for 10 min. The first centrifugation cycle allowed removal of the medium (supernatant). The pigment was solubilized by cell treatment with DMSO, and in the second centrifugation cycle, the supernatant containing the dye was separated from the bacteria. Finally, an aliquot of the supernatant (200 μL) was added to 96-well plates, and the absorbance was read at 595 nm in the FLUOstar Omega plate reader, BMG LABTECH (Ortenberg, Germany). The products were tested in triplicate.

#### 3.2.2. Antimicrobial Activity

The susceptibility of Gram-negative Salmonella enterica CECT 456, Campylobacter jejuni CECT 9112, and Pseudomonas aeruginosa CECT 108 and Gram-positive Staphylococcus aureus CECT 794 to the different compounds and concentrations was evaluated either with the broth microdilution method (for the larger lactone libraries) or the disk diffusion method (aldehyde precursors).

*Broth microdilution method*. The procedure was carried out in 96-well plates and followed the EUCAST recommendations for each microbial strain [59,60,61]. Two or three colonies of a microorganism were selected and incubated in liquid medium (MH broth or Sabouraud dextrose) with shaking for 18–24 h at 37 °C. The preinoculum was then subjected to serial dilutions, so that the concentration of the final inoculum was the recommended by EUCAST [59,60,61].

Meanwhile, the compounds were dissolved in DMSO to achieve standard (40 mM) concentrations. The standard was diluted in the liquid medium, so that after taking a 50 μL aliquot and mixing it with the same volume of the inoculum (50 μL) in the plate well, the final product concentration was 200 μM. The inoculated plates were incubated as commented for the preinoculum. Then, the absorbance was measured at 595 nm in the plate reader (FLUOstar Omega, BMG LABTECH). The wells where growth (or turbidity) was not visually observed were subjected to a viable cells count on agar plates. The products were tested in triplicate.

*Disk diffusion method.* To prepare the inoculum, the desired microbial strain was cultured on a Mueller Hinton (MH), LB, or Sabouraud 4% glucose agar plate and incubated for 18–24 h at 37 °C (exhaustion of media procedure). Then, 2–3 colonies isolated from this plate were introduced in a tube containing 3 mL of sterile physiological saline solution until a turbidity of 0.5 MacFarland was reached (measured with a Grant biodensitometer DEN-1B), which corresponded to 1–2 × 10^8^ CFU/mL.

Meanwhile, paper disks containing the potential antimicrobial were prepared. The disks were impregnated with standard (12.5 mM) solutions of the compounds in 7:3 ethanol/DMSO mixtures so that each disk contained 0.25 μmol of the potential antimicrobial. For the positive controls, the disks were impregnated with tetracycline (30 μg, 0.06 μmol). For the negative control, the disks were soaked into the vehicle.

Once the inoculum and the disks were ready, MH-agar plates were inoculated (100 μL of the inoculum), and then the disks were placed on the top. The plates were incubated for 18–24 h at 37 °C, and afterwards the inhibition zones were measured in mm. The products were tested in triplicate.

### 3.3. In Silico ADME Studies

The in silico ADME studies were performed with the SwissADME tool, developed by the Swiss Institute of Bioinformatics [66,67] as commented on in the text and the references.

## 4. Conclusions

In summary, a library of AHLs with a variety of *N*-substituents and a library of AHL aldehyde precursors were prepared in good yields and from readily available, low-cost hydroxyproline substrates. An initial oxidative radical fragmentation, which cleaved the pyrrolidine ring, afforded unusual *N*-substituted 4-oxohomoalanine derivatives in good yields as pure enantiomers. Then, a one-pot reduction–lactonization reaction under two different conditions gave a variety of AHL derivatives, including *N*-substituted amino lactones with *N*-acyl, *N*-carbamoyl and *N*-sulfonyl groups or *N*,*N*-disubstituted amino lactones, in high optical purity.

In order to identify a potential antibiotic, the antimicrobial and quorum-quenching activities of the library were evaluated. To determine the quorum-quenching activity of lactones, the reporter strain of the Gram-negative pathogen *Chromobacterium violaceum* CECT 494 was used, and the generation of the violet pigment violacein was measured. For the first time, sulfonamides and benzamides of related AHLs were compared. Also, for the first time, the activities of *N*,*N-*disubstituted AHLs were compared with those of their *N*-substituted analogs.

Sulfonamides **1a**–**c** and **1e**, the benzyl carbamate **1o**, and the *N*-methyl toluenesulfonamide **1q** were the most active compounds. In contrast, the benzamides **1f-m** displayed little quorum-quenching activity. To our satisfaction, the most active QQ lactones presented low antimicrobial activity against C. *violaceum* CECT 494, a requisite for pure QQ agents. The activity against *S. aureus* CECT 794, *C. jejuni* CECT 9112, *Salmonella enterica* CECT 456, and *P. aeruginosa* CECT 108,was also evaluated, observing low activity.

In contrast, some of the aldehyde precursors displayed antimicrobial activity. The sulfonamide derivatives **2b**–**e** and the dinitrobenzamide **2k** displayed promising activity against *S. aureus* CECT 794 and *C. jejuni* CECT 9112, compared with the respective lactones. It suggests an important role of the 4-carbonyl group in the interaction with biological receptors.

Finally, in silico ADME studies carried out with the SwissADME tools suggest that these compounds have low toxicity and favorable ADME properties.

## Figures and Tables

**Table 1:** Synthesis of aldehydes **6a**–**p** by oxidative radical scission of the hydroxypyrrolidines **5a**–**p**.

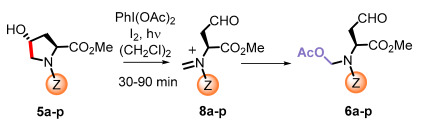			
entry	substrate	Z	Product (%)
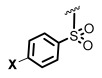			
1	**5a**	X = H	**6a** (80)
2	**5b**	X = Me	**6b** (73)
3	**5c**	X = Cl	**6c** (73)
4	**5d**	X = I	**6d** (86)
5	**5e**	X = NO_2_	**6e** (71)
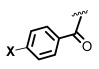			
6	**5f**	X = H	**6f** (88)
7	**5g**	X = F	**6g** (85)
8	**5h**	X = Cl	**6h** (82)
9	**5i**	X = I	**6i** (73)
10	**5j**	X = NO_2_	**6j** (87)
11	**5k**	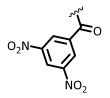	**6k** (78)
12	**5l**	Ac	**6l** (72)
13	**5m**	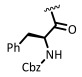	**6m** (70)
14	**5n**	Boc	**6n** (70)
15	**5o**	Cbz	**6o** (56)
16	**5p**	CO_2_Ph	**6p** (79)

**Table 2:** Synthesis of lactones **7a**–**p** by reduction–cyclization of aldehydes **6a**–**p**.

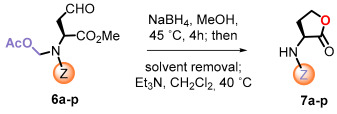			
entry	substrate	Z	Product (%)
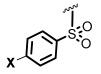			
1	**6a**	X = H	**7a** (63)
2	**6b**	X = Me	**7b** (65)
3	**6c**	X = Cl	**7c** (29)
4	**6d**	X = I	**7d** (52)
5	**6e**	X = NO_2_	**7e** (56)
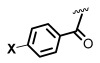			
6	**6f**	X = H	**7f** (67)
7	**6g**	X = F	**7g** (67)
8	**6h**	X = Cl	**7h** (40)
9	**6i**	X = I	**7i** (59)
10	**6j**	X = NO_2_	**7j** (53)
11	**6k**	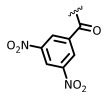	**7k** (34)
12	**6l**	Ac	**7l** (43)
13	**6m**	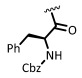	**7m** (57)
14	**6n**	Boc	**7n** (70)
15	**6o**	Cbz	**7o** (30)
16	**6p**	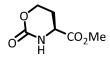	**7p** (23)

**Table 3:** Results of the IQS detection assay in the *C. violaceum* CECT 494 strain.

Compound	C (μM)	Inhibition of Violacein Production (%) μ ± DE ^a^
**7a**	200	**70.42 ± 3.55**
**7a**	100	**57.27 ± 5.40**
**7a**	50	**41.94 ± 4.64**
**7b**	200	**62.10 ± 3.89**
**7b**	100	**49.59 ± 6.16**
**7b**	50	31.73 ± 7.31
**7c**	200	**51.55 ± 3.62**
**7c**	100	**44.39 ± 7.43**
**7c**	50	39.42 ± 4.90
**7d**	200	26.37 ± 10.15
**7e**	200	**48.74 ± 5.69**
**7e**	100	36.96 ± 7.51
**7f**	200	4.16 ± 3.41/NS
**7g**	200	26.21 ± 7.63
**7h**	200	32.80 ± 3.38
**7i**	200	23.34 ± 4.73
**7j**	200	NI
**7k**	200	6.98 ± 3.02
**7l**	200	NI
**7m**	200	NI
**7n**	200	30.47 ± 1.82
**7o**	200	**67.06 ± 3.82**
**7o**	100	**51.94 ± 2.21**
**7o**	50	**43.76 ± 1.28**
**7p**	NT	NT
**7q**	200	**43.52 ± 4.72**
**7r**	200	11.79 ± 3.77/NS
**7s**	200	8.19 ± 1.47/NS
**7t**	200	7.36 ± 2.25/NS
7u	200	10.87 ± 3.72/NS

^a^ The results are shown as percentages of inhibition of violacein production by treatment with the α-amino-γ-lactone derivatives, compared to an untreated control. The results are given as the average percentage of inhibition ± standard deviation (n = 3). The values show significant differences (*p* < 0.05) with respect to the non-treated control according to the one-way ANOVA statistical procedure. NI: non-inhibition. NS = no significant differences with untreated control. NT = Not tested. The most relevants results are in bold, and for inhibition >50% are highlighted in red.

**Table 4:** Antimicrobial activity of aldehydes **6a**–**6p** against bacterial pathogens using the disk diffusion assay.

Compound	Zone of Inhibition (mm)			
	*S. aureus* (CECT 794)	*C. jejuni* (CECT9112)	*S. enterica* (CECT456)	*P. aeruginosa* (CECT108)
**6a**	------	------	------	------
**6b**	13.30 ± 0.60	15.00 ± 1.20	NI	NI
**6c**	13.70 ± 0.30	14.00 ± 0.00	NI	NI
**6d**	12.00 ± 1.00	12.00 ± 1.20	NI	NI
**6e**	12.30 ± 0.30	17.00 ± 0.60	NI	NI
**6f**	NI	NI	NI	NI
**6g**	NI	NI	NI	NI
**6h**	NI	NI	NI	NI
**6i**	NI	NI	NI	NI
**6j**	NI	NI	NI	NI
**6k**	13.00 ± 0.90	16.00 ± 0.60	NI	NI
**6l**	NI	NI	NI	NI
**6m**	NI	NI	NI	NI
**6n**	NI	NI	NI	NI
**6o**	NI	NI	NI	NI
**6p**	NI	NI	NI	NI
Tetracycline	26.00 ± 1.00	25.00 ± 1.20	24.00 ± 1.60	15.00 ± 2.90

Results as average of inhibition zone ± standard deviation (n = 3) in millimeters (mm). NI: No inhibition.

**Table 5:** Summary of in silico physicochemical properties for lactones **7a**–**o** and **7q**–**u** and selected aldehydes **6a**–**e** and **6k**.

Compound	MW (g/mol)	N° H-Bond Donors	N° H-Bond Acceptors	N° Rotable Bonds	TPSA (Å²)	LogP_o/w_	LogS (SILICOS-IT)
**7a**	241.26	1	5	3	80.85	0.90	−2.89 Soluble
**7b**	255.29	1	5	3	80.85	1.23	−3.27 Soluble
**7c**	275.71	1	5	3	80.85	1.41	−3.50 Soluble
**7d**	367.16	1	5	3	80.85	1.53	−3.78 Soluble
**7e**	286.26	1	7	4	126.67	0.22	−2.74 Soluble
**7f**	205.21	1	3	3	55.40	1.26	−2.91 Soluble
**7g**	223.20	1	4	3	55.40	1.58	−3.19 Soluble
**7h**	239.65	1	3	3	55.40	1.80	−3.53 Soluble
**7i**	331.11	1	3	3	55.40	1.94	−3.83 Soluble
**7j**	250.21	1	5	4	101.22	0.70	−2.77 Soluble
**7k**	295.21	1	7	5	147.04	0.07	−2.62 Soluble
**7l**	143.14	1	3	2	55.40	−0.05	−0.75 Soluble
**7m**	382.41	2	5	10	93.73	2.18	−5.96 Mod. Sol.
**7n**	201.22	1	4	4	64.63	0.95	−1.34 Soluble
**7o**	235.24	1	4	5	64.63	1.42	−3.06 Soluble
**7p**	159.14	1	4	2	64.63	0.01	−0.50 Soluble
**7q**	269.32	0	5	3	72.06	1.45	−2.94 Soluble
**7r**	237.23	0	4	3	46.61	1.75	−2.86 Soluble
**7s**	267.25	0	5	5	55.84	1.74	−3.00 Soluble
**7t**	235.24	0	4	4	55.84	1.55	−2.33 Soluble
**7u**	293.27	0	6	7	82.14	1.54	−2.40 Soluble
**6a**	343.35	0	8	10	115.43	0.77	−2.58 Soluble
**6b**	357.38	0	8	10	115.43	1.11	−2.96 Soluble
**6c**	377.80	0	8	10	115.43	1.32	−3.17 Soluble
**6d**	469.25	0	8	10	115.43	1.28	−3.42 Soluble
**6e**	388.35	0	10	11	161.25	0.22	−2.41 Soluble
**6k**	397.29	0	10	12	181.62	−0.04	−2.29 Soluble

**Table 6:** Summary of in silico pharmacological properties for lactones **7a**–**p** and **7q**–**u** and selected aldehydes **6a**–**e** and **6k**.

Compound	GI Absorp	BBB Permeant	P-gp Substrate	CYP Inhibitor	Log Kp (cm/s)	Druglikeness: Lipinki, Ghose, etc.	Abbot Bio. Score	PAINS/Brenk Alerts
**7a**	High	No	No	No	−7.08	Yes, 0 violations	0.55	0 alerts
**7b**	High	No	No	No	−6.91	Yes, 0 violations	0.55	0 alerts
**7c**	High	No	No	No	−6.85	Yes, 0 violations	0.55	0 alerts
**7d**	High	No	No	No except CYP2C19	−7.39	Yes, 0 violations	0.55	P: 0 alerts; B: Iodo
**7e**	High	No	No	No	−7.48	Yes, 0 violations	0.55	P: 0 alerts; B: Nitro
**7f**	High	Yes	No	No	−6.64	Yes, 0 violations	0.55	0 alerts
**7g**	High	Yes	No	No	−6.67	Yes, 0 violations	0.55	0 alerts
**7h**	High	Yes	No	No except P450 1A2	−6.41	Yes, 0 violations	0.55	0 alerts
**7i**	High	Yes	No	No except CYP1A2	−6.94	Yes, 0 violations	0.55	P:0 alerts; B: Iodine
**7j**	High	No	No	No	−7.04	Yes, 0 violations	0.55	P:0 alerts; B: Nitro
**7k**	Low	No	No	No	−7.43	No, Veber rules (TPSA > 140) and Egan: TPSA > 131	0.55	P:0 alerts; B: Nitro
**7l**	High	No	Yes	No	−7.44	No, Muegge and Ghose, low MW	0.55	0 alerts
**7m**	High	No	Yes	No except CYP3A4	−6.60	Yes, 0 violations	0.55	P:0 alerts; B: >2 esters
**7n**	High	No	No	No	−6.80	Yes, 0 violations	0.55	P: 0 alerts; B: >2 esters
**7o**	High	Yes	No	No	−6.65	Yes, 0 violations	0.55	P: 0 alerts; B: >2 esters
**7p**	High	No	No	No	−7.25	No, Muegge and Ghose, low MW	0.55	P: 0 alerts; B: >2 esters
**7q**	High	Yes	No	No except CYP2C19	−6.86	Yes, 0 violations	0.56	0 alerts
**7r**	High	Yes	No	No	−6.63	Yes, 0 violations	0.55	0 alerts
**7s**	High	Yes	No	No	−6.85	Yes, 0 violations	0.55	0 alerts
**7t**	High	Yes	No	No	−6.48	Yes, 0 violations	0.55	0 alerts
**7u**	High	No	No	No	−6.84	Yes, 0 violations	0.55	0 alerts
**6a**	High	No	No	No except CYP2C19	−8.07	Yes, 0 violations	0.55	P: 0 alerts; B: aldehyde
**6b**	High	No	No	Same as **6a**	−7.90	Yes, 0 violations	0.55	Same as **6a**
**6c**	High	No	No	Same as **6a**	−7.84	Yes, 0 violations	0.55	Same as **6a**
**6d**	High	No	No	Same as **6a**	−8.38	Yes, 0 violations	0.55	Same as **6a**
**6e**	Low	No	Yes	No	−8.47	No, high TPSA and rotors, O > 10	0.55	P: 0 alerts; B: aldehyde, NO_2_
**6k**	Low	No	Yes	No	−8.43	Same as **6e**	0.55	Same as **6e**

## Data Availability

Data contained within the article.

## Supplementary Materials

The following supporting information can be downloaded at: https://www.mdpi.com/article/10.3390/ijms26041775/s1.
