# Supplementary material for: Short Synthesis of Structurally Diverse N-Acylhomoserine Lactone Analogs and Discovery of Novel Quorum Quenchers Against Gram-Negative Pathogens
Source: Int J Mol Sci. 2025 Feb 19;26(4):1775. doi: 10.3390/ijms26041775 (PMC11855090; doi:10.3390/ijms26041775)
Supplement: Supplementary file 1 [file ijms-26-01775-s001.zip › ijms-3432111-supplementary.pdf]

International Journal of
Molecular Sciences

MDPI

Supporting Information

Short Synthesis of Structurally Diverse *N*-Acylhomoserine Lactone Analogs and Discovery of Novel Quorum Quenchers Against Gram-Negative Pathogens

Marina Porras, Dácil Hernández * and Alicia Boto *

Instituto de Productos Naturales y Agrobiología del CSIC, Avda. Astrofísico Fco. Sánchez, 3, 38206 La Laguna, Tenerife, Spain; mporras@ipna.csic.es
* Correspondence: dacil@ipna.csic.es (D.H.); alicia@ipna.csic.es (A.B.)

Table of contents

General procedure for preparation of *trans*-4-hydroxy-L-proline substrates **5a-p**	Page 2
Characterization data for *trans*-4-hydroxy-L-proline substrates **5a-p**	Page 2
NMR data for new compounds	Page 6

 https://doi.org/10.3390/ijms26041775

General procedures for the protection of the amine group in 4-*trans*-L-hydroxyproline.

General method for the preparation of sulfonamides (method A). A solution of commercial *trans*-4-hydroxy-L-proline methyl ester hydrochloride (3.0 g, 16.5 mmol) in dichloromethane (25 ml) was cooled to 0 °C and treated with Et_3N (5.0 g, 6.9 mL, 49.5 mmol) and the corresponding sulfonyl chloride (24.8 mmol). After stirring for 20 h at room temperature, the mixture was acidified with 10% aqueous HCl solution and extracted with CH_2Cl_2. The organic phase was dried and concentrated as usual, and the crude was purified by silica gel column chromatography (n-hexane/ethyl acetate mixtures) to obtain the corresponding sulfonamide.

General method for the preparation of amides (method B). Commercial *trans*-4-hydroxy-L-proline methyl ester hydrochloride (3.0 g, 16.5 mmol) was added to a 1:1 mixture of THF and saturated $NaHCO_3$ aqueous solution (40 mL). The reaction was cooled to 0 °C, the corresponding acyl or benzoyl chloride (33.0 mmol) was injected dropwise and the mixture was allowed to react for 20 h. Then the mixture was acidified with 10% aqueous HCl and extracted with ethyl acetate. The combined organic extracts were treated as in method A to obtain the corresponding amide, after purification by silica gel column chromatography.

General method for the preparation of carbamates (method C). Commercial *trans*-4-hydroxy-L-proline methyl ester hydrochloride (3.0 g, 16.5 mmol) was added to a 1:1 mixture of THF and saturated $NaHCO_3$ aqueous solution (40 mL). The reaction was cooled to 0 °C, and the chloroformate reagent (18.2 mmol) was injected dropwise. The mixture was allowed to react for 20 h, poured into 10% aqueous HCl and extracted with ethyl acetate. The combined organic extracts were treated as in method A to obtain the corresponding amide, after purification by silica gel column chromatography.

(2*S*,4*R*)-4-Hydroxy-1-(phenylsulfonyl)-L-proline methyl ester (5a). Obtained from commercial *trans*-4-hydroxy-L-proline methyl ester hydrochloride according to the method A, using phenyl sulfonyl chloride (4.36 g, 3.2 mL) as reagent. After purification (*n*-hexane/ethyl acetate, 50:50), sulfonamide **5a** (2.54 g, 54%) was isolated as a crystalline solid: mp 115–117 ºC (from n-hexane/EtOAc); $[\alpha]_D$: –115 (*c* 0.10, $CHCl_3$). IR ($CHCl_3$) ν_{max} 3464, 1724, 1332, 1153, 1073 cm^{-1}. ^{1}H NMR (500 MHz, $CDCl_3$, 26 °C) δ_H 7.90 (br d, *J* = 7.1 Hz, 2H), 7.59 (dd, *J* = 7.6, 7.2 Hz, 2H), 7.52 (dd, *J* = 7.6, 7.2 Hz, 2H), 4.47–4.44 (m, 2H), 3.72 (s, 3H), 3.60 (dd, *J* = 11.2, 4.2 Hz, 1H), 3.43 (dt, *J* = 11.0, 2.3 Hz, 1H), 2.25–2.18 (m, 1H), 2.16–2.09 (m, 1H), 1.73–1.70 (br b, 1H). ^{13}C RMN (125.7 MHz, $CDCl_3$, 26 °C) δ_C 172.5 (C), 138.5 (C), 133.0 (CH), 129.1 (2 × CH), 127.9 (2 × CH), 70.4 (CH), 59.6 (CH), 56.5 (CH_2), 52.6 (CH_3), 39.8 (CH_2). HRMS (ESI) calculated for $C_{12}H_{15}NO_5SNa$ [M + Na]$^+$ 308.0569, found 308.0574. Anal. Calcd for $C_{12}H_{15}NO_5S$: C, 50.52; H, 5.30; N, 4.91; S, 11.24. Found: C, 50.29; H, 5.39; N, 5.28; S, 11.52.

(2*S*,4*R*)-4-Hydroxy-1-(toluenesulfonyl)-L-proline methyl ester (5b). Obtained from commercial *trans*-4-hydroxy-L-proline methyl ester hydrochloride according to the method A, using *p*-toluensulfonyl chloride (4.72 g) as reagent. After purification (*n*-hexane/ethyl acetate, 50:50), sulfonamide **5b** (2.47 g, 50%) was isolated as a crystalline solid: mp 98–100 ºC (from n-hexane/EtOAc); $[\alpha]_D$: –88 (*c* 0.50, $CHCl_3$). IR ($CHCl_3$) ν_{max} 1730, 1423, 1155, 1081 cm^{-1}. ^{1}H NMR (500 MHz, $CDCl_3$, 26 °C) δ_H 7.76 (br d, *J* = 8.4 Hz, 2H), 7.31 (br d, *J* = 7.9 Hz, 2H), 4.46–4.42 (m, 1H), 4.39 (t, *J* = 7.7 Hz, 1H), 3.74 (s, 3H), 3.59 (dd, *J* = 11.4, 4.0 Hz, 1H), 3.37 (dt, *J* = 11.4, 2.0 Hz, 1H), 2.42 (s, 3H), 2.23–2.17 (m, 1H), 2.09 (ddd, *J* = 13.1, 8.3, 4.6 Hz, 1H), 1.91–1.84 (br b, 1H). ^{13}C RMN (125.7 MHz, $CDCl_3$, 26 °C) δ_C 172.7 (C), 144.0 (C), 134.9 (C), 129.8 (2 × CH), 127.9 (2 × CH), 70.3 (CH), 59.5 (CH), 56.5 (CH_2), 52.7 (CH_3), 39.6 (CH_2), 21.7 (CH_3). HRMS (ESI) calculated for $C_{13}H_{17}NO_5SNa$ [M + Na]$^+$ 322.0725, found 322.0727. Anal. Calcd for $C_{13}H_{17}NO_5S$: C, 52.16; H, 5.72; N, 4.68; S, 10.71. Found: C, 52.04; H, 5.75; N, 4.56; S, 10.52.

(2*S*,4*R*)-1-(*p*-Chlorophenylsulfonyl)-4-hydroxy-L-proline methyl ester (5c). Obtained from commercial *trans*-4-hydroxy-L-proline methyl ester hydrochloride according to method A, using *p*-chlorophenylsulfonyl chloride (5.22 g) as reagent. After purification (n-hexane/ethyl acetate, 30:70), sulfonamide **5c** (5.12 g, 97%) was isolated as a crystalline solid: mp 133–135 ºC (from n-hexane/EtOAc); $[\alpha]_D$: –98 (*c* 0.64, $CHCl_3$). IR ($CHCl_3$) ν_{max} 3465, 1718, 1350, 1159, 1076 cm^{-1}. ^{1}H NMR (500 MHz, $CDCl_3$, 26 °C) δ_H 7.82 (d, *J* = 8.7 Hz, 2H), 7.49 (d, *J* = 8.7 Hz, 2H), 4.47–4.43 (m, 1H), 4.41 (t, *J* = 8.1 Hz, 1H), 3.74 (s, 3H), 3.57 (dd, *J* = 11.4, 3.9 Hz, 1H), 3.41 (dt, *J* = 11.4, 1.8 Hz, 1H), 2.24 (dddd, *J* = 13.3, 7.6, 2.7, 1.8 Hz, 1H), 2.09 (ddd, *J* = 13.3, 8.6, 4.5 Hz, 1H), 2.00 (d, *J* = 3.7 Hz, 1H). ^{13}C RMN (125.7 MHz, $CDCl_3$, 26 °C) δ_C 172.6 (C), 139.6 (C), 136.6 (C), 129.4 (2 × CH), 129.3 (2 × CH), 70.2 (CH), 59.6 (CH), 56.6 (CH_2), 52.8 (CH_3), 39.6 (CH_2).

HRMS (ESI) calculated for $C_{12}H_{14}ClNO_5SNa$ [M + Na]$^+$ 342.0179, found 342.0182. Anal. Calcd for $C_{12}H_{14}ClNO_5S$: C, 45.08; H, 4.41; N, 4.38; S, 10.03. Found: C, 45.33; H, 4.32; N, 4.36; S, 9.65.

(2*S*,4*R*)-4-Hydroxy-1-(*p*-iodophenylsulfonyl)-L-proline methyl ester (5d). Obtained from commercial *trans*-4-hydroxy-L-proline methyl ester hydrochloride according to the method A, using *p*-iodophenylsulfonyl chloride (7.49 g) as reagent. After purification (n-hexane/ethyl acetate, 40:60), sulfonamide **5d** (6.18 g, 91%) was isolated as a crystalline solid: mp 98–100 ºC (from n-hexane/EtOAc); $[\alpha]_D$: –92 (*c* 0.36, $CHCl_3$). IR ($CHCl_3$) ν_{max} 3676, 3460, 1732, 1394, 1225, 1076 cm^{-1}. ^{1}H NMR (500 MHz, $CDCl_3$, 26 °C) δ_H 7.86 (br d, *J* = 7.6 Hz, 2H), 7.59 (dd, *J* = 8.7, 2.0 Hz, 2H), 4.48–4.44 (m, 1H), 4.40 (td, *J* = 8.0, 5.0 Hz, 1H), 3.74 (s, 3H), 3.60–3.54 (m, 1H), 3.40 (br d, *J* = 11.4 Hz, 1H), 2.27–2.21 (m, 1H), 2.15–2.03 (m, 1H). ^{13}C RMN (125.7 MHz, $CDCl_3$, 26 °C) δ_C 172.6 (C), 138.3 (2 × CH), 137.7 (C), 129.2 (2 × CH), 100.7 (C), 70.1 (CH), 59.6 (CH), 56.6 (CH_2), 52.8 (CH_3), 39.6 (CH_2). HRMS (ESI) calculated for $C_{12}H_{14}INO_5SNa$ [M + Na]$^+$ 433.9535, found 433.9534. Anal. Calcd for $C_{12}H_{14}INO_5S$: C, 35.05; H, 3.43; N, 3.41; S, 7.80. Found: C, 34.98; H, 3.54; N, 3.62; S, 7.85.

(2*S*,4*R*)-4-Hydroxy-1-(*p*-nitrophenylsulfonyl)-L-proline methyl ester (5e). Obtained from commercial *trans*-4-hydroxy-L-proline methyl ester hydrochloride according to the method A, using *p*-nitrophenylsulfonyl chloride (5.24 g) as reagent. After purification (n-hexane/ethyl acetate, 40:60), sulfonamide **5e** (5.07 g, 93%) was isolated as a crystalline solid: mp 154–156 ºC (from n-hexane/EtOAc); $[\alpha]_D$: –98 (*c* 0.50, $CHCl_3$). IR ($CHCl_3$) ν_{max} 3670, 3400, 1731, 1527, 1218, 1075 cm^{-1}. ^{1}H NMR (500 MHz, (CD_3)OD, 26 °C) δ_H 8.44 (d, *J* = 9.0 Hz, 2H), 8.14 (d, *J* = 9.2 Hz, 2H), 4.45–4.40 (m, 1H), 4.36 (t, *J* = 8.5 Hz, 1H), 4.06 (d, *J* = 3.1 Hz, 1H), 3.71 (s, 3H), 3.62 (dd, *J* = 11.1, 3.8 Hz, 1H), 3.46 (dt, *J* = 11.1, 1.8 Hz, 1H), 2.20 (dddd, *J* = 13.1, 7.5, 2.7, 1.8 Hz, 1H), 2.12–2.05 (m, 1H). ^{13}C RMN (125.7 MHz, (CD_3)OD, 26 °C) δ_C 172.9 (C), 151.2 (C), 144.8 (C), 130.0 (2 × CH), 125.0 (2 × CH), 70.2 (CH), 60.9 (CH), 57.7 (CH_2), 52.6 (CH_3), 40.2 (CH_2). HRMS (ESI) calculated for $C_{12}H_{14}N_2O_7SNa$ [M + Na]$^+$ 353.0419, found 353.0417. Anal. Calcd for $C_{12}H_{14}N_2O_7S$: C, 43.64; H, 4.27; N, 8.48; S, 9.71. Found: C, 43.65; H, 4.25; N, 8.21; S, 9.77.

(2*S*,4*R*)-1-Benzoyl-4-hydroxy-L-proline methyl ester (5f). Obtained from commercial *trans*-4-hydroxy-L-proline methyl ester hydrochloride according to the method B, using benzoyl chloride (5.0 g) as reagent. After purification (n-hexane/ethyl acetate, 20:80), benzamide **5f** (6.20 g, 90%) was isolated as a crystalline solid. The spectroscopic data agree with those reported in the literature [84].

(2*S*,4*R*)-1-(*p*-Fluorobenzoyl)-4-hydroxy-L-proline methyl ester (5g). Obtained from commercial *trans*-4-hydroxy-L-proline methyl ester hydrochloride according to the method B, using *p*-fluorobenzoyl chloride (2.88 g, 2.1 mL) as reagent. After purification (n-hexane/ethyl acetate, 40:60), benzamide **5g** (4.02 g, 91%) was isolated as a crystalline solid: mp 148–150 ºC (from n-hexane/EtOAc); $[\alpha]_D$: –129 (*c* 0.48, $CHCl_3$). IR ($CHCl_3$) ν_{max} 3447, 1735, 1603, 1442, 1084 cm^{-1}. ^{1}H NMR (500 MHz, $CDCl_3$, 26 °C) δ_H 7.62–7.50 (2H, m), 7.12–7.00 (2H, m), 4.82 (t, *J* = 8.4 Hz, 1H), 4.52–4.42 (m, 1H), 3.79 (m, 1H), 3.76 (s, 3H), 3.50 (d, *J* = 11.3 Hz, 1H), 3.47–3.40 (br b, 1H), 2.36 (ddt, *J* = 13.4, 7.9, 2.2 Hz, 1H), 2.12 (ddd, *J* = 13.4, 8.7, 4.6 Hz, 1H). ^{13}C RMN (125.7 MHz, $CDCl_3$, 26 °C) δ_C 172.9 (C), 169.4 (C), 164.0 (C, d, J_{CF} = 250.4 Hz), 131.7 (C), 130.0 (2 × CH, d, J_{CF} = 9.2 Hz), 115.5 (2 × CH, d, J_{CF} = 21.8 Hz), 70.4 (CH), 58.14 (CH), 58.10 (CH_2), 52.5 (CH_3), 37.8 (CH_2). HRMS (ESI) calculated for $C_{13}H_{14}FNO_4Na$ [M + Na]$^+$ 290.0805, found 290.0806. Anal. Calcd for $C_{13}H_{14}FNO_4$: C, 58.42; H, 5.28; N, 5.24. Found: C, 58.61; H, 4.95; N, 5.45.

(2*S*,4*R*)-1-(*p*-Chlorobenzoyl)-4-hydroxy-L-proline methyl ester (5h). Obtained from commercial *trans*-4-hydroxy-L-proline methyl ester hydrochloride according to the method B, using *p*-chlorobenzoyl chloride (3.18 g) as reagent. After purification (n-hexane/ethyl acetate, 40:60), benzamide **5h** (3.84 g, 82%) was isolated as a crystalline solid: mp 124–126 ºC (from n-hexane/EtOAc); $[\alpha]_D$: –126 (*c* 0.38, $CHCl_3$). IR ($CHCl_3$) ν_{max} 3434, 3215, 1757, 1605, 1436, 1203, 1087 cm^{-1}. ^{1}H NMR (500 MHz, $CDCl_3$, 26 °C) δ_H 7.47 (d, *J* = 8.4 Hz, 2H), 7.36 (d, *J* = 8.5 Hz, 2H), 4.79 (t, *J* = 8.4 Hz, 1H), 4.50–4.40 (m, 1H), 3.77–3.74 (m, 4H), 3.46 (d, *J* = 11.3 Hz, 1H), 3.10–2.90 (br b, 1H), 2.39–2.30 (m, 1H), 2.08 (ddd, *J* = 13.5, 8.9, 4.4 Hz, 1H). ^{13}C RMN (125.7 MHz, $CDCl_3$, 26 °C) δ_C 172.8 (C), 169.3 (C), 136.8 (C), 134.0 (C), 129.1 (2 × CH), 128.7 (2 × CH), 70.4 (CH), 58.1 (CH), 58.0 (CH_2), 52.6 (CH_3), 37.9 (CH_2). HRMS (ESI) calculated for $C_{13}H_{14}ClNO_4$ [M]$^+$ 283.0611, found 283.0615. Anal. Calcd for $C_{13}H_{14}ClNO_4$: C, 55.04; H, 4.97; N, 4.94. Found: C, 55.11; H, 4.67; N, 4.88.

(2*S*,4*R*)-4-Hydroxy-1-(*p*-iodobenzoyl)-L-proline methyl ester (5i). Obtained from commercial *trans*-4-hydroxy-L-proline methyl ester hydrochloride according to the method B, using *p*-iodobenzoyl chloride (4.96

g) as reagent. After purification (n-hexane/ethyl acetate, 40:60), benzamide **5i** (4.65 g, 75%) was isolated as a crystalline solid: mp 143–145 ºC (from n-hexane/EtOAc); $[\alpha]_D$: –94 (*c* 0.48, $CHCl_3$). IR ($CHCl_3$) ν_{max} 3300, 1749, 1527, 1157, 1090 cm^{-1}. ^{1}H NMR (500 MHz, $CDCl_3$, 26 °C) δ_H 7.73 (d, *J* = 8.4 Hz, 2H), 7.25 (d, *J* = 8.4 Hz, 2H), 4.77 (t, *J* = 8.4 Hz, 1H), 4.46–4.40 (m, 1H), 3.73 (s, 3H), 3.74–3.71 (m, 1H), 3.45 (d, *J* = 11.3 Hz, 1H), 2.91 (d, *J* = 3.8 Hz, 1H), 2.34 (ddt, *J* = 13.5, 8.1, 2.2 Hz, 1H), 2.07 (ddd, *J* = 13.4, 8.9, 4.5 Hz, 1H). ^{13}C RMN (125.7 MHz, $CDCl_3$, 26 °C) δ_C 172.8 (C), 169.5 (C), 137.7 (2 × CH), 135.0 (C), 129.3 (2 × CH), 97.2 (C), 70.4 (CH), 58.1 (CH), 58.0 (CH_2), 52.6 (CH_3), 37.9 (CH_2). HRMS (ESI) calculated for $C_{13}H_{14}INO_4Na$ [M + Na]$^+$ 397.9865, found 397.9870. Anal. Calcd for $C_{13}H_{14}INO_4$: C, 41.62; H, 3.76; N, 3.73. Found: C, 41.61; 3, 4.95; N, 3.45.

(2*S*,4*R*)-4-Hydroxy-1-(*p*-nitrobenzoyl)-L-proline methyl ester (5j). Obtained from commercial *trans*-4-hydroxy-L-proline methyl ester hydrochloride according to the method B, using *p*-nitrobenzoyl chloride (3.37 g) as reagent. After purification (n-hexane/ethyl acetate, 50:50), benzamide **5j** (2.52 g, 52%) was isolated as a crystalline solid: mp 140–142 ºC (from n-hexane/EtOAc); $[\alpha]_D$: –135 (*c* 0.34, $CHCl_3$). IR ($CHCl_3$) ν_{max} 3361, 1735, 1523, 1255, 1079 cm^{-1}. ^{1}H NMR (500 MHz, $CDCl_3$, 26 °C) mixture of rotamers with overlapping signals, only those of the major rotamer are described δ_H 8.26 (d, *J* = 8.9 Hz, 2H), 7.72 (d, *J* = 8.9 Hz, 2H), 4.82 (t, *J* = 8.4 Hz, 1H), 4.54–4.49 (m, 1H), 3.78 (s, 3H), 3.76 (dd, *J* = 11.3, 4.0 Hz, 1H), 3.46–3.39 (m, 1H), 2.43–2.35 (m, 1H), 2.18–2.10 (m, 1H). ^{13}C RMN (125.7 MHz, $CDCl_3$, 26 °C) δ_C 172.4 (C), 168.2 (C), 149.1 (C), 141.9 (C), 128.6 (2 × CH), 123.9 (2 × CH), 70.5 (CH), 58.1 (CH), 57.8 (CH_2), 52.6 (CH_3), 38.1 (CH_2). HRMS (ESI) calculated for $C_{13}H_{14}N_2O_6Na$ [M + Na]$^+$ 317.0750, found 317.0748. Anal. Calcd for $C_{13}H_{14}N_2O_6$: C, 53.06; H, 4.80; N, 9.52. Found: C, 52.71; H, 4.90; N, 9.89.

(2*S*,4*R*)-4-Hydroxy-1-(3,5-dinitrobenzoyl)-L-proline methyl ester (5k). Obtained from commercial *trans*-4-hydroxy-L-proline methyl ester hydrochloride according to the method B, using 3,5-dinitrobenzoyl chloride (4.19 g) as reagent. After purification (n-hexane/ethyl acetate, 30:70), benzamide **5k** (4.76 g, 85%) was isolated as a crystalline solid: mp 137–139 ºC (from n-hexane/EtOAc); $[\alpha]_D$: –160 (*c* 0.82, $CHCl_3$). IR ($CHCl_3$) ν_{max} 3628, 3069, 1739, 1417, 1171, 1064 cm^{-1}. ^{1}H NMR (500 MHz, $CDCl_3$, 26 °C) δ_H 9.11 (t, *J* = 2.1 Hz, 1H), 8.76 (d, *J* = 2.1 Hz, 2H), 4.84 (t, *J* = 8.5 Hz, 1H), 4.62–4.55 (m, 1H), 3.89 (dd, *J* = 11.0, 3.8 Hz, 1H), 3.80 (s, 3H), 3.53 (s, 1H), 3.49 (d, *J* = 11.0 Hz, 1H), 2.49–2.41 (m, 1H), 2.19 (ddd, *J* = 13.4, 9.0, 4.5 Hz, 1H). ^{13}C RMN (125.7 MHz, $CDCl_3$, 26 °C) δ_C 172.2 (C), 165.4 (C), 148.6 (2 × C), 139.0 (C), 128.0 (2 × CH), 120.5 (CH), 70.5 (CH), 58.4 (CH), 57.9 (CH_2), 52.9 (CH_3), 37.9 (CH_2). HRMS (ESI) calculated for $C_{13}H_{13}N_3O_8$ [M]$^+$ 339.0703, found 339.0705. Anal. Calcd for $C_{13}H_{13}N_3O_8$: C, 46.02; H, 3.86; N, 12.39. Found: C, C, 45.93; H, 4.02; N, 12.45.

(2*S*,4*R*)-1-Acetyl-4-hydroxy-L-proline methyl ester (5l). To a solution of *trans*-4-hydroxy-L-proline methyl ester hydrochloride (0.50 g, 2.75 mmol) in methanol (14 mL) were added triethylamine (1.5 mL, 11.0 mmol) and acetic anhydride (1.6 mL, 16.50 mmol). The mixture was stirred at room temperature for 2 h. Then it was concentrated under vacuum, and the residue was purified by silica gel column chromatography (EtOAc:MeOH, 40:60), yielding the acetamide **5l** (508.9 mg, 2.72 mmol, 99%), whose characterization data were already reported [57].

(2*S*,4*R*)-1-(*N*-Benzyloxycarbonylphenylalanyl)-4-hydroxy-L-proline methyl ester (5m). A solution of *trans*-4-hydroxy-L-proline methyl ester hydrochloride (499.5 mg, 2.75 mmol) and *N* (benzyloxycarbonyl)-L-phenylalanine (822.6 mg, 2.75 mmol) in dichloromethane (5 mL) was cooled to 0°C, followed by addition of *O*-(benzotriazol-1-yl)-*N,N,N′,N′*-tetramethyluronium hexafluorophosphonate (HBTU, 1.15 g, 3.03 mmol) and dropwise injection of *N,N*-diisopropylethylamine (DIPEA, 1.07 g, 1.40 mL, 8.25 mmol). The mixture was stirred at 0°C for 30 min and then allowed to reach room temperature for 2 h. Afterwards the solution was poured onto water and washed with a saturated $NaHCO_3$ aqueous solution and 10% aqueous HCl. The organic phase was dried over anhydrous sodium sulfate, filtered and concentrated under vacuum. The residue was purified by silica gel column chromatography (n-hexane/EtOAc, 40:60), yielding the dipeptide **5m** (1.0 mg, 2.35 mmol, 85%) as a white foam, whose characterization data were already reported [57].

Hydroxyproline derivatives **5n** (Boc-Hyp-OMe) and **5o** (Cbz-Hyp-OMe) are commercially available.

(2*S*,4*R*)-4-Hydroxy-1-(*N*-phenoxycarbonyl)-L-proline methyl ester (5p). Obtained from commercial *trans*-4-hydroxy-L-proline methyl ester hydrochloride according to the method C, using phenyl chloroformate (5.17 g) as reagent. After purification (n-hexane/ethyl acetate, 40:60), carbamate **5p** (3.28 g, 75%) was isolated as a colorless oil.

$[\alpha]_D$: −69 (*c* 0.32, $CHCl_3$). IR ($CHCl_3$) ν_{max} 2972, 2901, 1734, 1683, 1405 cm^{-1}. ^{1}H NMR (500 MHz, CD_3CN, 70 °C) δ_H 7.39 (t, *J* = 7.9 Hz, 2H), 7.23 (t, *J* = 7.5 Hz, 1H), 7.17–7.04 (br d, *J* = 7.9 Hz, 2H), 4.70–4.56 (m, 1H), 4.52–4.38 (m, 1H), 3.73 (s, 3H), 3.72–3.44 (m, 2H), 3.13 (s, 1H), 2.39–2.24 (m, 1H), 2.21–2.07 (m, 1H). ^{13}C RMN (125.7 MHz, CD_3CN, 70 °C) rotamer mixture δ_C 152.8 (C), 130.5 (C + 2 × CH), 126.5 (CH), 122.8 (2 × CH), 70.8/70.0 (CH), 59.6 (CH), 56.3 (CH_2), 53.1 (CH_3), 40.3/39.4 (CH_2). The signals of carbonyl groups were not clearly observed. HRMS (ESI) calculated for $C_{13}H_{15}NO_5Na$ $[M + Na]^+$ 288.0848, found 288.0848. Anal. Calcd for $C_{13}H_{15}NO_5$: C, 58.86; H, 5.70; N, 5.28. Found: C, 58.82; H, 5.78; N, 5.53.

Compound **5a**, ^{1}H and ^{13}C NMR at 26 °C in $CDCl_3$

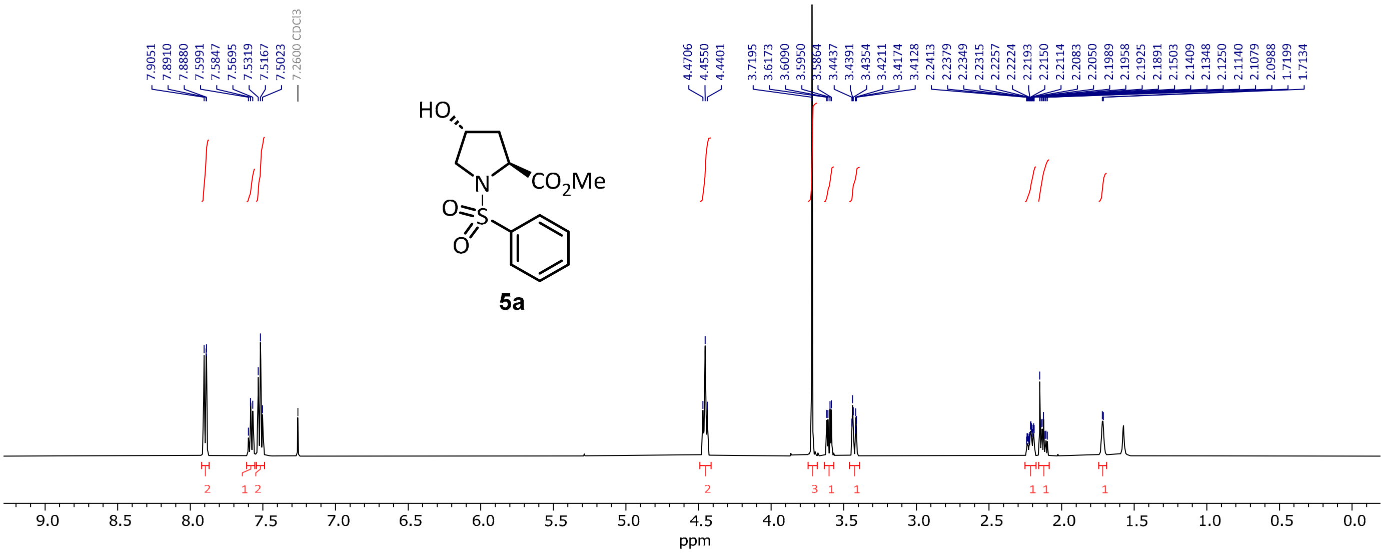

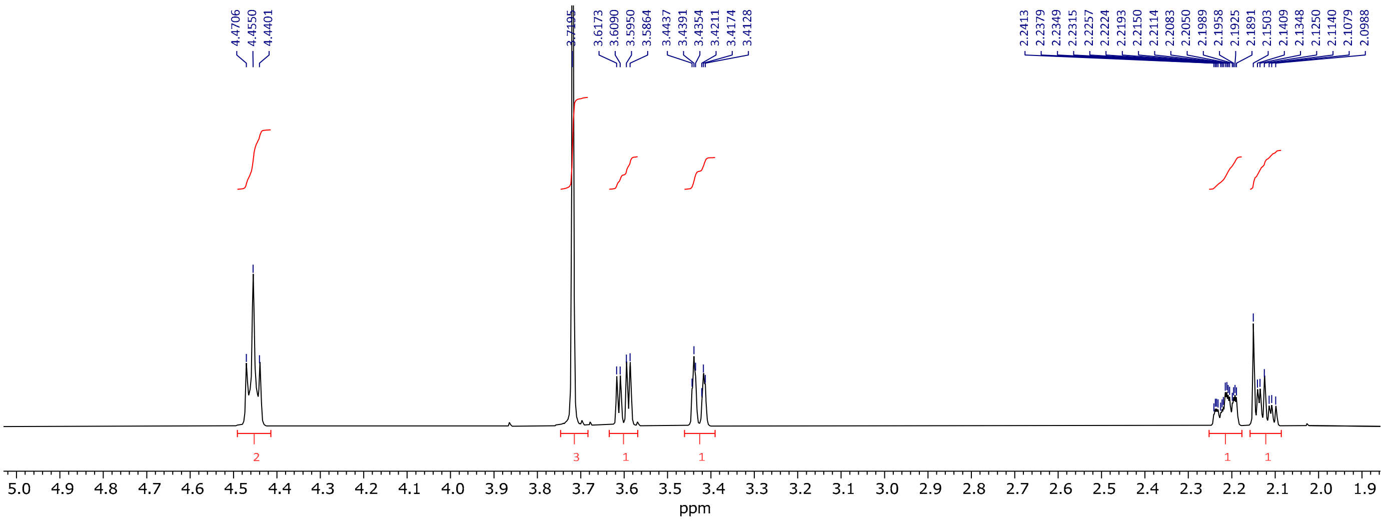

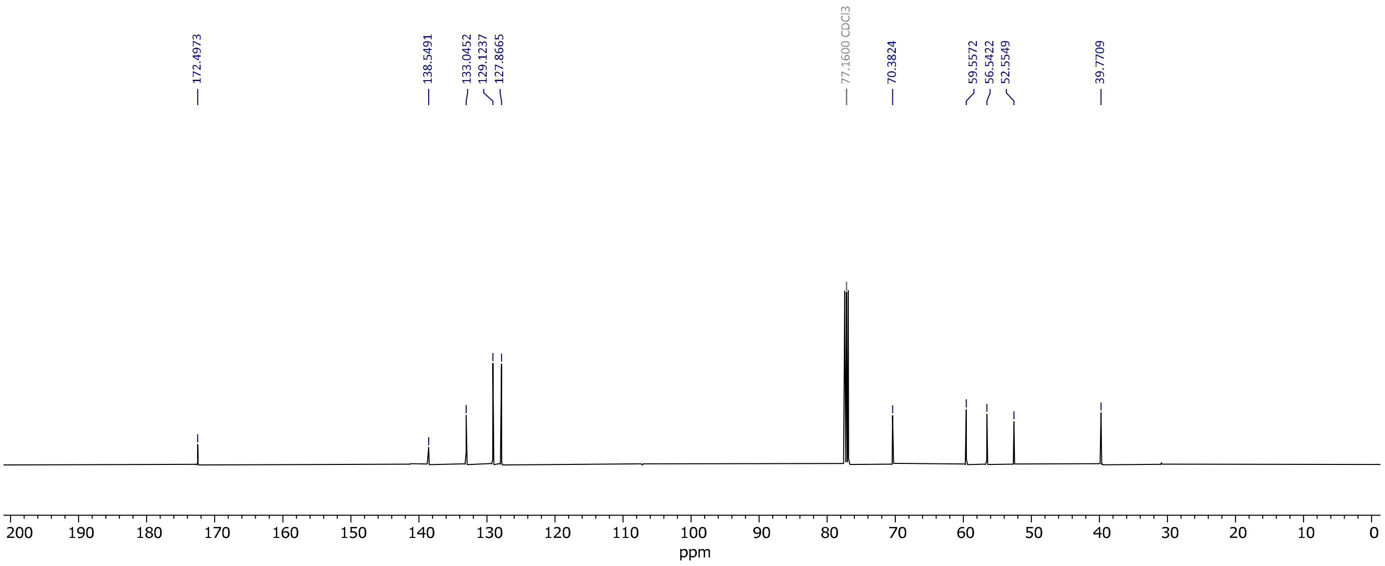

Compound **5b**, ^{1}H and ^{13}C NMR at 26 °C in $CDCl_3$

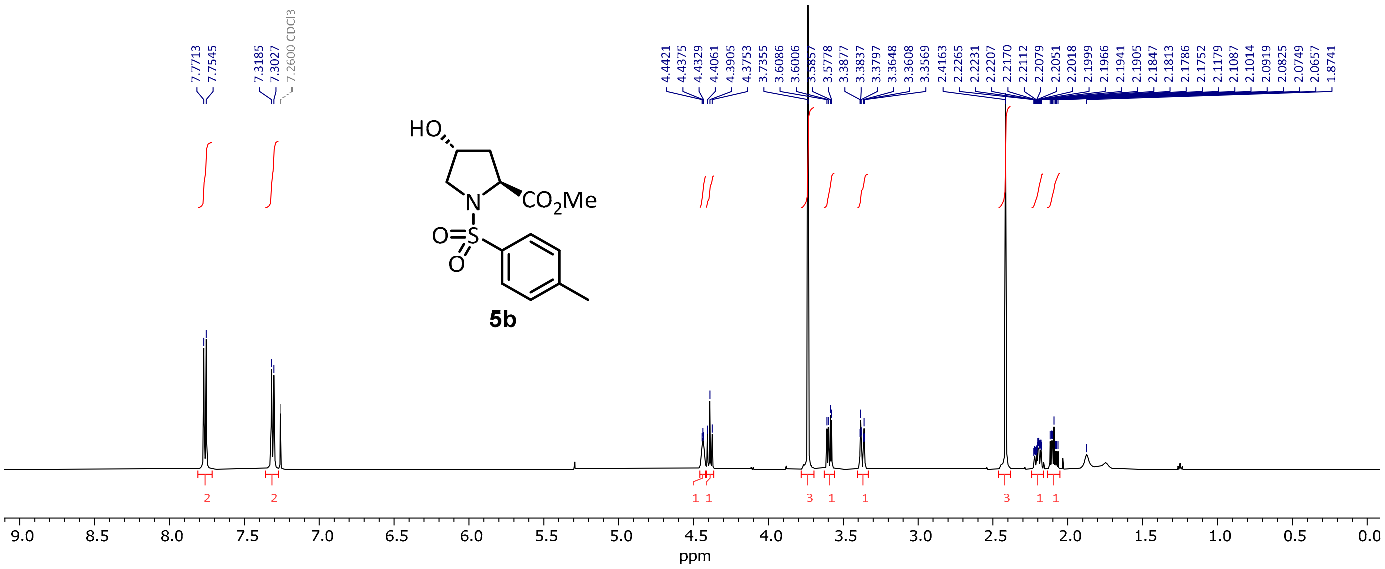

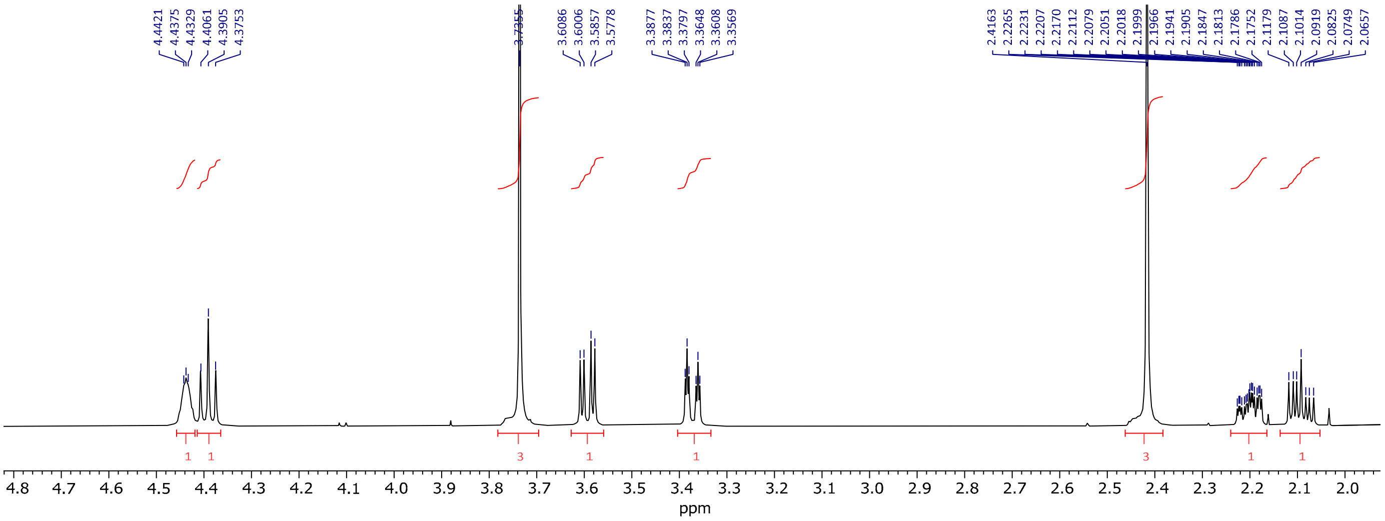

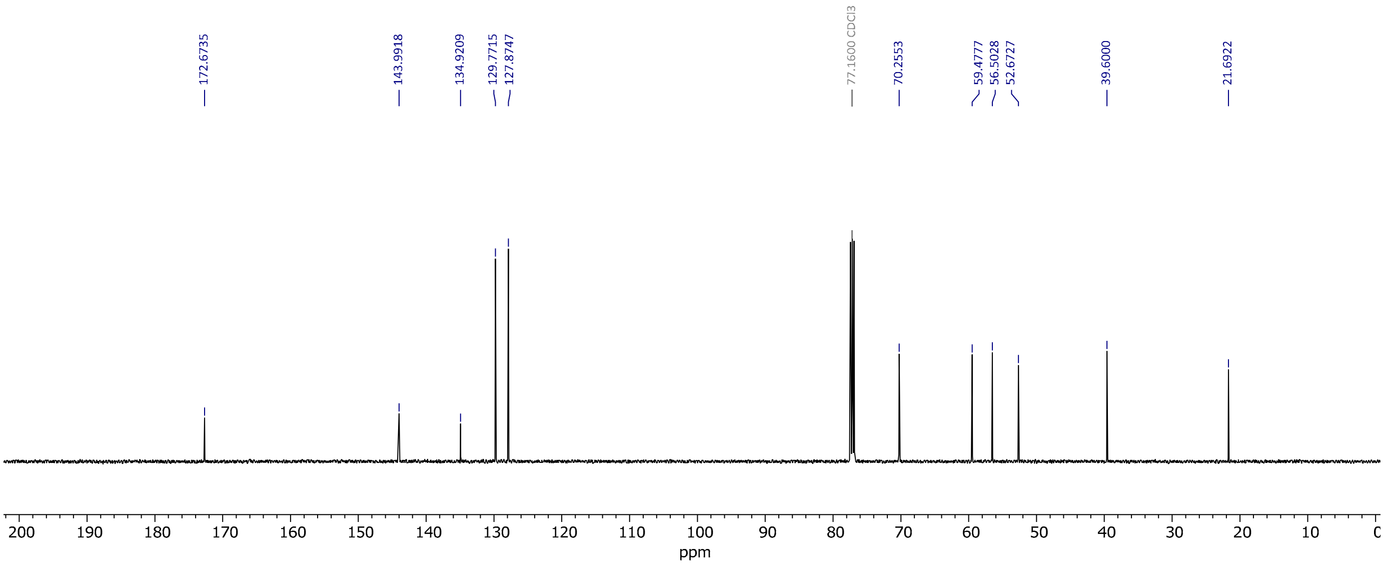

Compound **5c**, ¹H and ¹³C NMR at 26 °C in CDCl₃

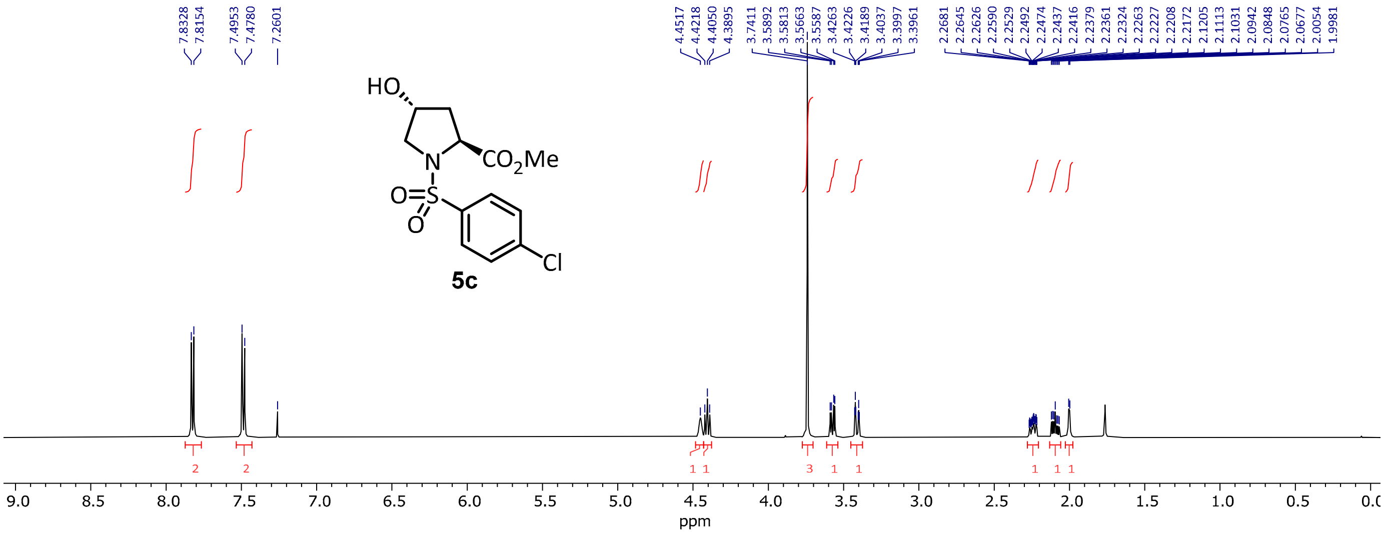

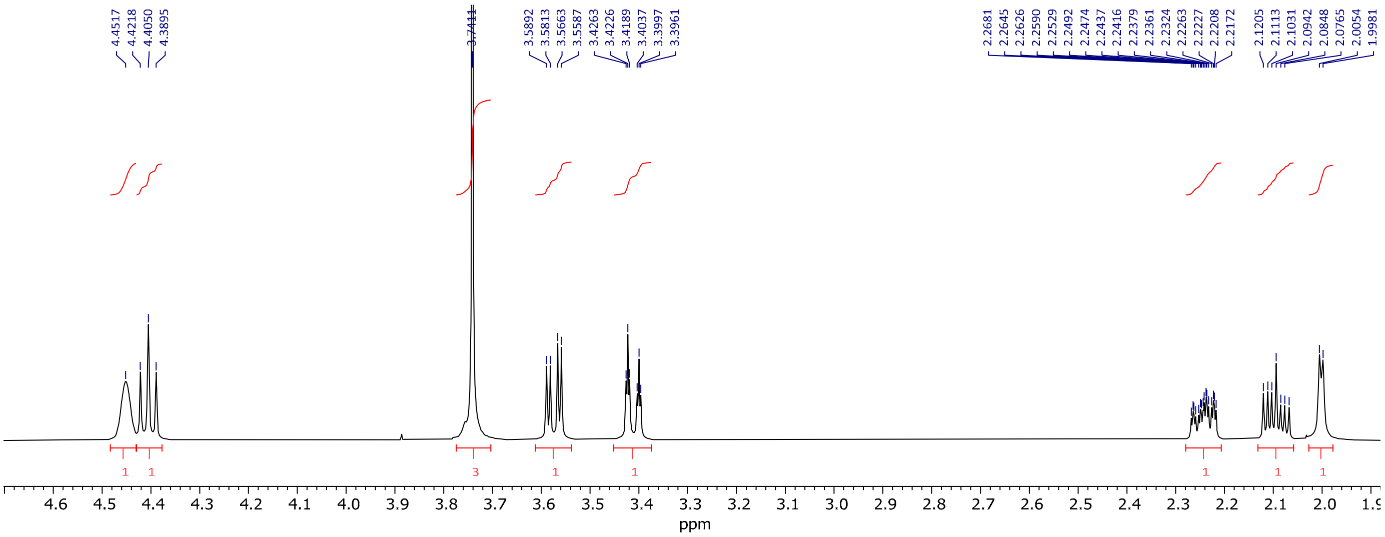

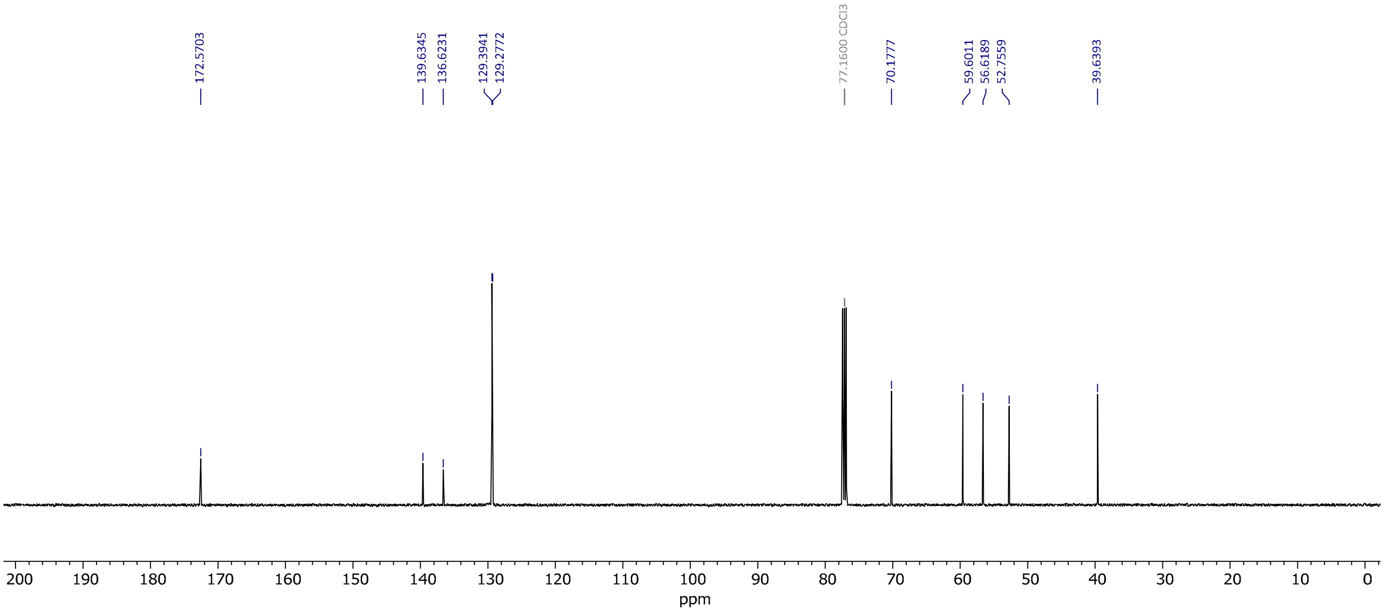

Compound **5d**, ^{1}H and ^{13}C NMR at 26 °C in $CDCl_3$

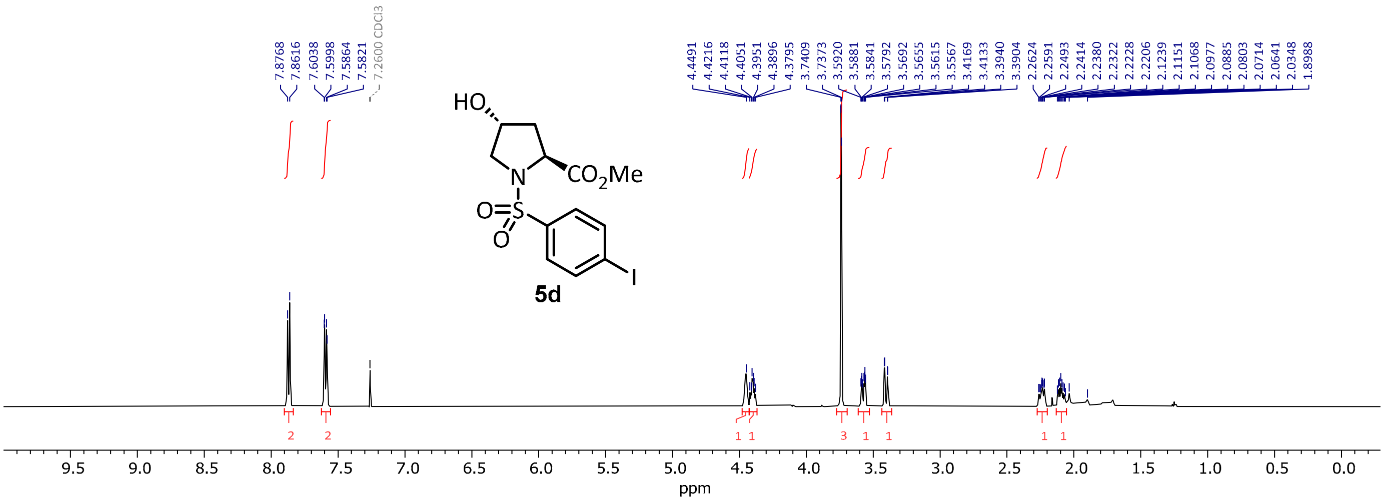

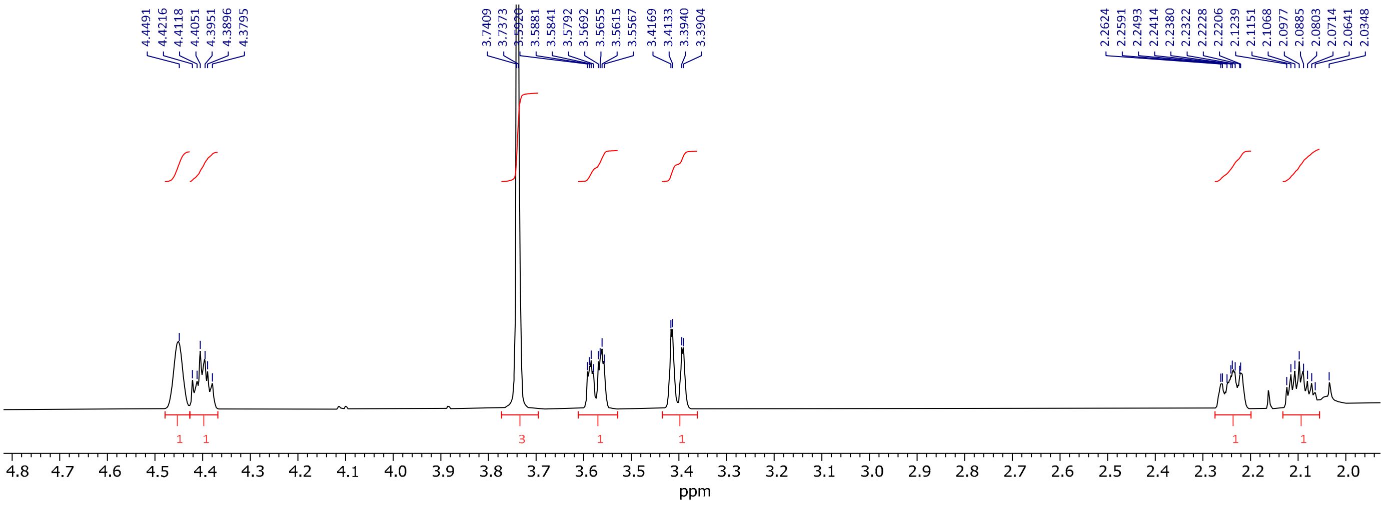

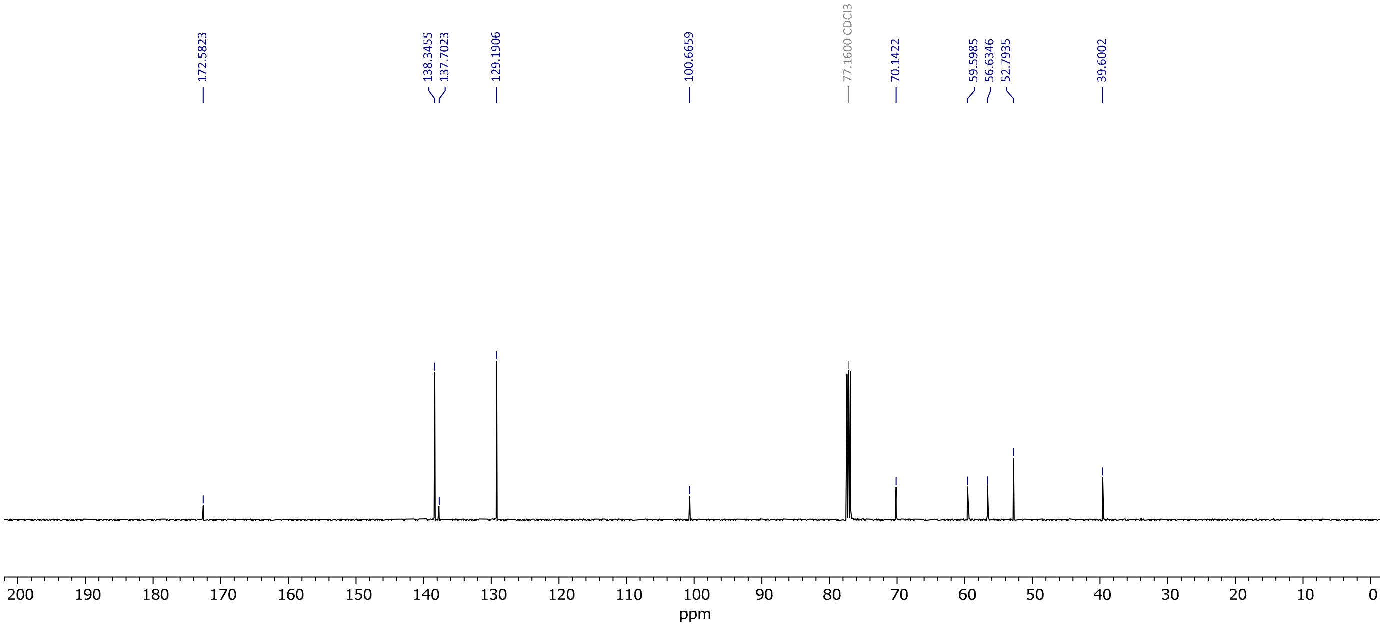

Compound **5e**, ^{1}H and ^{13}C NMR at 26 °C in $(CD_3)_2CO$

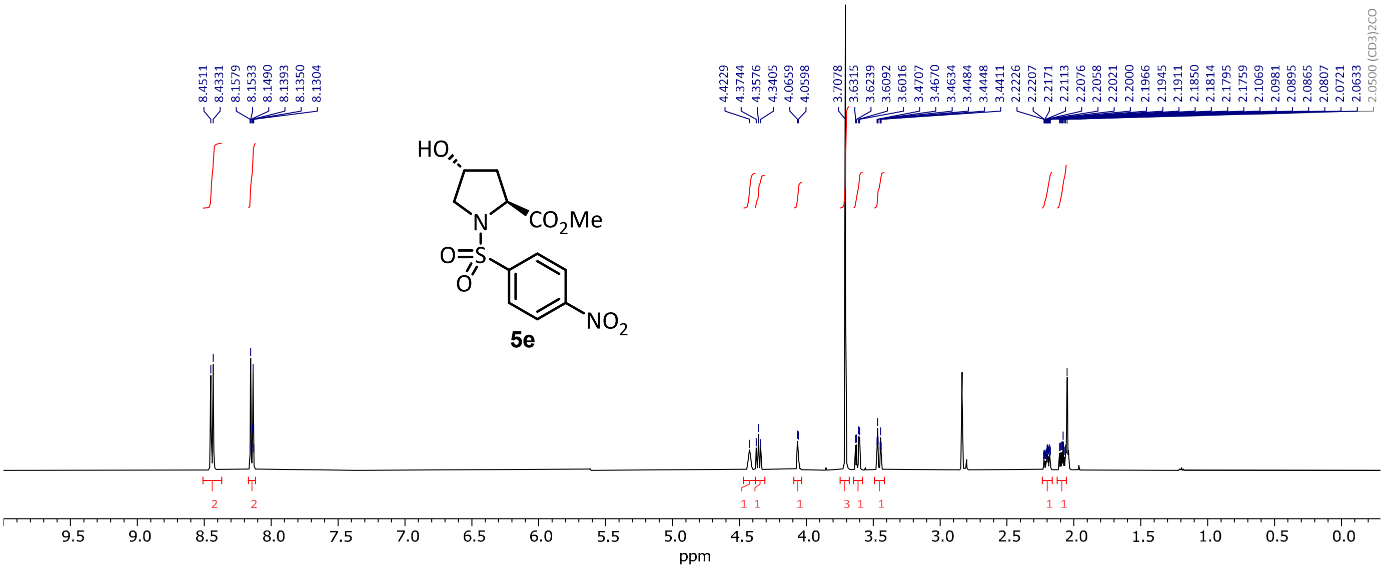

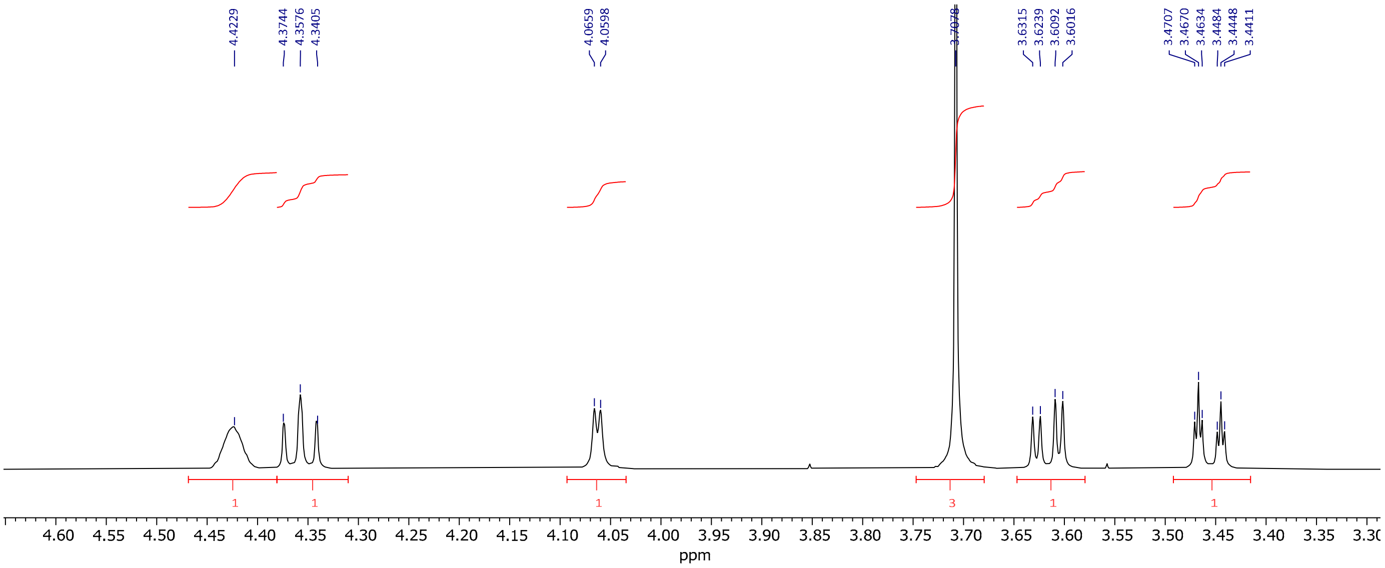

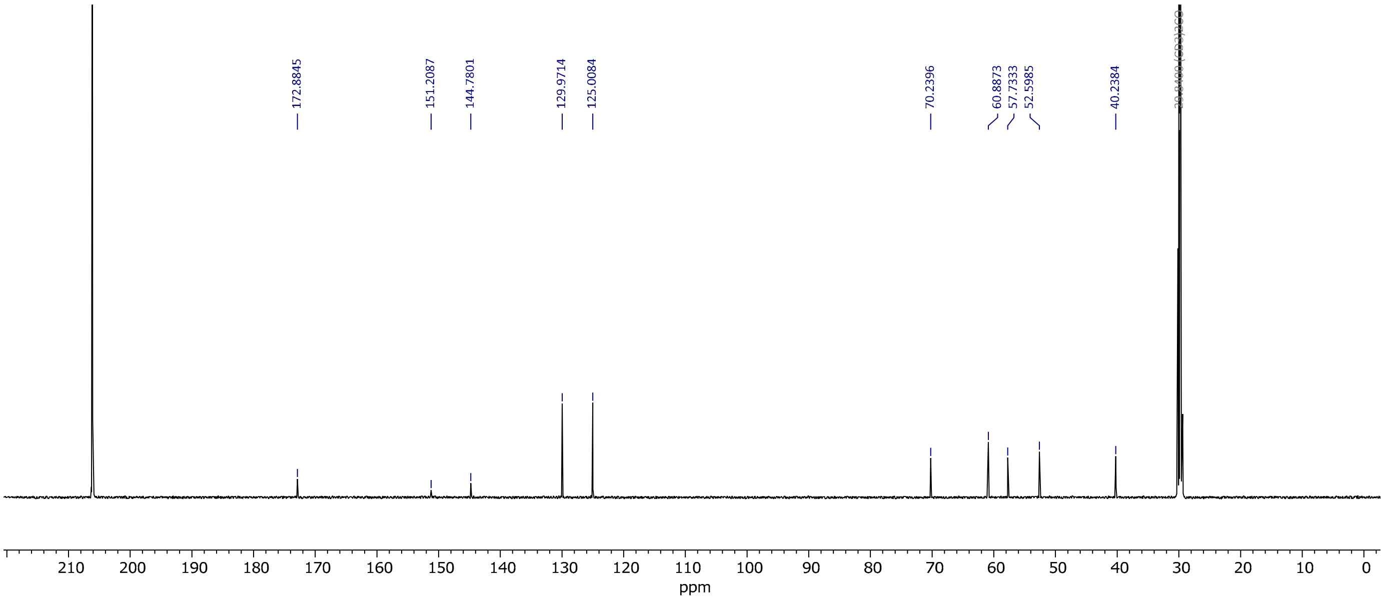

Compound **5g**, ^{1}H and ^{13}C NMR at 26 °C in $CDCl_3$

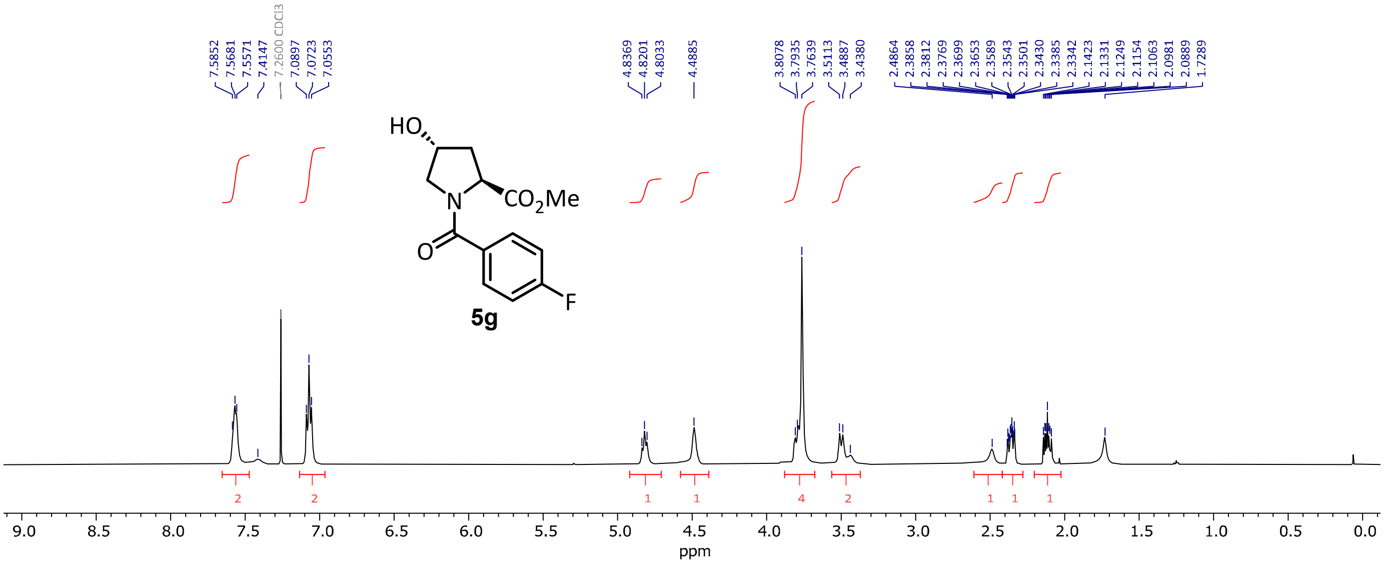

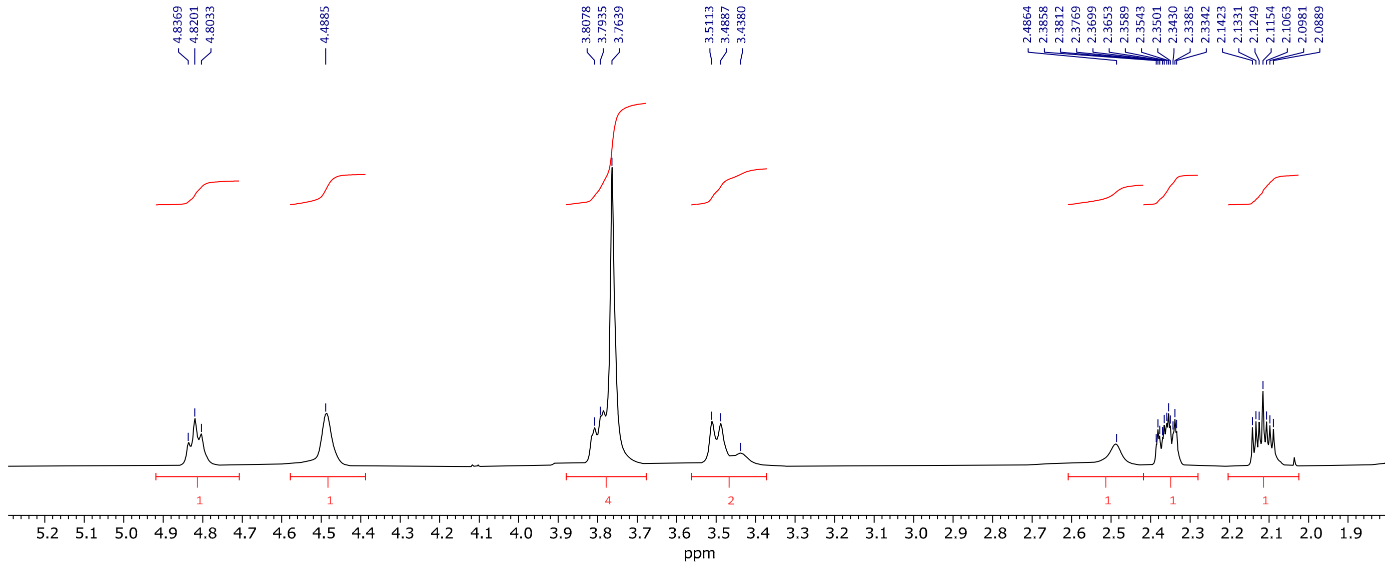

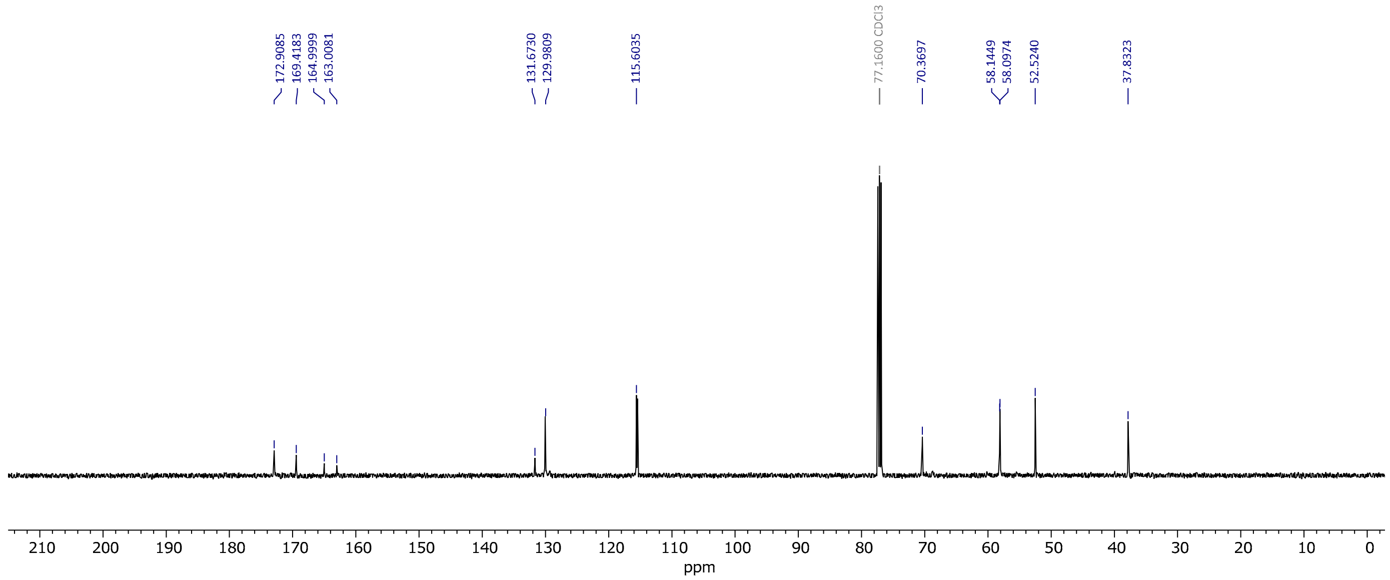

Compound **5h**, ^{1}H and ^{13}C NMR at 26 °C in $CDCl_3$

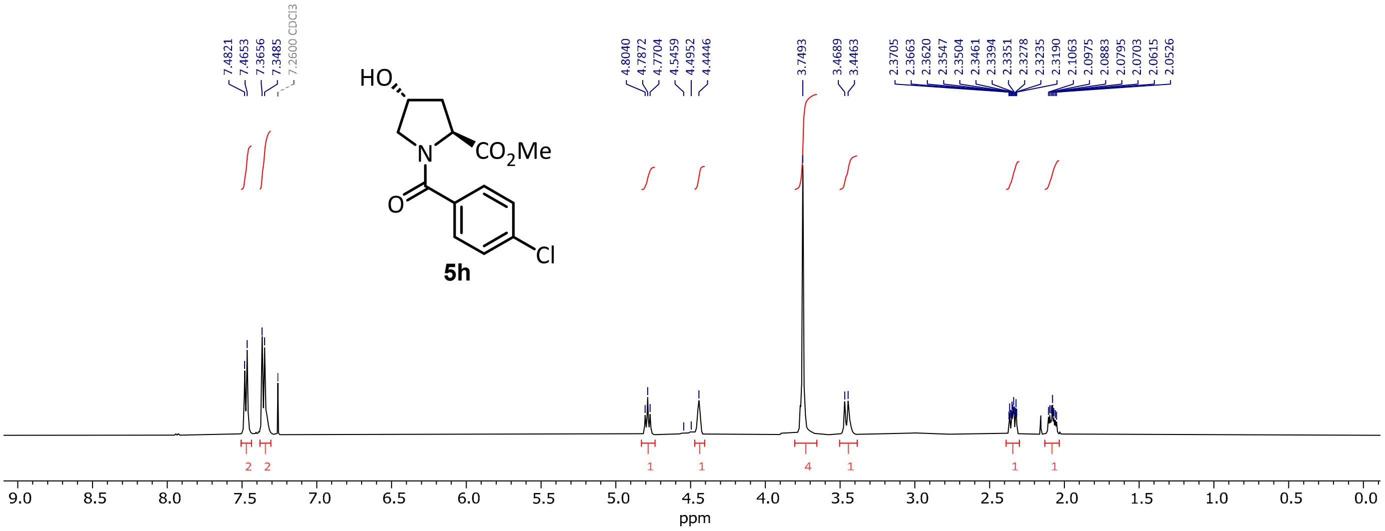

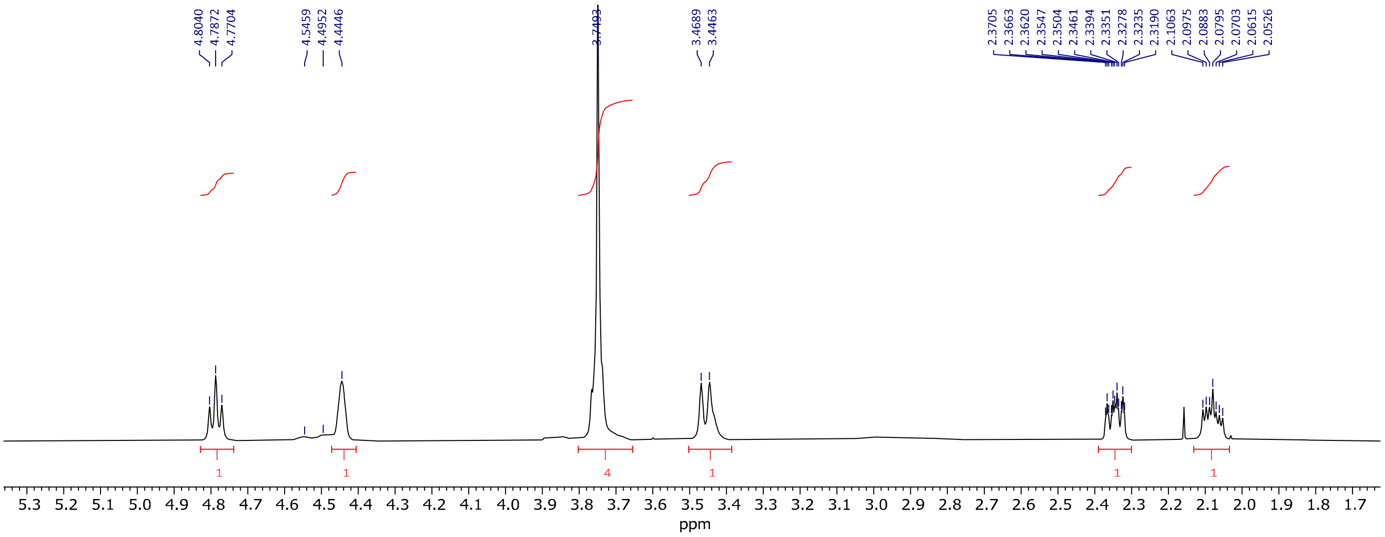

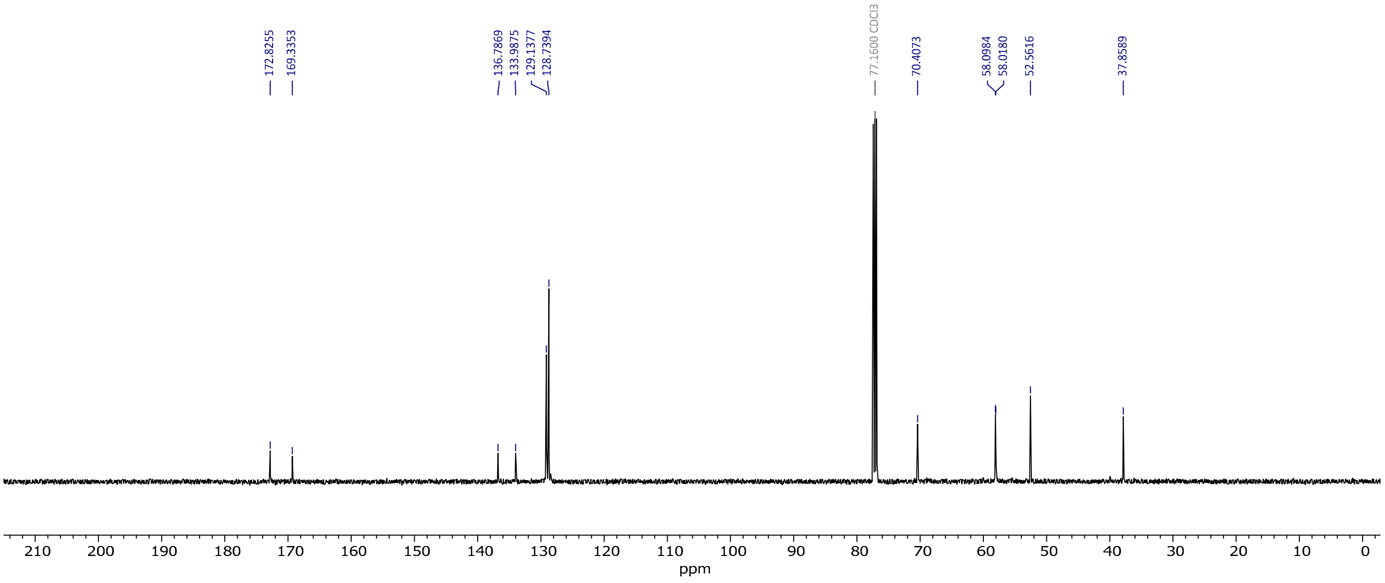

Compound **5i**, ^{1}H and ^{13}C NMR at 26 °C in $CDCl_3$

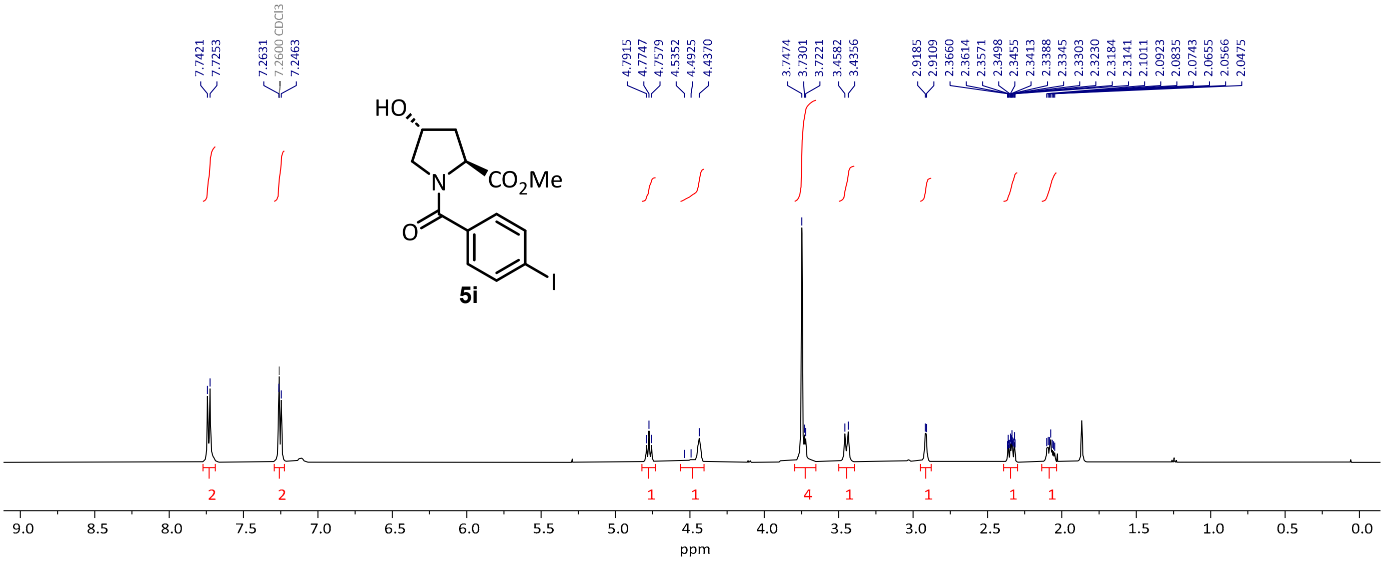

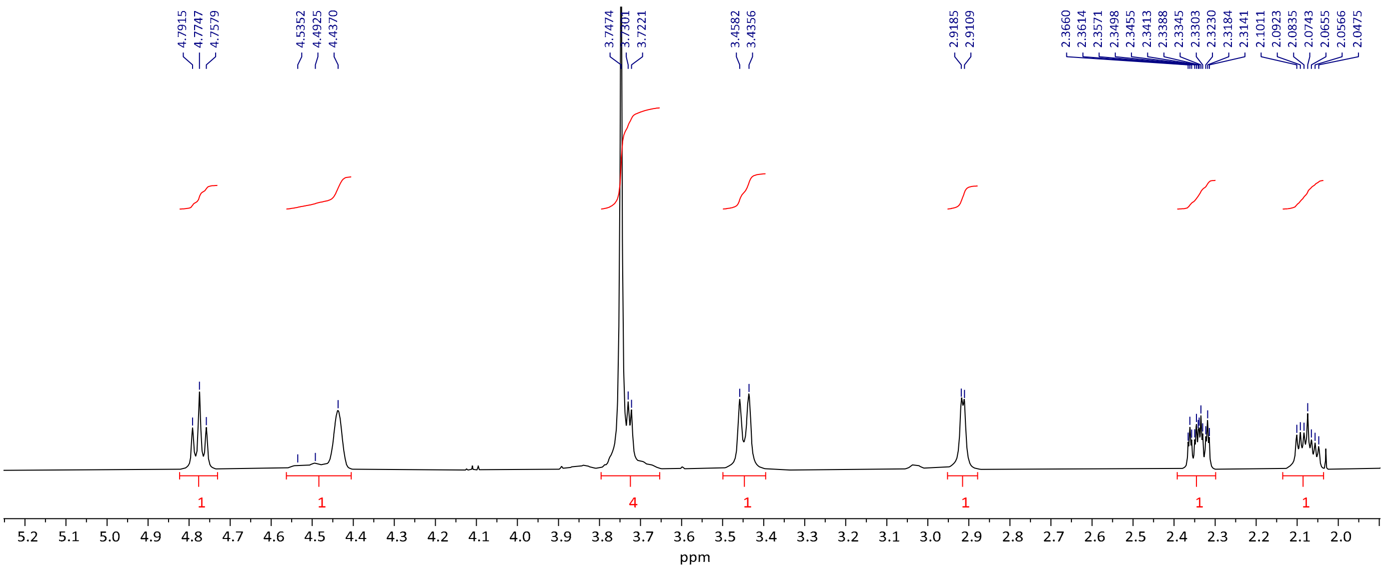

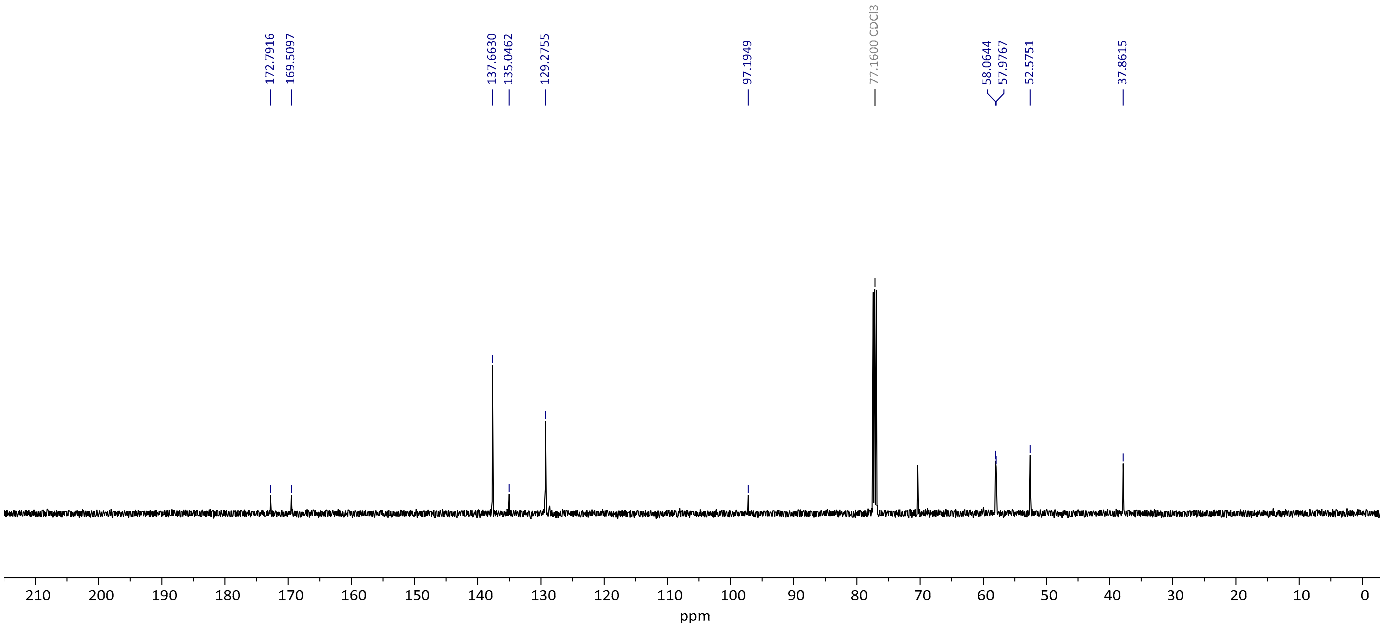

Compound **5j**, ^{1}H and ^{13}C NMR at 26 °C in $CDCl_3$

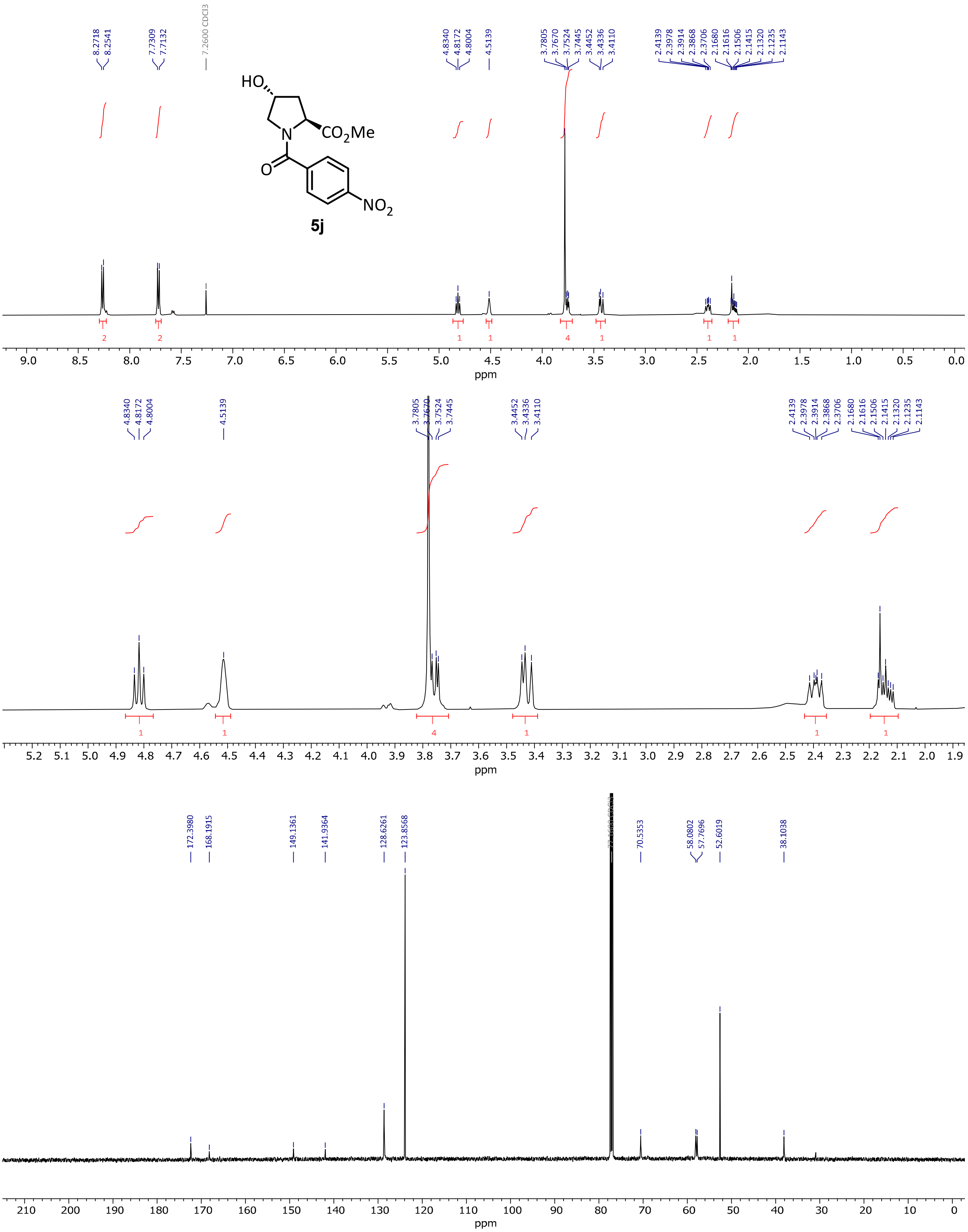

Compound **5k**, ^{1}H and ^{13}C NMR at 26 °C in $CDCl_3$

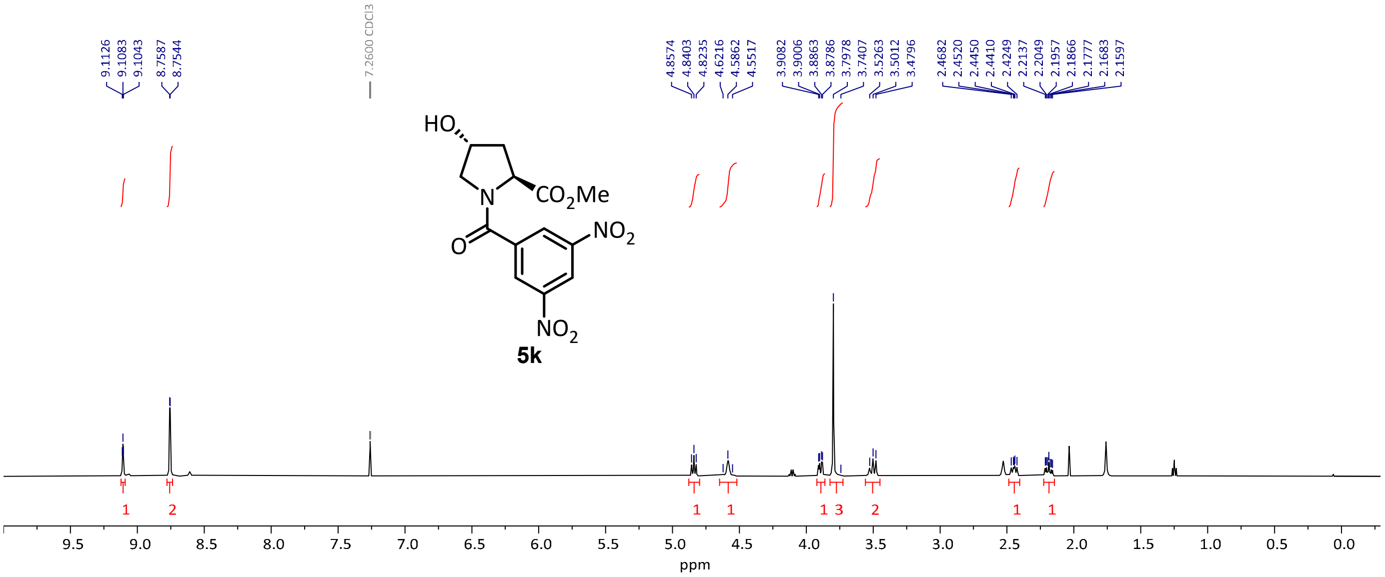

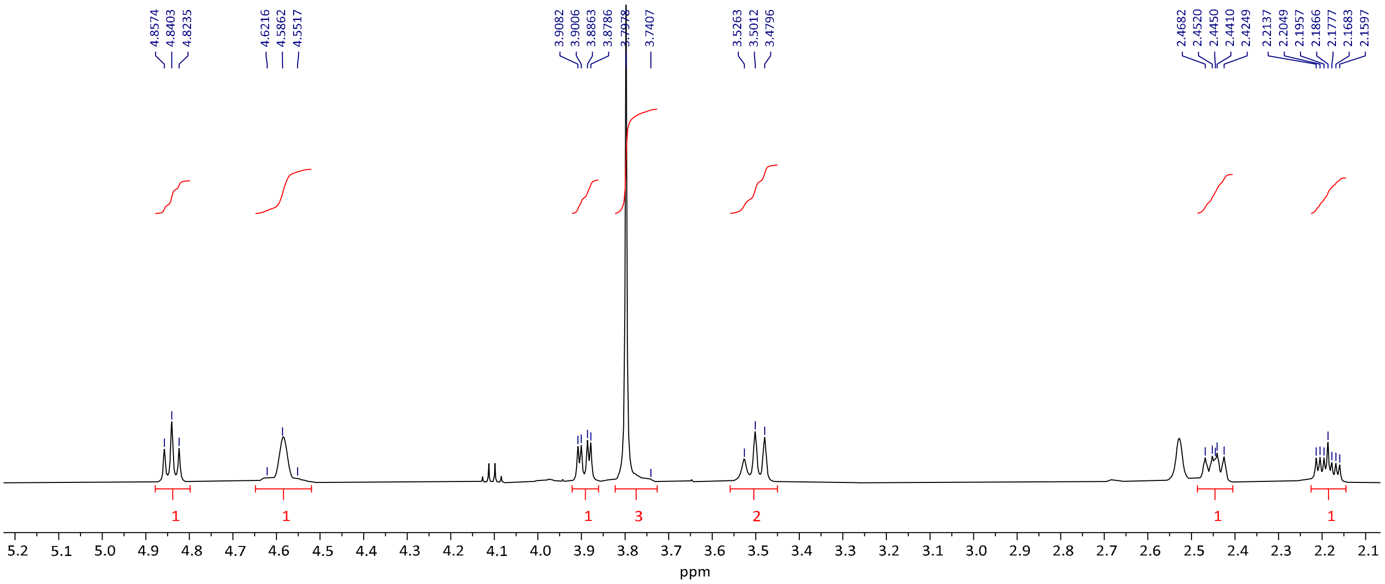

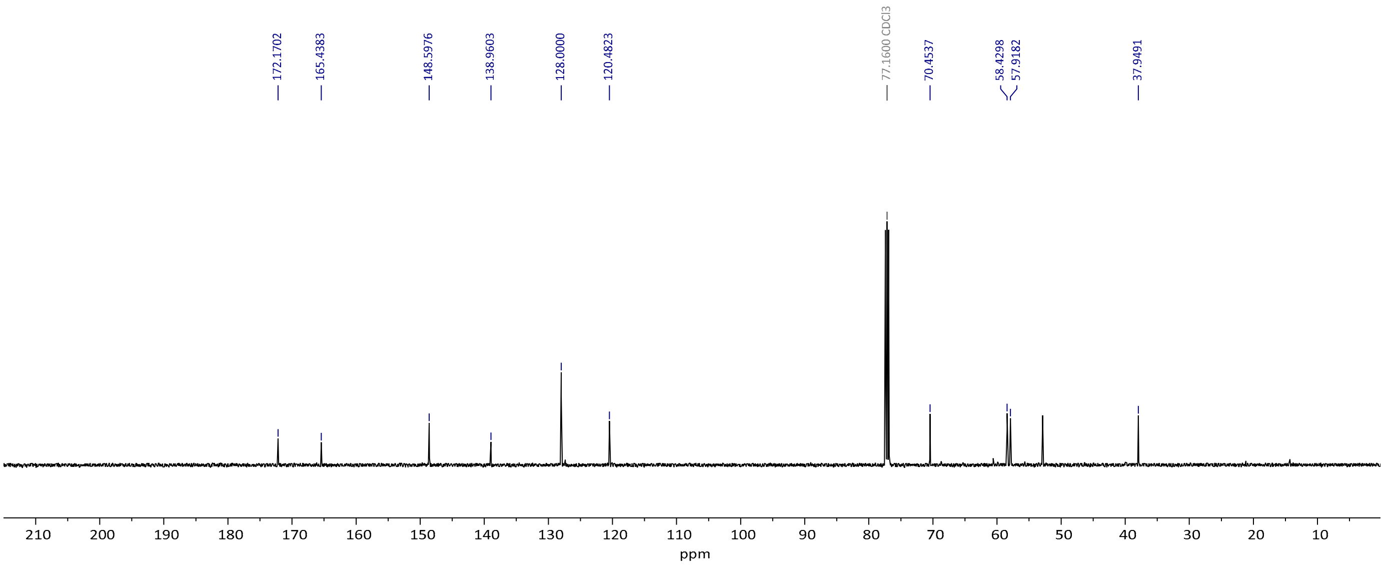

Compound **5p**, ^{1}H and ^{13}C NMR at 70 °C in CD3CN

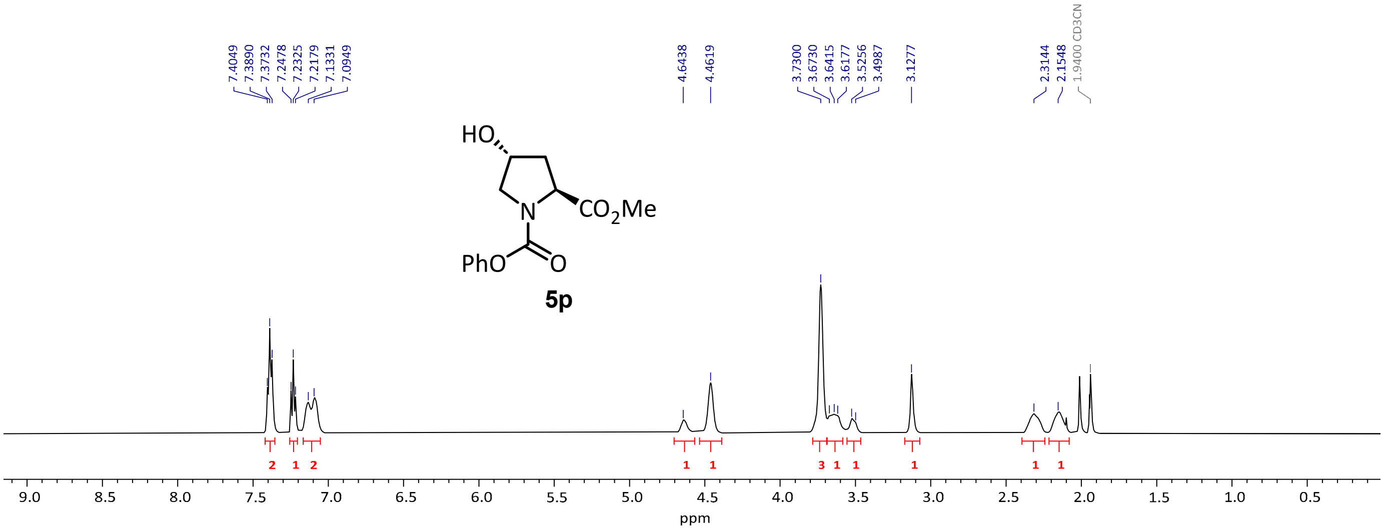

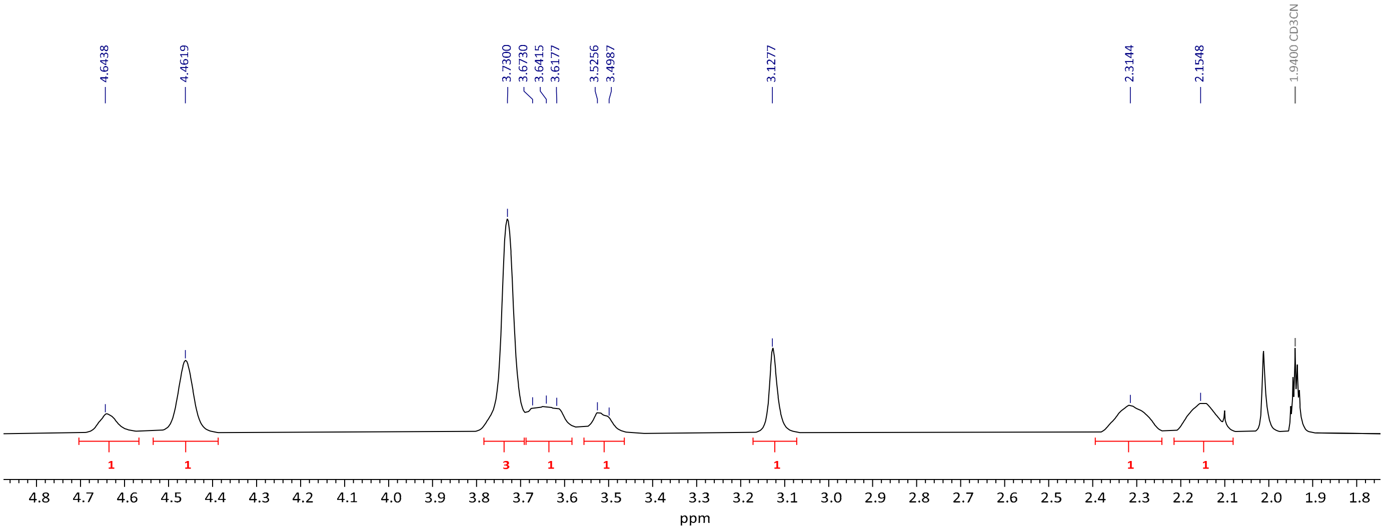

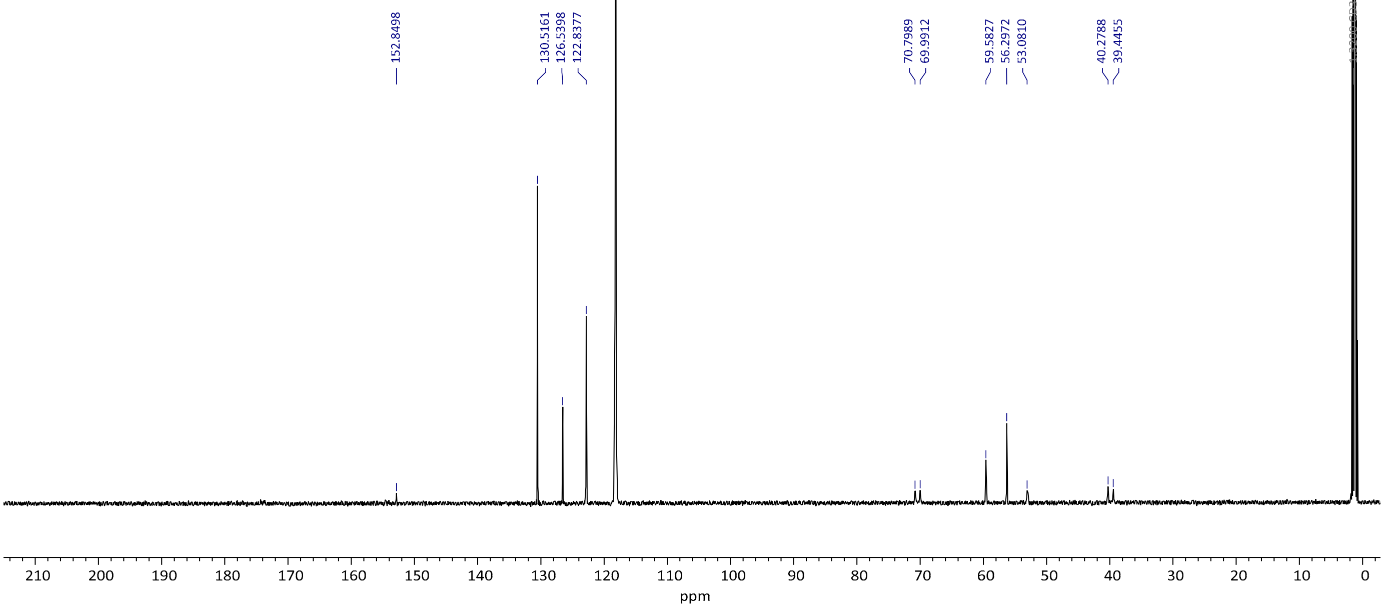

Compound **6a**, ^{1}H and ^{13}C NMR at 26 °C in $CDCl_3$

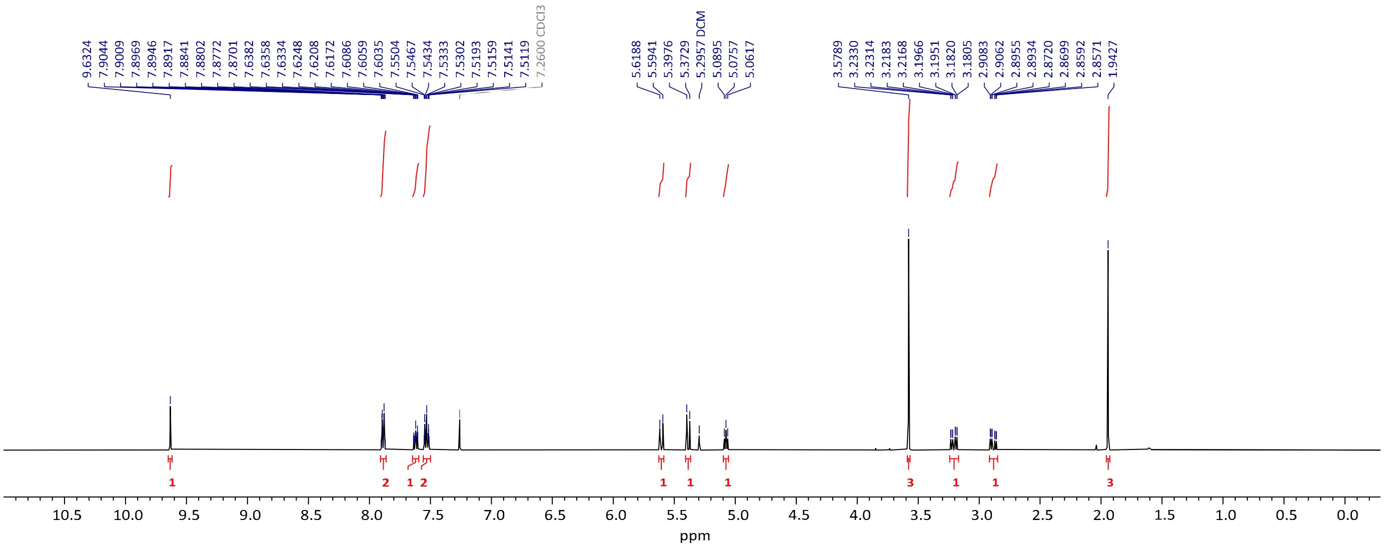

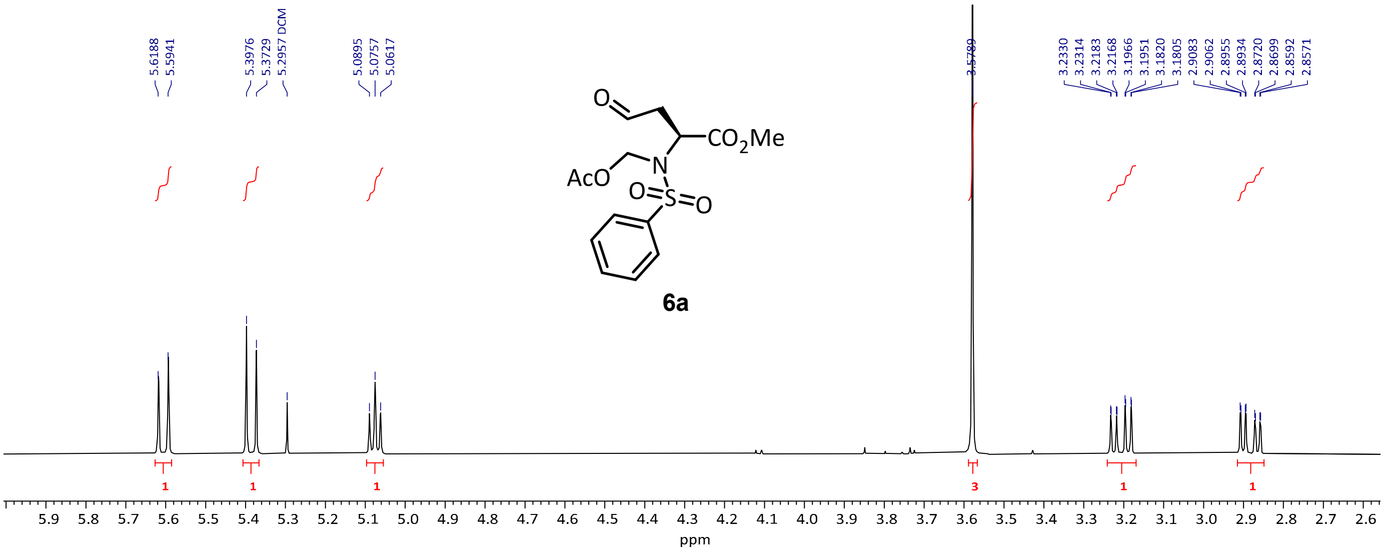

6a

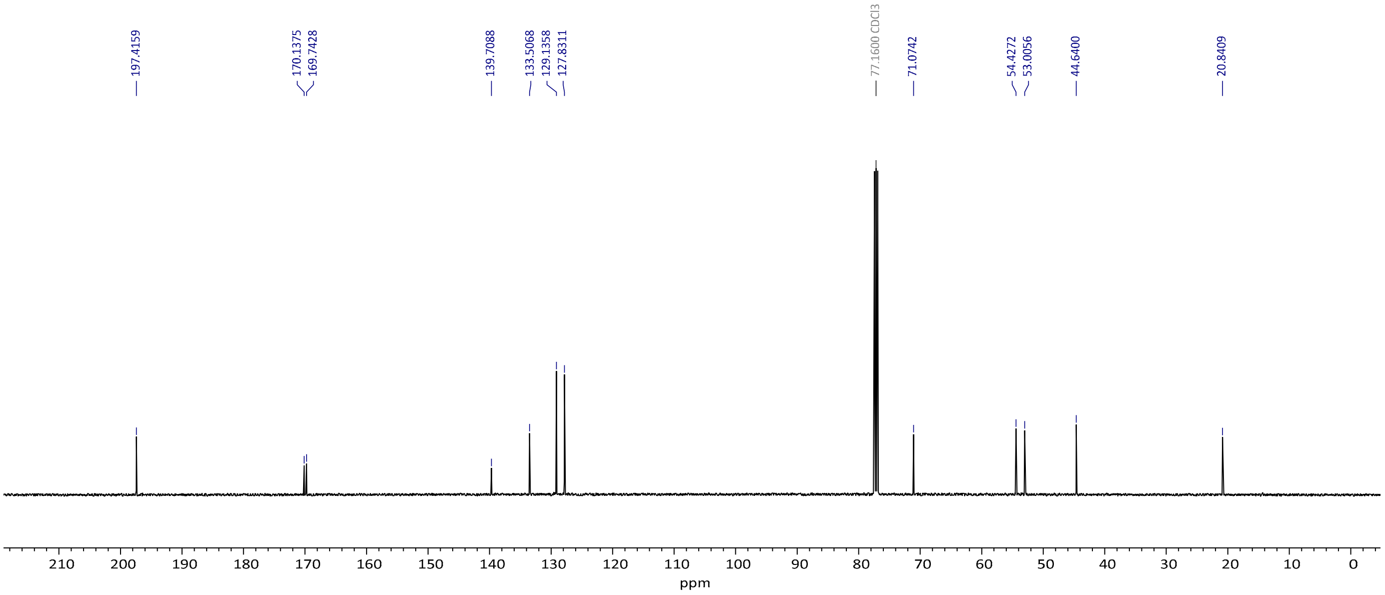

Compound **6b**, ^{1}H and ^{13}C NMR at 26 °C in $CDCl_3$

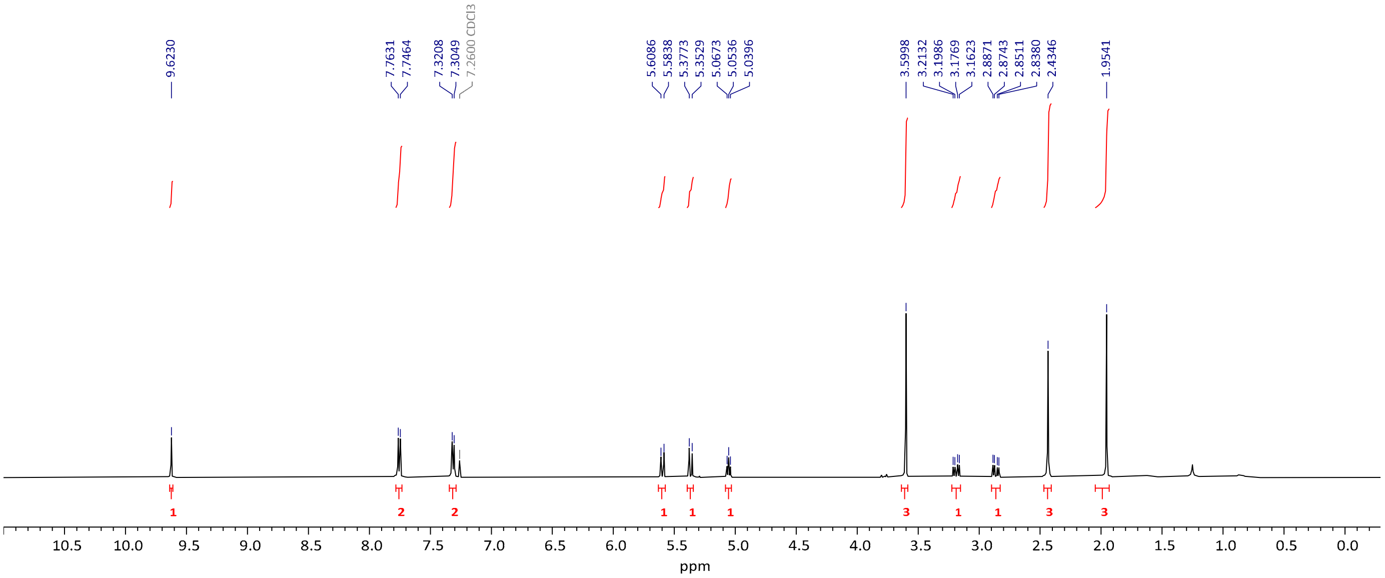

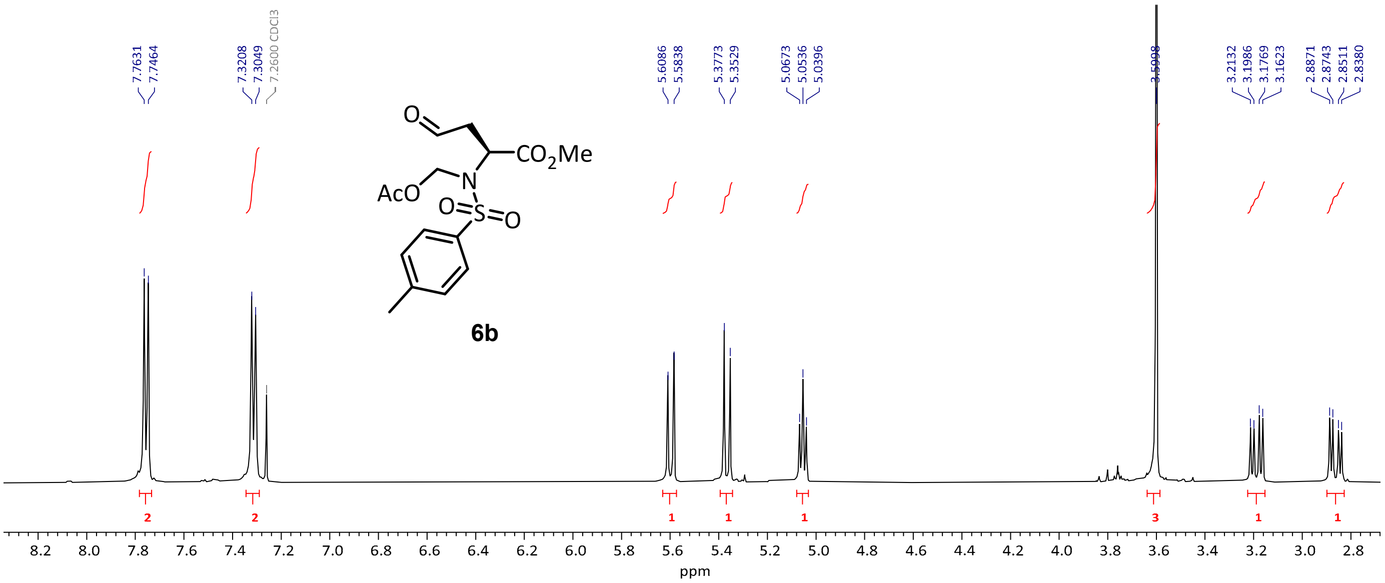

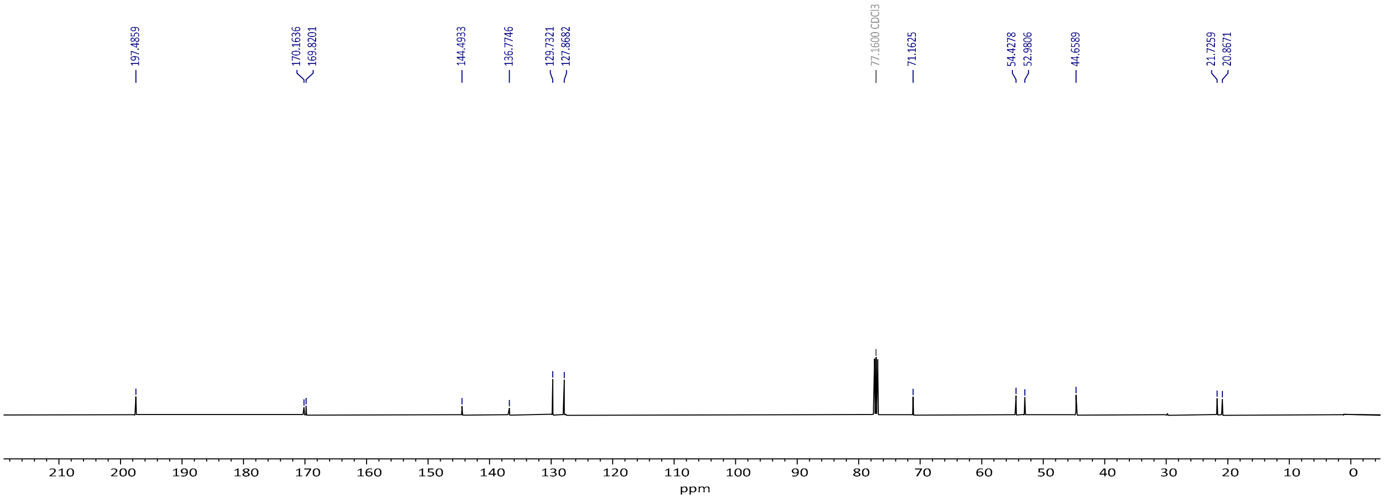

Compound **6c**, ^{1}H and ^{13}C NMR at 26 °C in $CDCl_3$

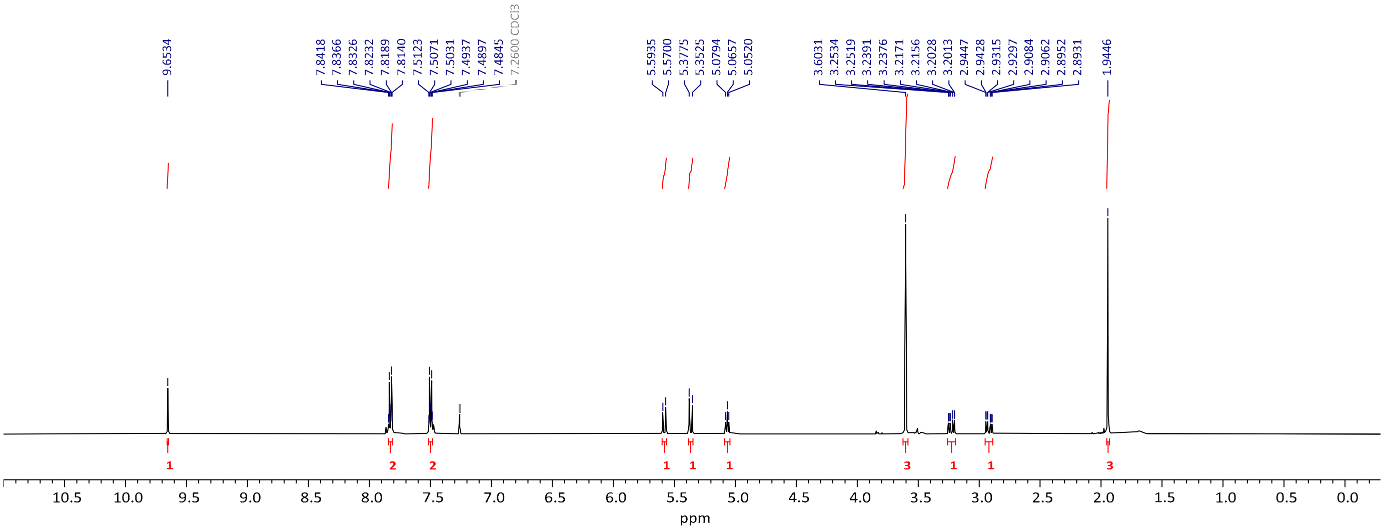

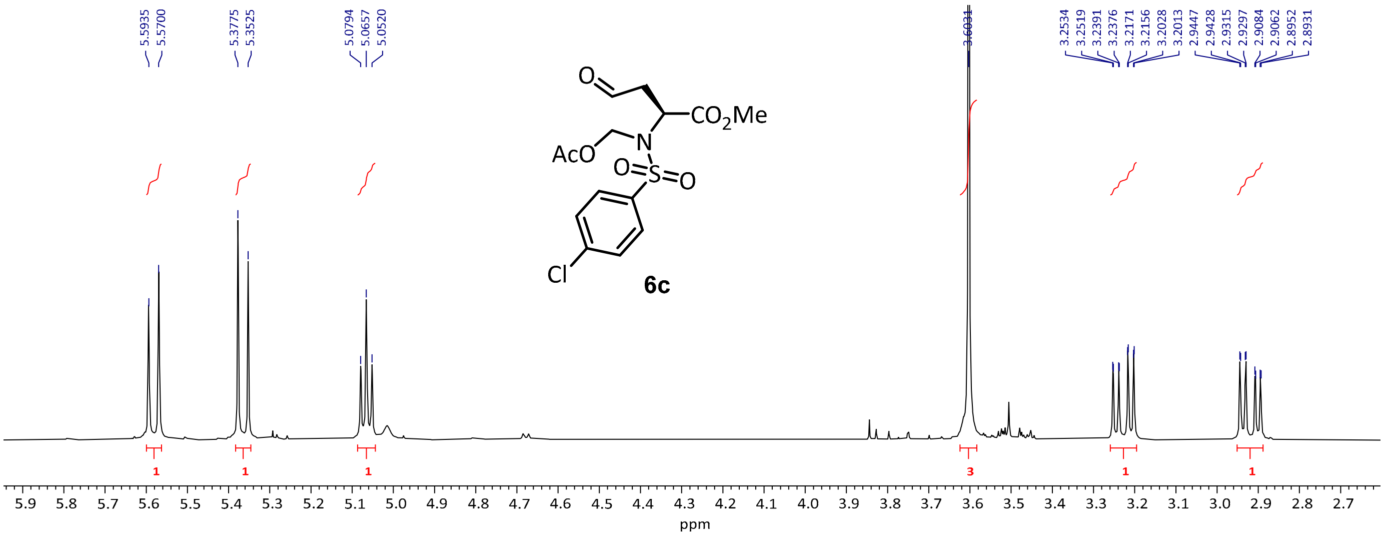

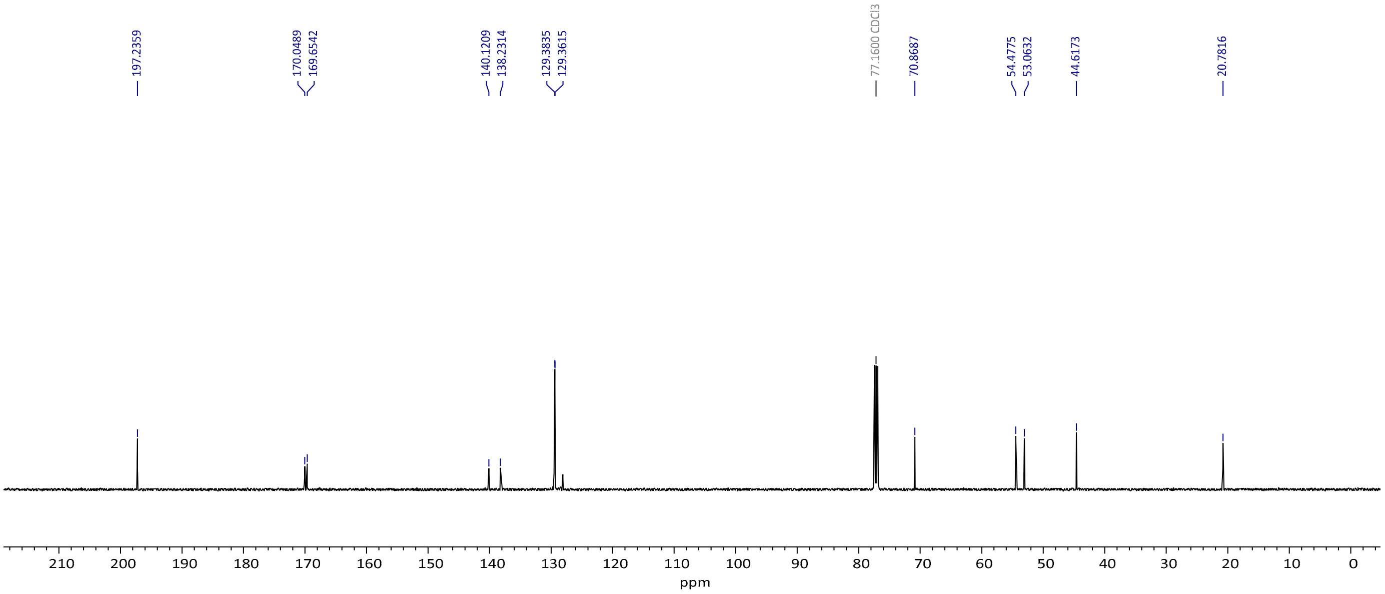

Compound **6d**, ^{1}H and ^{13}C NMR at 26 °C in $CDCl_3$

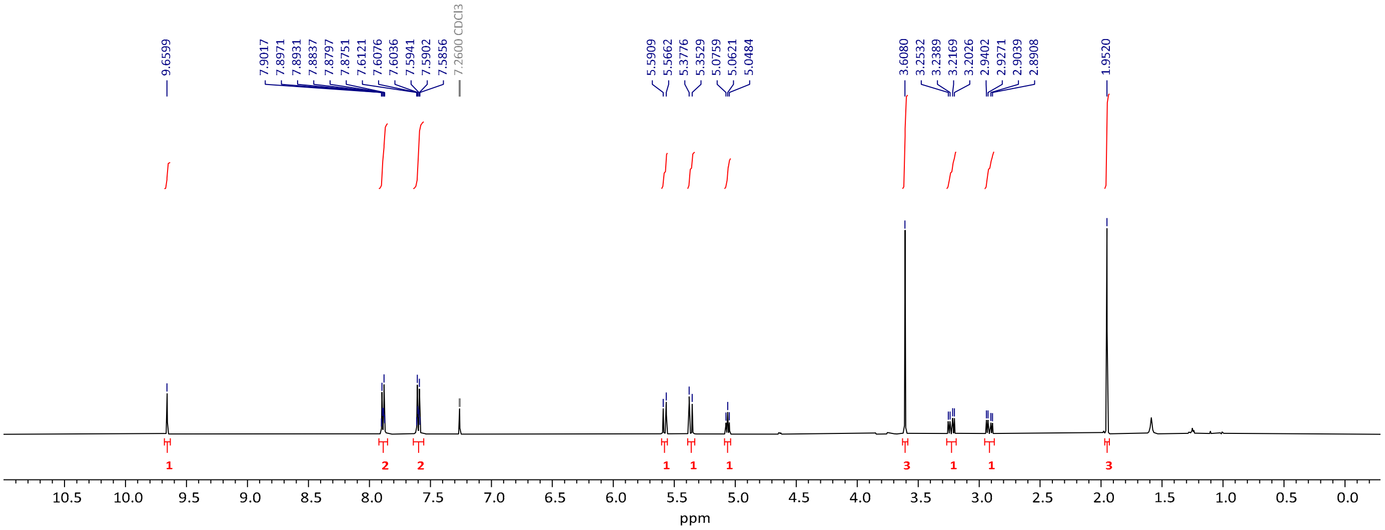

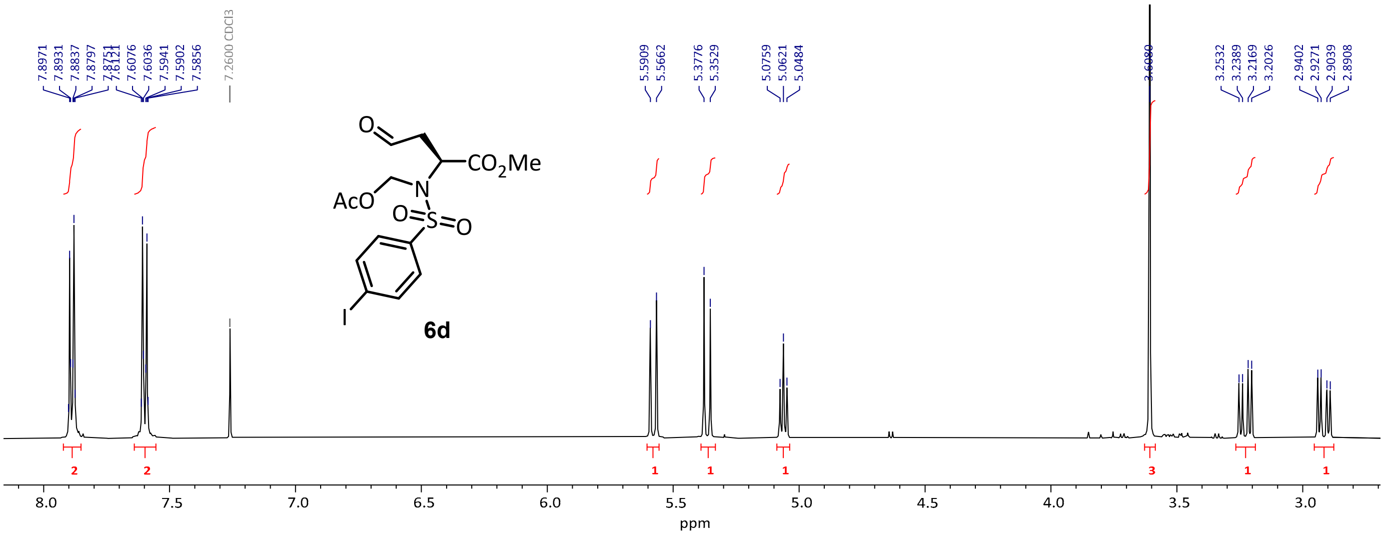

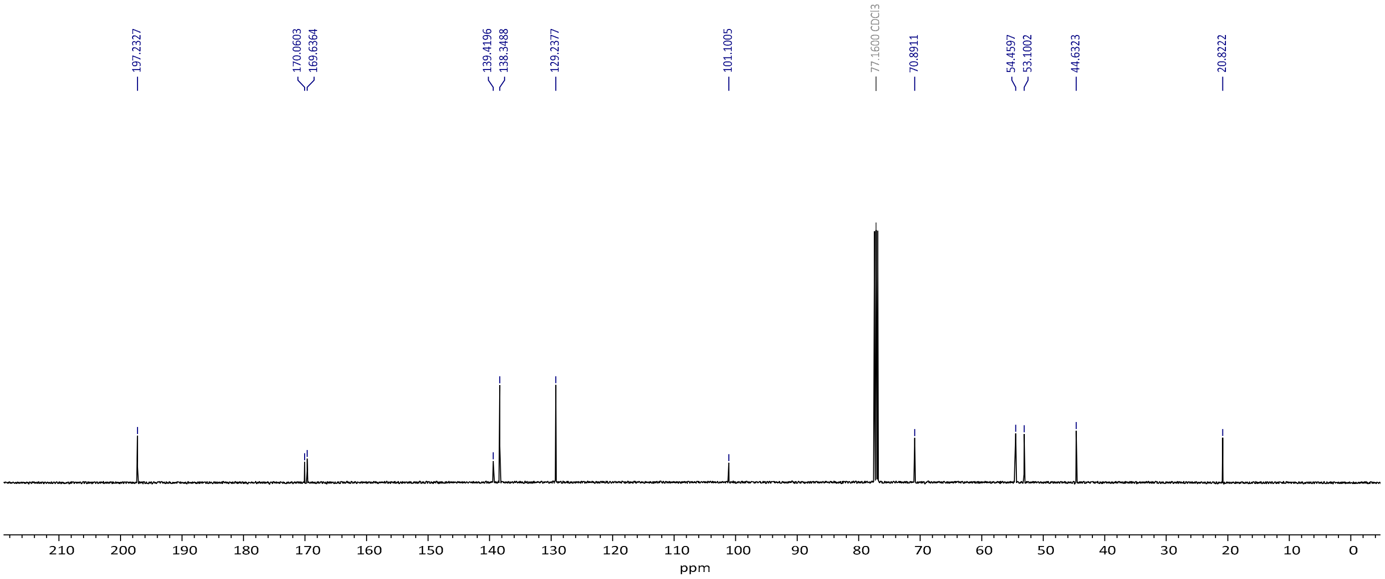

Compound **6e**, ^{1}H and ^{13}C NMR at 26 °C in $CDCl_3$

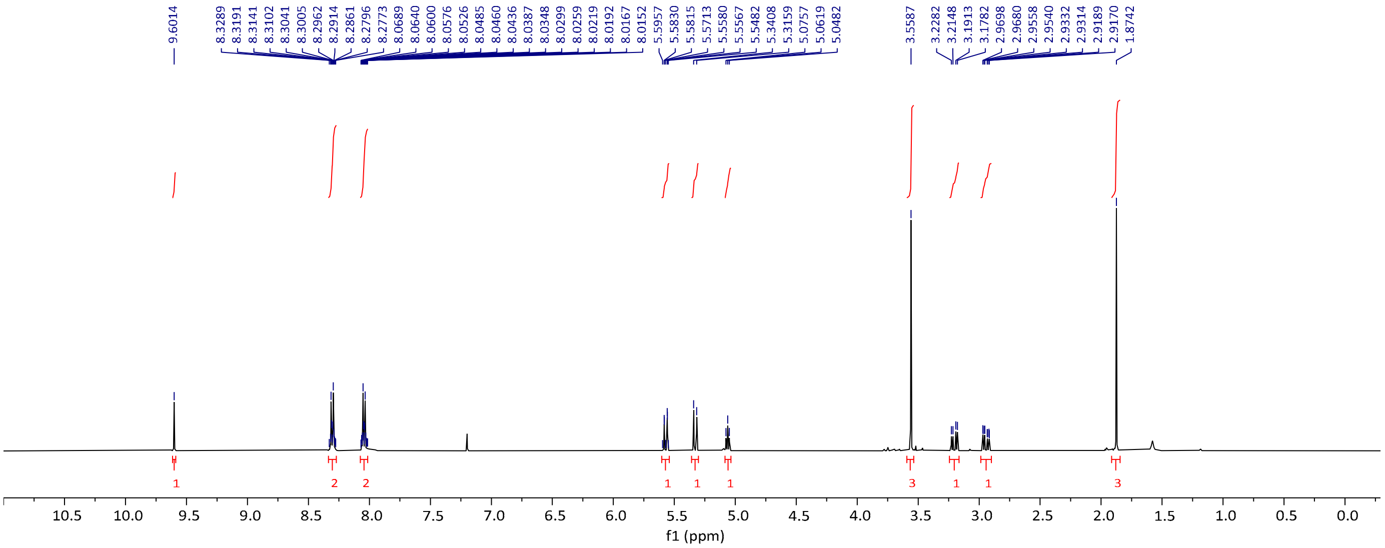

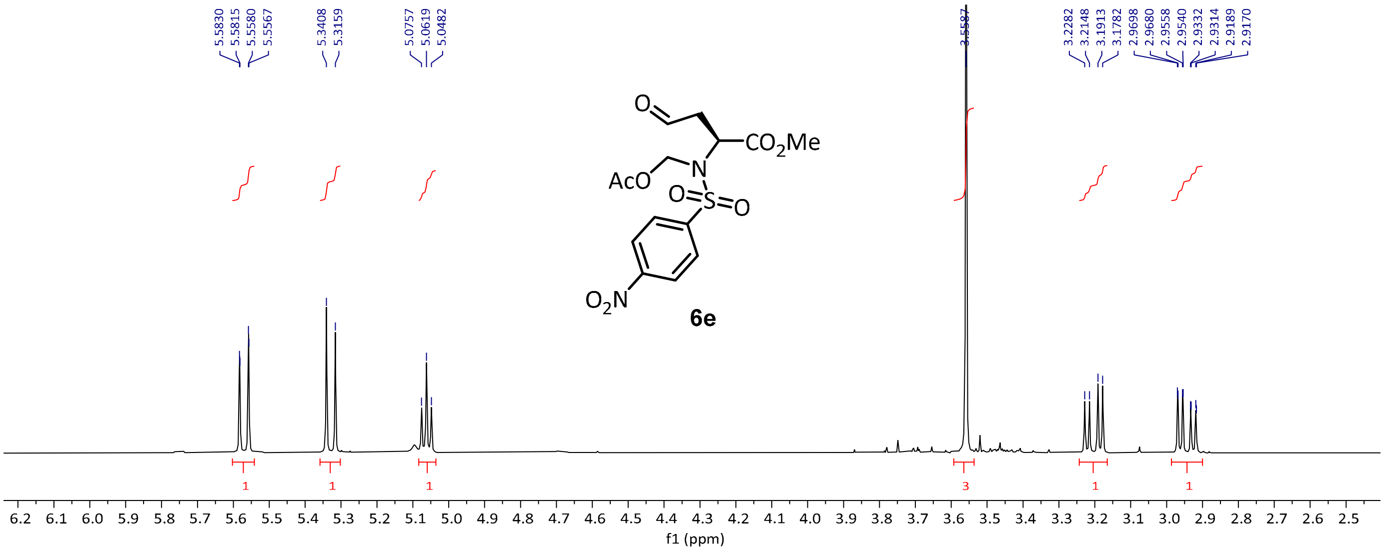

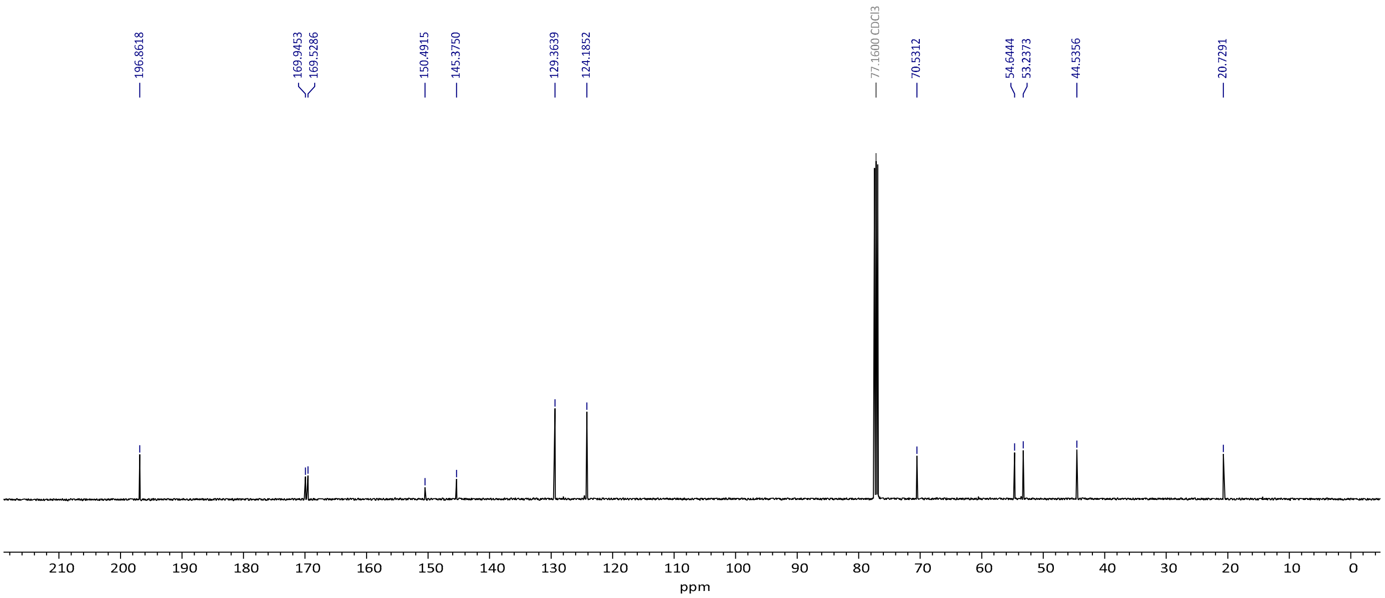

Compound **6g**, ^{1}H and ^{13}C NMR at 55 °C in $CDCl_3$

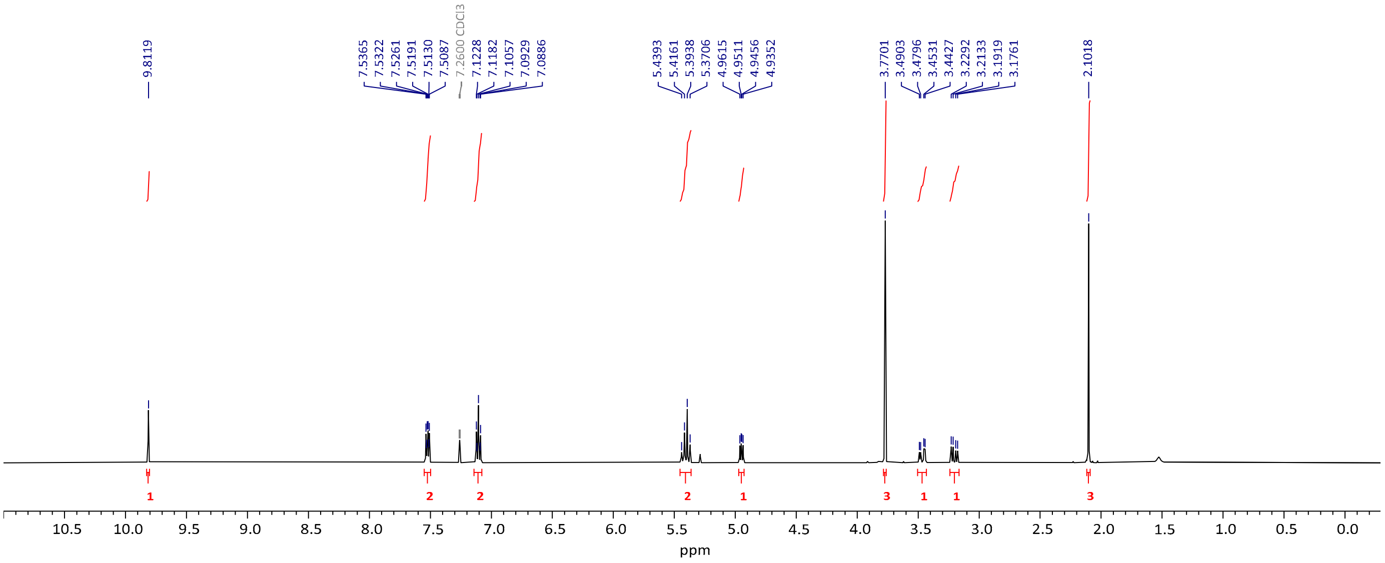

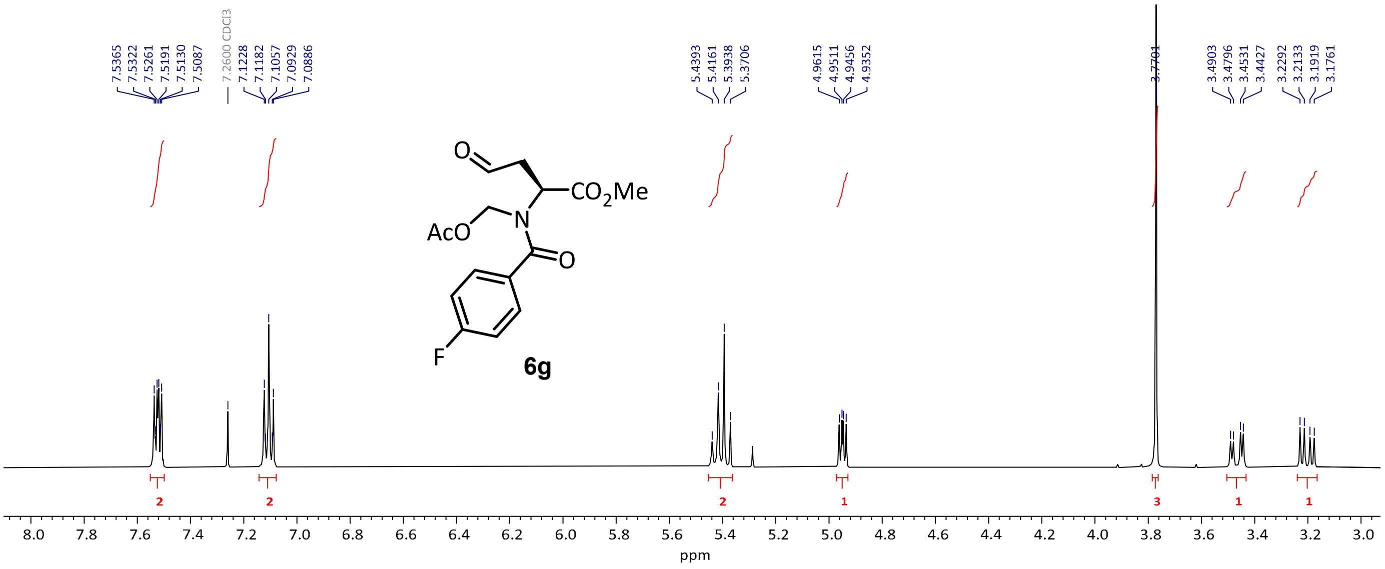

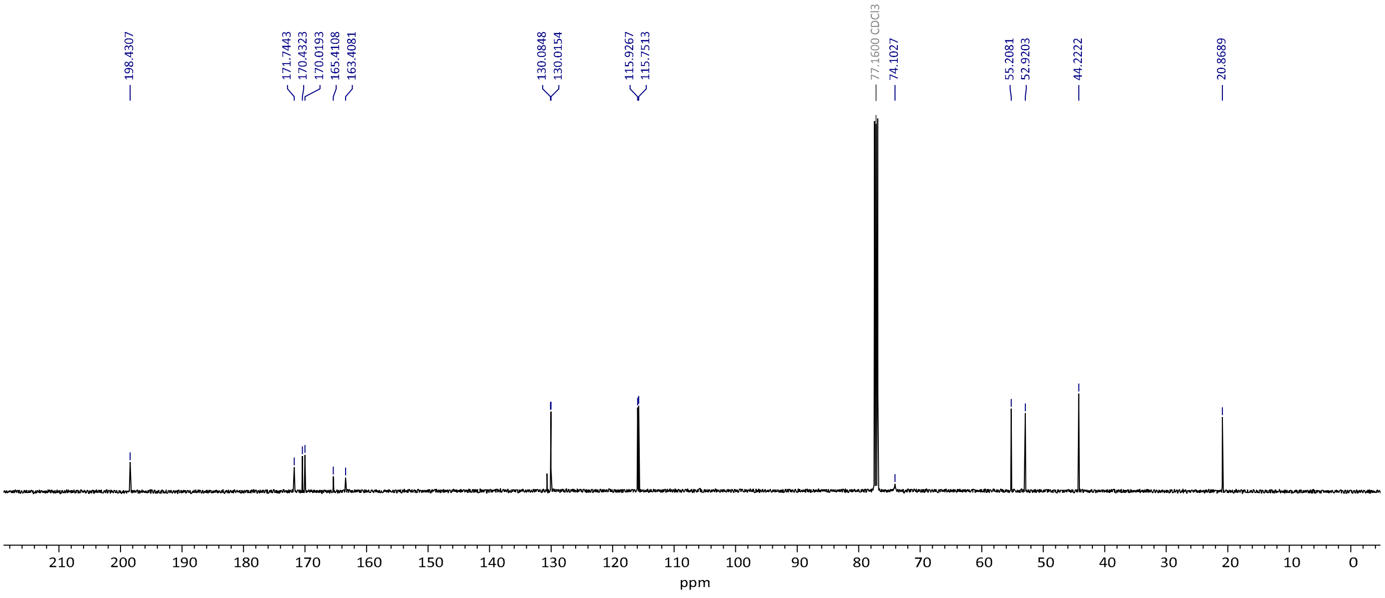

Compound **6h**, ^{1}H and ^{13}C NMR at 26 °C in $CDCl_3$

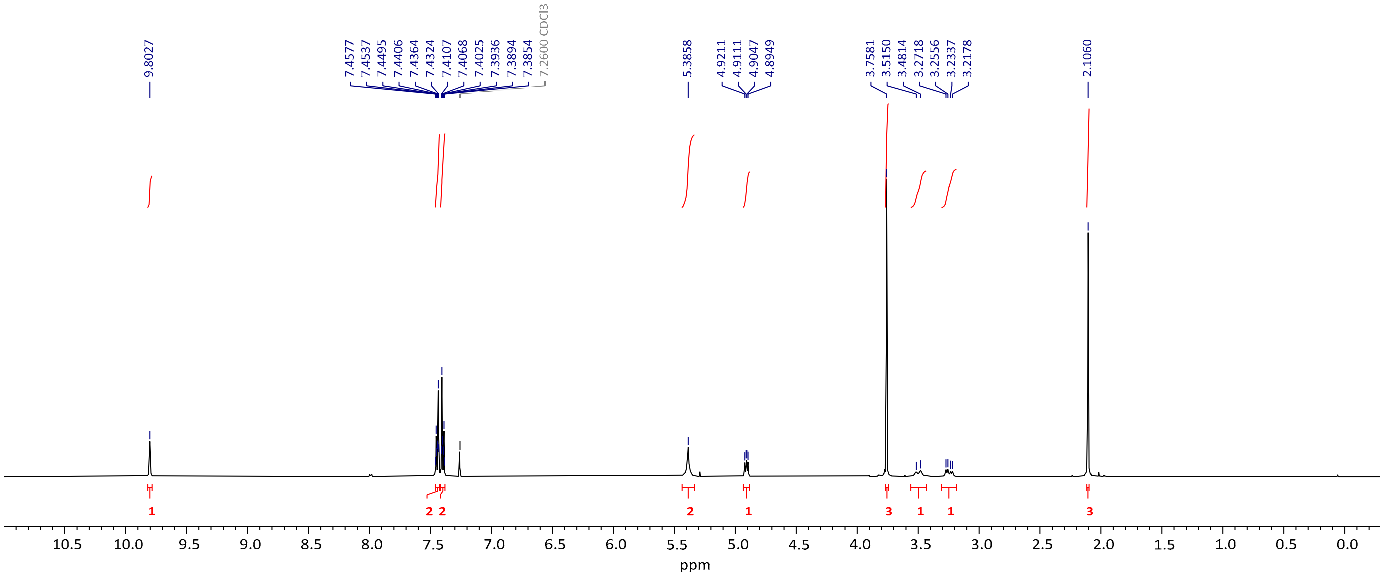

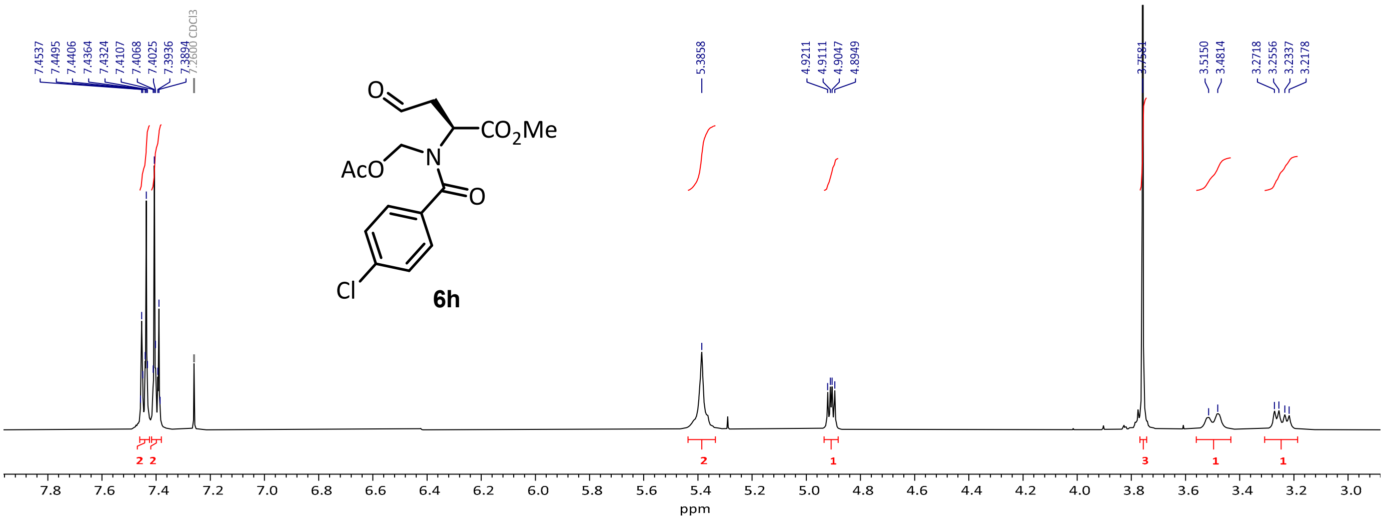

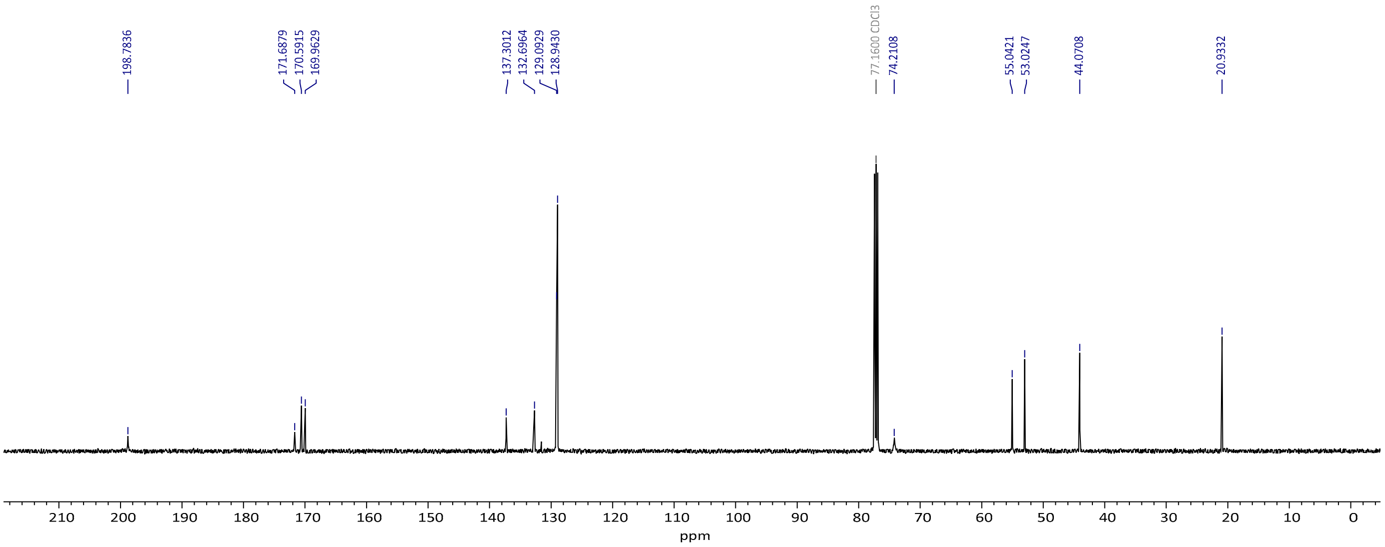

Compound **6i**, ^{1}H and ^{13}C NMR at 26 °C in $CDCl_3$

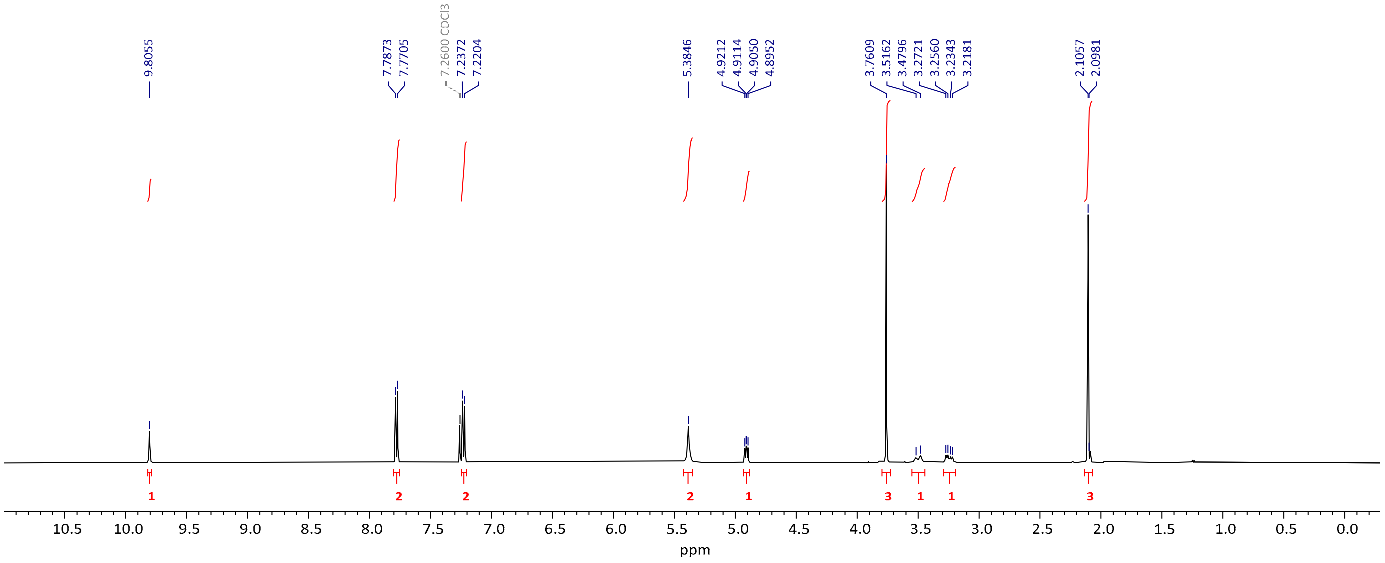

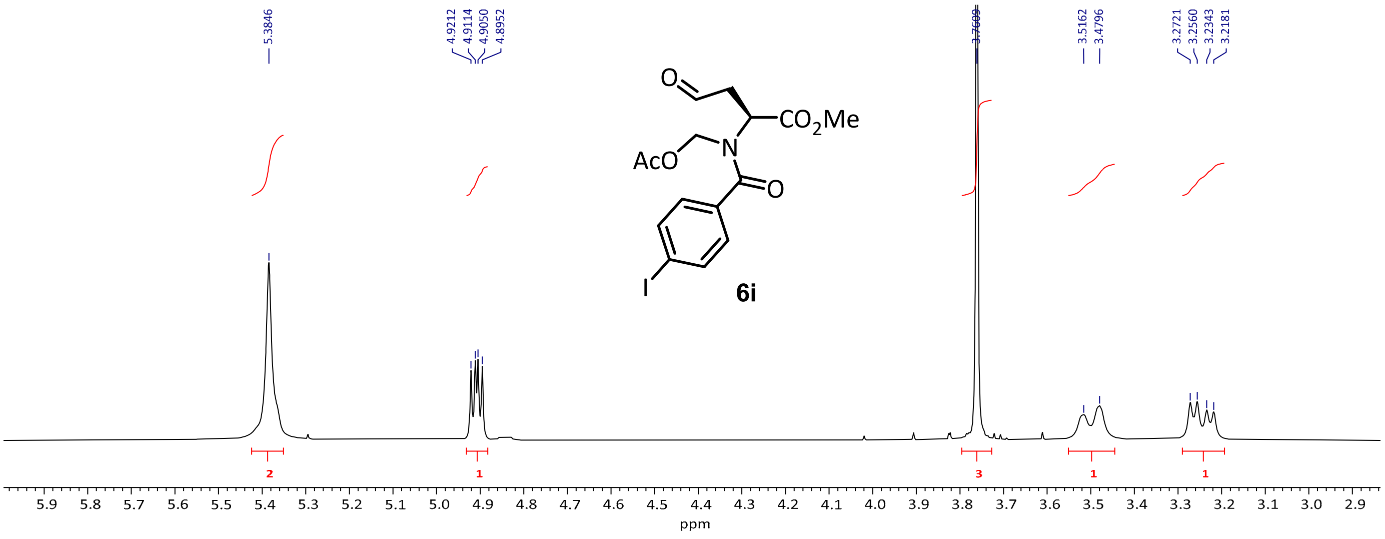

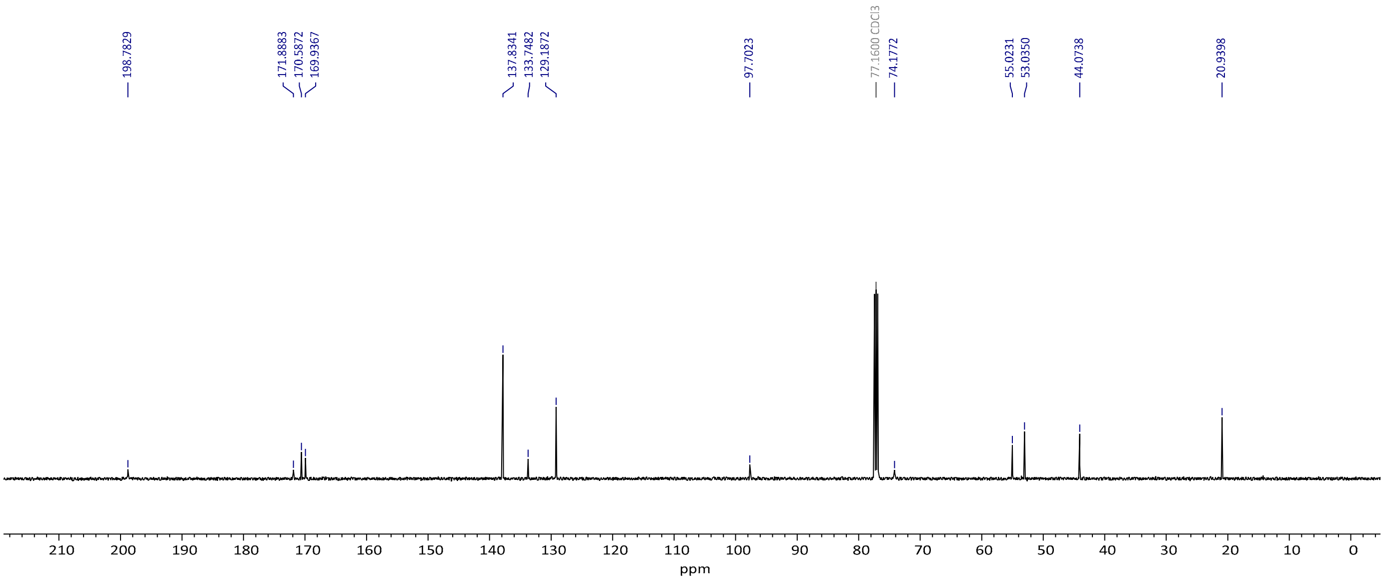

Compound **6j**, ^{1}H and ^{13}C NMR at 26 °C in $CDCl_3$

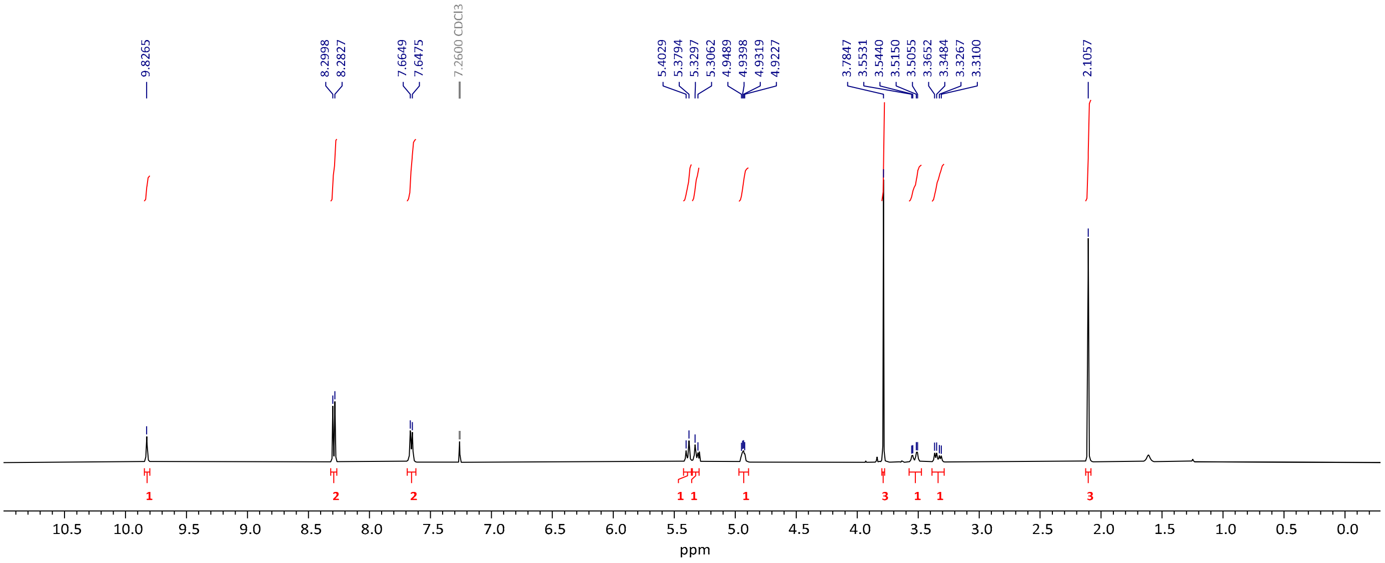

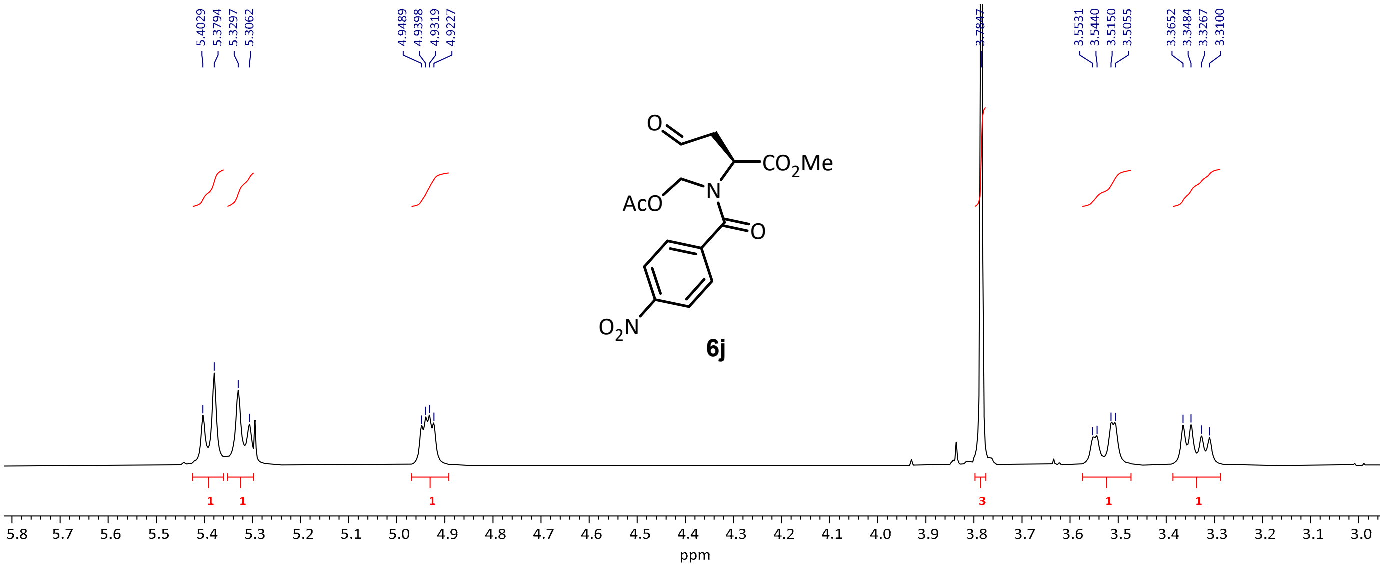

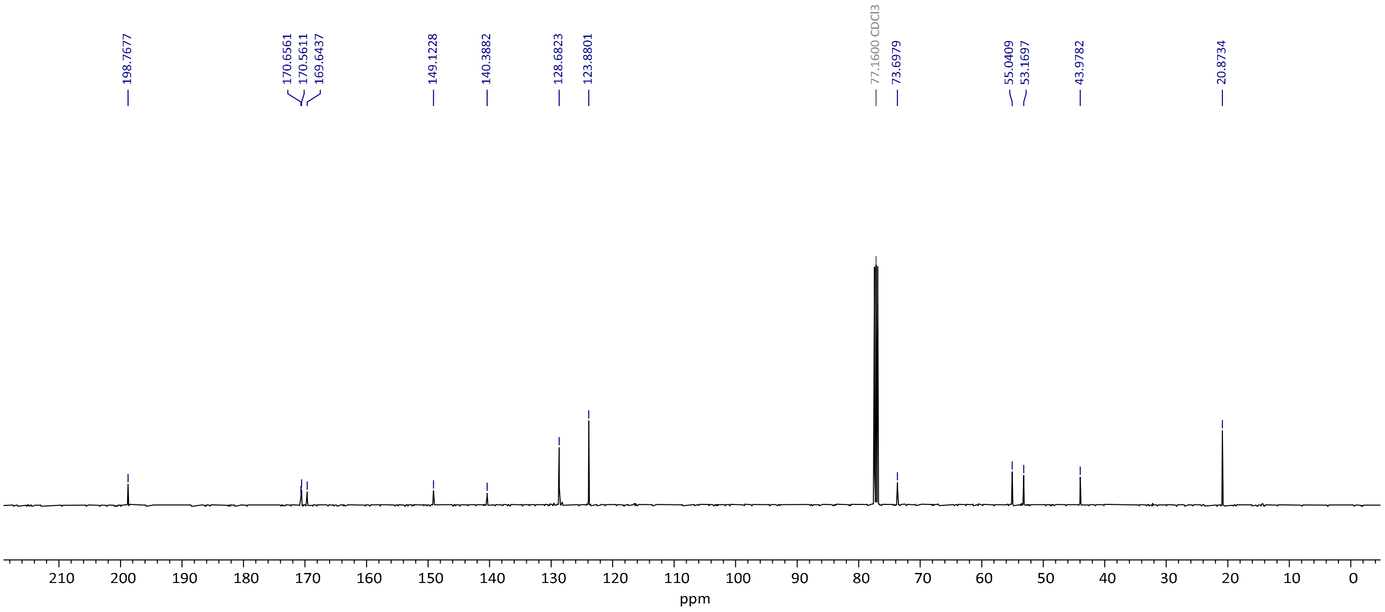

Compound **6k**, ^{1}H and ^{13}C NMR at 26 °C in CDCl3

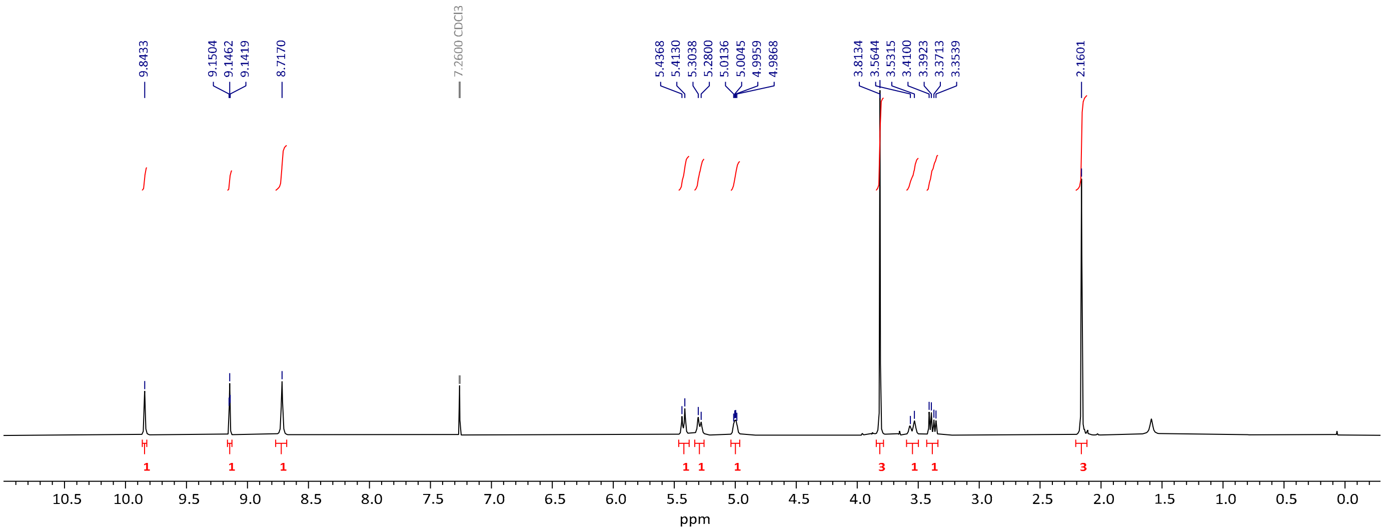

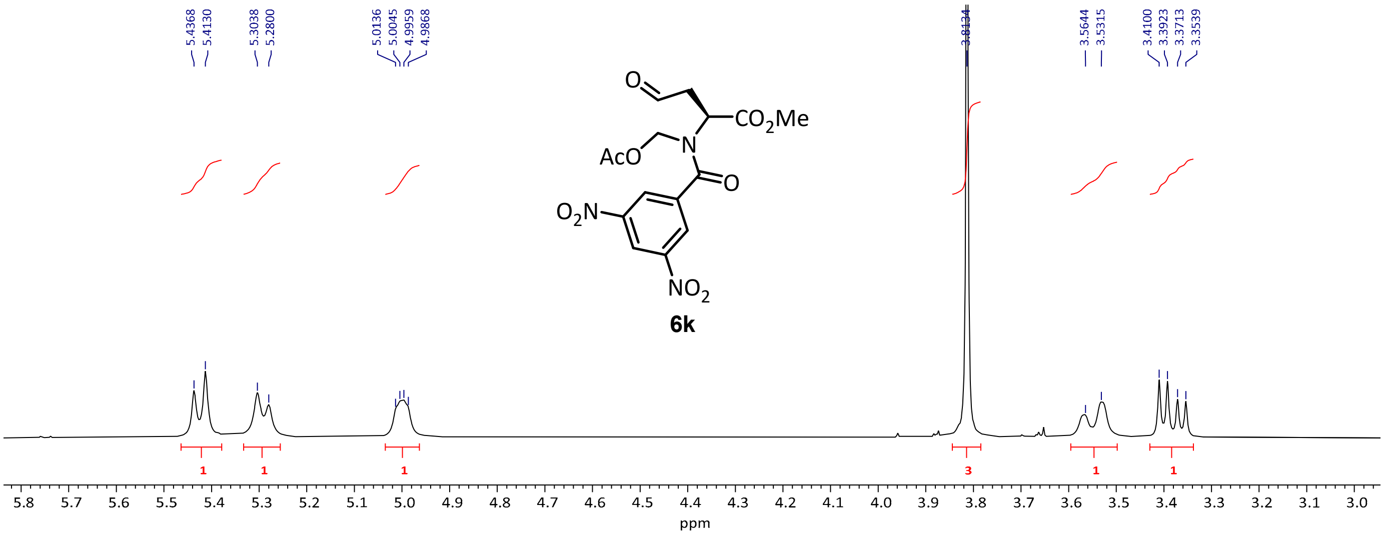

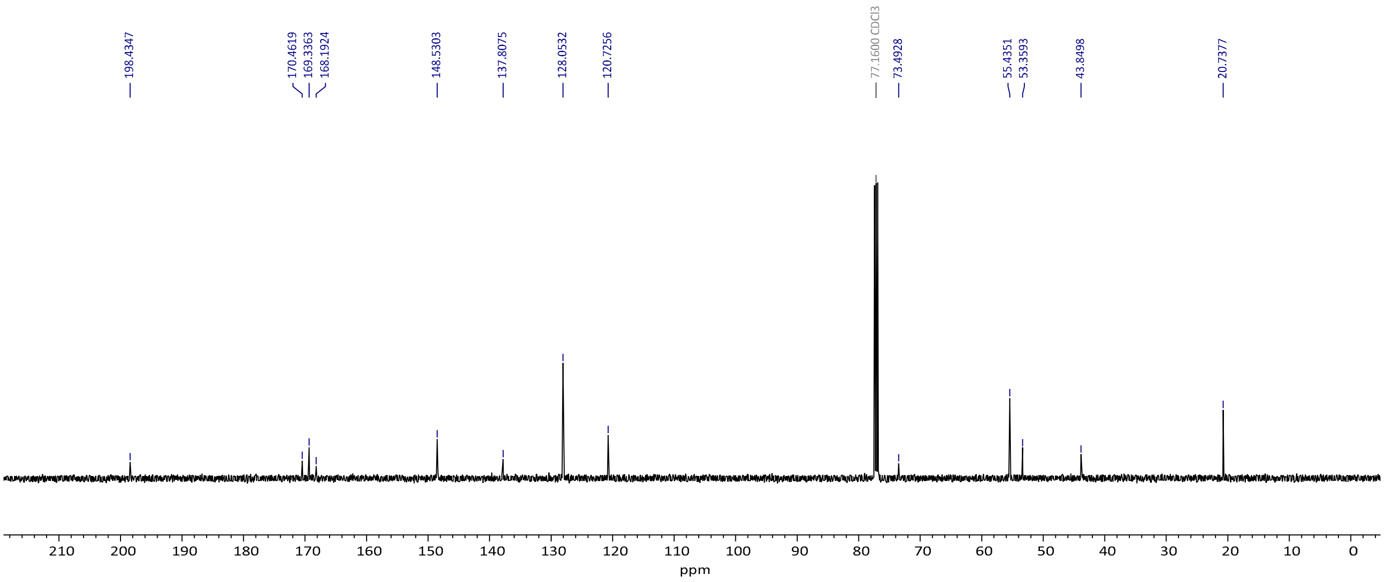

Compound **6l**, ^{1}H and ^{13}C NMR at 26 °C in CDCl3

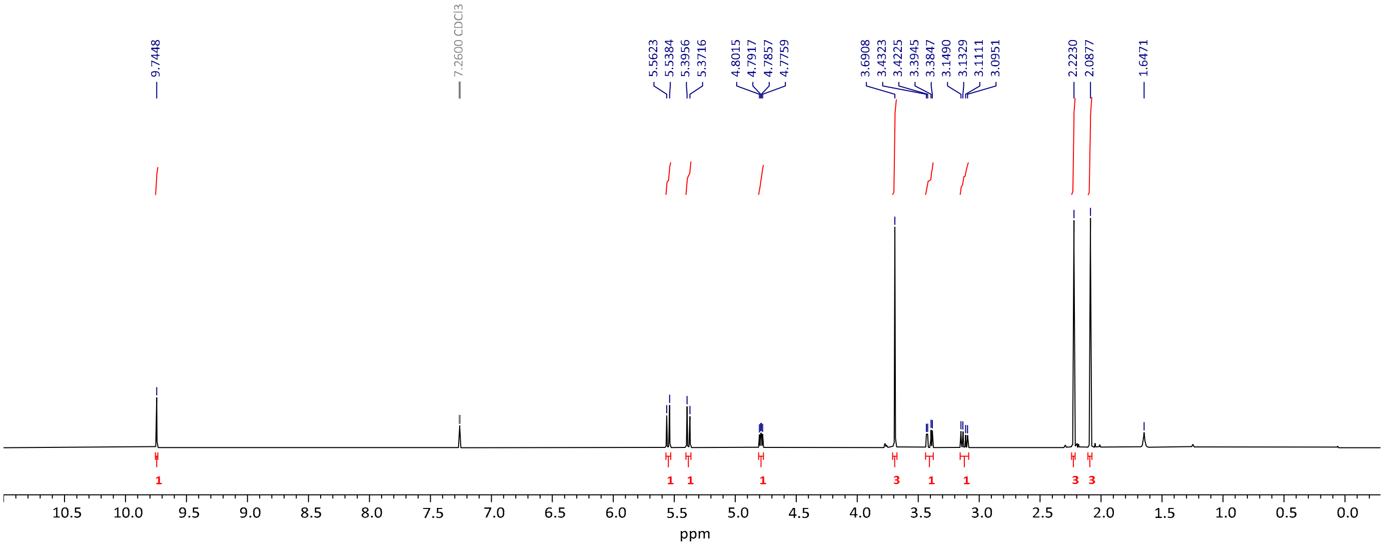

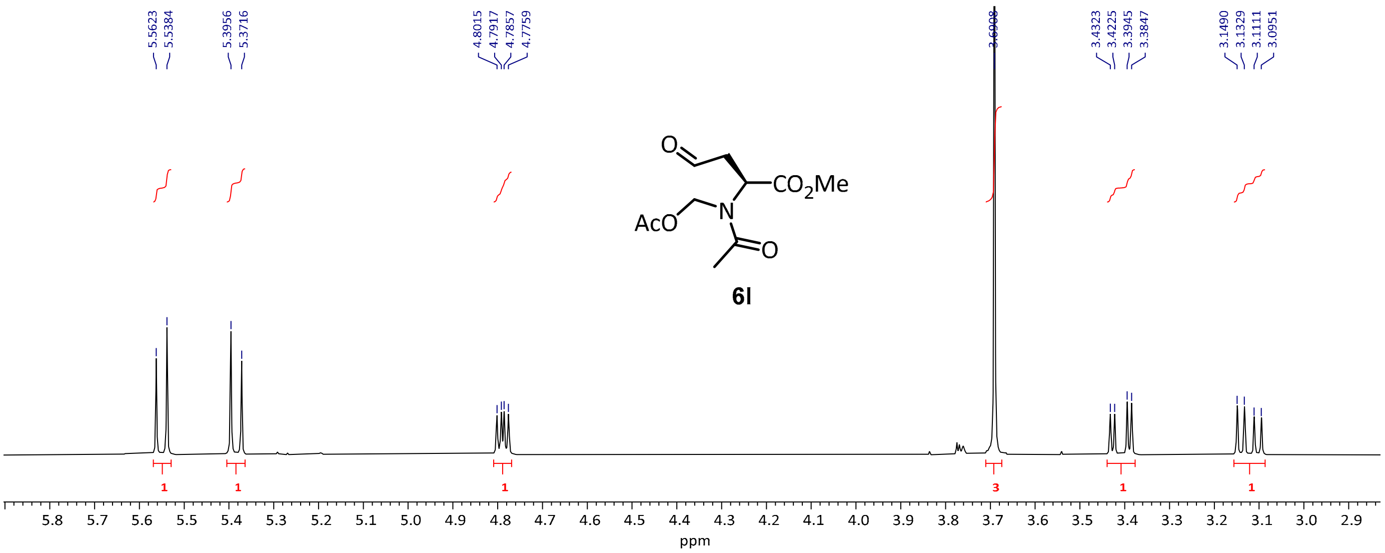

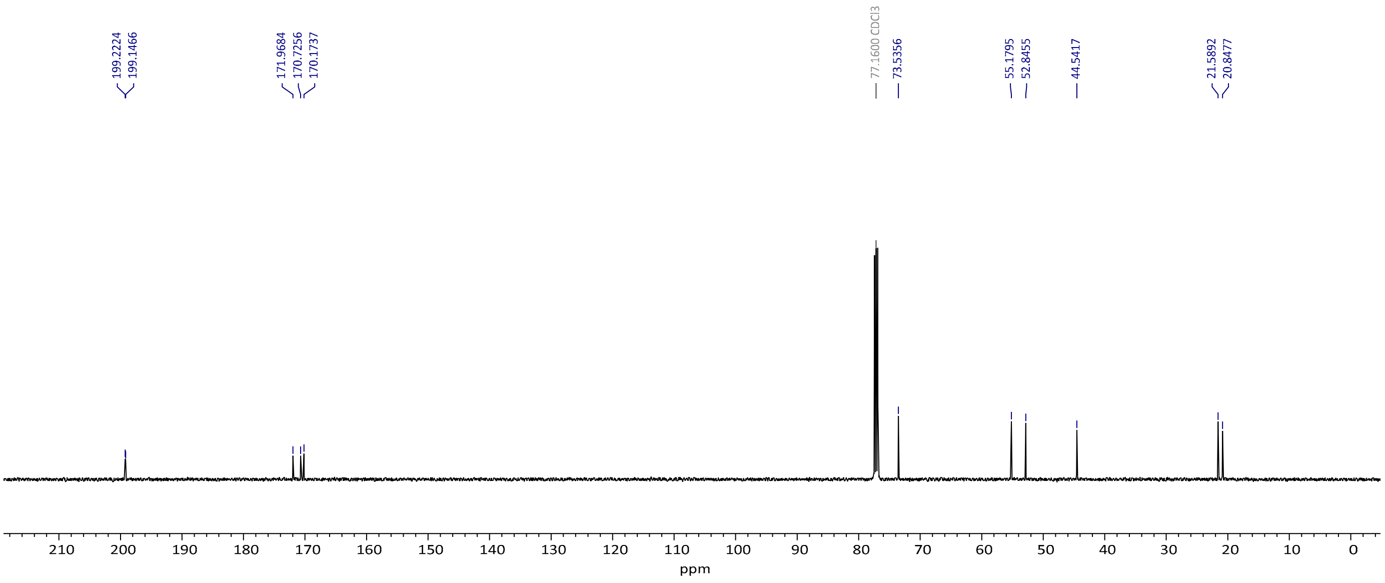

Compound **6m**, ^{1}H and ^{13}C NMR at 26 °C in $CDCl_3$

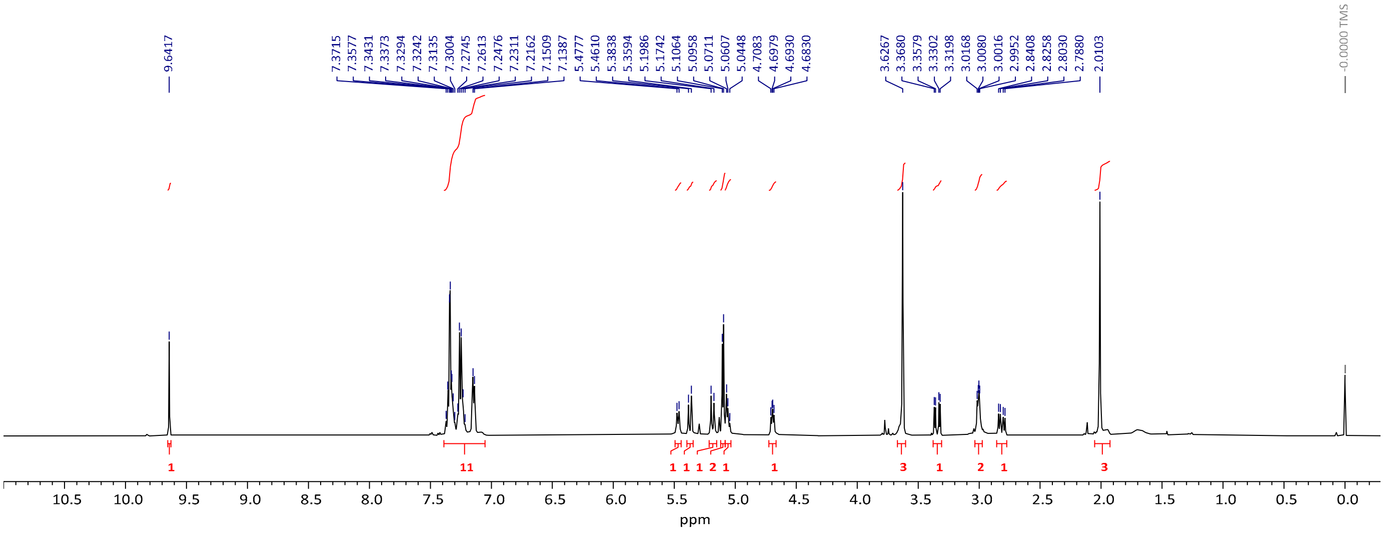

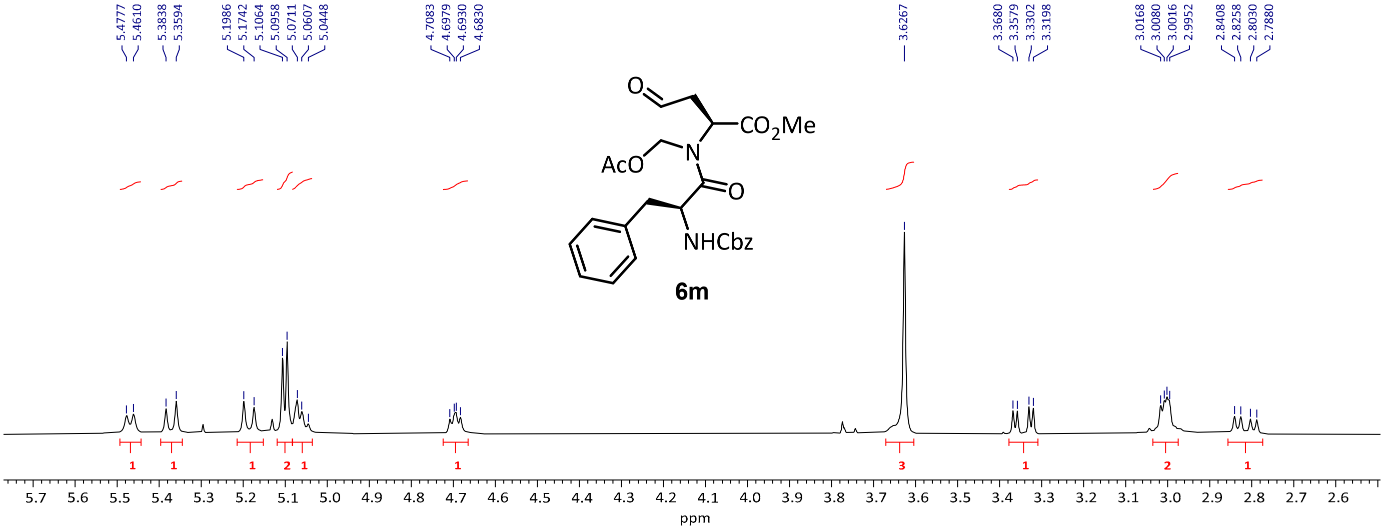

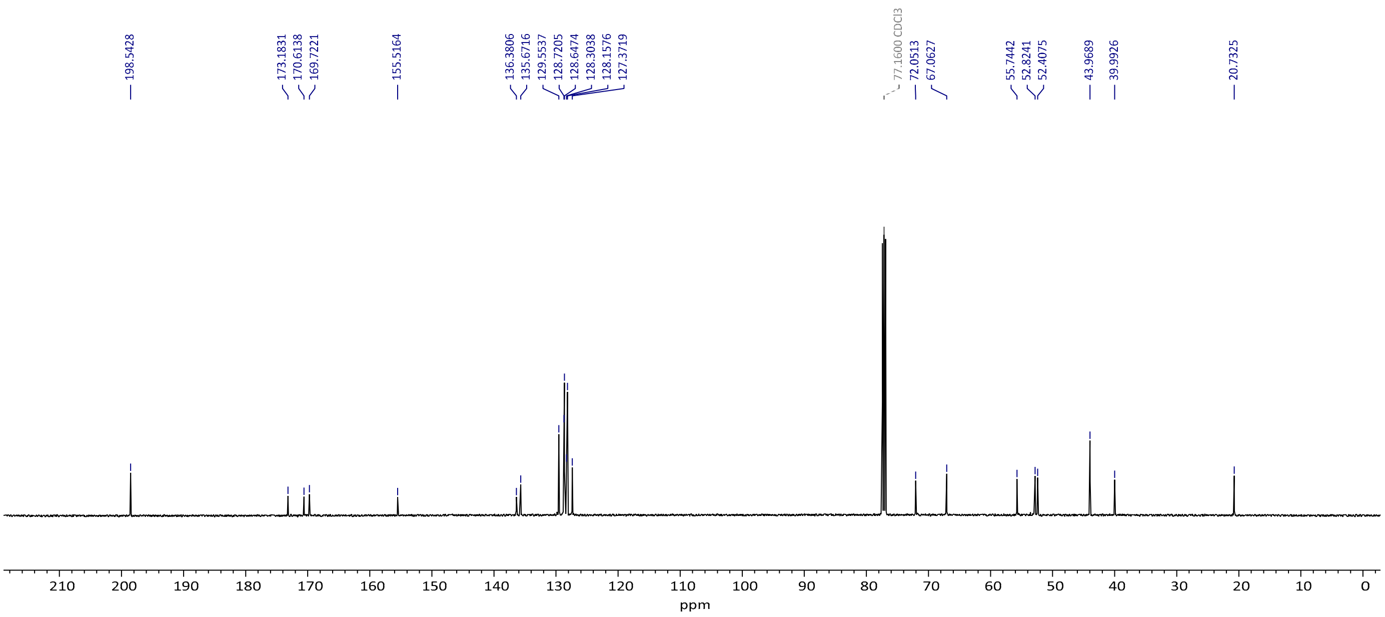

Compound **6o**, ^{1}H and ^{13}C NMR at 70 °C in CD_3CN

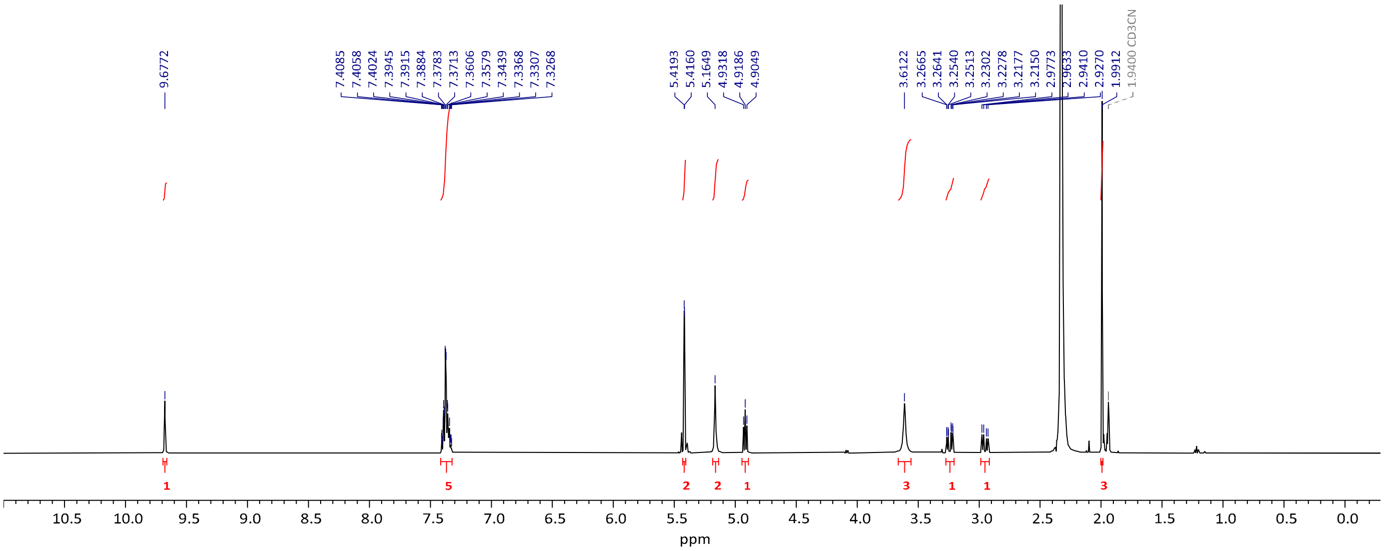

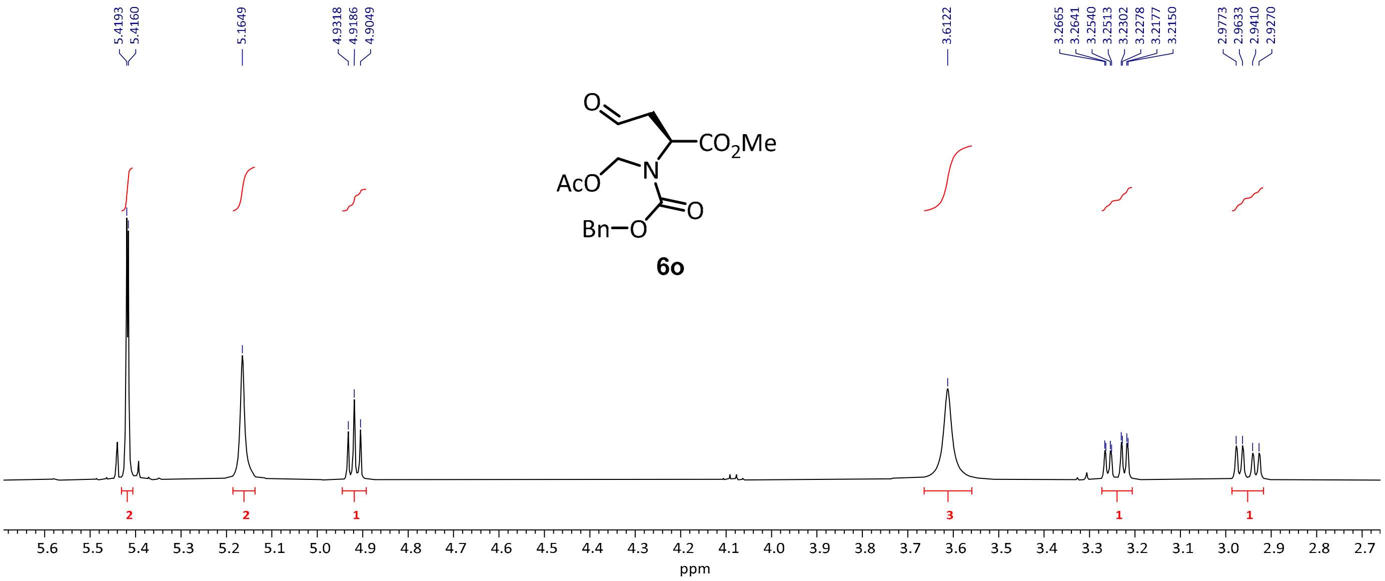

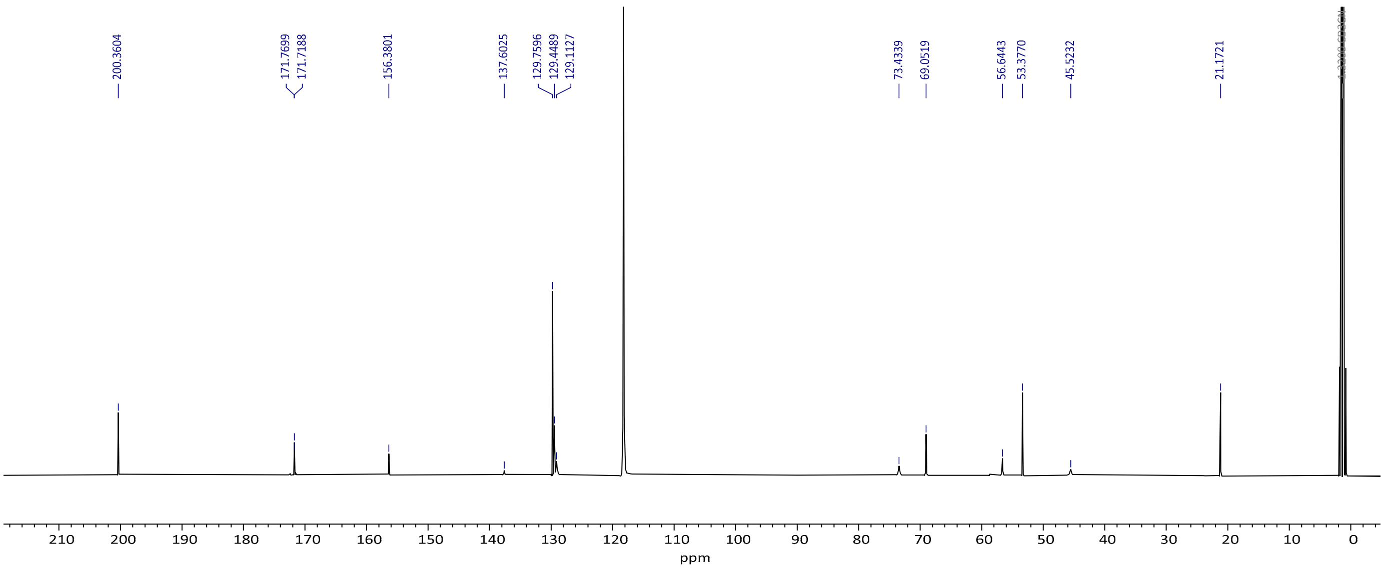

Compound **6p**, ^{1}H and ^{13}C NMR at 70 °C in CD_3CN

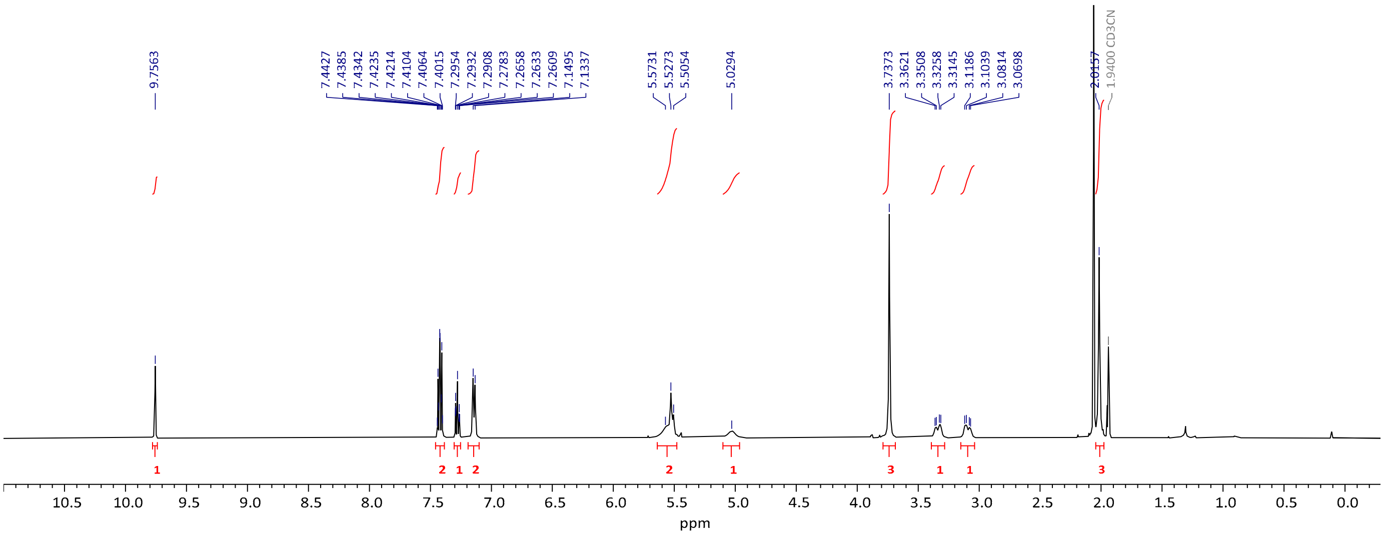

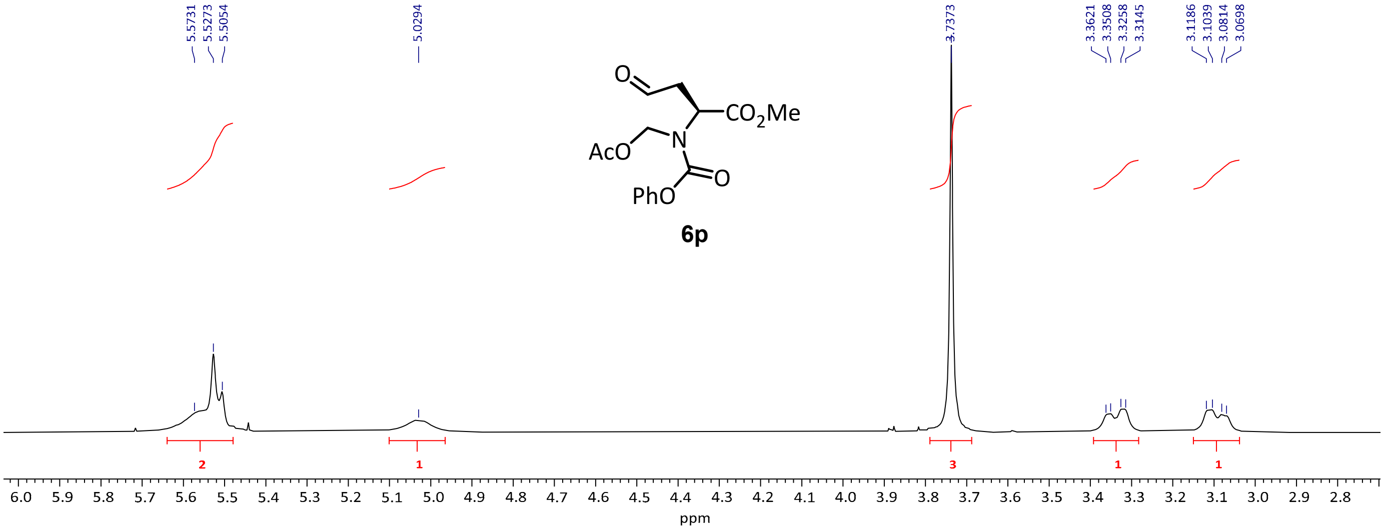

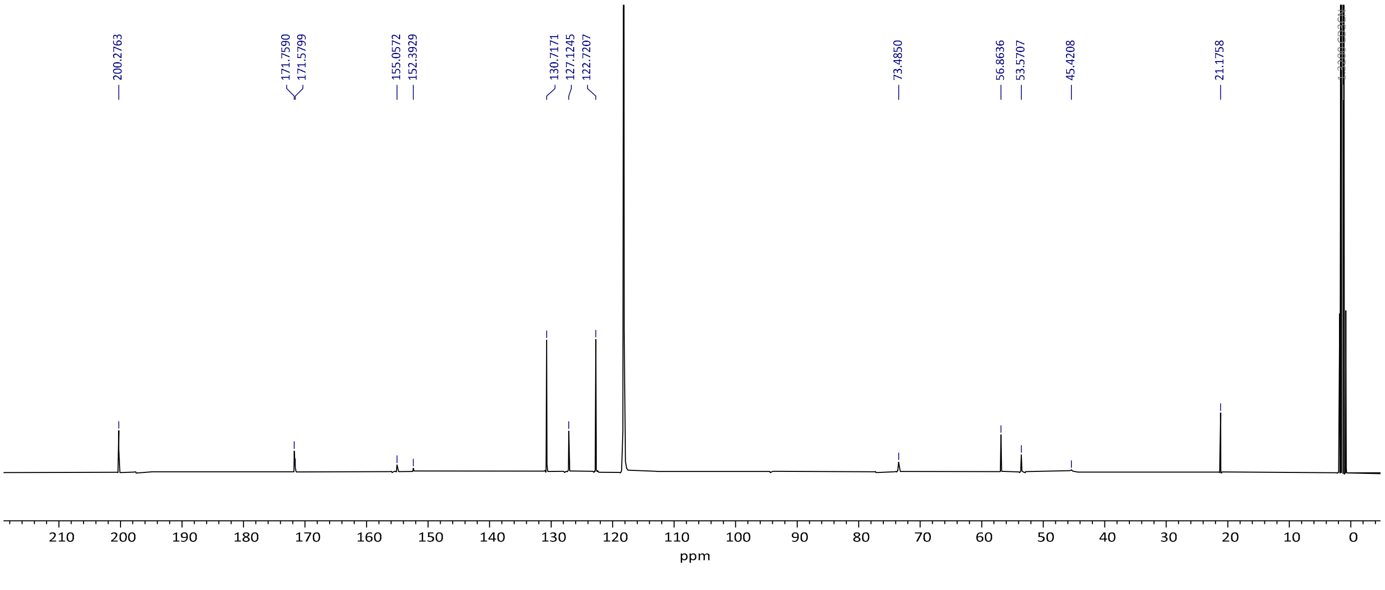

Compound **7a**, ^{1}H and ^{13}C NMR at 26 °C in $CDCl_3$

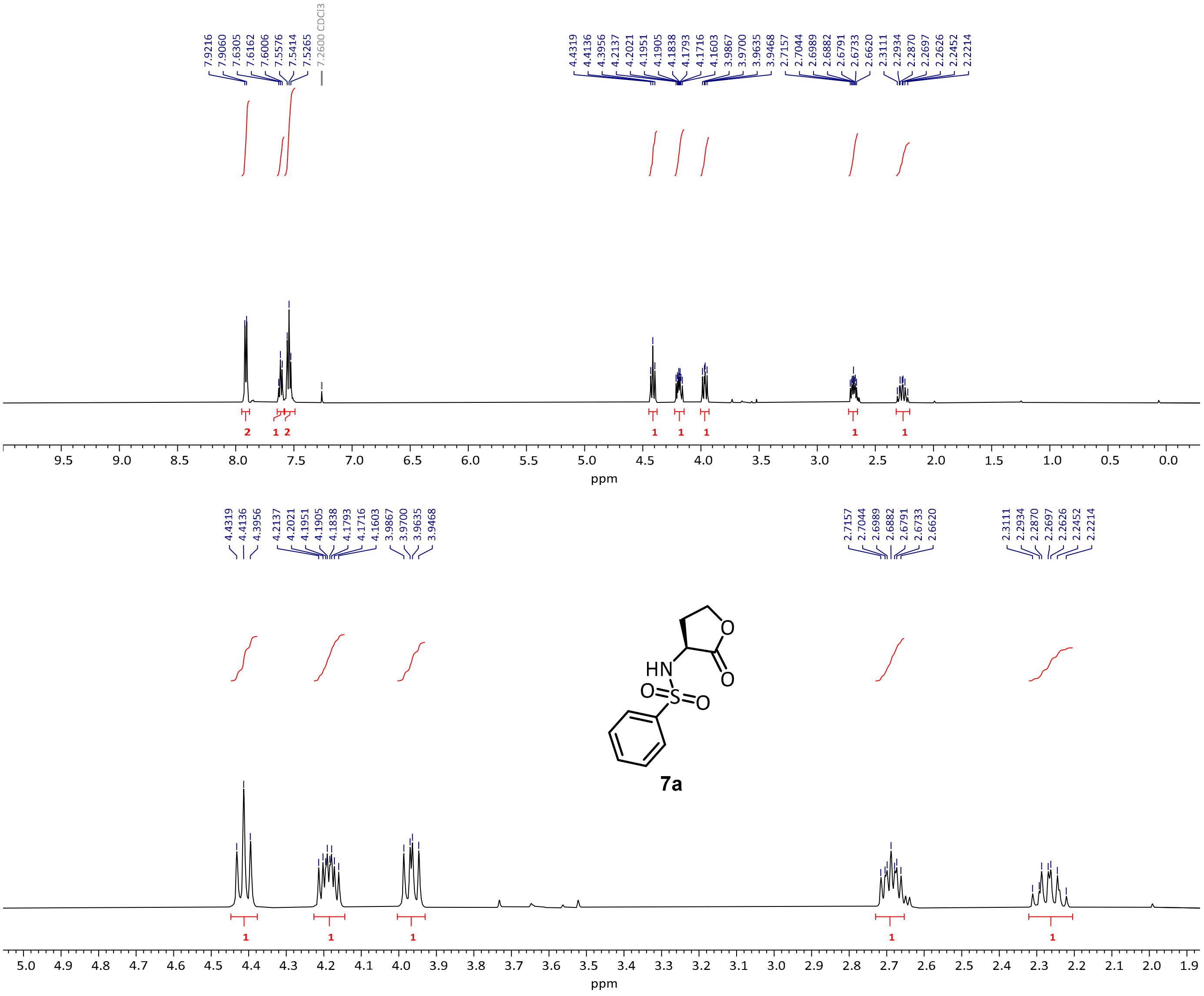

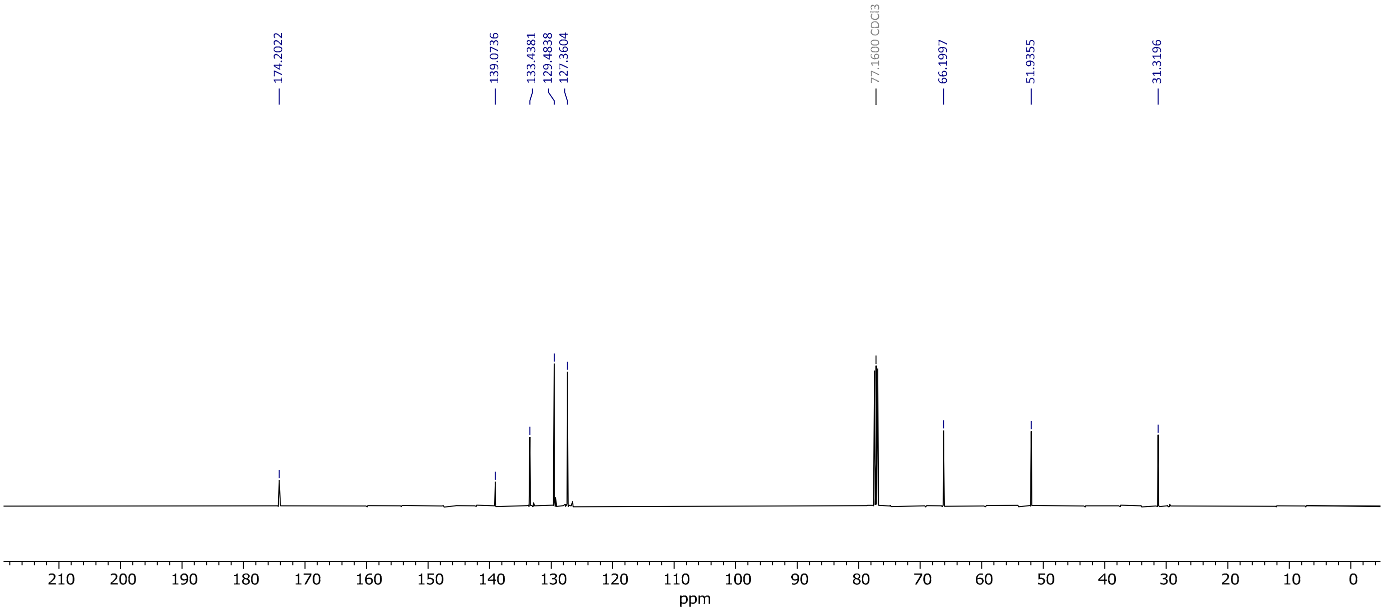

Compound **7b**, ^{1}H and ^{13}C NMR at 55 °C in $CDCl_3$

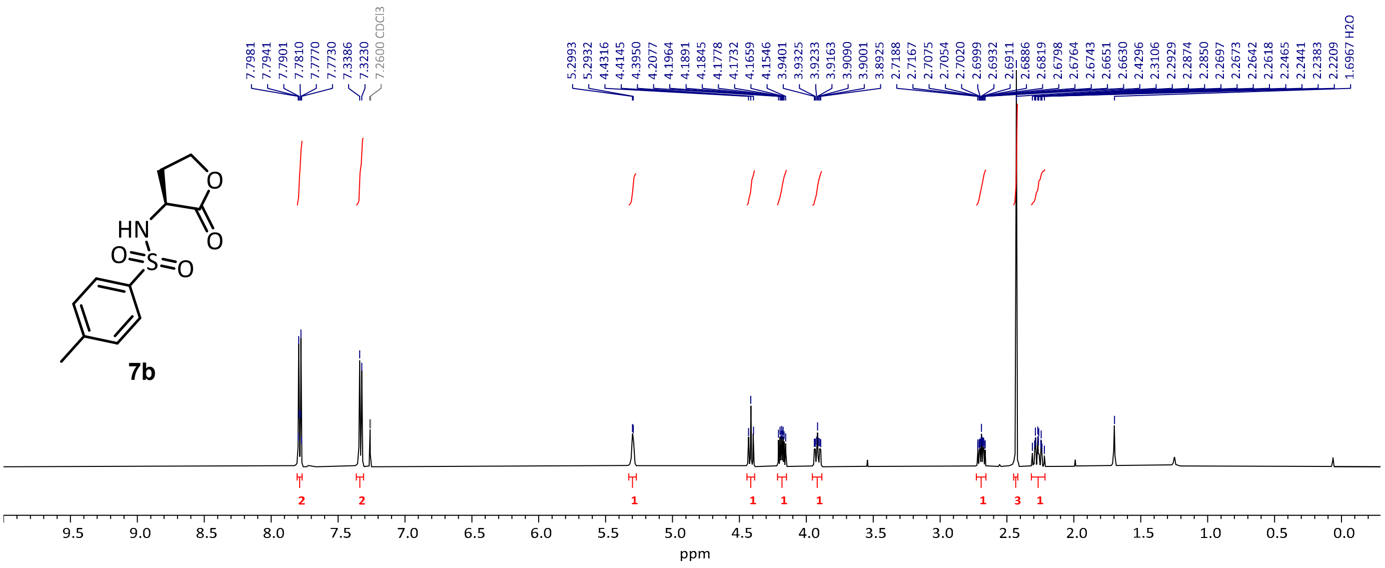

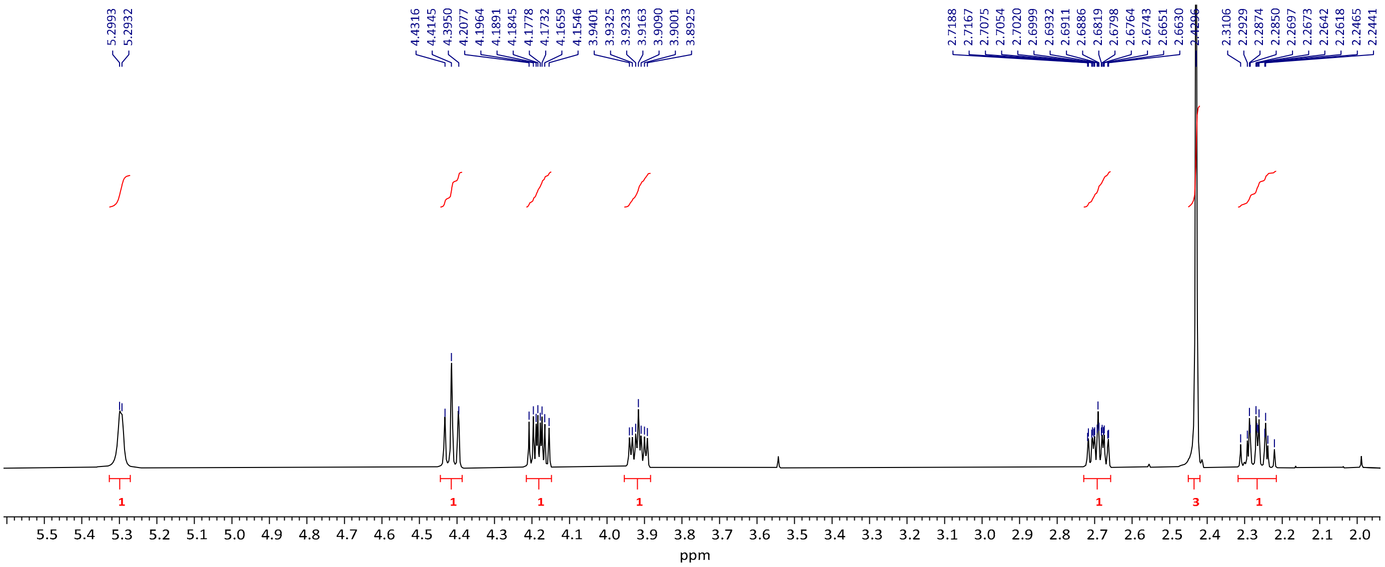

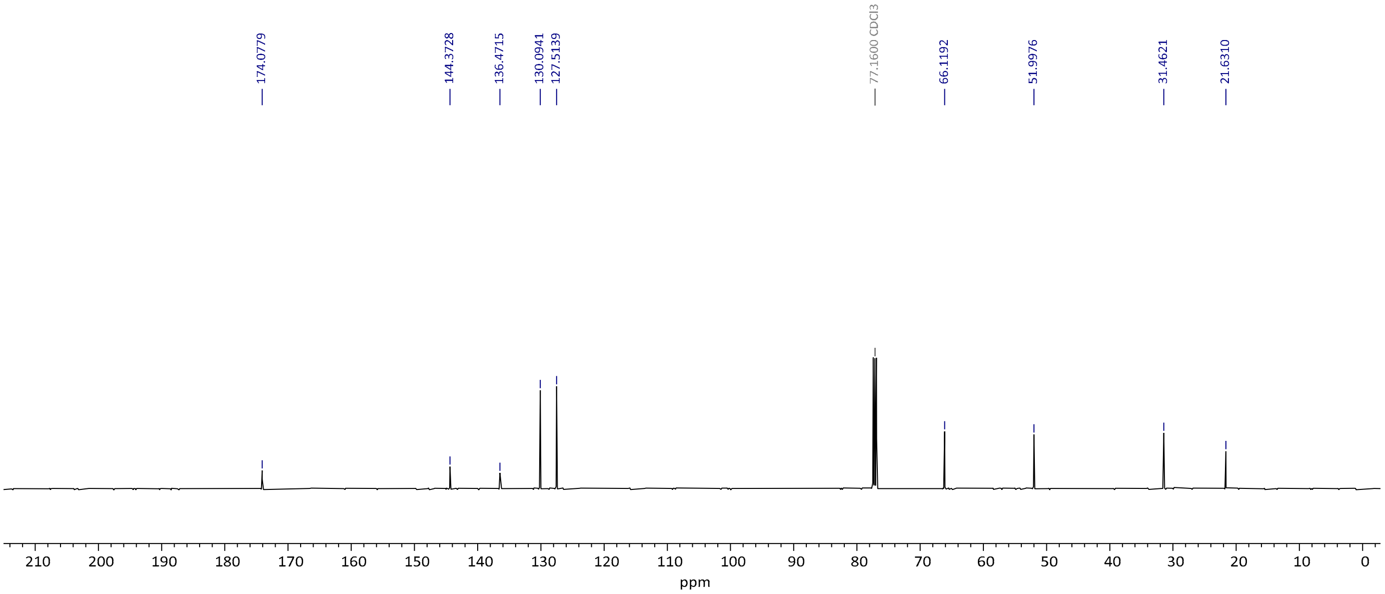

Compound **7c**, ^{1}H and ^{13}C NMR at 26 °C in $CDCl_3$

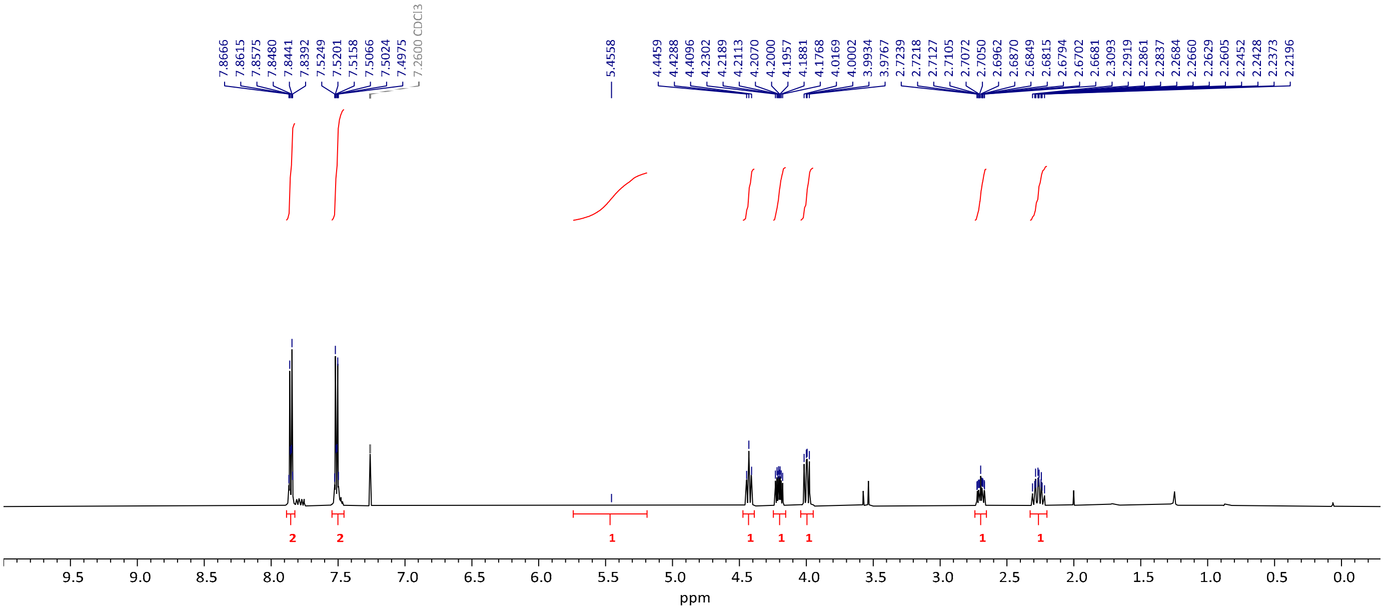

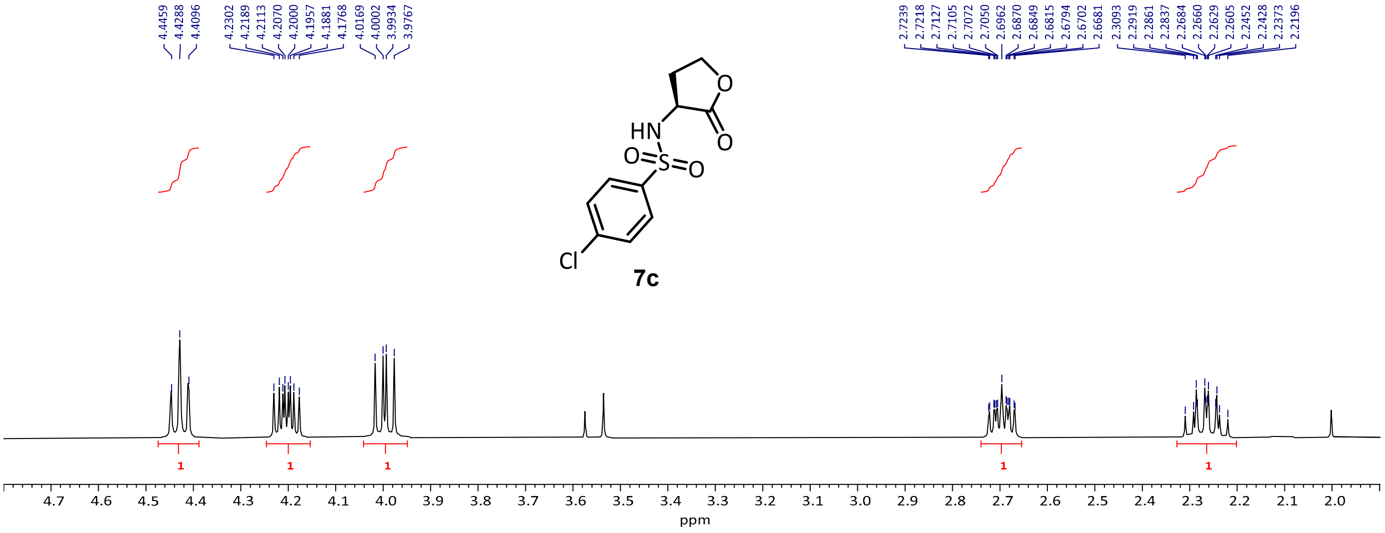

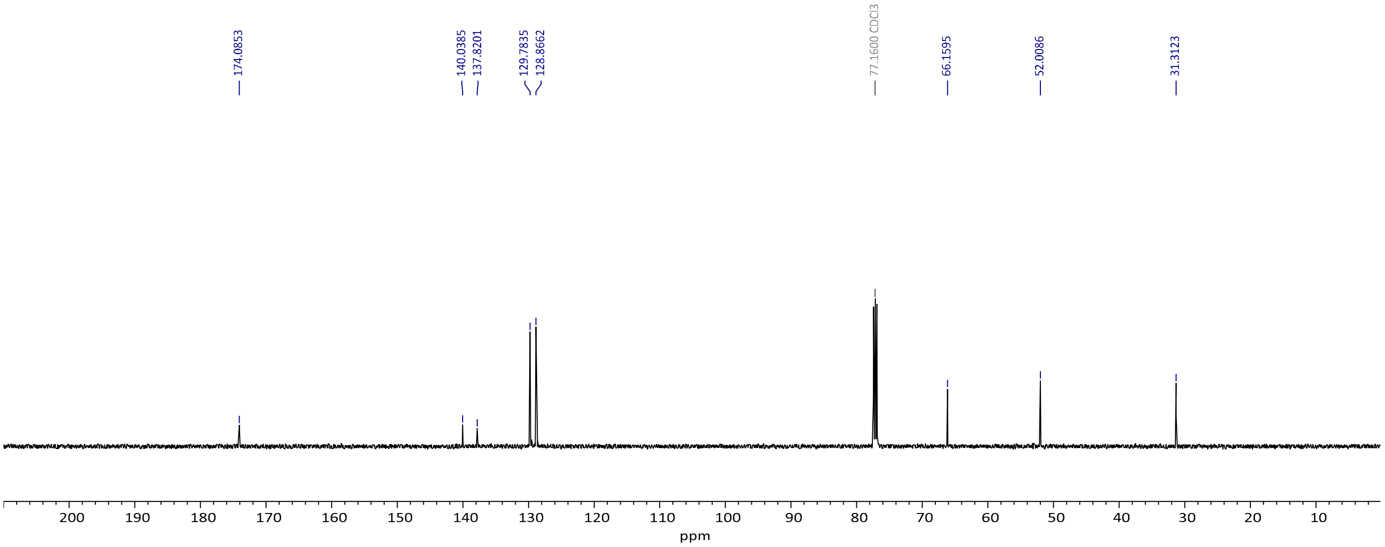

Compound **7d**, ^{1}H and ^{13}C NMR at 26 °C in $(CD_3)_2CO$

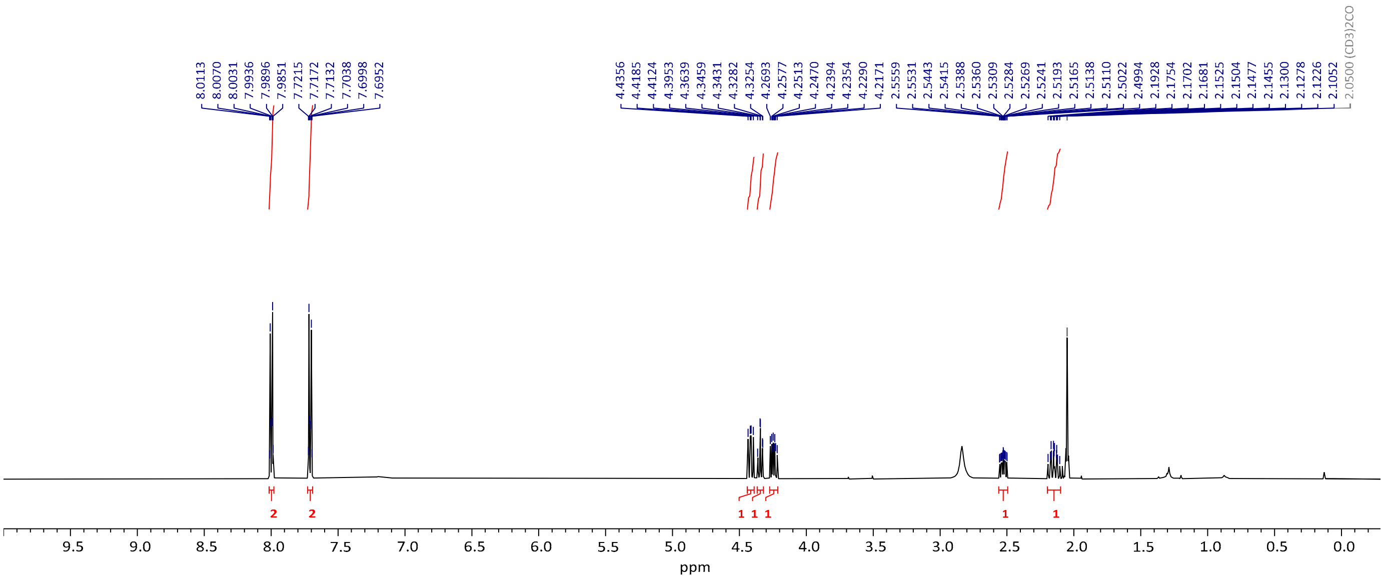

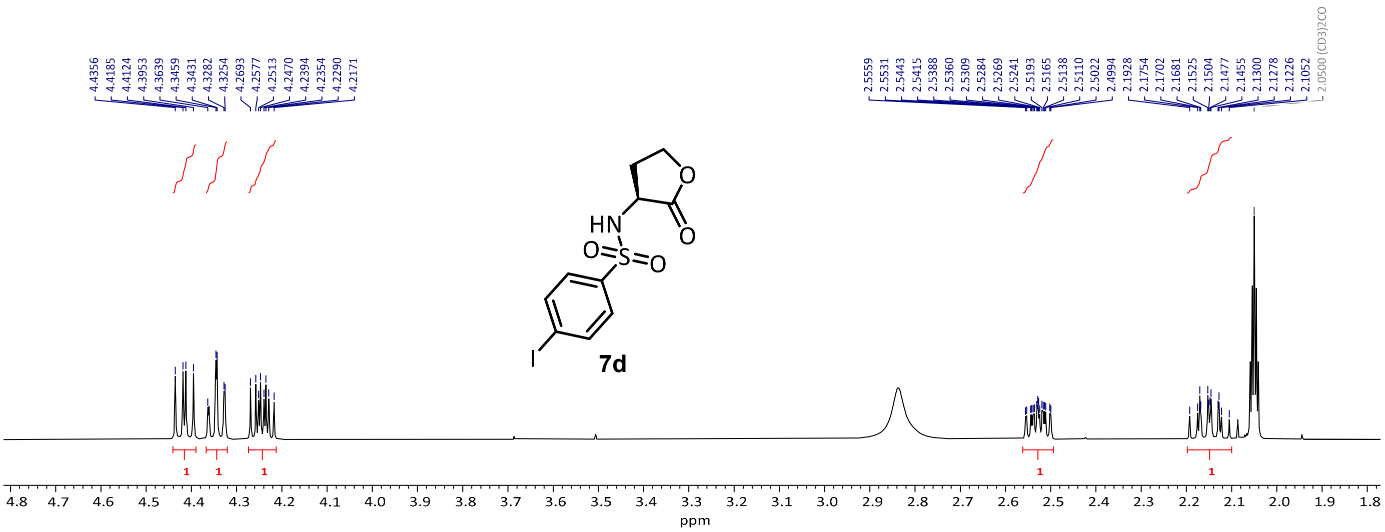

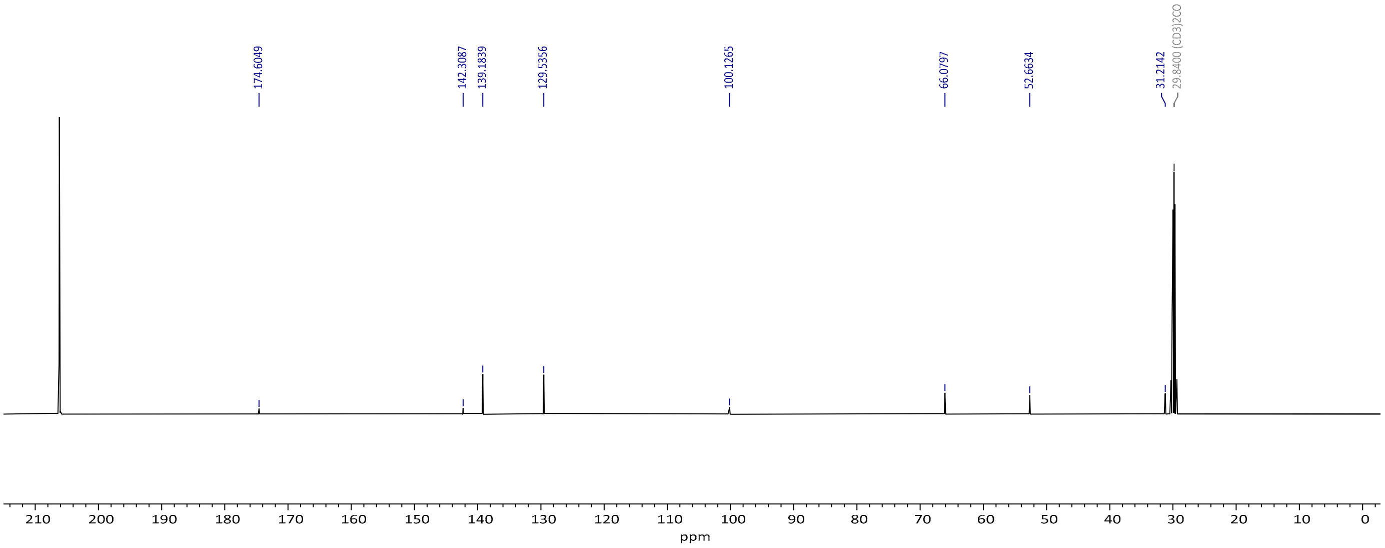

Compound **7e**, ^{1}H and ^{13}C NMR at 26 °C in $(CD_3)_2CO$

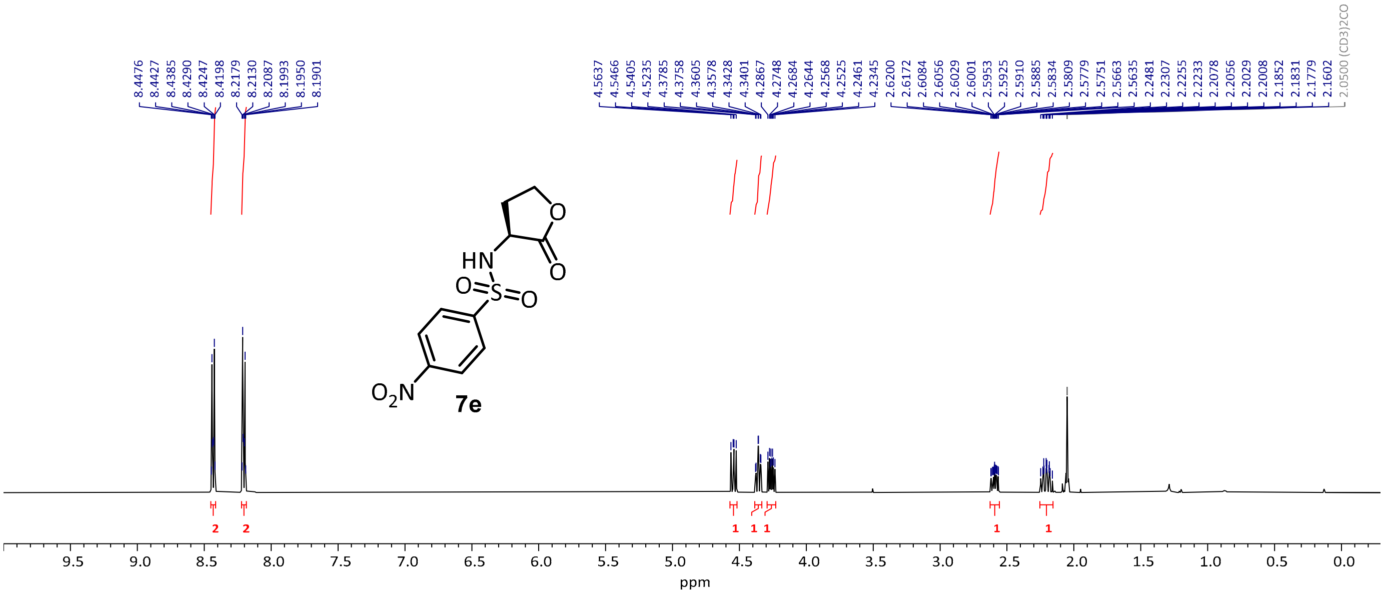

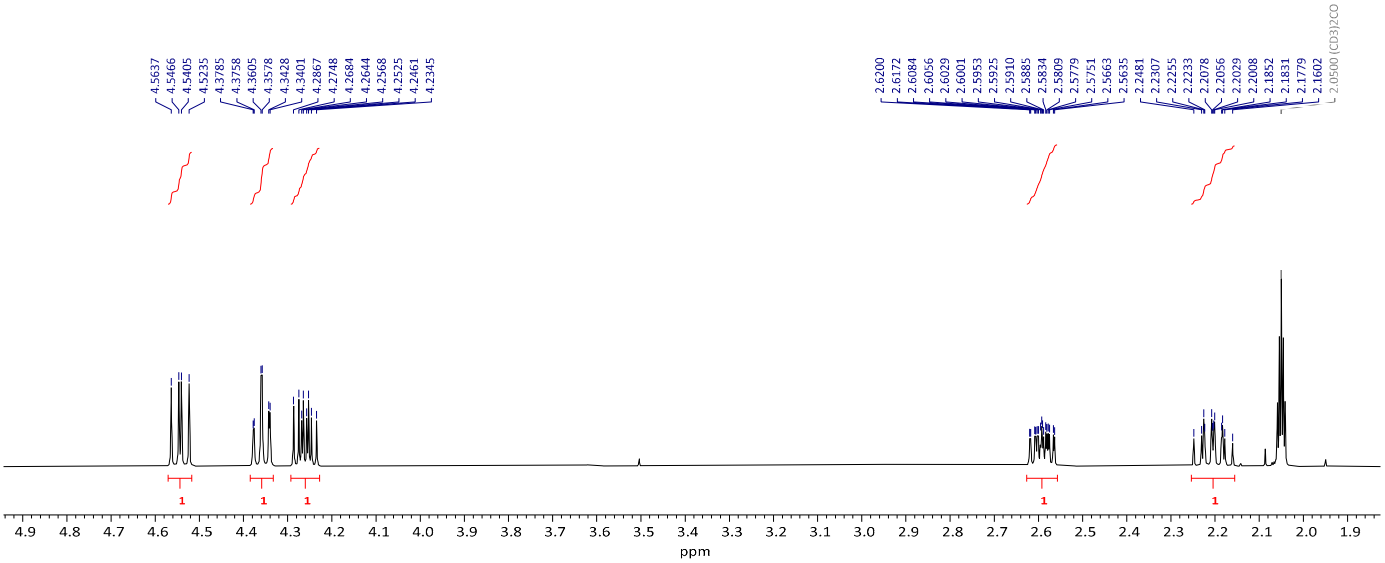

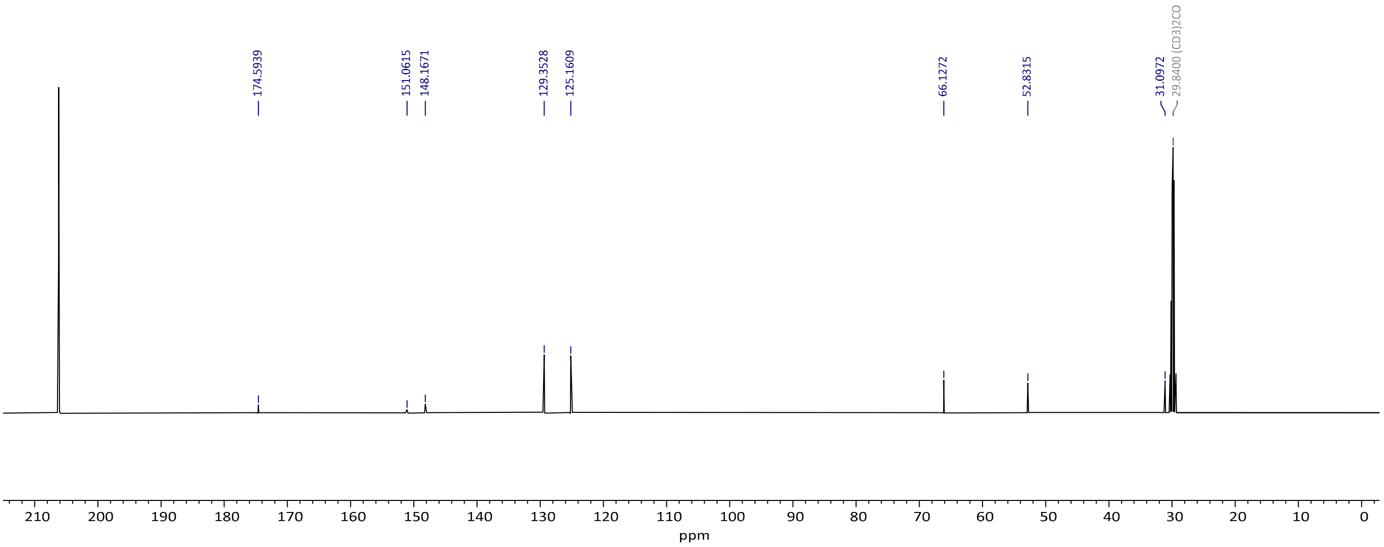

Compound **7f**, ^{1}H and ^{13}C NMR at 70 °C in CD_3CN

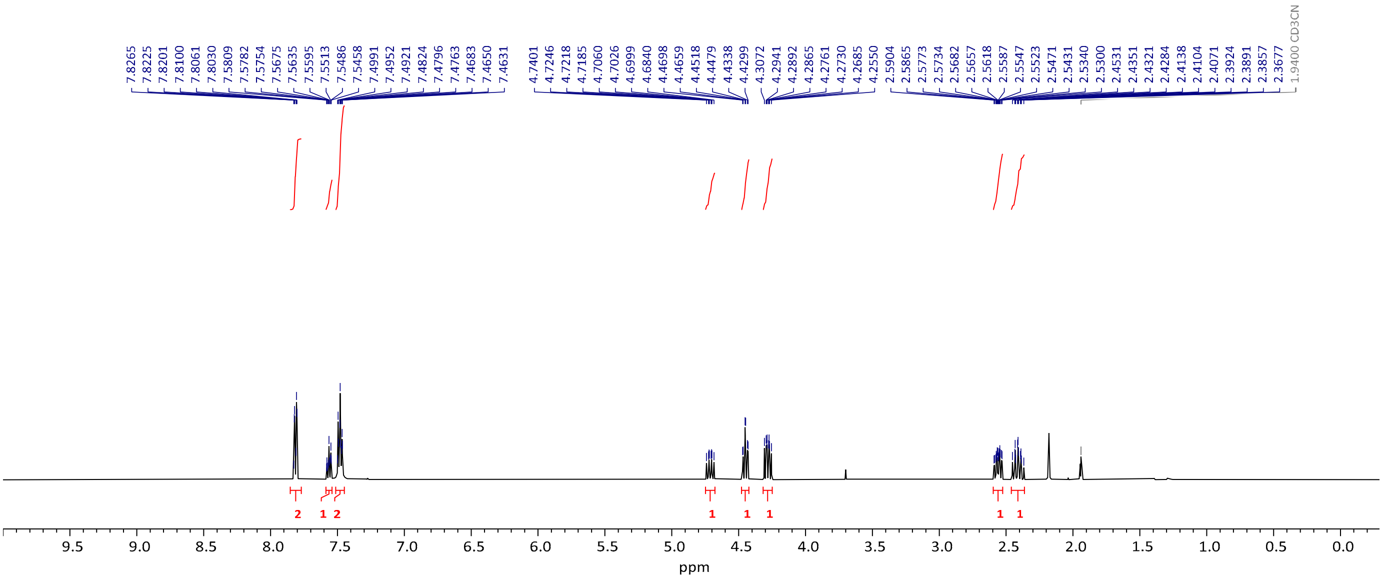

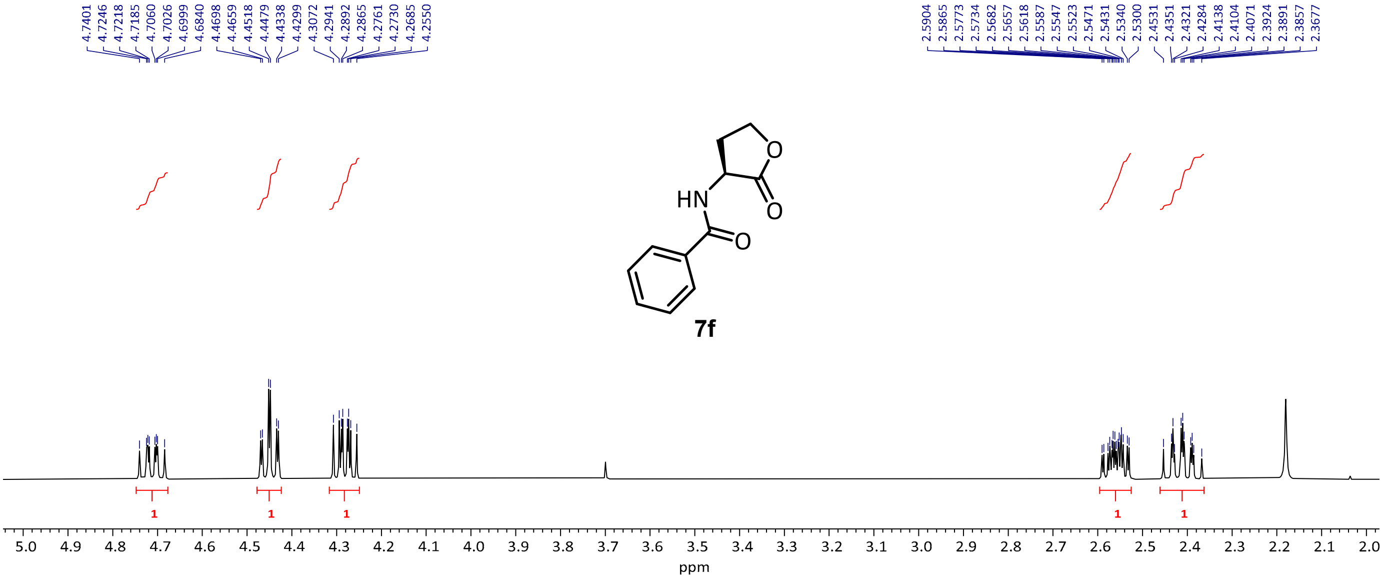

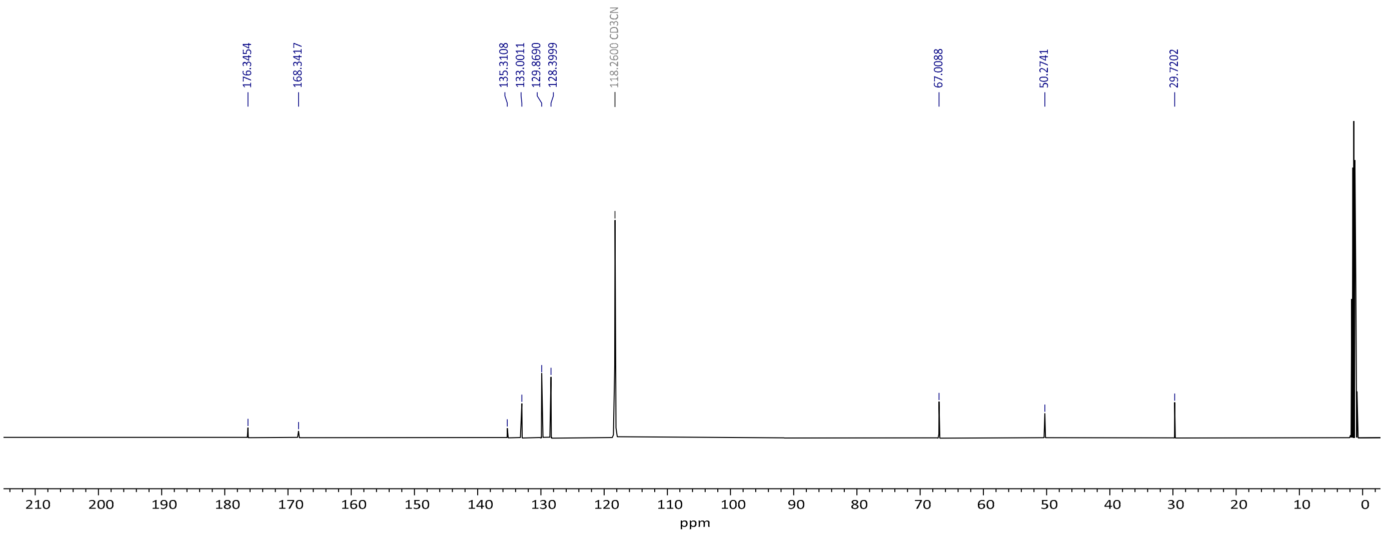

Compound **7g**, ^{1}H and ^{13}C NMR at 26 °C in $CDCl_3$

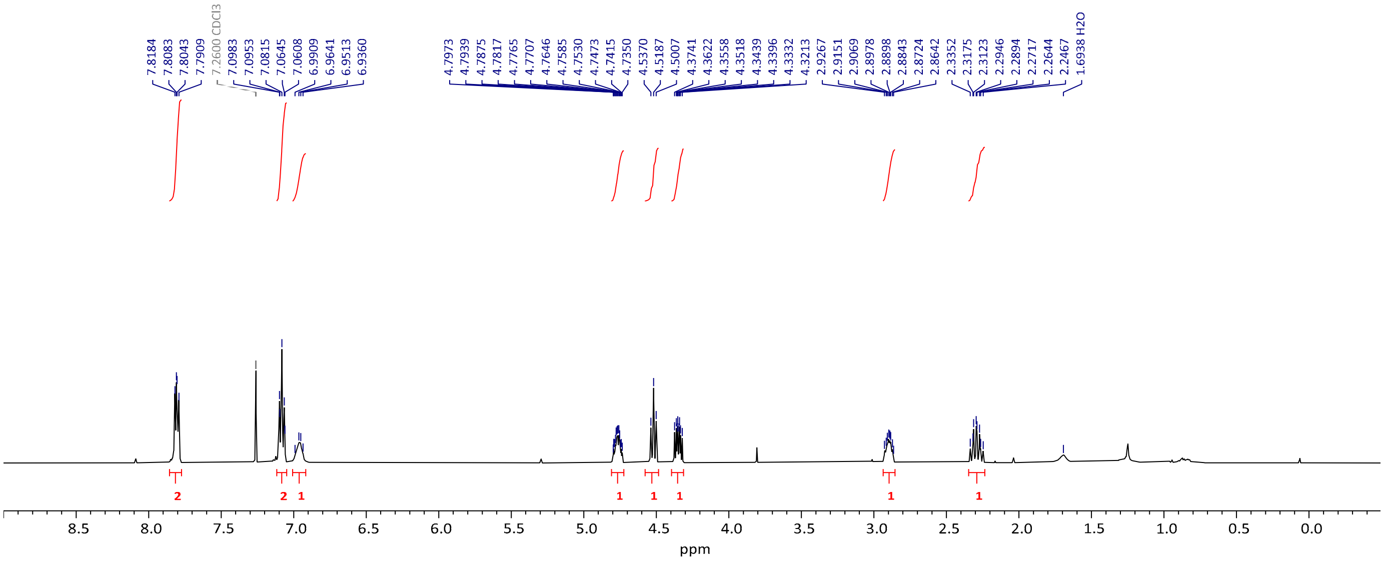

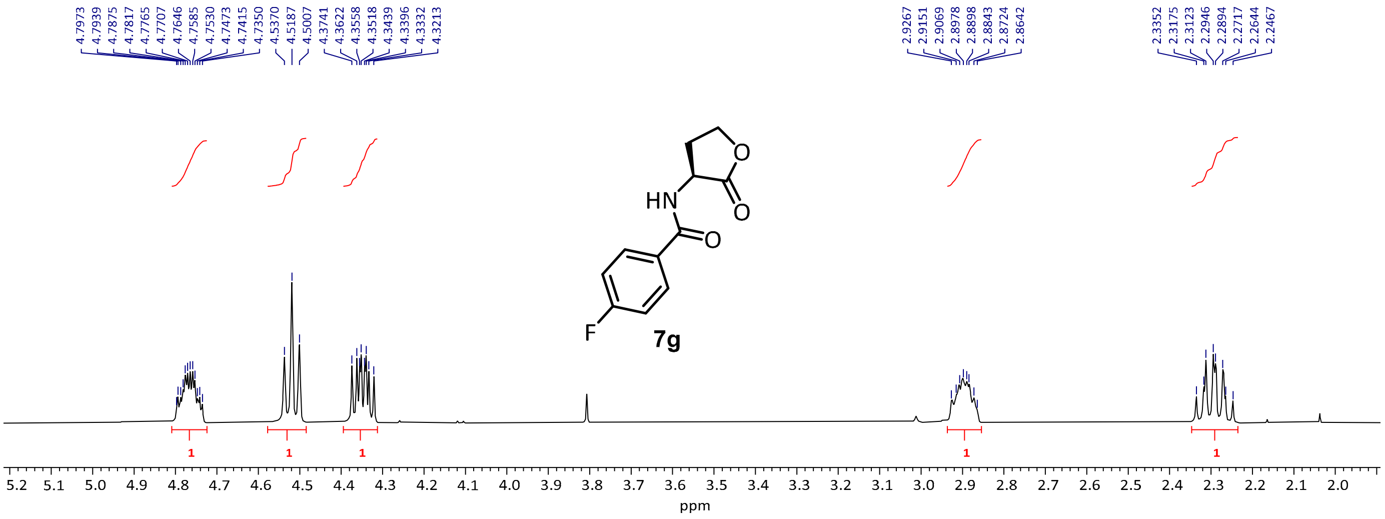

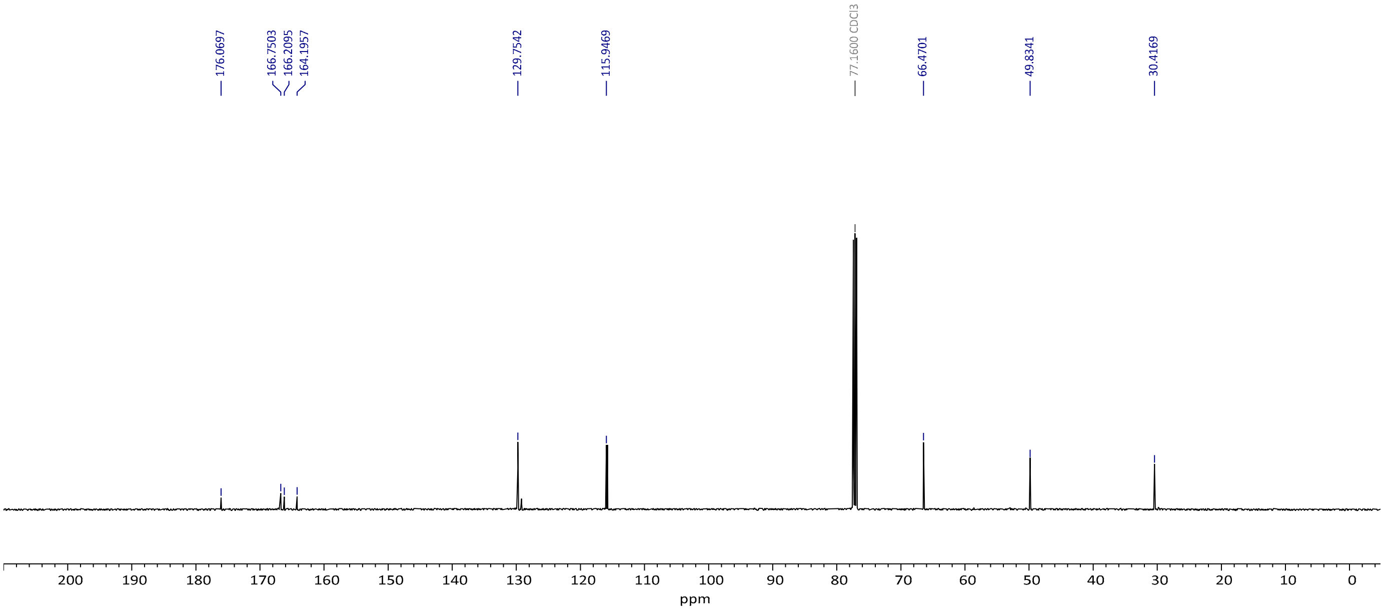

Compound **7h**, ^{1}H and ^{13}C NMR at 26 °C in $(CD_3)_2CO$

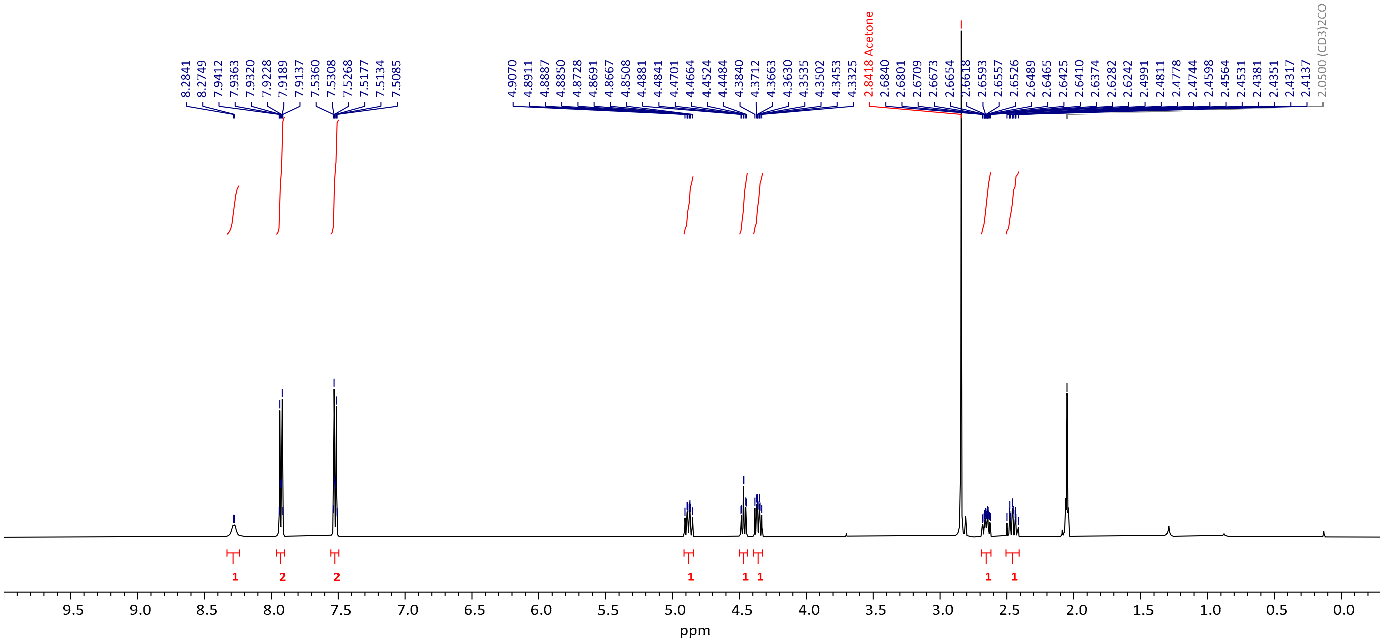

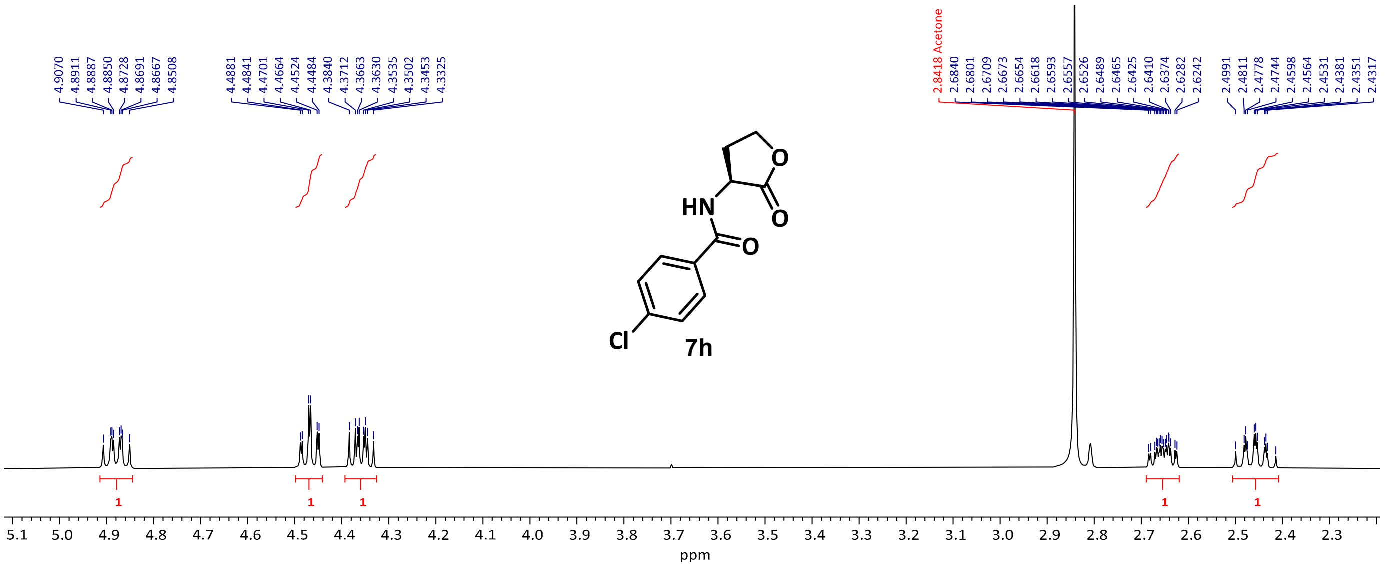

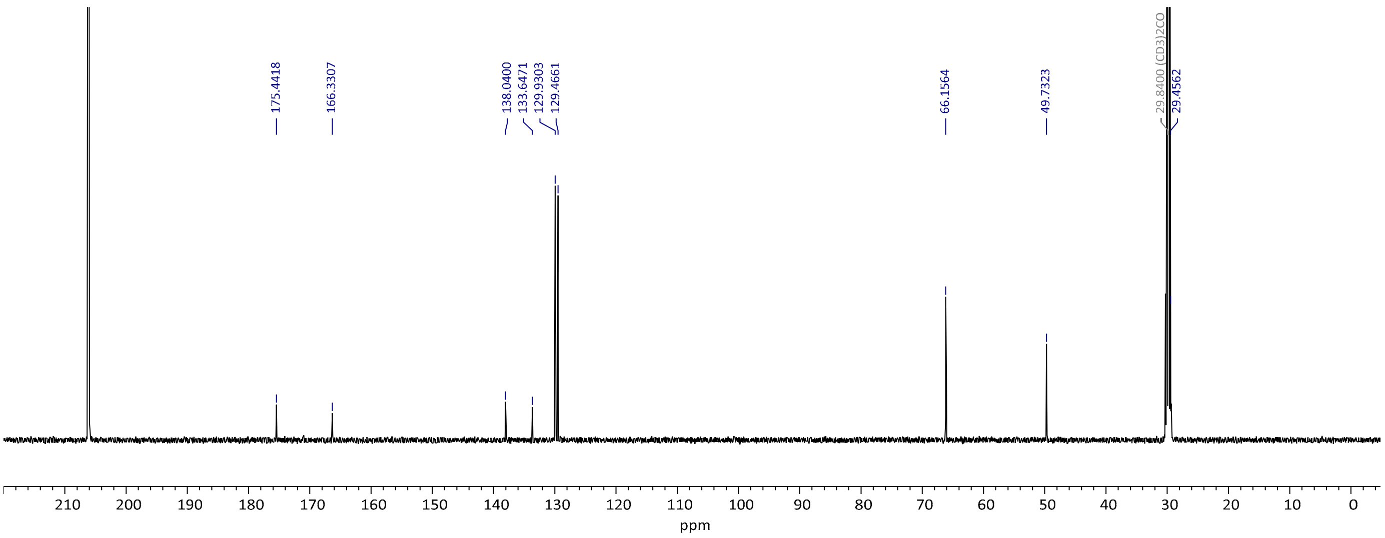

Compound **7i**, ^{1}H and ^{13}C NMR at 26 °C in $(CD_3)_2CO$

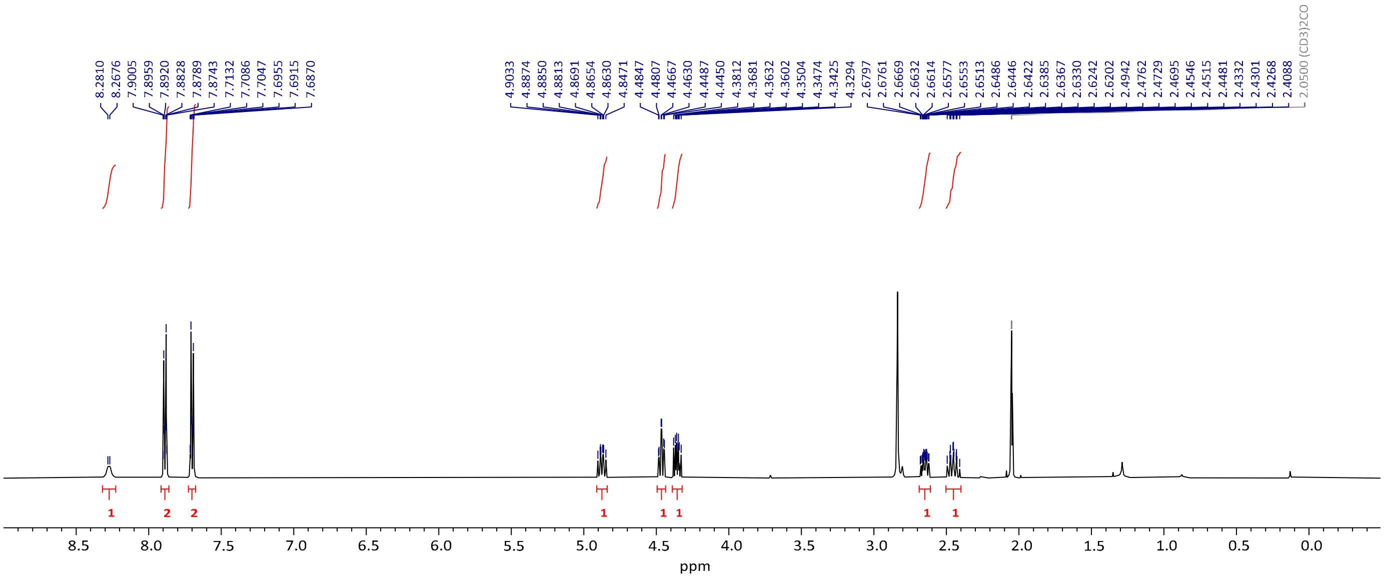

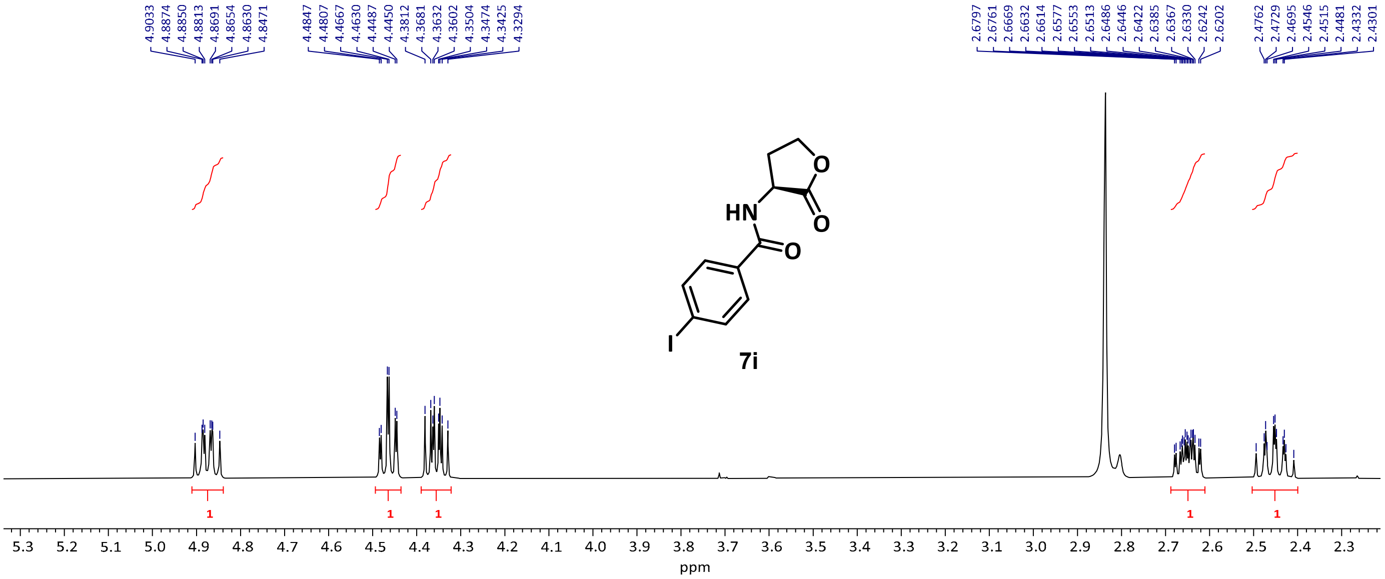

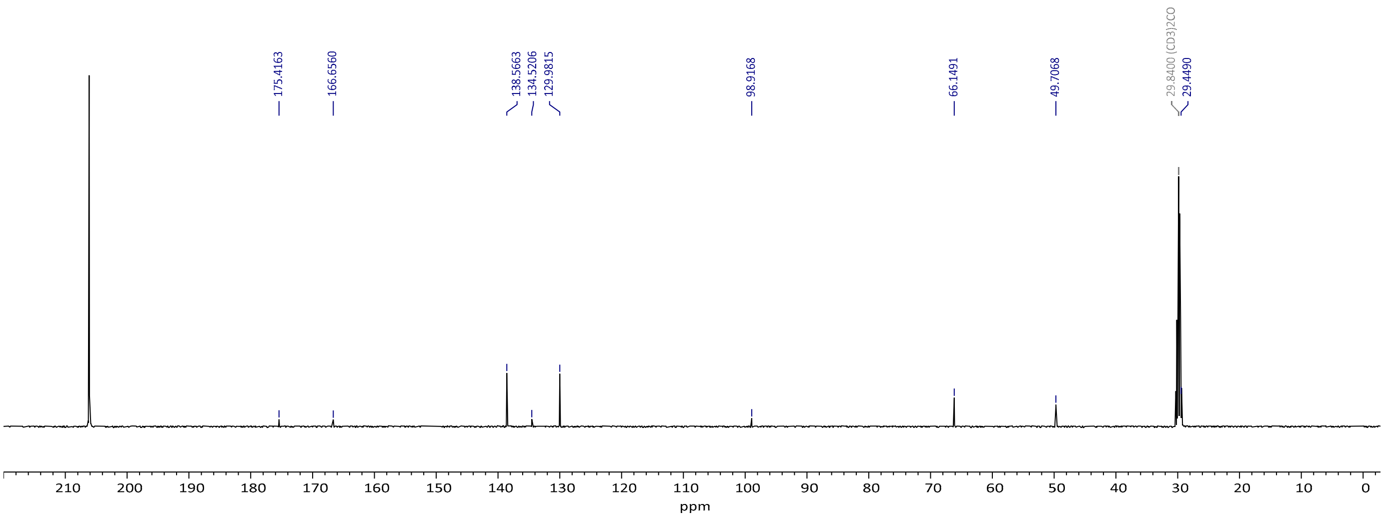

Compound **7j**, ^{1}H and ^{13}C NMR at 26 °C in $(CD_3)_2CO$

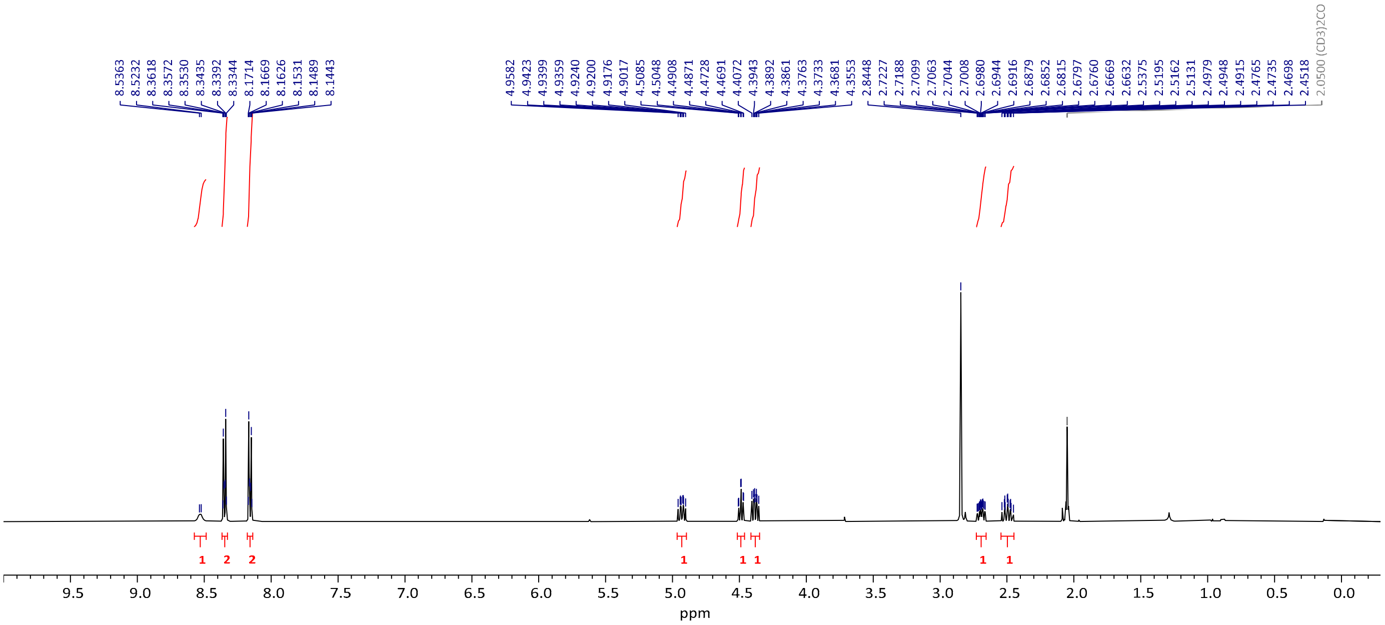
8.5363
8.5232
8.3618
8.3572
8.3530
8.3435
8.3392
8.3344
8.1714
8.1669
8.1626
8.1531
8.1489
8.1443
4.9582
4.9423
4.9399
4.9359
4.9240
4.9200
4.9176
4.9017
4.5085
4.5048
4.4908
4.4871
4.4728
4.4691
4.4072
4.3943
4.3892
4.3861
4.3763
4.3733
4.3681
4.3553
2.8448
2.7227
2.7188
2.7099
2.7063
2.7044
2.7008
2.6980
2.6944
2.6916
2.6879
2.6852
2.6815
2.6797
2.6760
2.6669
2.6632
2.5375
2.5195
2.5162
2.5131
2.4979
2.4948
2.4915
2.4765
2.4735
2.4698
2.4518
2.0500 (CD3)2CO
ppm

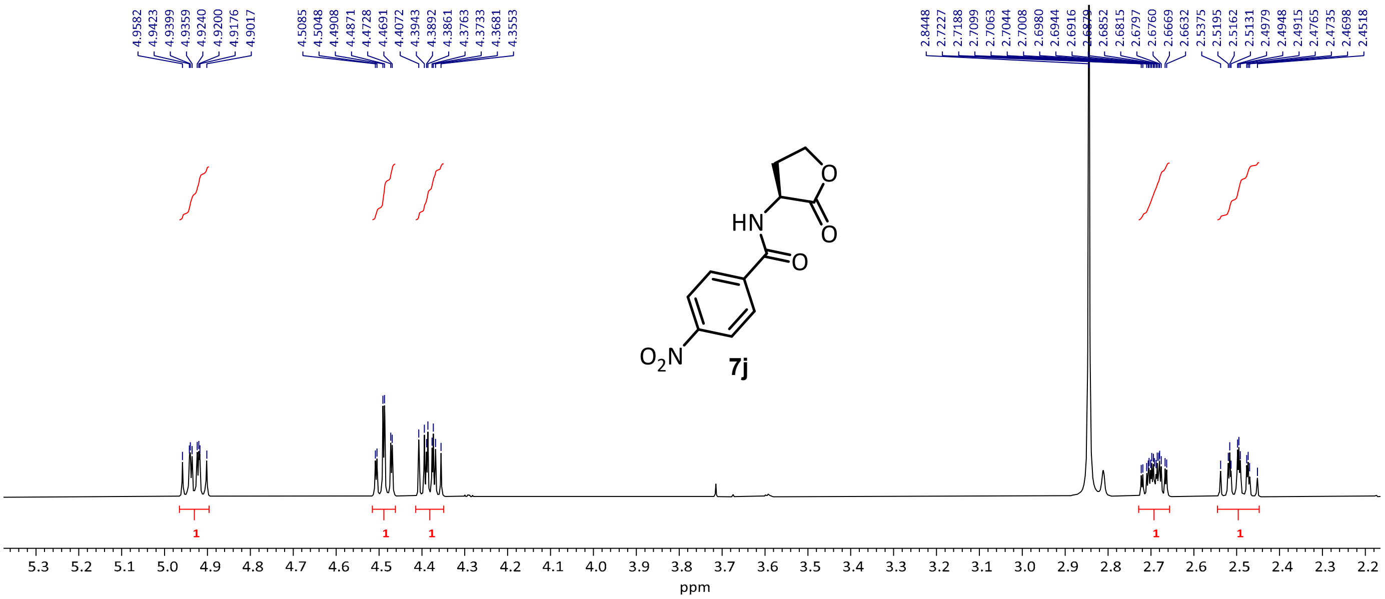
HN
O
O
O
O_2N
7j
ppm

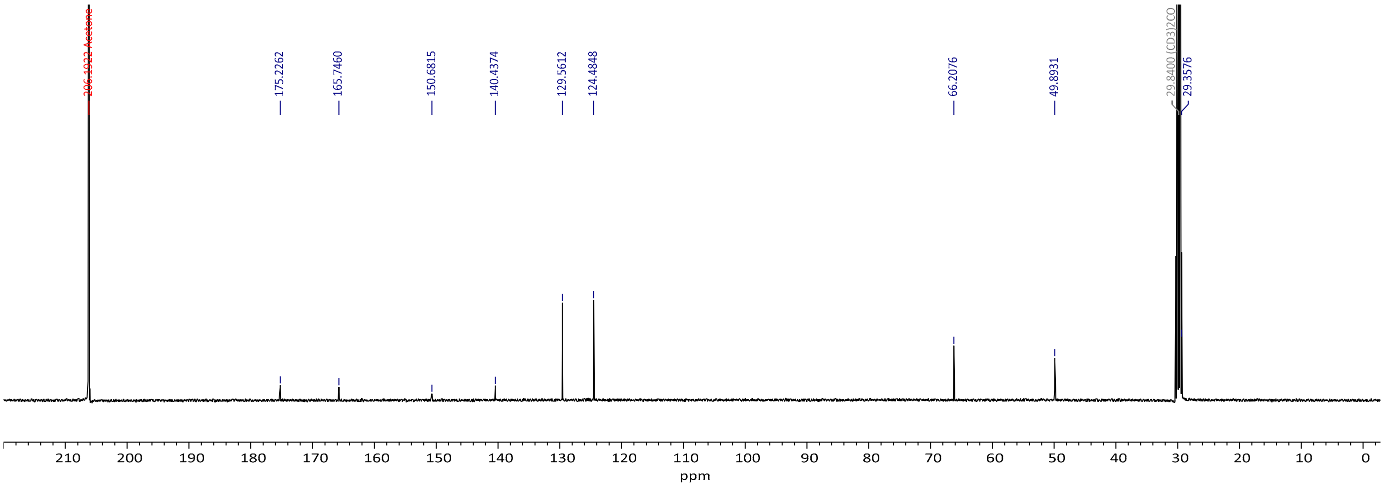
206.1922 Acetone
175.2262
165.7460
150.6815
140.4374
129.5612
124.4848
66.2076
49.8931
29.8400 (CD3)2CO
29.3576
ppm

Compound **7k**, ^{1}H and ^{13}C NMR at 26 °C in $(CD_3)_2CO$

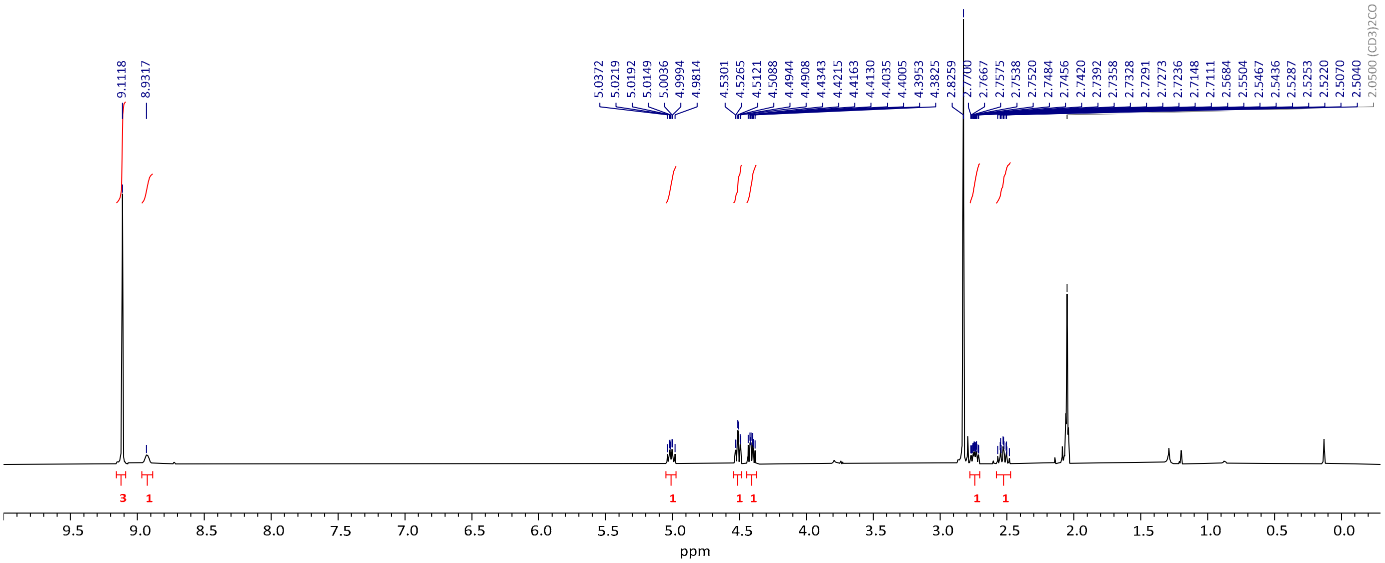

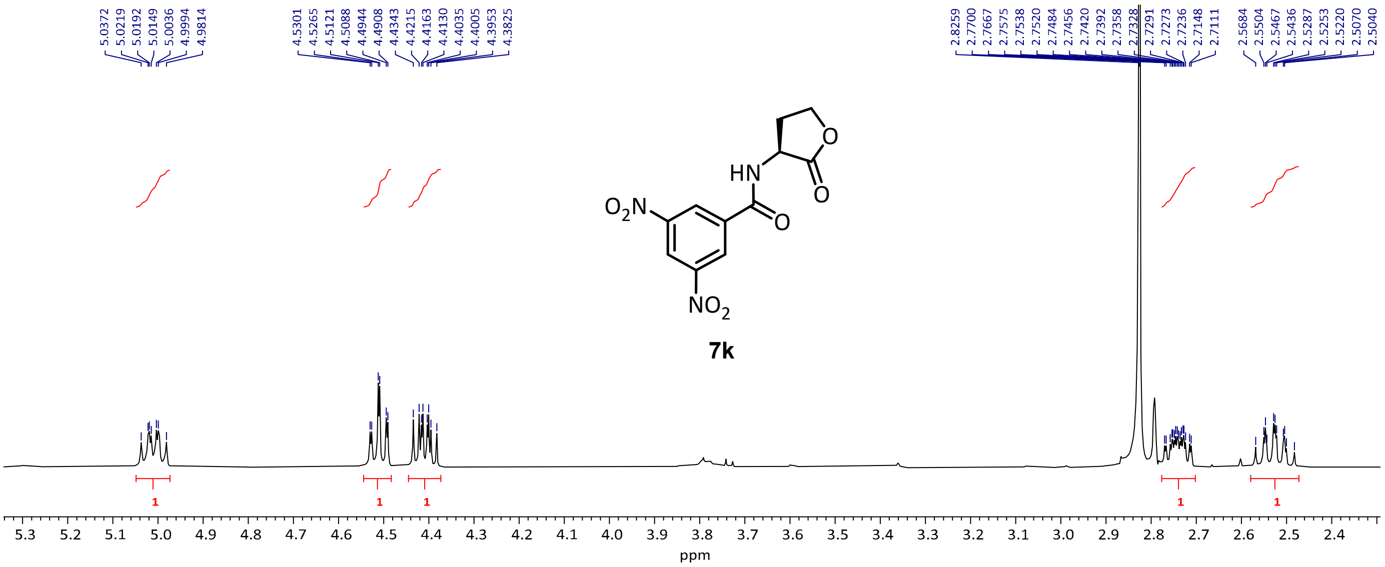

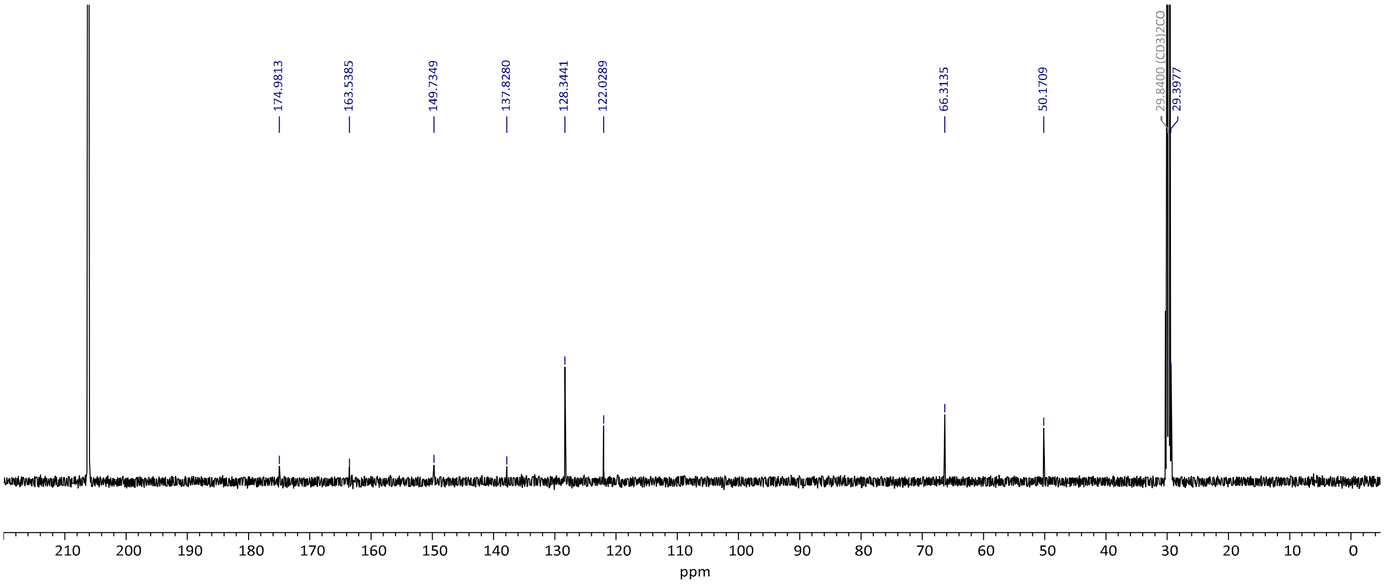

Compound **7l**, ^{1}H and ^{13}C NMR at 26 °C in $CDCl_3$

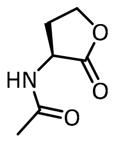

7l

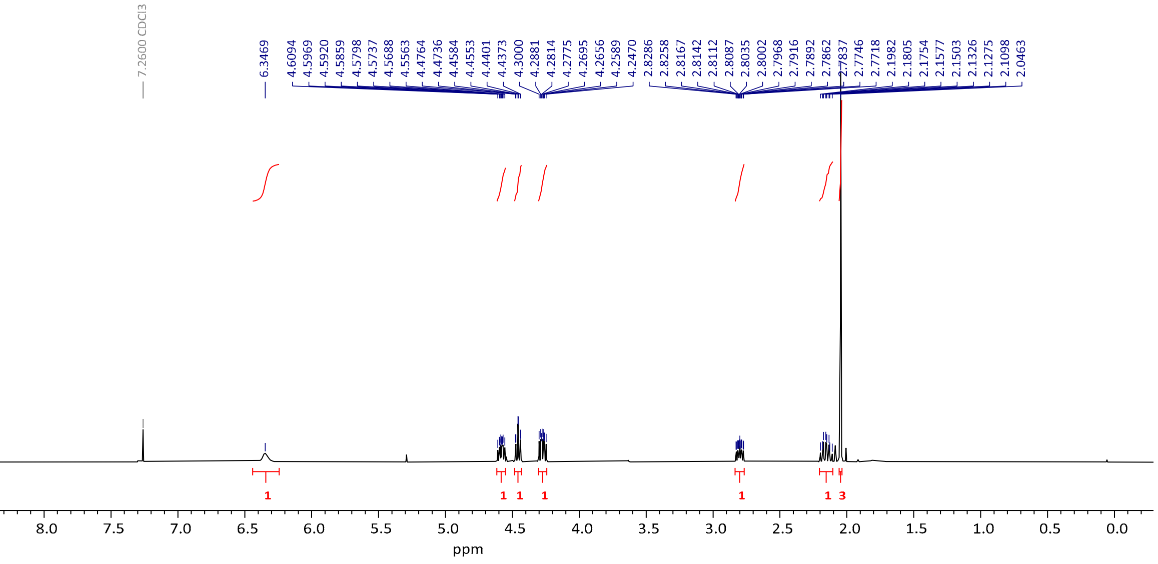

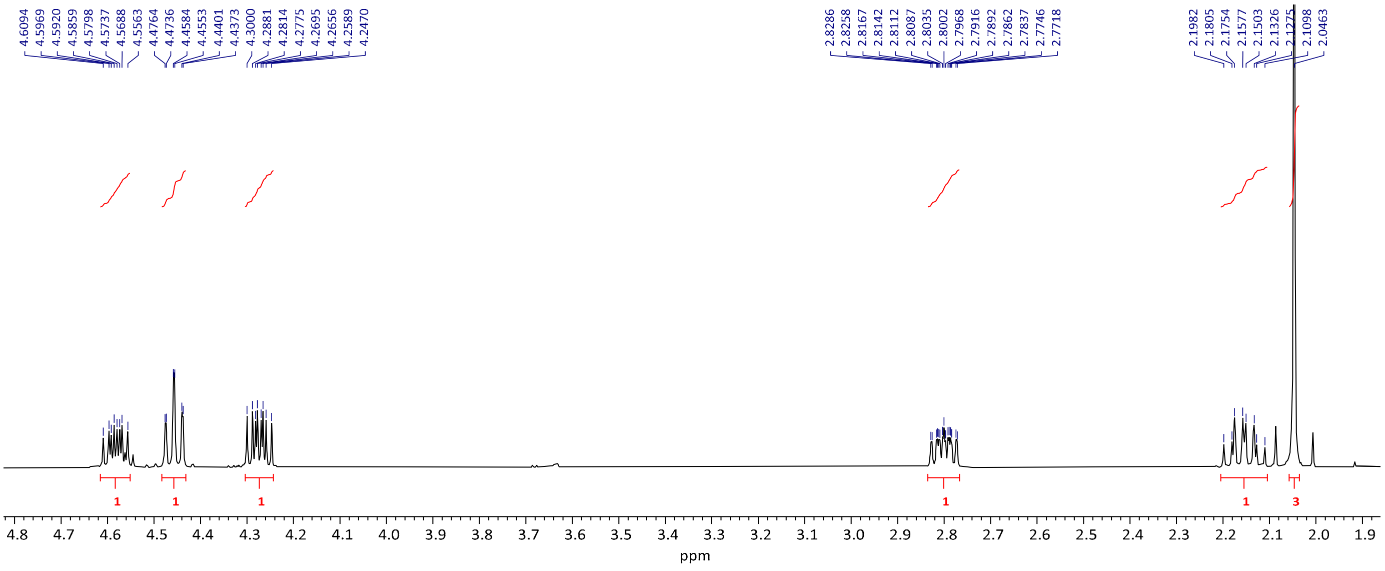

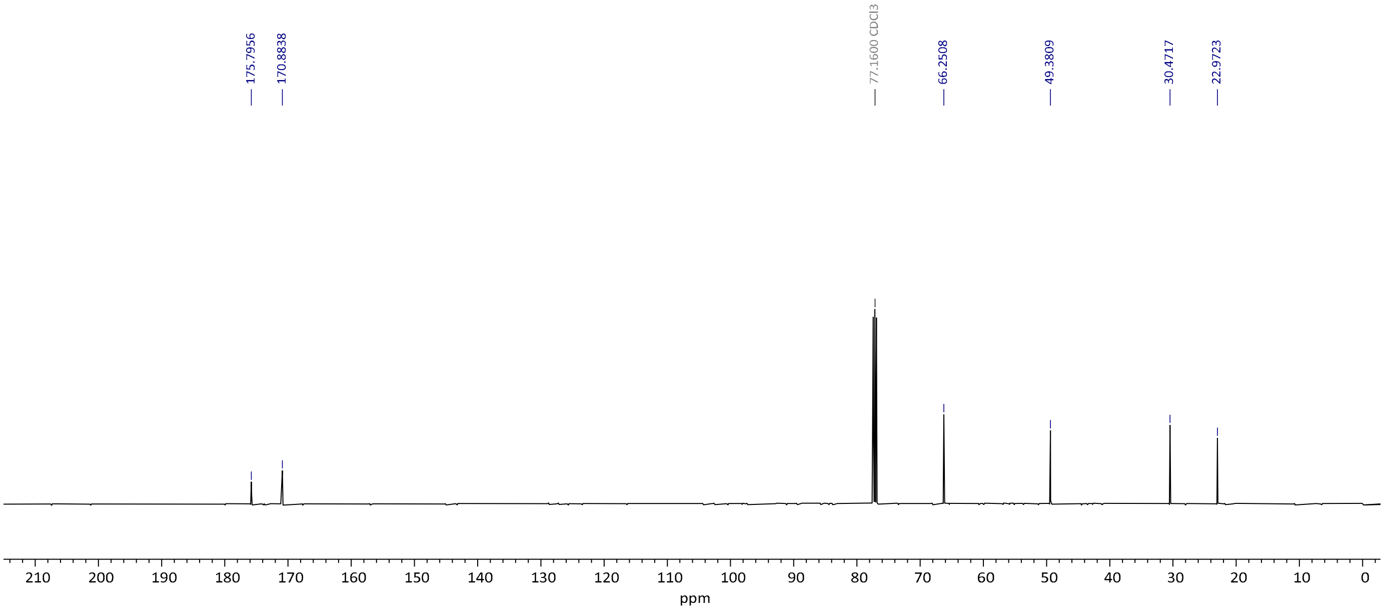

Compound **7m**, ^{1}H and ^{13}C NMR at 70 °C in CD3CN

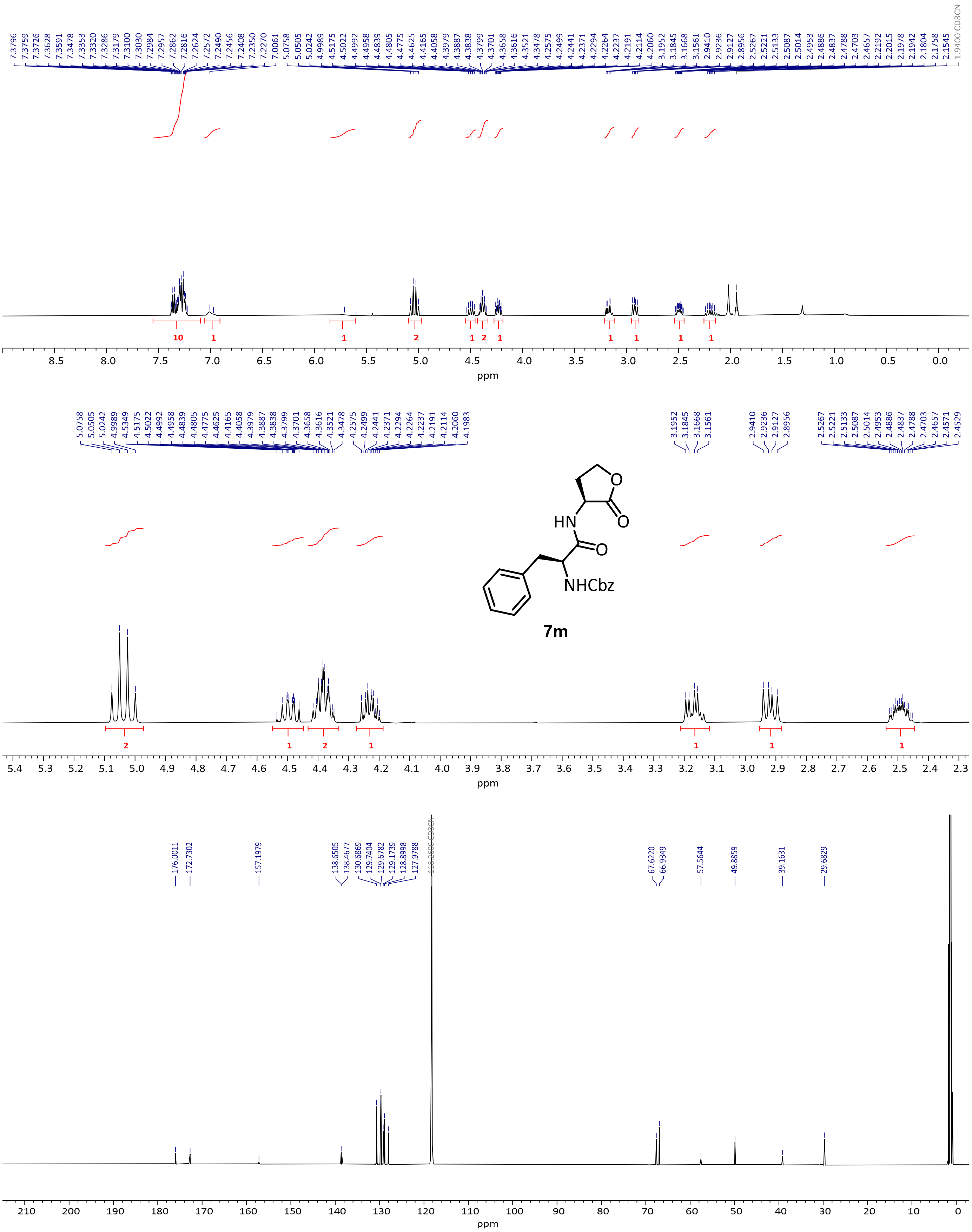

Compound **7n**, ^{1}H and ^{13}C NMR at 26 °C in $CDCl_3$

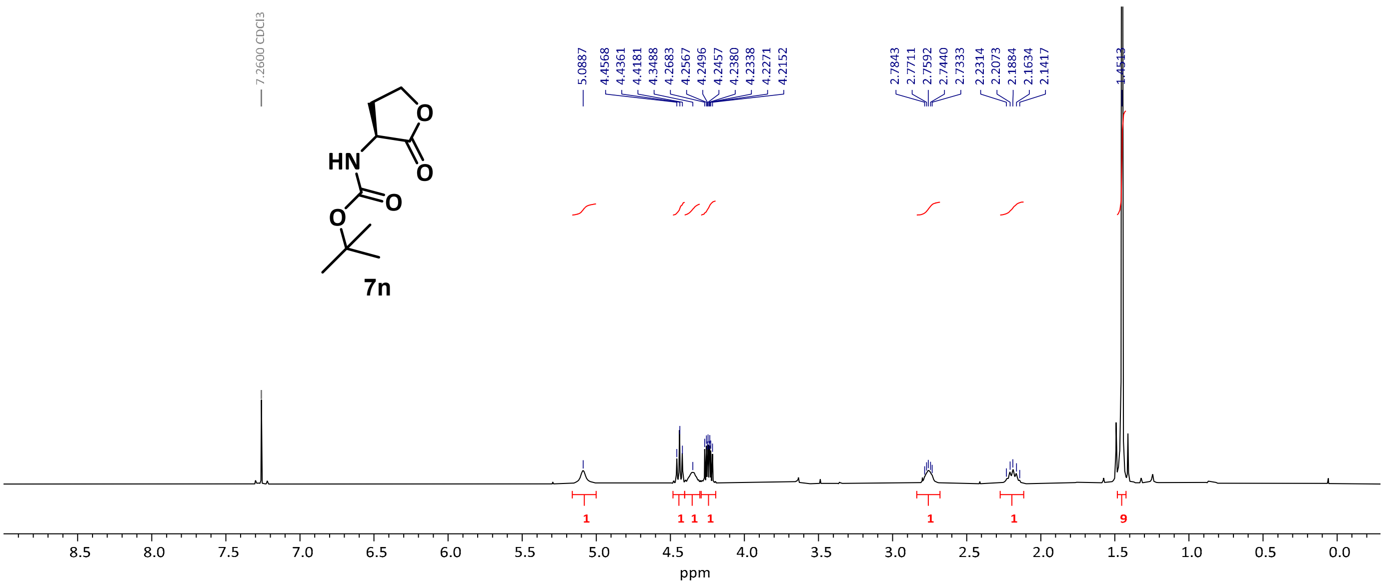

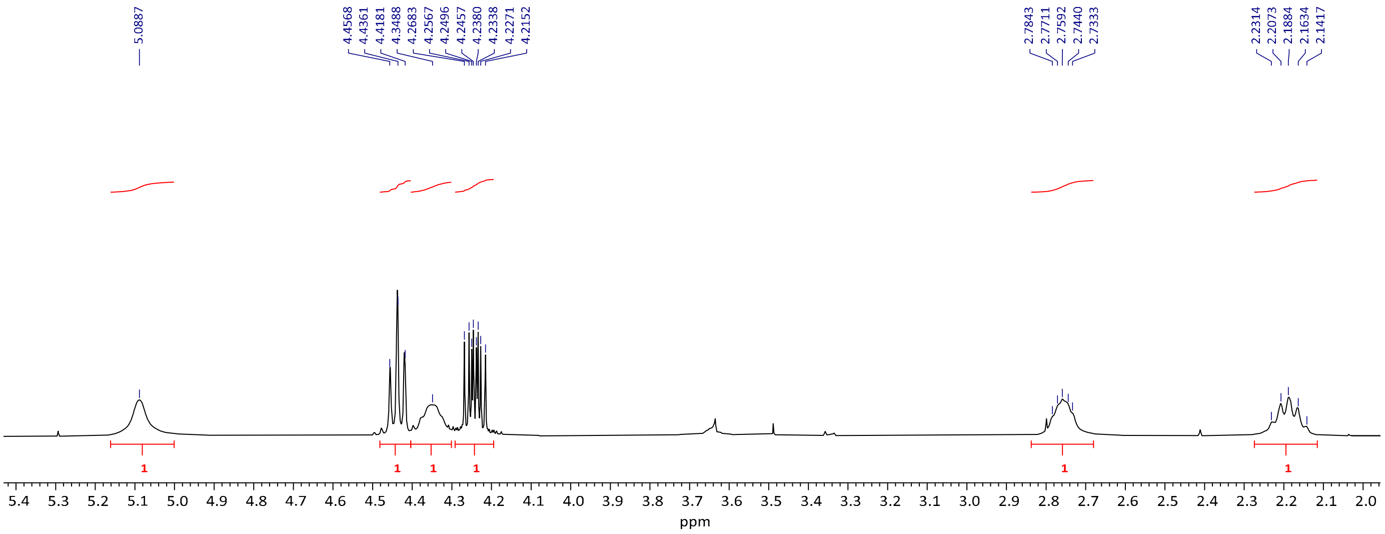

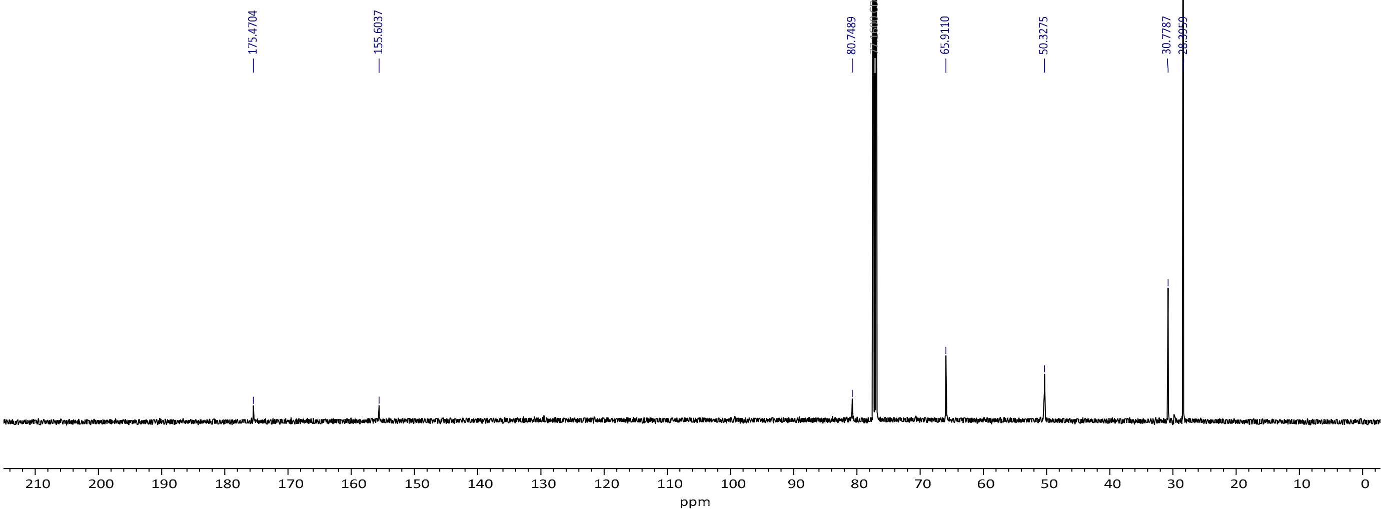

Compound **7o**, ^{1}H and ^{13}C NMR at 26 °C in $CDCl_3$

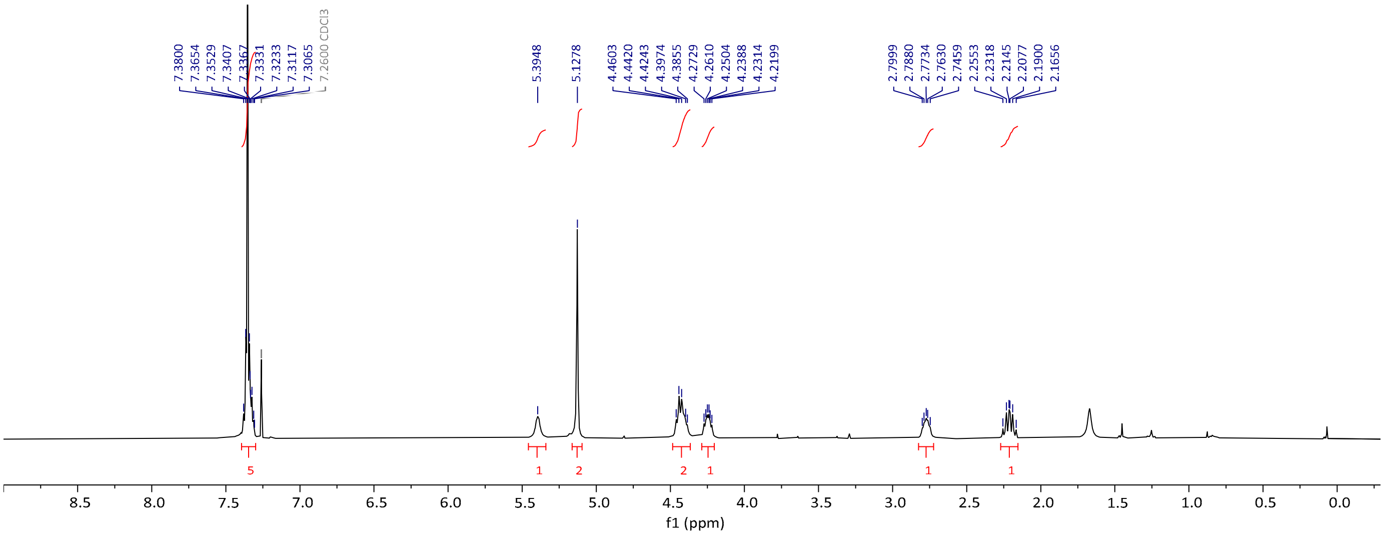

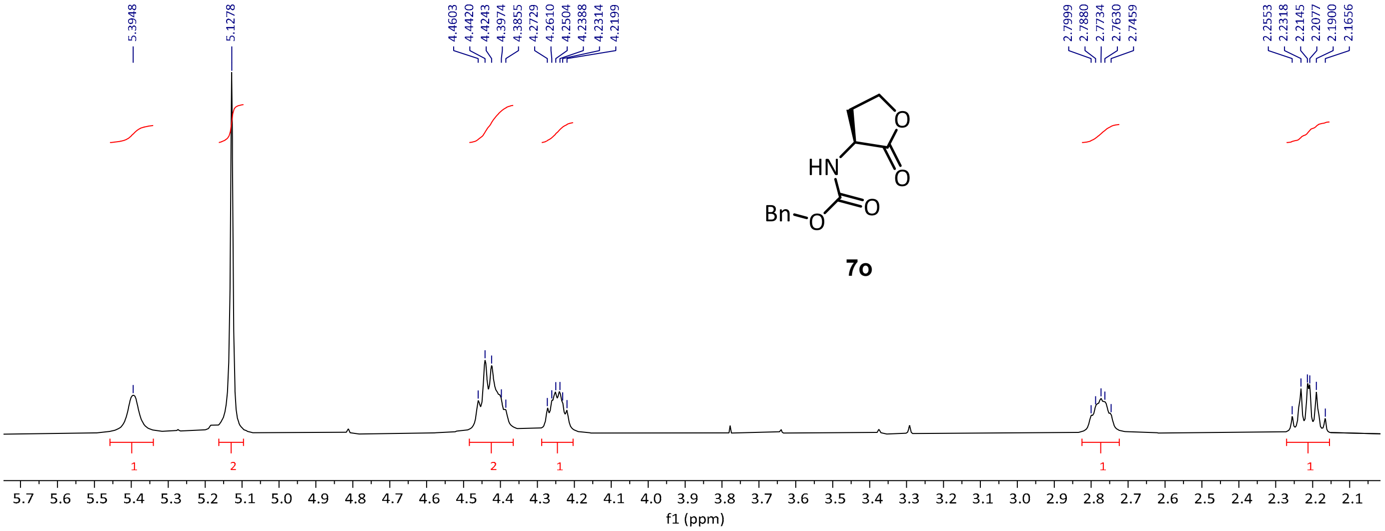

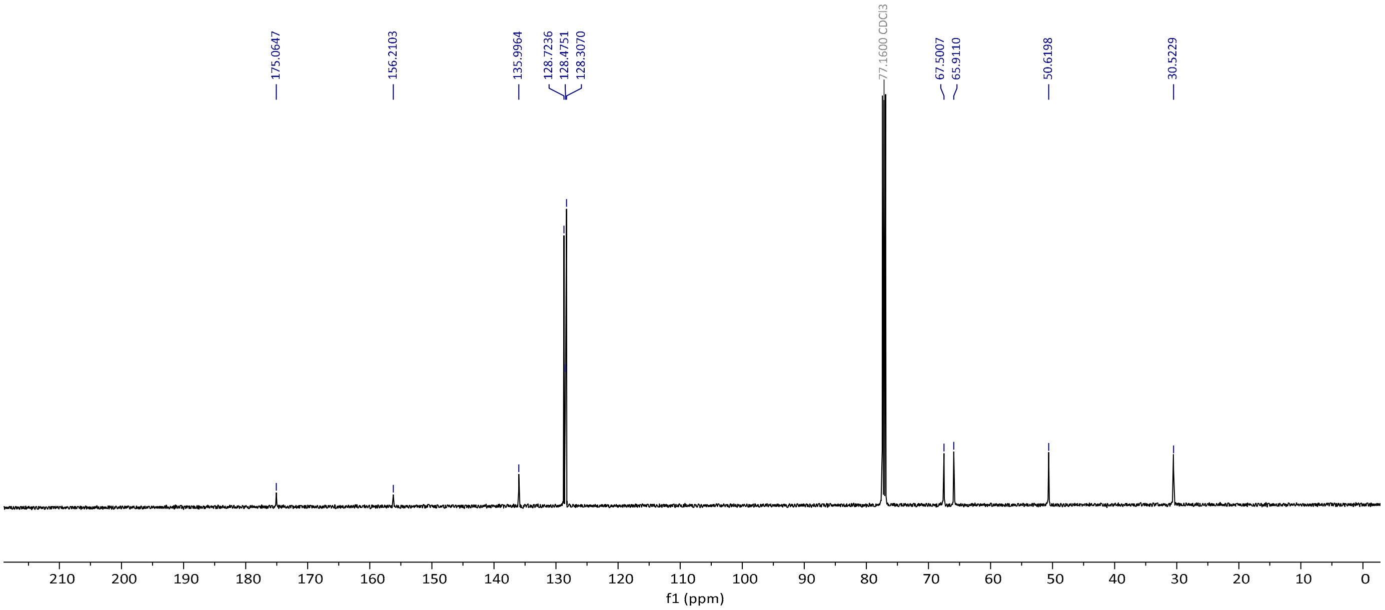

Compound **7p**, ^{1}H and ^{13}C NMR at 26 °C in $CDCl_3$

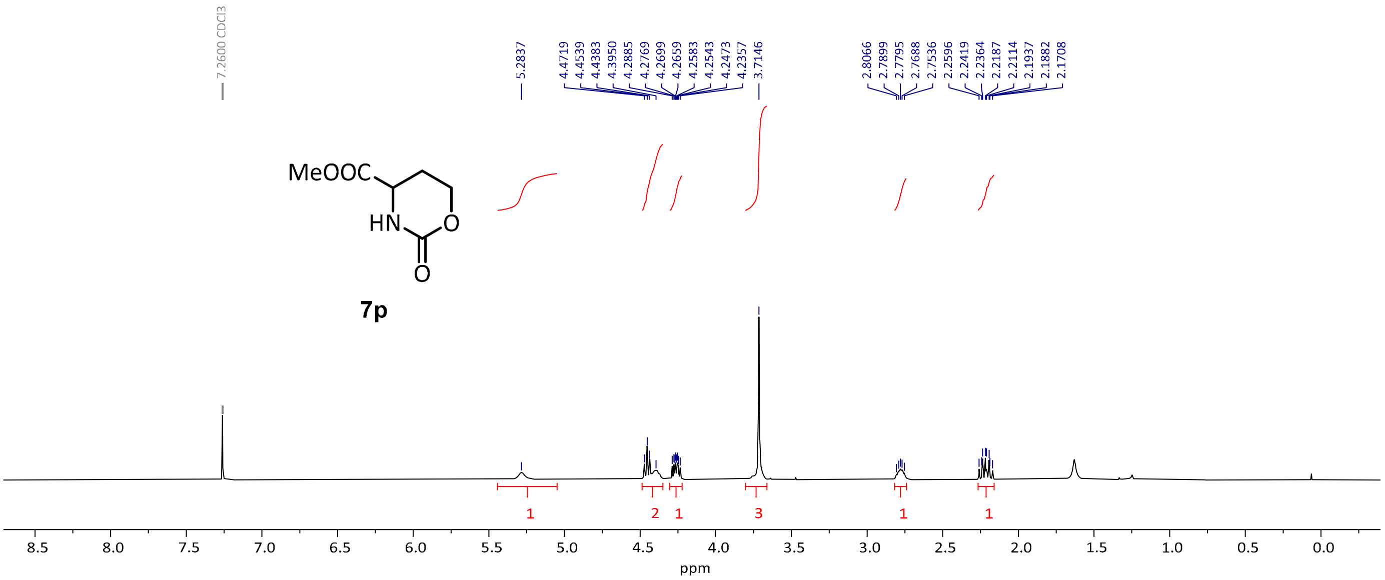

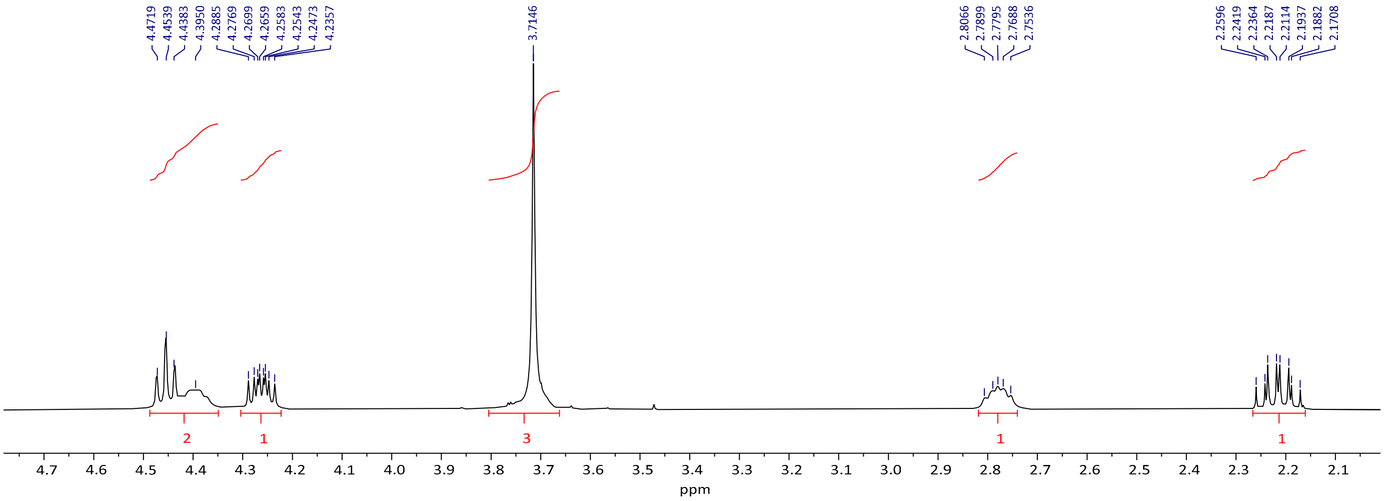

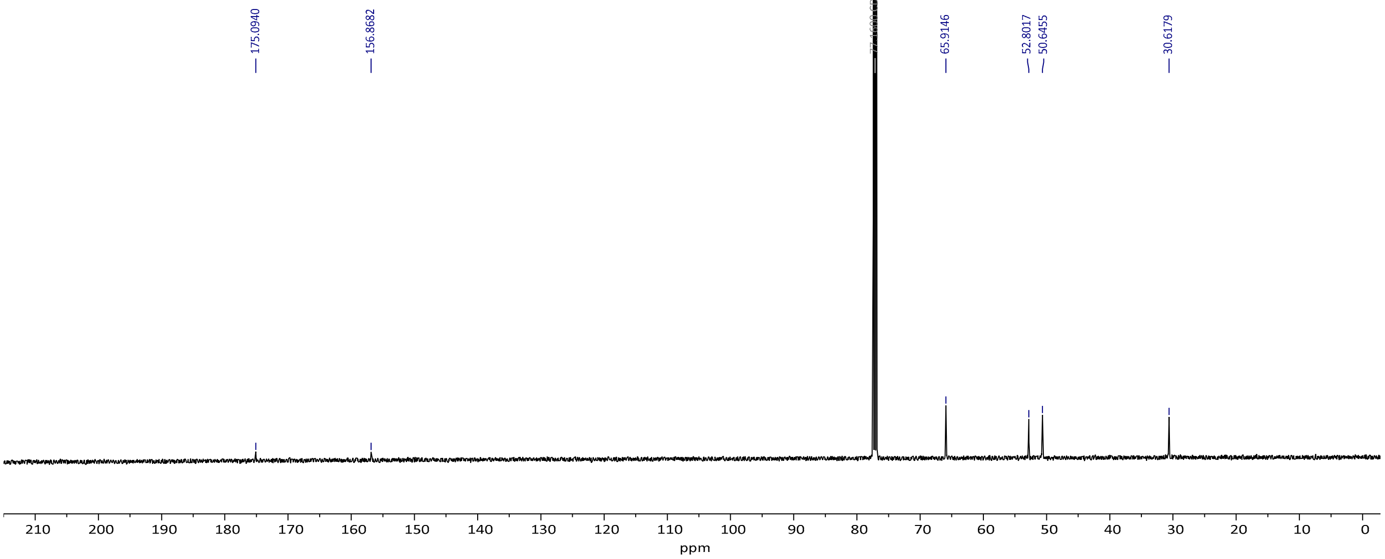

Compound **7q**, ¹H and ¹³C NMR at 26 °C in $CDCl_3$

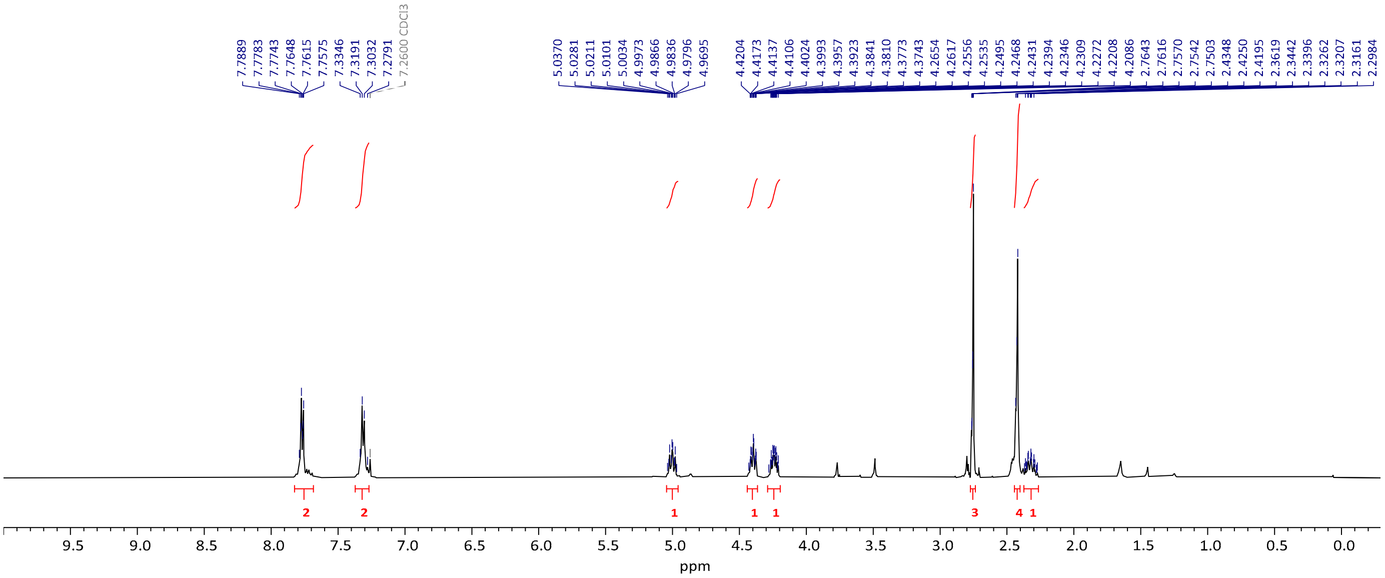

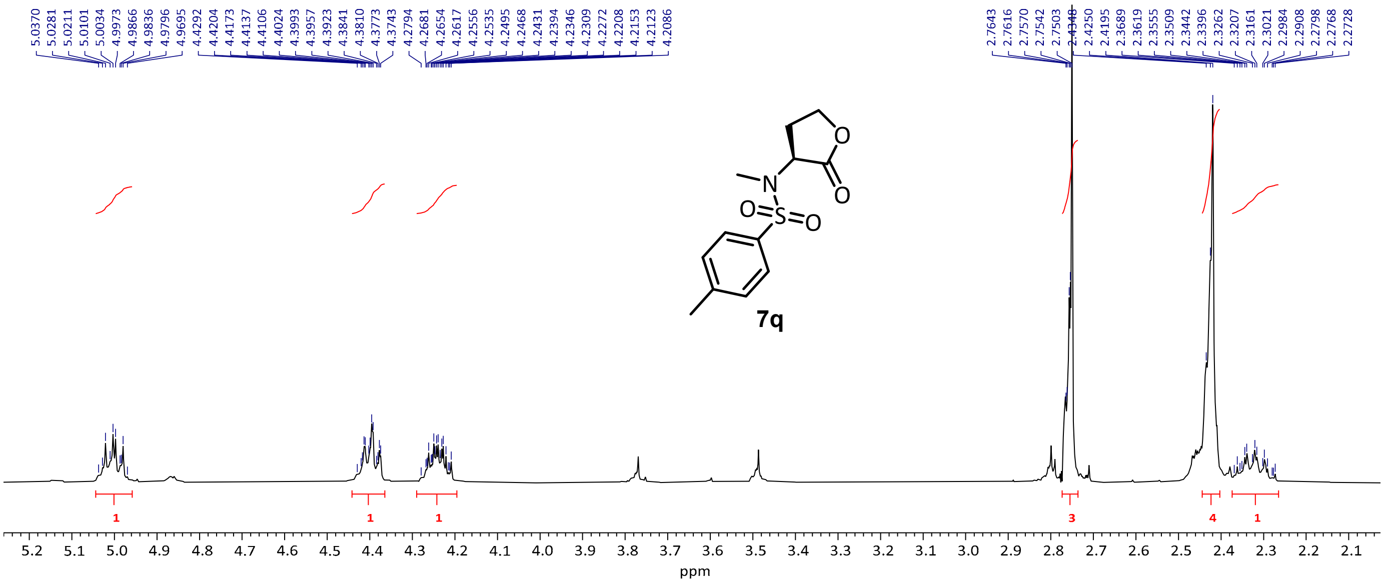

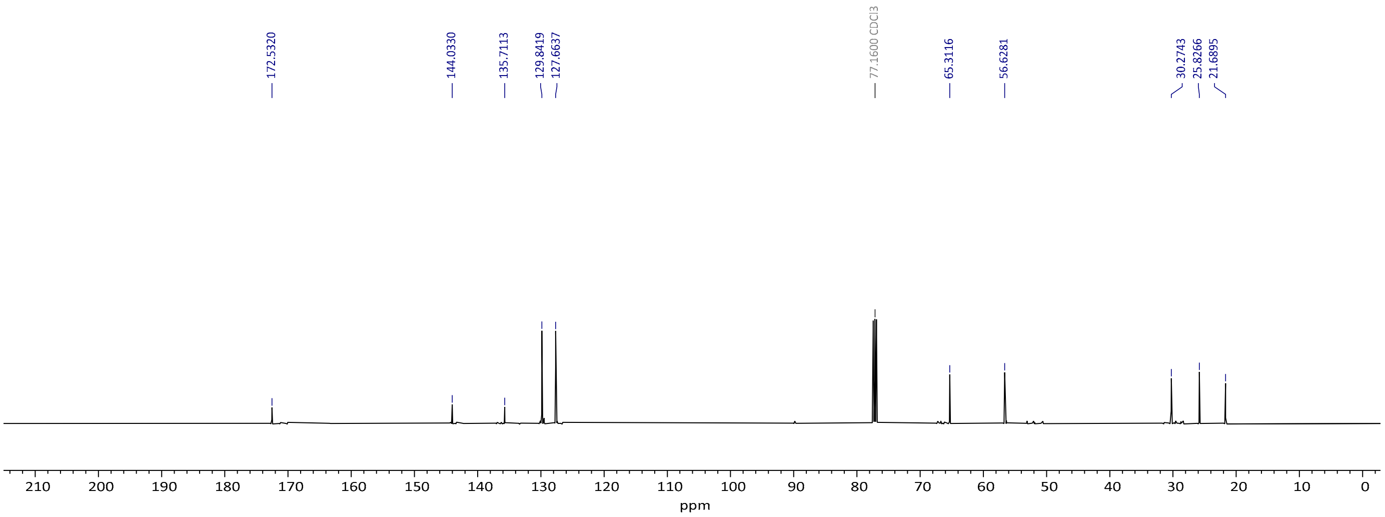

Compound **7r**, ^{1}H and ^{13}C NMR at 26 °C in $CDCl_3$

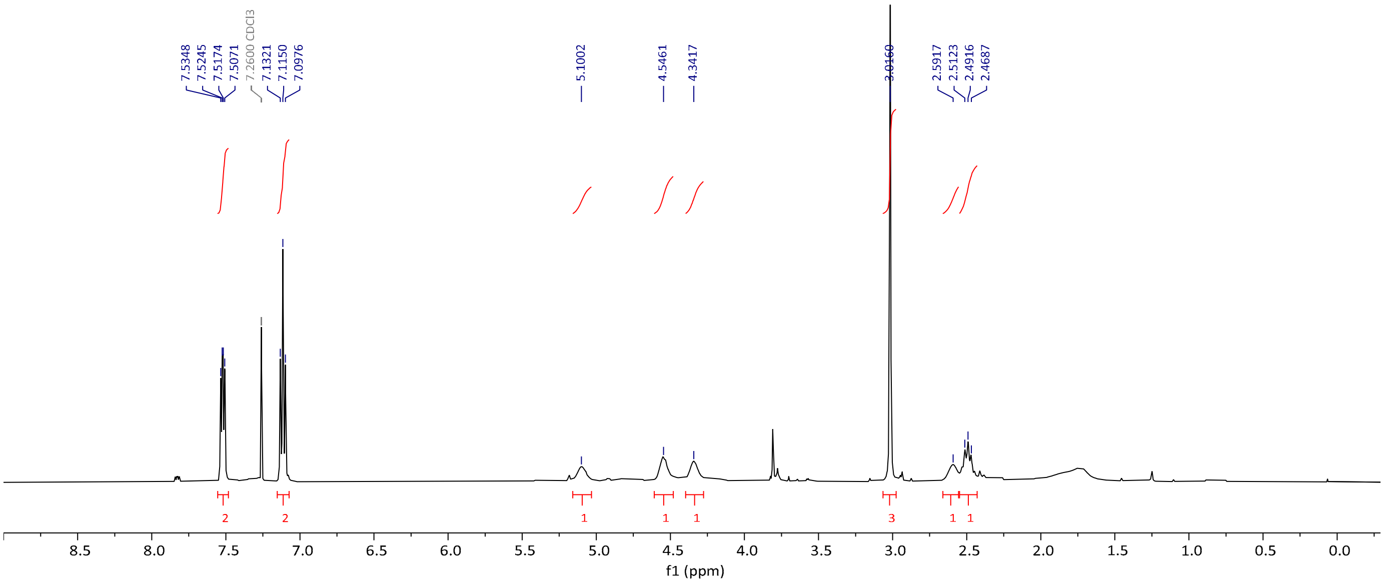

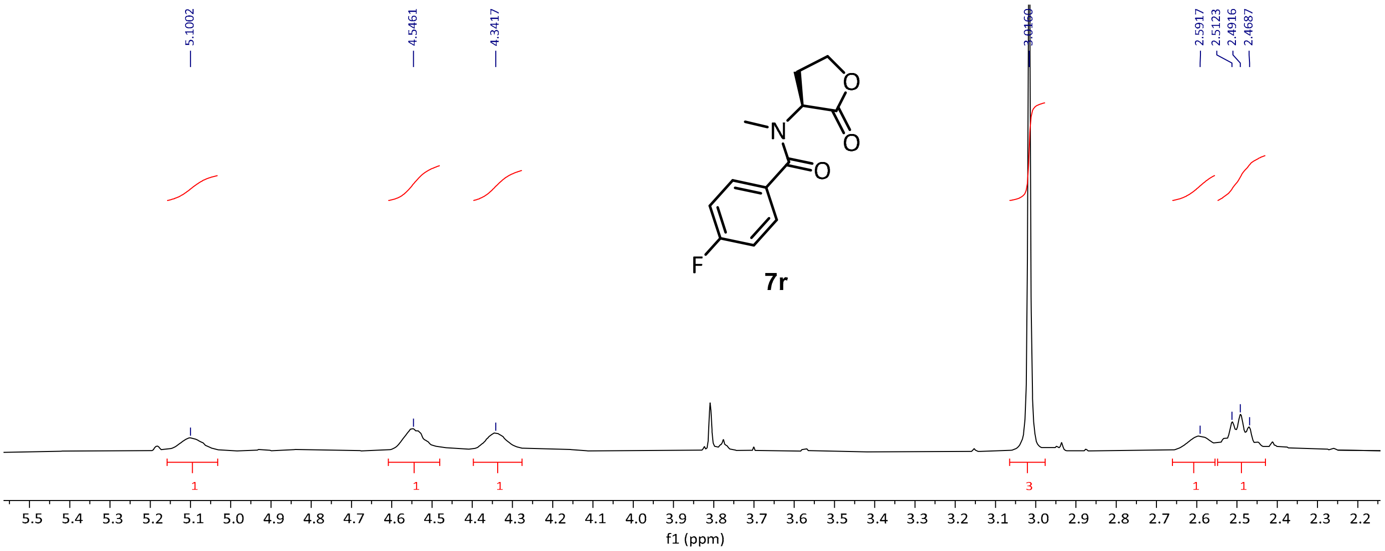

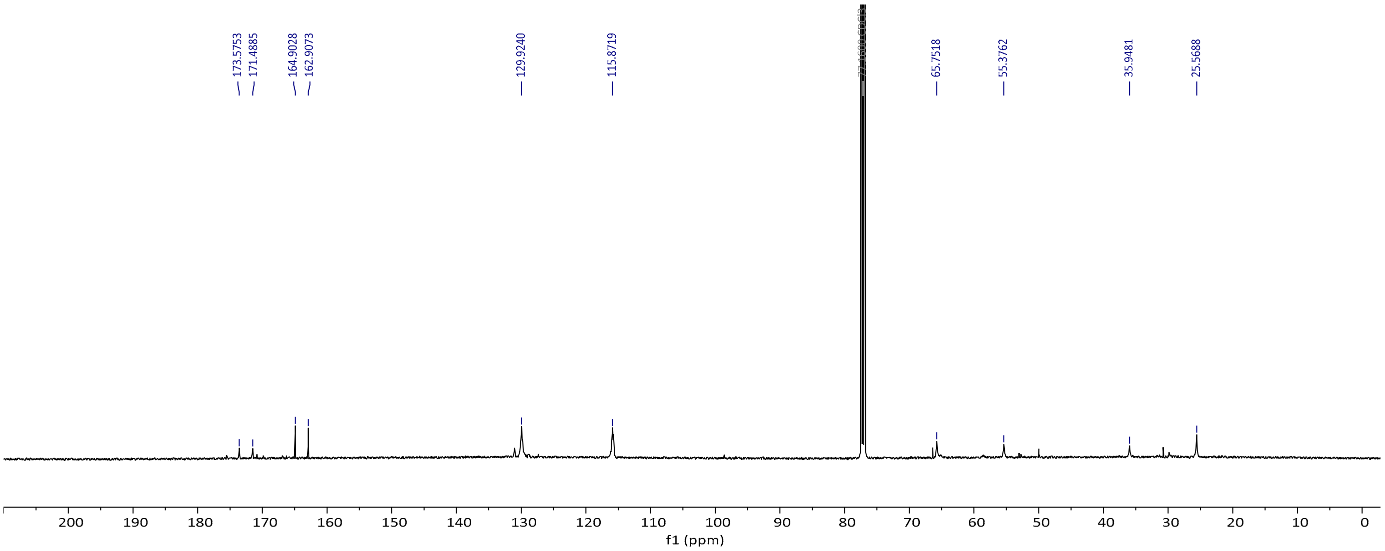

Compound **7s**, ^{1}H and ^{13}C NMR at 26 °C in $CDCl_3$

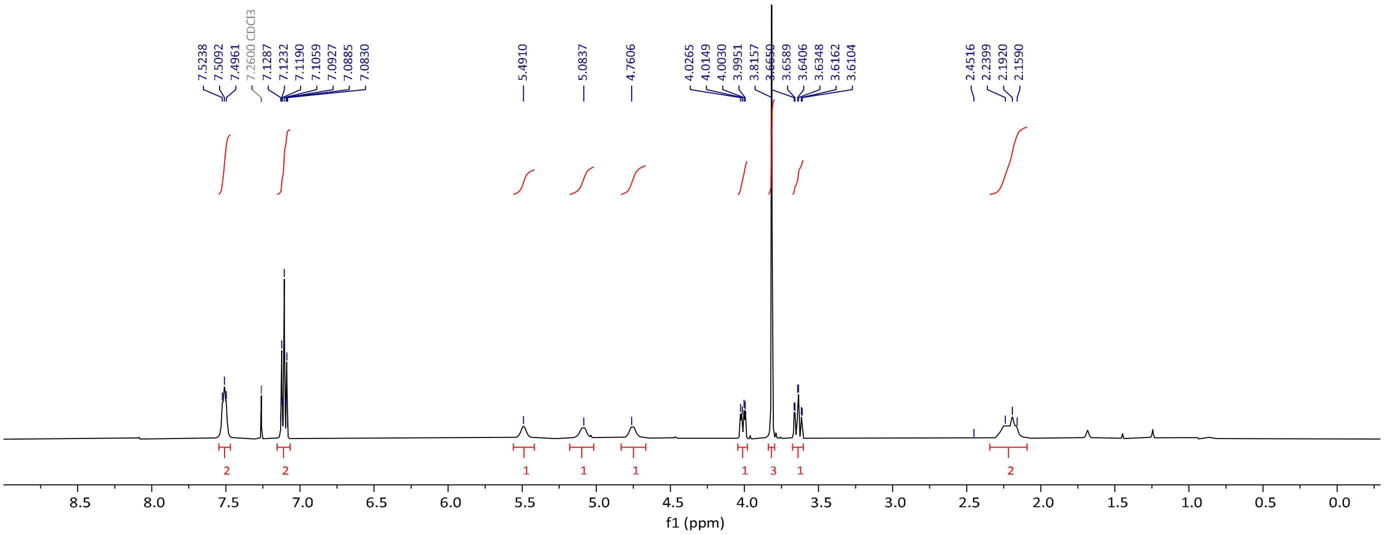

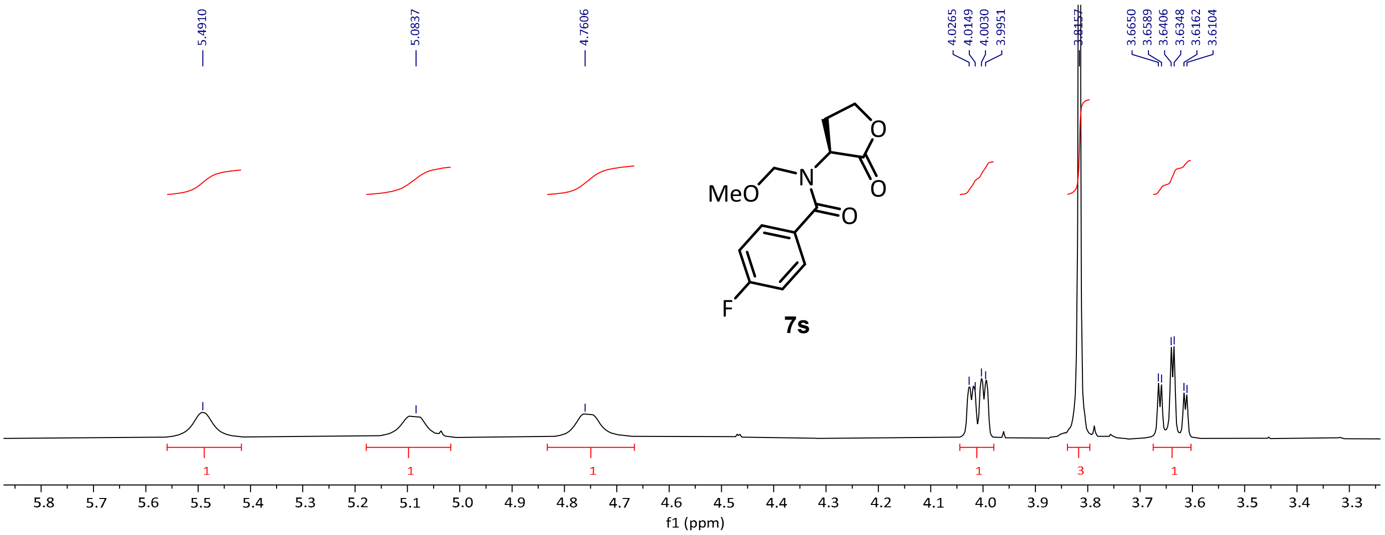

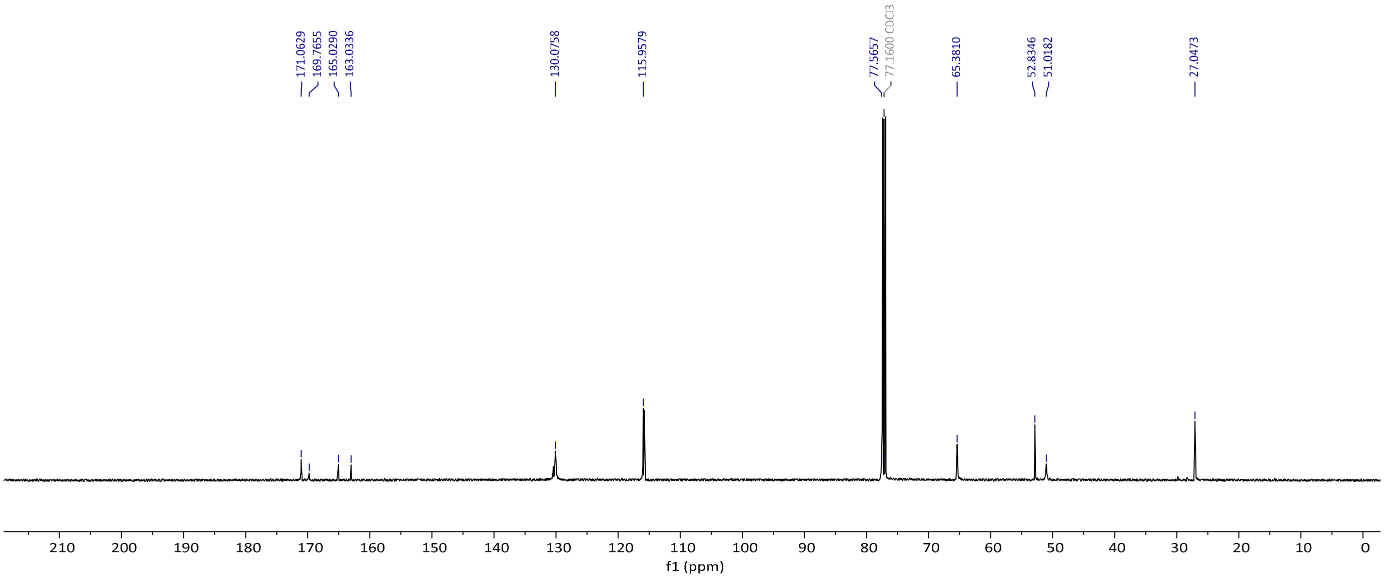

Compound **7t**, ^{1}H and ^{13}C NMR at 70 °C in CD_3CN

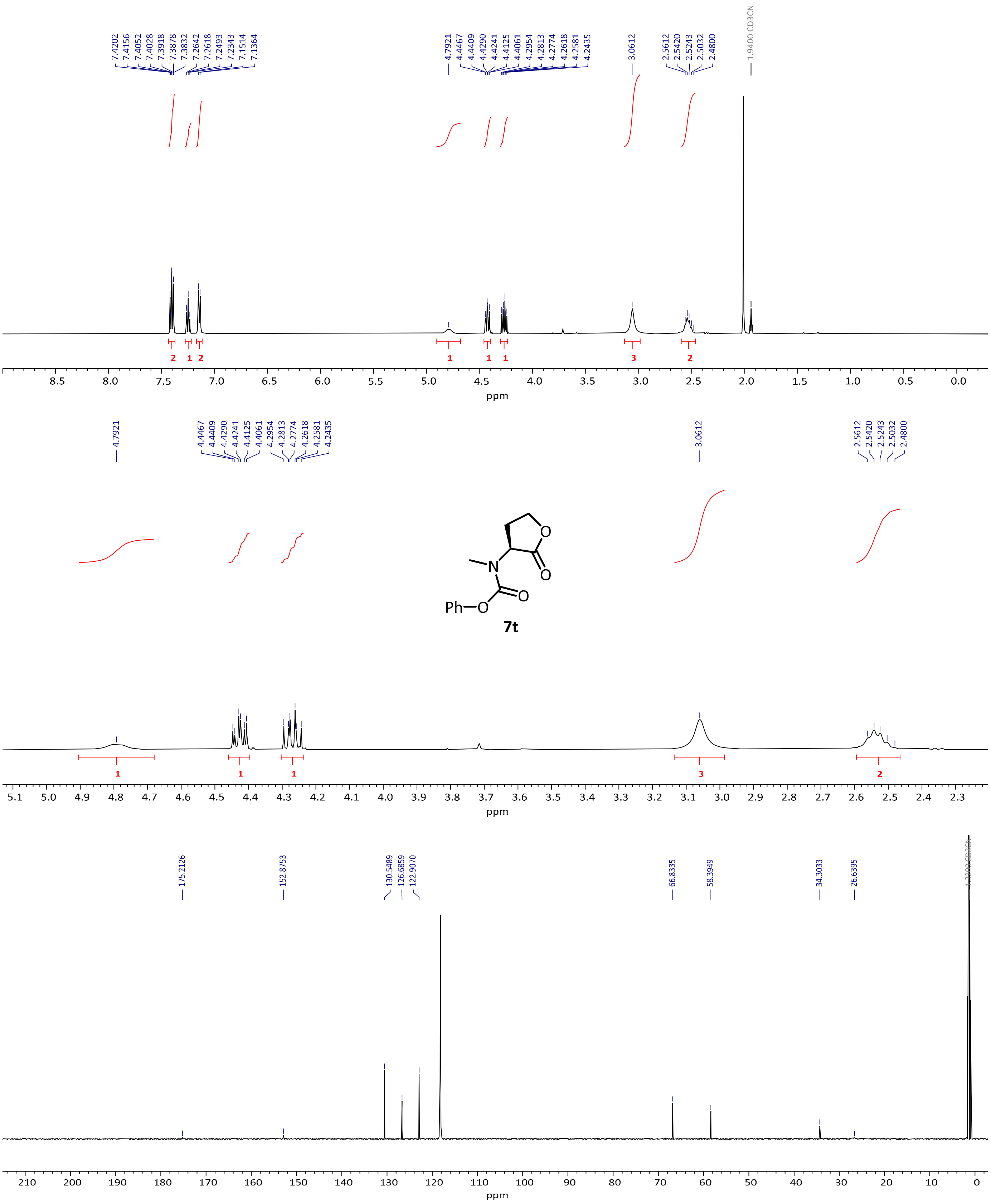

Compound **7u**, ^{1}H and ^{13}C NMR at 70 °C in CD_3CN

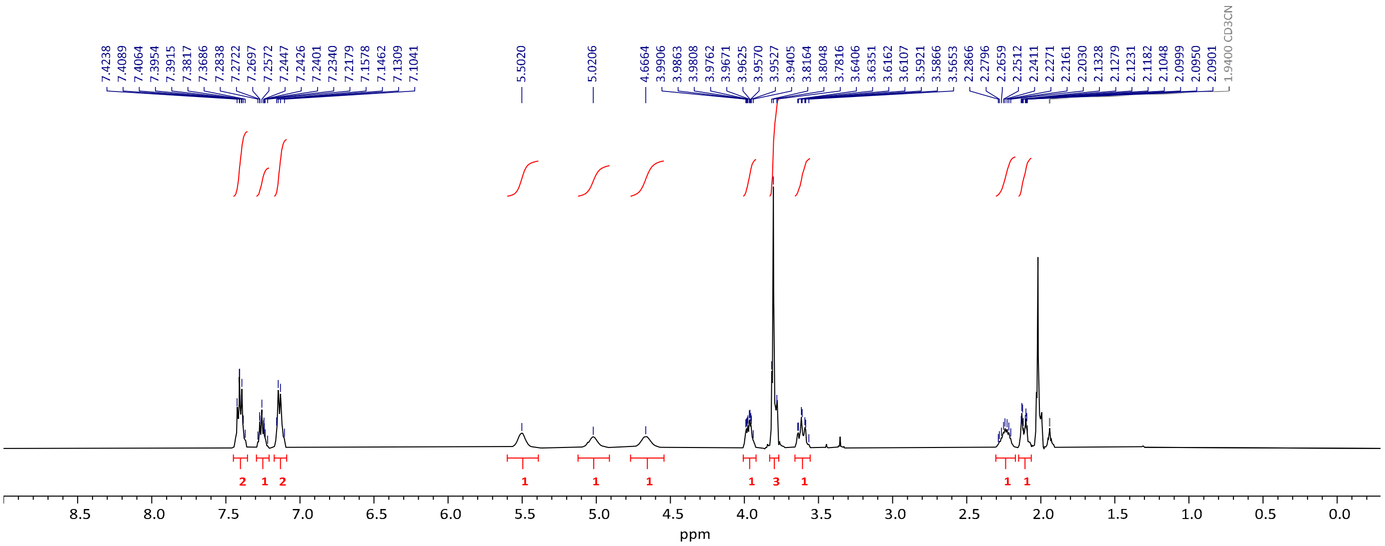
7.4238
7.4089
7.4064
7.3954
7.3915
7.3817
7.3686
7.2838
7.2722
7.2697
7.2572
7.2447
7.2426
7.2401
7.2340
7.2179
7.1578
7.1462
7.1309
7.1041
5.5020
5.0206
4.6664
3.9906
3.9863
3.9808
3.9762
3.9671
3.9625
3.9570
3.9527
3.9405
3.8164
3.8048
3.7816
3.6406
3.6351
3.6162
3.6107
3.5921
3.5866
3.5653
2.2866
2.2796
2.2659
2.2512
2.2411
2.2271
2.2161
2.2030
2.1328
2.1279
2.1231
2.1182
2.1048
2.0999
2.0950
2.0901
1.9400 CD3CN
2 1 2 1 1 1 1 3 1 1 1
ppm

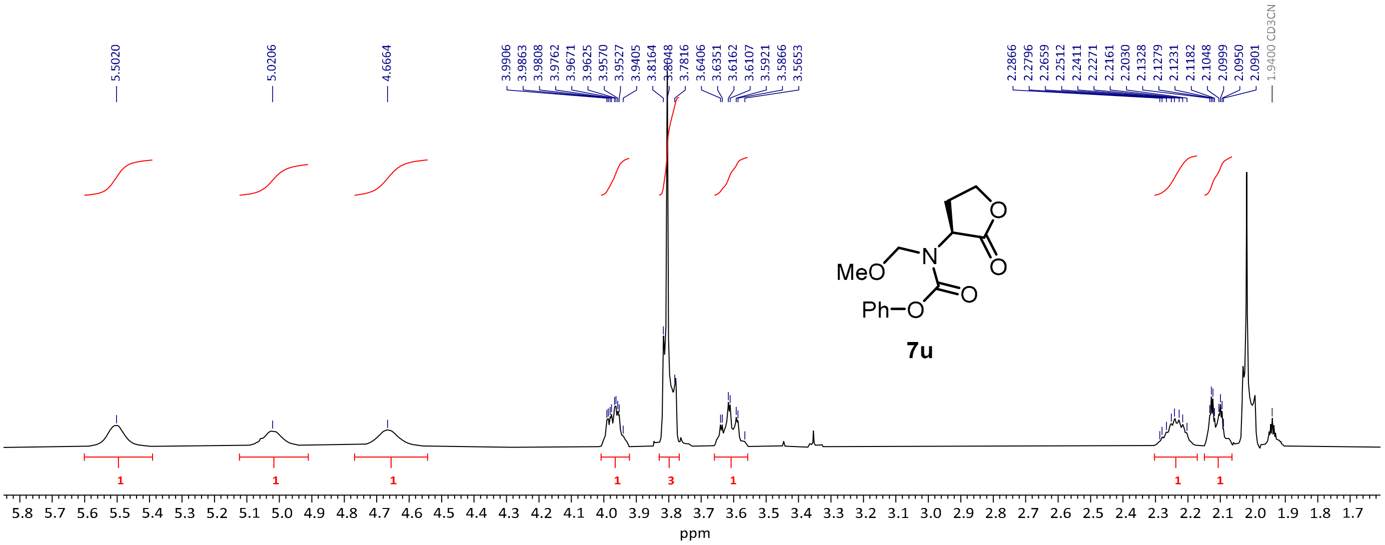
5.5020
5.0206
4.6664
3.9906
3.9863
3.9808
3.9762
3.9671
3.9625
3.9570
3.9527
3.9405
3.8164
3.8048
3.7816
3.6406
3.6351
3.6162
3.6107
3.5921
3.5866
3.5653
2.2866
2.2796
2.2659
2.2512
2.2411
2.2271
2.2161
2.2030
2.1328
2.1279
2.1231
2.1182
2.1048
2.0999
2.0950
2.0901
1.9400 CD3CN
MeO
N
O
O
Ph–O
O
7u
1 1 1 1 3 1 1 1
ppm

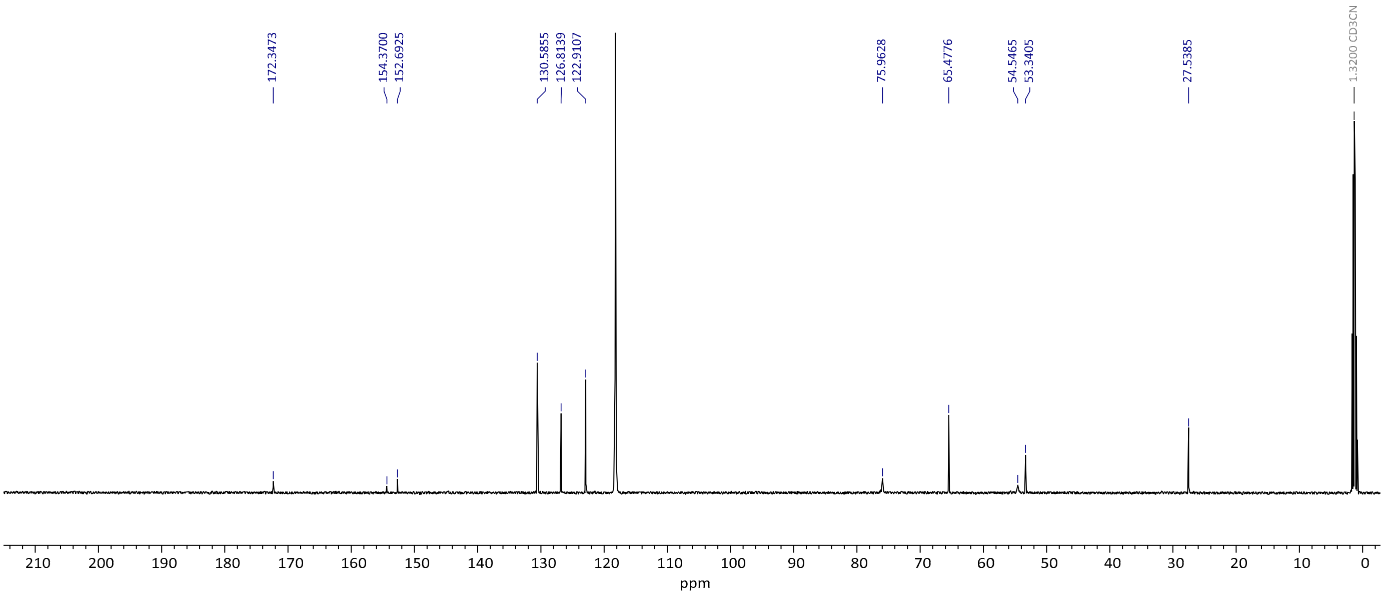
172.3473
154.3700
152.6925
130.5855
126.8139
122.9107
75.9628
65.4776
54.5465
53.3405
27.5385
1.3200 CD3CN
ppm
